# A series of spontaneously blinking dyes for super-resolution microscopy

**DOI:** 10.1038/s41592-026-03062-5

**Published:** 2026-04-15

**Authors:** Katie L. Holland, Sarah E. Plutkis, Brian P. English, Timothy A. Daugird, Abhishek Sau, Jonathan B. Grimm, Qinsi Zheng, Ankith Sharma, Sandeep Dave, Anja Schmidt, Fariha Rahman, Liangqi Xie, Peng Dong, Ariana N. Tkachuk, Timothy A. Brown, Robert H. Singer, Zhe J. Liu, Siegfried M. Musser, Wesley R. Legant, Catherine G. Galbraith, Luke D. Lavis

**Affiliations:** 1 Janelia Research Campus, Howard Hughes Medical Institute, Ashburn, VA USA; 2 Department of Pharmacology, University of North Carolina-Chapel Hill Medical School, Chapel Hill, NC USA; 3 Department of Cell Biology and Genetics, Texas A&M University, College of Medicine, College Station, TX USA; 4 Lampe Joint Department of Biomedical Engineering, University of North Carolina-Chapel Hill and North Carolina State University, Chapel Hill, NC USA; 5 Department of Anatomy and Structural Biology, Albert Einstein College of Medicine, Bronx, NY USA; 6 Department of Biomedical Engineering and Knight Cancer Institute, Oregon Health and Science University, Portland, OR USA

**Keywords:** Chemical tools, Super-resolution microscopy, Chromatin structure

## Abstract

Spontaneously blinking fluorophores toggle between nonfluorescent and fluorescent forms without caging groups or redox buffers, enabling super-resolution imaging. The intrinsic blinking of such dyes is governed by molecular structure and modulated by environment; there is no one-size-fits-all fluorophore suitable for every imaging context. We report dyes with tuned on:off ratios that enable single-molecule localization microscopy and super-resolution optical fluctuation imaging of biomolecular structures in vitro and in cells.

## Abstract (web summary)

A series of spontaneously blinking dyes in the far-red range facilitate single-molecule localization microscopy. These dyes vary in their blinking properties and can be matched to the applications and microscopy modalities as needed.

## Main

**Fig. 1:**
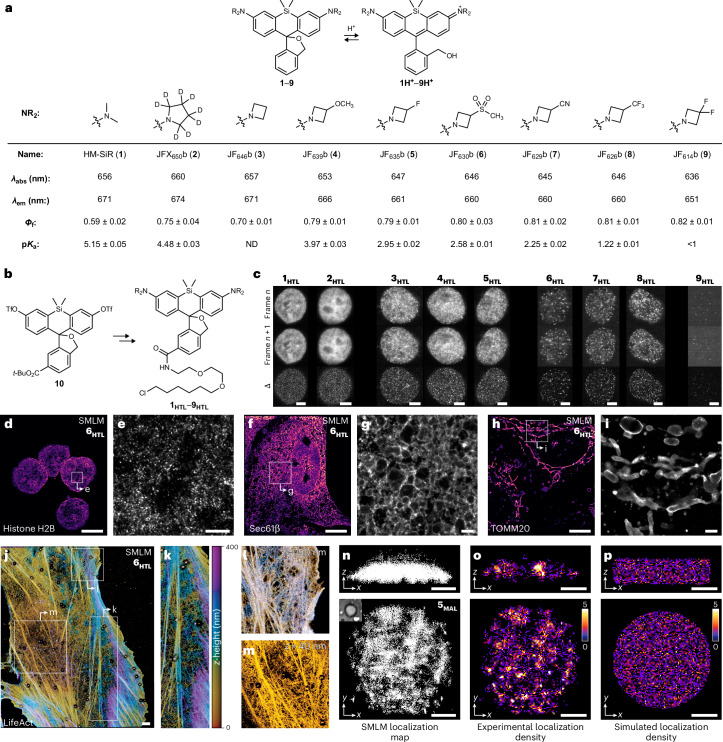
Characterization of spontaneously blinking fluorophores and their use in SMLM. **a**, Structure, spectral properties, and p*K*_a_ values for compounds **1**–**9**; *λ*_abs_, *λ*_em_ and *Φ*_f_ measured in 10 mM citrate buffer, pH 3.0, containing 150 mM NaCl and 0.1% (w/v) SDS; *Φ*_f_ is reported as ±s.d. (*n* = 2) and p*K*_a_ is reported as ±95% CI from curve fitting (*n* = 2). **b**, Synthesis of HaloTag ligands **1**_**HTL**_–**9**_**HTL**_ from ditriflate **10**. **c**, Two subsequent imaging frames (25 ms exposure time) and the difference between them (Δ) from SMLM imaging sessions in paraformaldehyde-fixed COS-7 cells expressing HaloTag–histone H2B fusion proteins labeled to saturation with ligands **1**_**HTL**_–**9**_**HTL**_; contrast was adjusted to provide maximal dynamic range for each fluorophore; scale bars, 5 μm. **d–i**, SMLM images of paraformaldehyde-fixed cells expressing HaloTag fused to histone H2B (**d**,**e**; COS-7 cells), Sec61β (**f,g**; U2OS cells) or TOMM20 (**h**,**i**; U2OS cells) and labeled with JF_630_b–HaloTag ligand (**6**_**HTL**_); localization precision (*σ*; mean ± s.d.): 13.0 ± 5.8 nm (**d**), 10.7 ± 2.9 nm (**f**) and 13.0 ± 5.5 nm (**h**); scale bars, 10 μm (**d**,**f**,**h**) and 1 μm (**e**,**g**,**i**). **j–m**, 3D SMLM (iPALM) image of a paraformaldehyde-fixed U2OS cell expressing HaloTag–LifeAct labeled with JF_635_b-HTL (**5**_**HTL**_). **n**,**o**, 3D SMLM images of a FUS coacervate aged for 12 h containing 2 nM FUS^A2C^–**5**_**MAL**_. Map of total localizations (*n* = 32,528); scale bars, 1 μm; brightfield image inset; scale bar, 2 μm (**n**). 2D localization density distributions (4,101 localizations per μm^3^) of 100-nm thick *x*–*y* or *x*–*z* slices; scale bars, 1 μm (**o**). **p**, Simulated coacervate with random localizations at the same density as in **n**,**o**; scale bars, 1 μm. All imaging experiments were repeated with similar results. Source data

Single-molecule localization microscopy (SMLM) and super-resolution optical fluctuation imaging (SOFI) bypass the diffraction limit by temporally separating individual emitters via stochastic activation and localization or by computationally exploiting intrinsic fluorescence intensity fluctuations of a given fluorophore^1–5^. Spontaneously blinking fluorophores^6–9^ such as hydroxymethyl-Si-rhodamine (HM-SiR^10^; **1**; Fig. 1a), simplify these techniques by eliminating the need for photoactivatable dyes^11^ or redox buffers^12^. Unfortunately, **1** exhibits an on:off ratio too high for general super-resolution microscopy. Although this limitation has been acknowledged and addressed by imaging ordered structures^10,13,14^, exploiting specialized environments^15,16^ or developing bespoke HM-SiR derivatives and analogs^6–9^, the field lacks systematically tuned dyes that accommodate diverse biological samples and techniques. This work describes a series of spontaneously blinking Janelia Fluor (JF) derivatives with on:off ratios that span two orders of magnitude, enabling subdiffraction imaging of different biomolecular structures in vitro and in cells.

**Extended Data Fig. 1:**
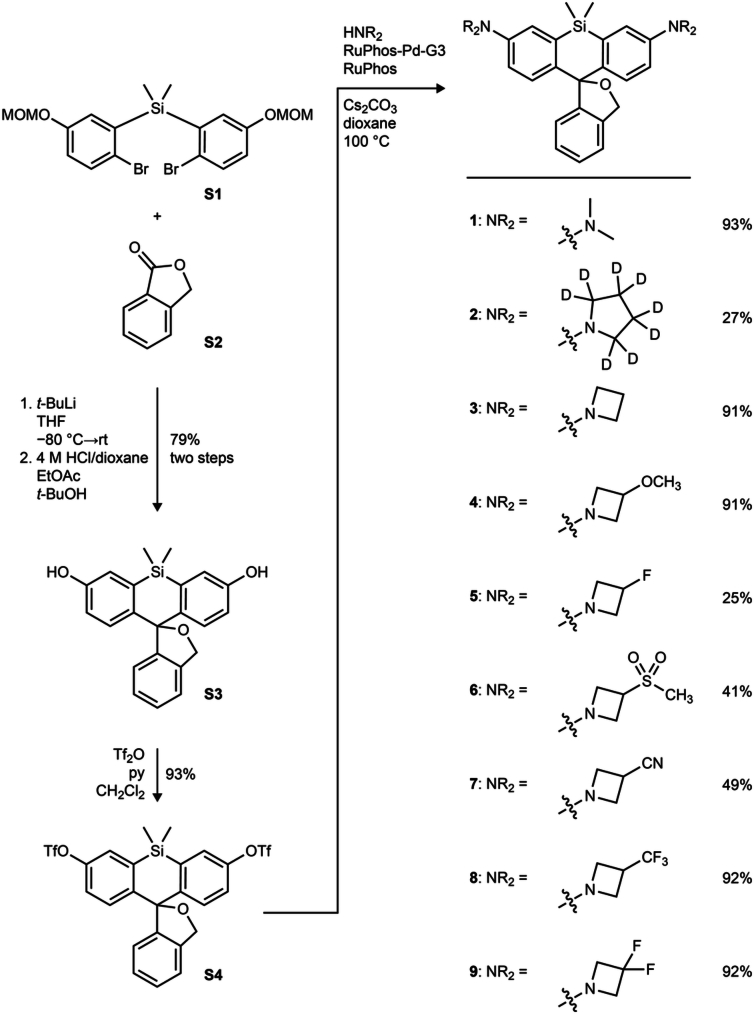
Synthesis of free dyes 1–9. Divergent route to compounds **1**–**9** using Pd-catalyzed cross-coupling.

**Extended Data Fig. 2:**
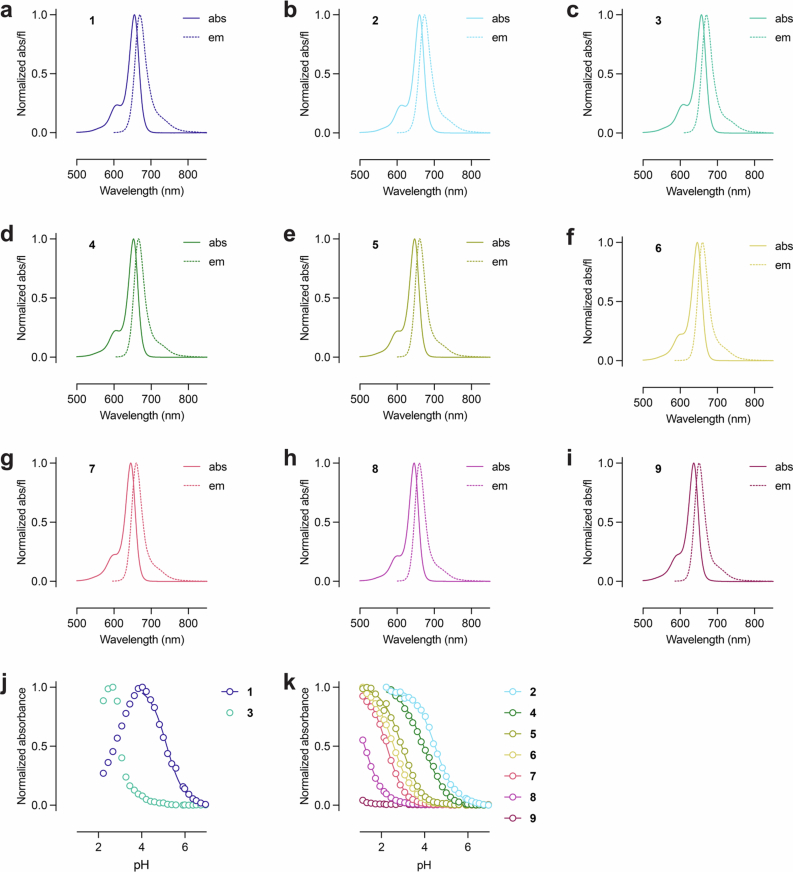
Spectral properties and pH titrations of 1–9. (**a–i**) Normalized absorbance and fluorescence emission of compounds **1**–**10** in 10 mM citrate buffer, pH 3.0 containing 150 mM NaCl and 0.1% (w/v) SDS. (**j,k**) Plots of normalized absorbance *vs*. pH to determine p*K*_a_ (mean values shown; *n* = 2 technical replicates). Source data

**Extended Data Fig. 3:**
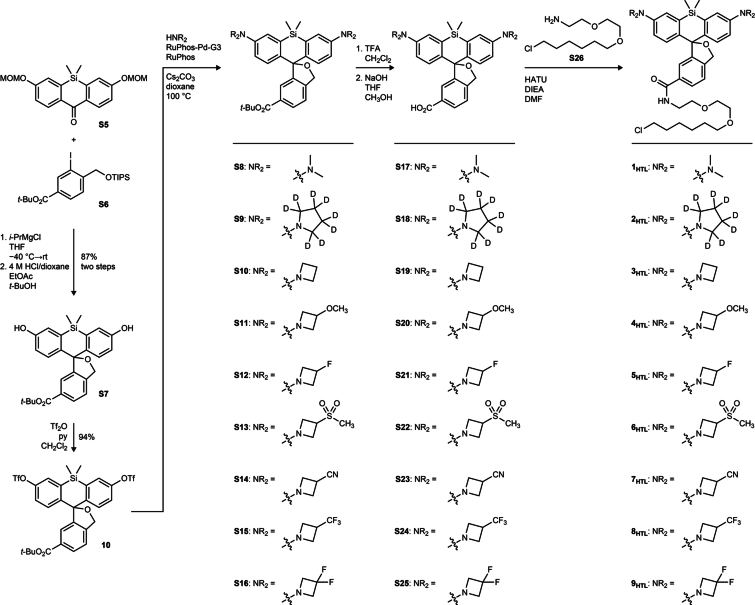
Synthesis of HaloTag ligands 1_HTL_–9_HTL_. Divergent route to compounds **1**_**HTL**_ –**9**_**HTL**_ using Pd-catalyzed cross-coupling.

Dyes **2**–**9** were synthesized in a divergent fashion (Fig. 1a, Extended Data Fig. 1 and Supplementary Note 1) and named after their parent fluorophores^17–21^—JFX_650_, JF_646_, JF_639_, JF_635_, JF_630_, JF_629_, JF_626_ and JF_614_—with a ‘b’ after the name to indicate their blinking character. The spectral and chemical properties of dyes **1**–**9** were evaluated at pH 3 with 0.1% sodium dodecyl sulfate (SDS) to promote the fluorescent form^6,22^ (Fig. 1a). HM-SiR (**1**) exhibited an absorption maximum (*λ*_abs_) of 656 nm, an emission maximum (*λ*_em_) of 671 nm and a fluorescence quantum yield (*Φ*_f_) of 0.59. The azetidine^17–19^ and deuterated pyrrolidine^20^ substituents in compounds **2**–**9** resulted in higher *Φ*_f_ values (0.70–0.82) and *λ*_abs_/*λ*_em_ values ranging from 660 nm/674 nm for JFX_650_b (**2**) to 636 nm/651 nm for JF_614_b (**9**; Extended Data Fig. 2a–i). The pH titration of **1** was complex^9^, with dye absorbance peaking at pH ≈ 4 and then decreasing, presumably due to protonation of the nitrogen atoms, giving an apparent p*K*_a_ = 5.15 (Extended Data Fig. 2j). JF_646_b (**3**) also exhibited a complicated pH titration (Extended Data Fig. 2j) but the other dyes (**2** and **4**–**9**) showed monotonic responses yielding p*K*_a_ values correlated with the electron-withdrawing character of the auxochromes (Fig. 1a and Extended Data Fig. 2k). Having demonstrated that the p*K*_a_ values of spontaneously blinking dyes could be systematically tuned, the HaloTag ligands **1**_**HTL**_–**9**_**HTL**_ were synthesized from Si-fluorescein ditriflate **10** (Fig. 1b, Extended Data Fig. 3 and Supplementary Note 2).

**Extended Data Fig. 4:**
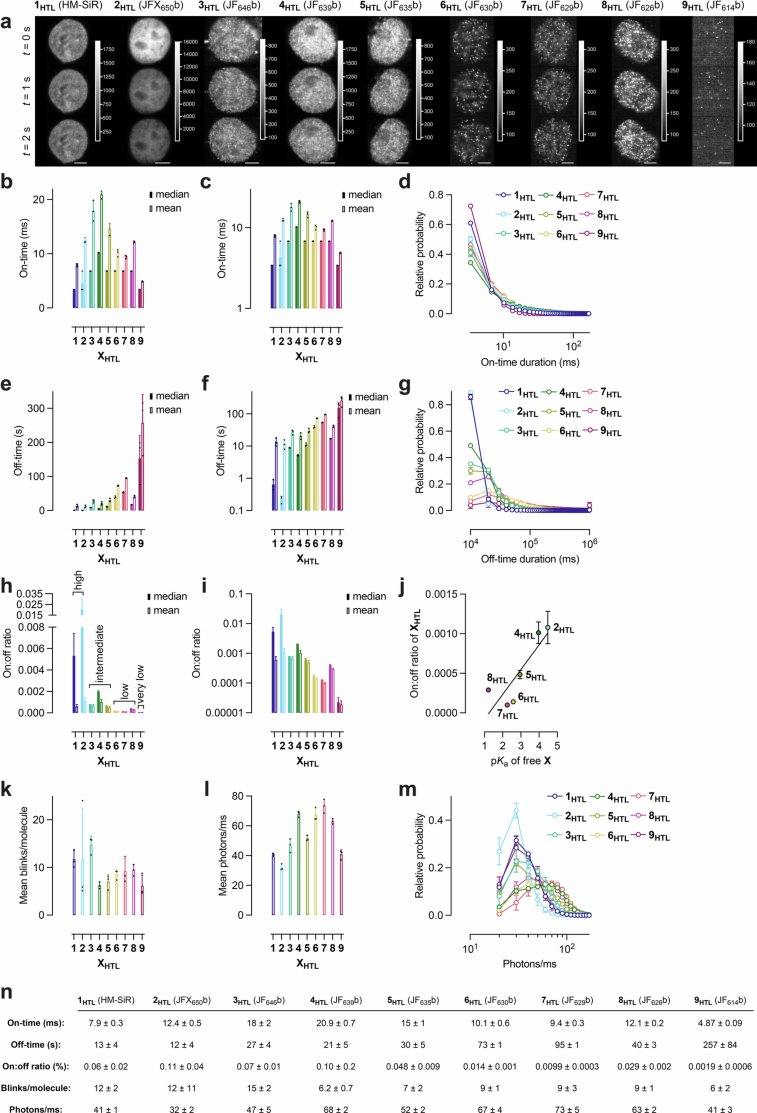
Characterization of 1_HTL_–9_HTL_ for SMLM. (**a**) Individual frames with intensity scales from an SMLM imaging session using formaldehyde-fixed COS-7 cells expressing histone H2B–HaloTag labeled to saturation with HaloTag ligands **1**_**HTL**_–**9**_**HTL**_; scale bars: 5 μm; imaging experiments were repeated with similar results. (**b,c**) Mean and median on-times for **1**_**HTL**_–**9**_**HTL**_ plotted on a linear- (**b**) and log-scale (**c**) ordinate; error bars indicate ±s.d. (**d**) Probability *vs*. on-time (ms; semi-log plot) for **1**_**HTL**_–**9**_**HTL**_; error bars indicate ±s.d. (**e,f**) Mean and median off-times for **1**_**HTL**_–**9**_**HTL**_ plotted on a linear- (**e**) and log-scale (**f**) ordinate; error bars indicate ±s.d. (**g**) Probability *vs*. off-time (ms; semi-log plot) for **1**_**HTL**_–**9**_**HTL**_; error bars indicate ±s.d. (**h,i**) Mean and median on:off ratios for **1**_**HTL**_–**9**_**HTL**_ plotted on a linear- (**h**) and log-scale (**i**) ordinate; error bars indicate ±s.d. (**j**) Plot of on:off ratio of **1**_**HTL**_–**9**_**HTL**_ *vs*. the p*K*_a_ of the parent free dyes **1**–**9**; error bars indicate ±s.e.m; R^2^ = 0.65. (**k**) Mean number of blinks per molecule for **1**_**HTL**_–**9**_**HTL**_; error bars indicate ±s.d. (**l**) Mean photon emission rate for **1**_**HTL**_–**9**_**HTL**_; error bars indicate ± s.d. (**m**) Probability *vs*. photons/ms (semi-log plot) for **1**_**HTL**_–**9**_**HTL**_; error bars indicate ±s.d. (**n**) Summary table with on-times, off-times, on:off ratio, blinks/molecule, and photons/ms for for **1**_**HTL**_–**9**_**HTL**_ (mean±s.d.). Parameters in **b**–**n** were calculated from *n* = 3 independent cellular imaging experiments at subsaturating labeling densities. Off-time determinations used ~100-fold lower concentrations compared to on-time determinations and grouped localizations within 100 nm over a 45 min acquisition window; the off-times represent upper-bound dark-state lifetimes under these conditions. Source data

Ligands **1**_**HTL**_–**9**_**HTL**_ were tested in fixed cells expressing HaloTag–histone H2B (Fig. 1c, Extended Data Fig. 4a and Supplementary Video 1). Examination of two adjacent frames and the difference between them (Δ; Fig. 1c) allowed qualitative categorization of the on:off ratios: HM-SiR, and JFX_650_b (**1**_**HTL**_–**2**_**HTL**_) as high; JF_646_b, JF_639_b and JF_635_b (**3**_**HTL**_–**5**_**HTL**_) as intermediate; JF_630_b, JF_629_b and JF_626_b (**6**_**HTL**_–**8**_**HTL**_) as low; and JF_614_b (**9**_**HTL**_) as very low. Quantitative analysis of these datasets revealed that electron-withdrawing substituents modestly attenuated on-times (Extended Data Fig. 4b–d) but substantially increased off-times (Extended Data Fig. 4e–g) yielding dyes with lower on:off ratios ranging from ~10^−2^ (JFX_650_b) to ~10^−4^ (JF_614_b; Extended Data Fig. 4h,i). The correlation of the p*K*_a_ values of the parent dyes and on:off ratios of their respective ligands is imperfect (Extended Data Fig. 4j), suggesting that HaloTag conjugation alters blinking in a dye-specific way^9^. Blinks per molecule varied by ~twofold across the series and the photon rate for **2**_**HTL**_–**9**_**HTL**_ was generally higher than **1**_**HTL**_, reflecting the improved *Φ*_f_ (Fig. 1a and Extended Data Fig. 4k–m). In summary, ligands **1**_**HTL**_–**9**_**HTL**_ exhibit similar on-times and blinks per molecule but trend to higher brightness, longer off-times and lower on:off ratios across the series (Extended Data Fig. 4n).

**Extended Data Fig. 5:**
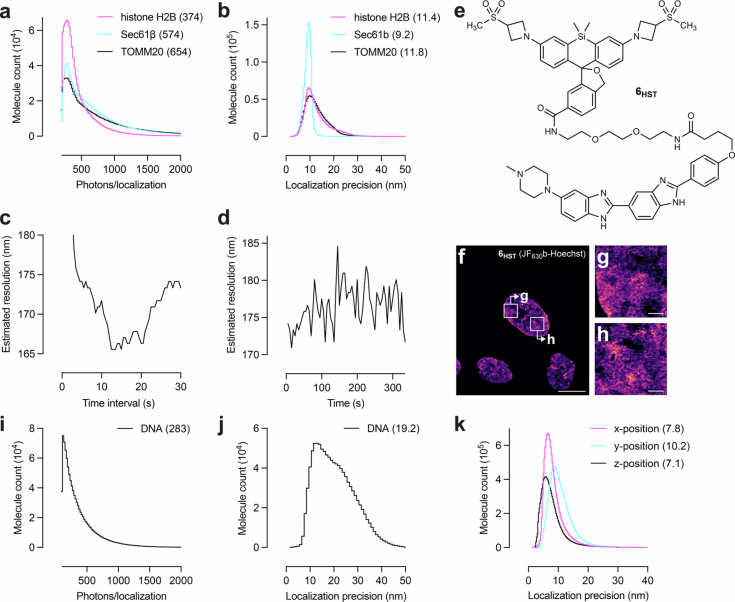
Statistics of SMLM imaging. (**a**) Molecule count *vs*. photons/localization of **6**_**HTL**_ in formaldehyde-fixed cells expressing histone H2B–HaloTag, HaloTag–Sec61β, or TOMM20–HaloTag; numbers in parentheses indicate median values in photons. (**b**) Probability *vs*. localization precision of **6**_**HTL**_ in formaldehyde-fixed cells expressing histone H2B–HaloTag, HaloTag–Sec61β, or TOMM20–HaloTag; numbers in parentheses indicate median values in nm. (**c**) Estimated resolution *vs*. time interval calculated by Fourier ring analysis for live-cell SMLM imaging of cells expressing HaloTag–Sec61β and labeled with **6**_**HTL**_ (**Supplementary Video 2**). (**d**) Estimated resolution *vs*. time calculated by Fourier ring analysis for live-cell SMLM imaging of cells expressing HaloTag–Sec61β and labeled with **6**_**HTL**_ (**Supplementary Video 2**). (**e**) Chemical structure of JF_630_-Hoechst (**6**_**HST**_). (**f–h**) SMLM images of paraformaldehyde-fixed hTERT RPE-1 cells labeled with **6**_**HST**_; scale bars: 2 μm. (**i**) Probability *vs*. photons/localization of **6**_**HST**_ in formaldehyde-fixed cells; number in parentheses indicates median values in photons. (**j**) Probability *vs*. localization precision of **6**_**HST**_ in formaldehyde-fixed cells; numbers in parentheses indicates median values in nm. (**k**) Probability *vs*. localization precision of **4**_**HTL**_ in formaldehyde-fixed cells expressing LifeAct**–**HaloTag and imaged using 3D SMLM (iPALM); numbers in parentheses indicates median values in nm for each of the three dimensions. All imaging experiments were repeated with similar results. Source data

Dyes with low or intermediate on:off ratios enabled SMLM imaging. Fixed cells expressing HaloTag fusions of histone H2B (Fig. 1d,e), Sec61β (Fig. 1f,g) or TOMM20 (Fig. 1h,i) were labeled with JF_630_b–HaloTag ligand (**6**_**HTL**_), providing images with high localization precisions (*σ* ≤ 13 nm; Extended Data Fig. 5a,b). Live-cell SMLM was also performed using HaloTag–Sec61β conjugated to **6**_**HTL**_ (Supplementary Video 2), giving subdiffraction dynamic imaging (Extended Data Fig. 5c,d). The Hoechst conjugate of JF_630_b (**6**_**HST**_) allowed super-resolution imaging of native chromatin (*σ* = 19 nm; Extended Data Fig. 5e–j). JF_635_b-based **5**_**HTL**_ enabled 3D iPALM imaging in cells expressing LifeAct–HaloTag with *σ* < 12 nm in all three dimensions (Fig. 1j–m and Extended Data Fig. 5k).

**Extended Data Fig. 6:**
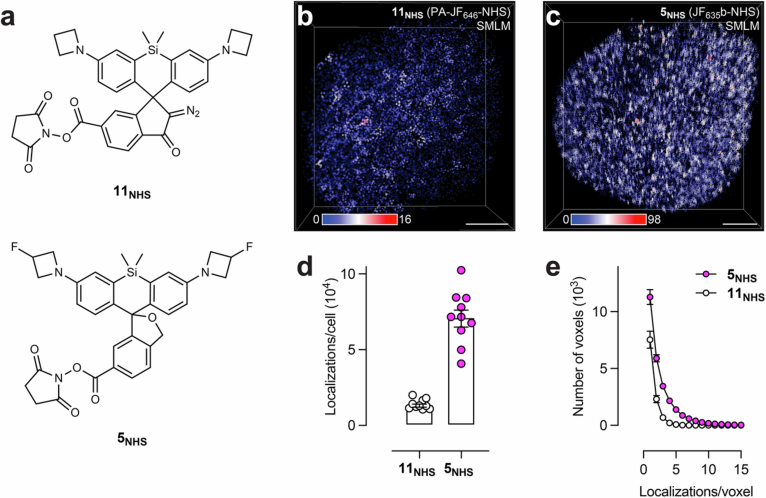
Comparison of PA-JF_646_ and JF_635_b for super-resolution ATAC experiments. (**a**) Structures of PA-JF_646_-NHS (**11**_**NHS**_) and JF_635_b-NHS (**5**_**NHS**_). (**b,c**) Representative 3D ATAC–SMLM rendering of accessible chromatin localizations in wild-type mouse embryonic stem (mES) cells using oligonucleotide probes labeled with **11**_**NHS**_ (**b**) or **5**_**NHS**_ (**c**); the color-coded localization density was calculated with a canopy radius of 250 nm; scale bars: 2 µm. (**d**) Localizations per cell for ATAC–SMLM experiments using oligonucleotides labeled with **11**_**NHS**_ or **5**_**NHS**_; error bars indicate ±s.e.m. (*n* = 10 cells). (**e**) Plot of number of 100 nm×100 nm×100 nm voxels *vs*. localizations per 100 nm×100 nm×100 nm voxel for ATAC–SMLM experiments using oligonucleotides labeled with **11**_**NHS**_ or **5**_**NHS**_; error bars indicate ±s.e.m. (*n* = 10 cells). Source data

**Extended Data Fig. 7:**
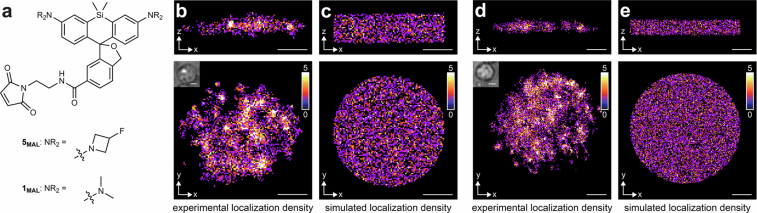
Characterization of FUS condensates. (**a**) Structures of JF_635_b-maleimide (**5**_**MAL**_) and HM-SiR-maleimide (**1**_**MAL**_). (**b**) 2D localization density distributions (4,028 localizations/μm^3^) of 100 nm thick x–y or x–z slices from a 3D SMLM image of a FUS coacervate aged for 12 h containing 2 nM FUS^A2C^–**5**_**MAL**_; (*N* = 21,731); scale bar: 1 μm; brightfield image inset; scale bar: 2 μm. (**c**) Simulated coacervate with random localizations at the same density as **b**; scale bar: 1 μm. (**d**) 2D localization density distributions (4,086 localizations/μm^3^) of 100 nm thick x–y or x–z slices from a 3D SMLM image of a FUS coacervate aged for 12 h containing 2 nM FUS^A2C^–**5**_**MAL**_; (*N* = 44,127); scale bar: 1 μm; brightfield image inset; scale bar: 2 μm. (**e**) Simulated coacervate with random localizations at the same density as **d**; scale bar: 1 μm.

Direct attachment of blinking dyes to biomolecules facilitated additional SMLM experiments. Oligonucleotides labeled with JF_635_b-*N*-hydroxysuccinimidyl ester (**5**_**NHS**_) or photoactivatable JF_646_-*N*-hydroxysuccinimidyl ester (PA-JF_646_-NHS, **11**_**NHS**_; Extended Data Fig. 6a) enabled super-resolution assays of transposase-accessible chromatin (super-resolution ATAC; Extended Data Fig. 6b,c)^23^. JF_635_b simplified imaging by eliminating photoactivation and provided sixfold higher localizations compared to PA-JF_646_ (Extended Data Fig. 6d,e), consistent with the number of blinks per molecule (Extended Data Fig. 4k). The A2C variant of the fused in sarcoma (FUS) protein was labeled with either HM-SiR-maleimide (**1**_**MAL**_) or JF_635_b-maleimide (**5**_**MAL**_; Extended Data Fig. 7a) and biomolecular condensates (BMCs) were formed from wild-type FUS doped with a 0.03% mole fraction of the conjugated protein. The higher on:off ratio of HM-SiR prevented reliable single-molecule detection, but the lower on:off ratio of JF_635_b allowed localization of individual molecules throughout the imaging experiment (Supplementary Video 3). SMLM analysis revealed non-uniform spatial distributions of localizations within the condensates, differing from random distributions (Fig. 1n–p and Extended Data Fig. 7b–e), demonstrating the potential of spontaneously blinking dyes for super-resolution imaging of BMCs. Although this heterogeneity aligns with recent reports^24–26^, the molecular basis of these patterns—molecular density, pH^25^ or polarity^24,26^—remains to be elucidated.

**Fig. 2:**
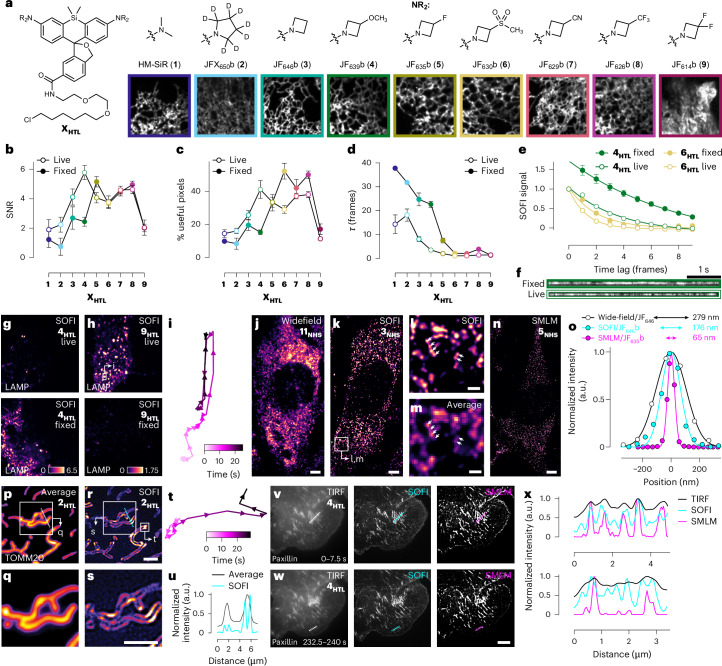
Spontaneously blinking fluorophores for SOFI. **a**, Structures of **1**_**HTL**_–**9**_**HTL**_ with corresponding live-cell SOFI images from 200 frames (2 s at 100 Hz) of U2OS cells expressing HaloTag–Sec61β; images are 252 × 252 SOFI pixels (12.3 × 12.3 μm). **b**–**d**, SOFI statistics from live and fixed cells from SOFIevaluator^4^; 10,000 frames (100 s at 100 Hz); error bars indicate ±s.e.m. (*n* = 4 cells). Mean SNR (**b**). Mean % useful pixels (**c**). Mean *τ* (**d**). **e**, SOFI decorrelation curves for **4**_**HTL**_ and **6**_**HTL**_ from live and fixed-cell experiments; error bars indicate ±s.e.m. (*n* = 4 cells). **f**, Kymographs from live and fixed cells labeled with **4**_**HTL**_. **g**,**h**, SOFI image of U2OS cells expressing HaloTag–LAMP and labeled with **4**_**HTL**_ (**g**) or **9**_**HTL**_ (**h**) in live cells (top) or fixed cells (bottom); length of intensity scale bar, 5 μm. **i**, Track of the individual lysosome indicated in **h**; length of time scale bar, 500 nm. **j**, Wide-field RNA-FISH image of mouse embryonic fibroblasts (MEFs) expressing MS2 in the 3′ UTR of the β-actin gene and labeled with JF_646_-oligonucleotide from **11**_**NHS**_; the image is from a single frame; scale bar, 5 μm. **k**, SOFI RNA-FISH image of MEF cells expressing MS2 in the 3′ UTR of the β-actin gene and labeled with JF_646_b-oligonucleotide from **5**_**NHS**_; scale bar, 5 μm. **l**,**m**, Zoomed area from **k** rendered as a SOFI image (**l**) or average intensity projection image (**m**); scale bars, 1 μm. **n**, SMLM RNA-FISH image of MEF cells expressing MS2 in the 3′ UTR of the β-actin gene and labeled with JF_635_b-oligonucleotide from **6**_**NHS**_; scale bar, 5 μm; images in **k**–**n** were acquired using highly inclined and laminated optical sheet (HILO) illumination with 30-ms exposure time (33.33 Hz). **o**, Normalized intensity profiles, Gaussian fits and full width half maximum measurements of RNA foci from RNA-FISH experiments using JF_646_/confocal (*n* = 3), JF_646_b/SOFI (*n* = 4) or JF_635_b/SMLM (*n* = 3). **p**–**u**, Data from live U2OS cells expressing TOMM20–HaloTag and labeled with JFX_650_b–HaloTag ligand (**2**_**HTL**_); scale bars, 5 μm. Average intensity projection TIRF image (**p**,**q**). SOFI image (**r**,**s**). Trajectory of the mitochondrial edge indicated in **r**; length of time scale bar, 2 μm (**t**). Normalized intensity profile of line scan indicated in **p** and **r** (**u**). **v**,**w**, Data from live CHOK1 cells expressing HaloTag–paxillin and labeled with JF_639_b–HaloTag ligand (**4**_**HTL**_) processed as average TIRF, SOFI and SMLM; images were created from 500 frames of single-molecule data (7.5 s at 66.7 Hz) at the beginning (**v**) or end (**w**) of the 240 s video; scale bars, 5 μm. **x**, Line scan intensity traces of positions shown in **v**,**w** that span multiple adhesion complexes formed by aggregation of paxillin distal or proximal to the cell edge. All imaging experiments were repeated with similar results. Source data

These ligands were then tested in SOFI^2–5^, which requires measurable intensity fluctuations that depend on the blinking parameters of the dye, the labeling density and the imaging speed. SOFI analysis can be performed on <1,000 frames, enabling super-resolution dynamic imaging. Sec61β–HaloTag-expressing cells were labeled with **1**_**HTL**_–**9**_**HTL**_ and SOFI was performed in both fixed and live (Fig. 2a) samples. SOFIevaluator^4^ quantified the signal-to-noise ratio (SNR; Fig. 2b) and % useful pixels (Fig. 2c). In fixed cells, ligands with low on:off ratios (**6**_**HTL**_–**8**_**HTL**_) performed best; JF_635_b–HaloTag ligand (**5**_**HTL**_) also exhibited high SNR. In live cells, dyes with intermediate on:off ratios, such as **4**_**HTL**_, excelled, suggesting blinking properties are different in fixed and live samples. Ligands with high (**1**_**HTL**_–**2**_**HTL**_) or very low (**9**_**HTL**_) on:off ratios showed poor SOFI performance.

**Extended Data Fig. 8:**
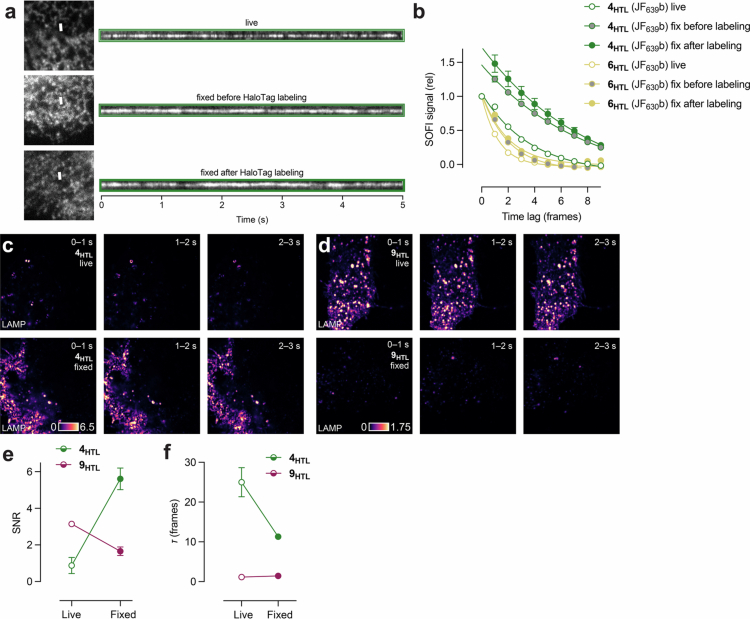
Super-resolution optical fluctuation imaging (SOFI) in live cells. (**a**) Kymographs showing blinking of JF_639_b in cells expressing Sec61β-HaloTag labeled and imaged live, labeled and imaged fixed, or labeled live and imaged fixed. (**b**) SOFI decorrelation curves for **4**_**HTL**_ and **6**_**HTL**_ from live and fixed cell experiments; error bars indicate ±s.e.m. (*n* = 4 cells). (**c,d**) SOFI images at selected time points of U2OS cells expressing HaloTag–LAMP and labeled with **4**_**HTL**_ (**c**) or **9**_**HTL**_ (**d**) in live (top) or fixed (bottom) cells; each frame was created from 1 s of single-molecule imaging data (100 frames at 0.01s/frame); scale bars: 5 μm. (**e**) SOFI SNR value calculated from images of live and fixed samples in **c** and **d**; error bars indicate ±s.e.m. (*n* = 5 cells). (**f**) SOFI *τ* value calculated from images of live and fixed samples in **c** and **d**; error bars indicate ±s.e.m. (*n* = 5 cells). All imaging experiments were repeated with similar results. Source data

SOFI performance was further compared using *τ*, the blinking period of the label calculated from exponential fitting of the decorrelation curves^4^. In fixed cells, the *τ* values for **1**_**HTL**_ to **9**_**HTL**_ decreased with an asymptote at ~one frame (Fig. 2d). This is consistent with the trends in fluorophore properties; dyes with higher on:off ratios give lower effective blinking periods in SOFI due to overlapping signals. Dyes with lower on:off ratios show *τ* ≈ 1 frame due to minimal signal overlap and the matched imaging frame rate (100 Hz) and on-time (~10 ms; Extended Data Fig. 4b–d,n). Live cells show lower *τ* values, especially for dye ligands **1**_**HTL**_–**5**_**HTL**_ (Fig. 2d), consistent with lower on:off ratios, leading to shorter effective blinking periods. Decorrelation curves from samples labeled with JF_639_b–HaloTag ligand (**4**_**HTL**_) and JF_630_b–HaloTag ligand (**6**_**HTL**_; Fig. 2e) and kymographs from experiments using **4**_**HTL**_ confirmed blinking changes upon fixation (Fig. 2f and Extended Data Fig. 8a,b). In summary, the *τ* metric revealed substantial differences in blinking properties in fixed versus live samples for dye ligands with higher on:off ratios such as JF_639_b (**4**), explaining the improved live-cell SOFI performance (Fig. 2b,c). This could stem from modification of a specific residue proximal to the dye binding site, such as Lys106 (ref. ^21^), or more general changes in HaloTag structure elicited by aldehyde fixation. In summary, the *τ* metric (Fig. 2d) is a key indicator of SOFI performance in addition to SNR and % useful pixels (Fig. 2b,c).

To assess utility in acidic compartments, cells expressing HaloTag–CD63 and labeled with either JF_639_b–HaloTag ligand (**4**_**HTL**_), an optimal dye for live-cell SOFI of cytosolic proteins (Fig. 2b–d) or JF_614_b–HaloTag ligand (**9**_**HTL**_), the dye with the lowest on:off ratio (Extended Data Fig. 4h,i). JF_639_b showed no appreciable SOFI signal (Fig. 2g) as the acidic environment of the lysosome promotes the fluorescent form, thereby reducing blinking. This pH-driven increase of on:off ratio is beneficial for JF_614_b, allowing SOFI (Fig. 2h) and tracking of individual lysosomes over time (Fig. 2i). Fixation neutralizes lysosomes, switching the performance of the dyes: JF_639_b could be used for SOFI but JF_614_b failed (Fig. 2g,h). The fixed-cell experiments using JF_639_b–HaloTag ligand (**4**_**HTL**_) yielded higher SNR and lower *τ* compared to the Sec61β experiments (Fig. 2b,d and Extended Data Fig. 8c–f), likely from decreased labeling density, resulting in a lower effective blinking period and improved SOFI.

**Extended Data Fig. 9:**
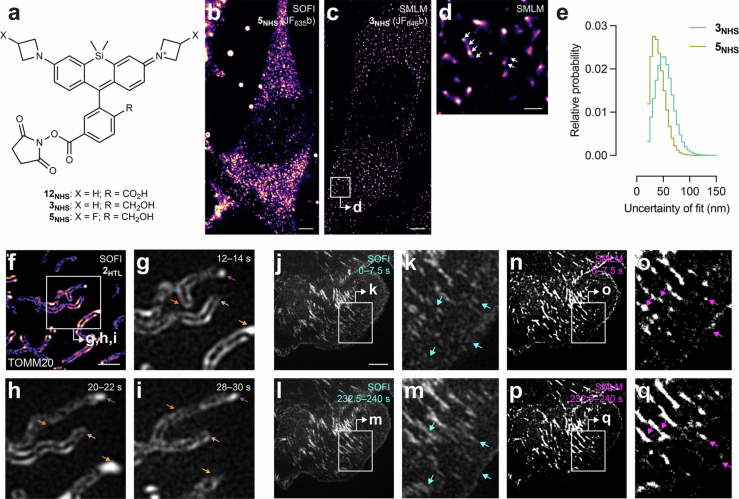
Comparison of SMLM and SOFI. **a**) Structures of JF_646_-NHS (**12**_**NHS**_), JF_646_b-NHS (**3**_**NHS**_), and JF_635_b-NHS (**5**_**NHS**_). (**b**) SOFI RNA-FISH image of MEFs expressing MS2 in the 3′ UTR of the beta-actin gene and labeled with JF_635_b-oligonucleotide prepared using **5**_**NHS**_; scale bar: 5 μm. (**c**) SMLM RNA-FISH image of MEFs expressing MS2 in the 3′ UTR of the beta-actin gene and labeled with JF_646_b-oligonucleotide prepared using **3**_**NHS**_; scale bar: 5 μm. (**d**) Zoomed area from **c** rendered as a SMLM image; *cf*. **Fig. 2l,m**; scale bar: 1 μm. (**e**) Relative probability *vs*. uncertainty of fit from SMLM images shown in **Fig. 2n** and panel **c**, calculated using 2,219,274 detections for the JF_646_b-oligonucleotide from **3**_**NHS**_, and 2,257,059 detections for JF_635_b-oligonucleotide from **5**_**NHS**_. Images in **c,d** were taken using HILO illumination with 30 ms exposure time (33.33 Hz). (**f–i**) Data from live U2OS cells expressing HaloTag–TOMM20 and labeled with JF_639_b–HaloTag ligand (**1**_**HTL**_) processed linearized second-order SOFI; initial frame (**f**) and time-lapse imaging (**g–i**); arrows highlight specific morphology changes in the high-resolution dynamic imaging set. (**j–q**) Data from live CHOK1 cells expressing HaloTag–paxillin and labeled with JF_639_b–HaloTag ligand (**4**_**HTL**_) processed as SOFI (**j–m**) or SMLM (**n–q**); images, created from 500 frames of single-molecule images (7.5 s at 66.7 Hz) at the beginning (**j,k,n,o**) or end (**l,m,p,q**) of the 240 s movie; arrows highlight changes in adhesion position between timepoints; scale bars: 5 μm. All imaging experiments were repeated with similar results. Source data

The spatial resolution of SOFI was evaluated in RNA fluorescence in situ hybridization (RNA-FISH). Amine-terminated oligonucleotides targeting the RNA sequence that binds the bacteriophage MS2 coat protein were labeled with nonblinking label JF_646_-*N*-hydroxysuccinimidyl ester^17^ (JF_646_-NHS; **12**_**NHS**_), JF_646_b-NHS (**3**_**NHS**_) or JF_635_b-NHS (**5**_**NHS**_; Extended Data Fig. 9a and Supplementary Note 2). These probes were used in RNA-FISH experiments with cells derived from a transgenic mouse containing an MS2 binding site in the 3′ untranslated region (UTR) of the β-actin gene^27^. Wide-field imaging experiments using JF_646_ gave diffraction-limited resolution of RNA foci (Fig. 2j). Both blinking labels could be visualized using SOFI (Fig. 2k–m and Extended Data Fig. 9b), and JF_635_b proved superior for SMLM due to its lower on:off ratio (Fig. 2n,o and Extended Data Fig. 9c–e).

The resolution of SOFI in live-cell time-lapse imaging experiments was assessed. Cells expressing TOMM20–HaloTag were labeled with JFX_650_b–HaloTag ligand (**2**_**HTL**_) and imaged (125 Hz) using total internal reflection (TIRF) microscopy. The 250 frames were processed to generate a diffraction-limited, average intensity projection image (Fig. 2p,q) or a SOFI image (Fig. 2r,s). SOFI enabled tracking of mitochondria at 0.5 Hz (Fig. 2t and Extended Data Fig. 9f–i) with resolution of the mitochondrial outer edges (Fig. 2u). HaloTag–paxillin-expressing cells were incubated with JF_639_b–HaloTag ligand (**4**_**HTL**_), imaged and processed as average TIRF, SOFI or SMLM images (Fig. 2v,w). Imaging was threefold faster than previous experiments with tdEos–paxillin^28^ with comparable resolution (Supplementary Video 4). SMLM yielded higher resolution compared to average TIRF or SOFI images in the densely labeled focal adhesions in the interior of the cell. In areas with lower labeling density, such as the nascent adhesions near the leading edge, SOFI proved superior for visualizing small aggregates (Fig. 2v–x and Extended Data Fig. 9j–q). These live-cell experiments demonstrate the utility of SOFI for microscopy with high spatiotemporal resolution and its complementarity to SMLM, especially when imaging cellular structures with lower labeling densities.

In summary, a panel of spontaneously blinking fluorophores was synthesized. Minor structural changes exerted substantial effects on p*K*_a_ (Fig. 1a) and blinking properties (Fig. 1c and Extended Data Fig. 4). For SMLM imaging in fixed cells using the HaloTag, low on:off ratio dyes such as JF_630_b provided high quality images (Fig. 1d–i). For localization microscopy experiments using labeled biomolecules, the intermediate on:off ratio JF_635_b enabled SMLM of BMCs (Fig. 1n–p), accessible chromatin (Extended Data Fig. 6) and RNA (Fig. 2n). These compounds also function as effective SOFI labels, but additional dyes are also useful depending on the environment, including JF_639_b for live-cell imaging in the cytosol (Fig. 2b–d,v–x), JF_614_b in acidic vesicles (Fig. 2g–i) and JF_646_b for RNA-FISH (Fig. 2k–m). This broad selection of bright, spontaneously blinking fluorophores will accommodate a range of experiments with diverse labeling strategies, labeling densities and biological environments to facilitate straightforward imaging below the diffraction limit.

## Methods

### Chemical synthesis

The development of an improved synthetic method for Si-fluorescein derivatives including compound **10** is described in Supplementary Note 1 in the Supplementary Information. Experimental details and characterization for the syntheses of **1**–**9**, **1**_**HTL**_–**9**_**HTL**_, **3**_**NHS**_, **5**_**NHS**_, **5**_**MAL**_ and **6**_**HST**_ can be found in Supplementary Note 2 in the Supplementary Information.

### Spectroscopy of free dyes 1–9

Initial attempts to measure the spectral properties of the dyes as HaloTag conjugates were unsuccessful due to instability of the HaloTag protein at the low pH values necessary to promote the visible-absorbing and fluorescent forms of the spontaneously blinking dyes. Instead, the spectral properties of free dyes **1**–**9** were measured in 10 mM citrate buffer, pH 3.0, containing 150 mM NaCl and 0.1% (w/v) SDS. The visible-absorbing form of Si-rhodamines bind to SDS micelles^6^ and these conditions afforded solutions with measurable absorbance and fluorescence across the compound series. All measurements were taken at ambient temperature (22 ± 2 °C) using 1-cm path length, 3.5-ml quartz cuvettes (Starna Cells). Dyes were prepared as stock solutions in dimethylsulfoxide (DMSO) and diluted such that the DMSO concentration was less than 0.1% (v/v). Maximum absorption wavelength (*λ*_abs_) of dyes **1**–**9** was measured on a Cary Model 4000 spectrometer (Agilent). Absorption spectra are averages (*n* = 3) and normalized spectra are shown for clarity. Fluorescence spectra and maximum emission wavelength (*λ*_em_) of dyes **1**–**9** were measured on a Cary Eclipse fluorometer (Agilent); normalized spectra are shown for clarity. Absolute fluorescence quantum yield values (*Φ*_f_) of the HaloTag ligands **1**–**9** were measured using a Quantaurus-QY spectrometer (model C11374, Hamamatsu). Measurements were carried out using dilute samples (*A* < 0.1), and self-absorption corrections were performed using the instrument software^29^. Reported *Φ*_f_ values are averages of measurements from two separate dye solutions. pH titrations were performed using mixtures of HCl and KCl for pH < 2.2 (0.2 M ionic strength) and mixtures of citric acid (0.1 M) and Na_2_HPO_4_ (0.2 M) for pH > 2.2. p*K*_a_ values were determined from curve fitting the absorbance data measured on a FlexStation3 microplate reader (Molecular Devices; *n* = 2).

### Cell culture and plating of cells for super-resolution microscopy

COS-7 (CRL-1651), U2OS (HTB-96), hTERT-immortalized retinal pigment epithelial cells (hTERT RPE-1; CRL-4000) and CHOK1 cells (CCL-61) were obtained from the ATCC. For imaging of cell nuclei using the HaloTag system, COS-7 cells with an integrated HaloTag–histone H2B fusion protein-expressing plasmid via the piggyBac transposon system^30^ were used. For imaging of mitochondria, U2OS cells with an integrated TOMM20–HaloTag fusion protein-expressing plasmid^19^, maintained under the selection of 500 μg ml^−1^ Geneticin (Life Technologies) were used. For imaging the endoplasmic reticulum, U2OS cells stably expressing a HaloTag–Sec61b fusion protein via lentiviral transduction and integration were used. All cell lines underwent regular mycoplasma testing by the Legant Laboratory, the Gailbraith Laboratory or by the Janelia Cell Culture Shared Resource. All cell lines were cultured in growth medium comprising Dulbecco’s modified Eagle medium (DMEM; Thermo Fisher Scientific, 11965118) supplemented with 10% (v/v) fetal bovine serum (FBS; Avantor 1300-500H) and 100 U ml^−1^ penicillin–streptomycin (Thermo Fisher Scientific, 15140122). Cell cultures were maintained in an incubator under standard conditions (37 °C, 5% (v/v) CO_2_). For all imaging experiments, cells were collected and plated 1 day before dye labeling. U2OS cells were plated at a density of 1 × 10^5^ cells per ml and hTERT RPE-1 and COS-7 cells were plated at a density of 2.5 × 10^5^ cells per ml in 6-, 12- or 24-well glass bottomed plates (Cellvis P06-1.5H-N, P12-1.5H-N, P24-1.5H-N).

### Labeling of cells with HaloTag ligands 1_HTL_–9_HTL_

HaloTag ligands were dissolved in anhydrous DMSO (Thermo Fisher Scientific, 50-148-9333) to achieve a final concentration of 1 mM. The ligand stock solution was aliquoted and stored at −20 °C; a freshly thawed aliquot was used immediately for each experiment. The ligand stock solution was diluted into complete growth medium and cells were incubated for 1 h. To achieve complete labeling of HaloTag proteins, 0.5–2 μM dye ligand was used. For the on-time, off-time and on:off ratio measurements, we utilized a serial dilution method to label cells with a range of different concentrations of each dye to achieve a labeling density at which multiple blinks could be unambiguously assigned to single dye molecules. The concentrations of dye ligand used for the off-time measurements were ~100-fold lower than used for the on-time measurements to limit false linking errors. After the 1 h incubation, cells were rinsed once with prewarmed phosphate-buffered saline (PBS; Corning 21-040-CMX12) and then placed in fresh growth medium for 20 min. The growth medium was refreshed after 20 min and incubated for an additional 20 min. For live-cell SMLM imaging, the medium was replaced with FluorBrite DMEM (Thermo Fisher Scientific, A1896701) supplemented with 10% (v/v) FBS (Avantor, 1300-500H) and 100 U ml^−1^ penicillin–streptomycin (Thermo Fisher Scientific, 15140122). For dye characterization experiments in fixed cells, samples were washed once with ambient temperature PBS and fixed in freshly prepared 4% (w/v) paraformaldehyde (Electron Microscopy Sciences, 15710) for 12 min. This was followed by a 10-s wash and 3 × 5-min washes with PBS. Simultaneous with labeling and fixations steps, a solution of wheat germ agglutinin (WGA) conjugated with nanodiamonds (WGA-ND; Adámas, NDNV1000-WGA custom order) was diluted in PBS and sonicated for at least 1 h. Following fixation and wash steps, the cellular samples were incubated in WGA-ND solution for 20 min on a rocker at ambient temperature. This was followed by a 10-s wash and 3 × 5-min washes with PBS. The sample then underwent a secondary fixation step for 5 min, followed again by a 10-s wash and 3 × 5-min washes with PBS. Samples were either imaged immediately following sample preparation or sealed with parafilm and stored overnight at 4 °C. For samples stored overnight, the PBS was replaced and samples were equilibrated to ambient temperature before imaging.

### Labeling of cells with Hoechst derivative 6_HST_

Hoechst derivative **6**_**HST**_ was dissolved in anhydrous DMSO (Thermo Fisher Scientific, 50-148-9333) to achieve a final concentration of 1 mM. The ligand stock solution was aliquoted and stored at −20 °C; a freshly thawed aliquot was used immediately for each experiment. The **6**_**HST**_ stock solution was diluted in PBS to a final concentration of 10 nM. Cells were prepared as described above and then incubated with this PBS solution throughout the imaging procedure.

### SMLM imaging of HaloTag-expressing cells labeled with 1_HTL_–9_HTL_ and 6_HST_

All samples were imaged using a custom-built highly inclined swept tile microscope^31^, based on a Nikon TI2 platform and utilizing a 60 × 1.27 numerical aperture objective (MRY10060, Nikon). Individual WGA-ND images were acquired using 11.5 mW 560 nm laser illumination with a Semrock FF01-680/42-32 emission filter. All single-molecule images were acquired using 560 nm (2RU-VFL-P-2000-560-B1R, MPB Communications) and 647 nm (2RU-VFL-P-2000-642-B1R, MPB Communications) laser illumination to optimally excite both the ND and dye molecules and imaged onto a camera (Fusion BT, Hamamatsu) through a bandpass emission filter (FF01-680/42, Semrock). Regions of interest (ROIs) were identified under low power illumination conditions (11.5 mW at 560 nm and 1.6 mW at 647 nm, measured at the objective back pupil). Each ROI contained at least three WGA-ND fiducial markers for saturating and blinking imaging conditions, and at least one WGA-ND fiducial for blink lifetime imaging conditions. To measure dye on-time, 20,000 frames were acquired with 2-ms exposure (3-ms cycle time) using 40 mW of 647 nm and 11.5 mW of 560 nm illumination. To measure dye off-time and for representative super-resolution renderings 100,000 frames were acquired with 25-ms exposure (27-ms cycle time) under the same illumination conditions as above. For live-cell imaging, cells were maintained at 37 °C and 5% (v/v) CO_2_ using a live-cell imaging chamber (Okolab, H301-PI-736). A total 20,000 frames were acquired with 2-ms exposure (3-ms cycle time) using 40 mW of 647 nm illumination light.

### SMLM image processing of HaloTag-expressing cells labeled with 1_HTL_–9_HTL_ and 6_HST_

Individual blink events within the images were fit using the SMAP localization software package^32^ based on MATLAB (The MathWorks) with the following parameters: Peak Finder (DoG, s: 1.2, dynamic: 1.7), Fitter (point spread function (PSF) Fix; PSFx start, 2 pix; ROI size, 7 pix; iterations, 25). The data were additionally filtered to keep only localizations that could be localized to <40 nm, emitted more than 100 photons per frame (for blink quantification) or 40 photons per frame (for blink lifetime measurement). Sample drift was compensated for by tracking multiple fiducial ND within the field of view and subtracting their motion over time from the localization coordinates. To quantify the photophysical properties of each dye, localizations were first assigned to individual cells using a manually outlined binary mask. To determine dye on-time, multiple localizations were considered to have come from the same dye molecule if they were observed in consecutive frames within 100 nm of a previous observation. To distinguish long-lived blinking events that occur from dye protonation/deprotonation from transient events that may be due to the Poisson nature of photon emission or noise, a ‘gap-closing’ parameter of five frames (~17 ms) was used such that if an ‘on’ molecule transiently drops below our detection threshold and reappears within five frames, it is still considered the same ‘on’ event. To validate the choice of a five-frame gap, the data from JF_630_b–HaloTag ligand (**6**_**HTL**_) were analyzed using gap-closing parameters of one, two, three and four frames. The calculated on-times (mean ± s.d.) were: 8.5 ± 0.7 ms (one frame), 9.3 ± 0.7 ms (two frames), 9.7 ± 0.6 ms (three frames) and 10.0 ± 0.6 ms (four frames). As expected, the on-times show a general trend to higher numbers as the frame gap increases, but the values are consistent with the reported five-frame value of 10.1 ± 0.6 ms (Kruskal–Wallis test, *H* = 7.067, *P* = 0.1324; Extended Data Fig. 4b,c,n). To determine dye off-time, sparse labeling was used such that groups of localizations could be unambiguously assigned to a single dye molecule even if the localizations occurred at distant time points. All localizations acquired within 100,000 frames (45 min of imaging) were plotted and groups were identified using the ‘dbscan’ function with a grouping radius of 100 nm to allow for sample drift and thermal fluctuations. Off-time events for each molecule were quantified as the intervening time experienced between localizations for each grouping. For live-cell analysis, the relationship between resolution and the number of frames used for super-resolution rendering was assessed by generating images from localizations collected over increasing time intervals (0.5–30 s). Resolution was quantified using a custom Python implementation of Fourier ring correlation^33^. To determine resolution over time, images were rendered from a fixed number of frames corresponding to 5 s (1,470 frames), and Fourier ring correlation was performed between pairs of sequential images.

### 3D SMLM Interferometric photoactivated localization microscopy

U2OS cells were transiently transfected with a LifeAct–HaloTag construct from Janelia Research Campus using a Bio-Rad Gene Pulser XCell (exponential decay protocol, 200 V, 950 μF, resistance ∞ and 4-mm cuvette). Cells were plated on 25-mm round coverslips containing gold nanorods (CC12-40-600-NEG-DIH-50-1, Nanopartz) to serve as fiducial markers for calibration and corrections. Cells were labeled with 0.5 μM JF_635_b–HaloTag ligand (**5**_**HTL**_) in McCoys’ 5 A medium for 1 h, rinsed twice in 1× PBS, fixed in 4% (w/v) formaldehyde for 10 min followed by multiple washes in PBS.

iPALM imaging followed the established protocols^34^. The fixed cells were covered with 100 μl PBS and covered with an 18-mm round coverslip (no. 1.5, CS-18R17, Warner Instruments) that was sealed with Valap (1:1:1 (v/v/v) Vasoline/lanolin/paraffin wax). The JF_635_b dye was excited with a 647 nm laser (Opto Engine) with a laser power of 2–3 kW cm^−2^ and fluorescence was collected with two opposed Nikon ×60/1.49 NA Apo TIRF objective lens using a 647 nm long-pass filter (LP02-647RU, Semrock) and three EMCCD cameras (iXon3-DU897E, Andor Technologies) with an exposure time of 30 ms. Single-molecule images of 500,000 frames were collected, localized, processed and rendered with the PeakSelector Software (Janelia Research Campus)^34,35^. The gold rods on the coverslip were used to align the three cameras with each other as well as to correct for drift in *x*, *y* and *z*. In addition, seven gold rods across the field of view were used to estimate the localization precision by measuring their full width half maximum in all three dimensions (Fig. 1j–m) resulting in a mean ± s.d. of 14.92 ± 3.15 nm in *x*, 22.92 ± 5.41 nm in *y* and 12.77 ± 6.00 nm in *z* (Extended Data Fig. 5k).

### Labeling of oligonucleotides for 3D super-resolution ATAC experiments

The 3D super-resolution ATAC experiments including purification of the Tn5 transposase followed a previous protocol^23^. The mosaic end (ME) adaptors for Tn5 transposase were synthesized by Integrated DNA Technologies with attachment of a 5′ end primary amino group by a six-carbon spacer arm (C6). The oligonucleotide sequences and their modifications are:

Tn5ME-A: 5′-amino-C6 TCGTCGGCAGCGTCAGATGTGTATAAGAGACAG3′

Tn5ME-B: 5′-amino-C6-GTCTCGTGGGCTCGGAGATGTGTATAAGAGACAG-3′

Tn5MErev: 5′-(phos)CTGTCTCTTATACACATCT-3′

The *N*-hydroxysuccinimide ester of PA-JF_646_ (PA-JF_646_-NHS; **11**_**NHS**_)^11^ or JF_635_b-NHS (**5**_**NHS**_) was dissolved in anhydrous DMSO immediately before conjugation. The Tn5ME-A and Tn5ME-B oligonucleotides were first dissolved in deionized water and then extracted 3× with CHCl_3_. The oligonucleotides in the aqueous fraction were precipitated by addition of 3 M sodium acetate (pH 5.2) and 70% (v/v) ethanol and the solid isolated by centrifugation (13,000*g*). The pellet was washed with 70% (v/v) ethanol, dried and dissolved in ultrapure H_2_O. The purified oligonucleotides were reacted with excess **11**_**NHS**_ or **5**_**NHS**_ (DMSO stock solutions; mass ratio 1:2.5 oligonucleotide:dye) in 0.1 M sodium tetraborate, pH 8.5. The reaction was incubated at ambient temperature overnight (>12 h) with constant stirring and protection from light. The crude labeled oligonucleotides were precipitated by addition of 3 M sodium acetate (pH 5.2) and ethanol. The solid was collected by centrifugation (13,000*g*) and the pellet was dissolved in 0.1 M TEAA (triethylammonium acetate; Thermo Fisher Scientific, 400613). Purification of oligonucleotide-dye conjugates was performed on an Agilent 1200 analytical high-performance liquid chromatography (HPLC) system equipped with an autosampler, diode array detector and fraction collector, using an Eclipse XDB-C18 column (4.6 × 150 mm, 5 µm; Agilent), and eluting with a linear gradient of 0–100% (v/v) CH_3_CN/H_2_O with constant 10 mM TEAA additive; 30 min run; 1 ml min^−1^, detection at 260 nm. Sample fractions were pooled and lyophilized to obtain the product as colorless solid.

### 3D super-resolution ATAC

The Tn5 transposome with dye-labeled oligonucleotide duplexes was assembled according to established protocols^23^. The HPLC-purified Tn5ME-A-dye or Tn5ME-B-dye oligonucleotides were dissolved in H_2_O to yield stock solutions of 100 μM. These oligonucleotides were then annealed to the Tn5MErev oligonucleotide by adding an equimolar amount of Tn5ME-A-dye or Tn5ME-B-dye to a solution of Tn5MErev in 10 mM Tris-HCl, 50 mM NaCl and 1 mM EDTA, pH 8.0. These solutions were denatured on a benchtop thermocycler at 95 °C for 5 min and then slowly cooled down to 25 °C at the rate of −1 °C min^−1^. We prepared wild-type mouse embryonic stem (mES) cells for 3D ATAC–SMLM experiments according to established protocols^23^. Cells were plated onto 5-mm coverslips (Warner Instruments, cat. no. 64-0700) at ~80% confluency with appropriate coating 24 h before the experiment. Cells were fixed with 4% (w/v) paraformaldehyde (Electron Microscopy Sciences, cat. no. 15710) for 10 min at ambient temperature. After fixation, cells were washed for 3 × 5 min with PBS and then permeabilized with ATAC lysis buffer (10 mM Tris-HCl, pH 7.4, 10 mM NaCl, 3 mM MgCl_2_ and 0.1% (w/v) Igepal CA-630) for 10 min at ambient temperature. After permeabilization, the slides were washed 2× with PBS and placed inside a humidity chamber at 37 °C. The transposase mixture solution (1× Tagmentation buffer: 10 mM Tris-HCl, pH 7.6, 5 mM MgCl_2_, 10% (v/v) dimethylformamide and 100 nM Tn5-dye conjugated oligonucleotide complex) was added to the cells and incubated for 30 min at 37 °C inside the humidity chamber. After the transposase reaction, slides were washed 3 × 15 min with 1× PBS containing 0.01% (w/v/) SDS and 50 mM EDTA at 55 °C before mounting onto the lattice light-sheet microscope for super-resolution ATAC imaging.

The 3D super-resolution ATAC data were acquired by the lattice light-sheet microscopy at ambient temperature^36^. The light sheet was generated from the interference of highly parallel beams in a square lattice and dithered to create a uniform excitation sheet. The inner and outer numerical apertures of the excitation sheet were set to be 0.44 and 0.55, respectively. A variable-flow peristaltic pump (Fisher Scientific, cat. no. 13-876-1) was used to connect a 2 L reservoir with the imaging chamber with PBS circulating through at a constant flow rate. Labeled cells seeded on coverslips were placed into the imaging chamber and each imaging volume took 100–200 image frames, depending on the depth of the field of view. The specimen was illuminated using a custom 0.65 NA excitation objective (Special Optics) and emitted light was collected by a detection objective (CFI Apo LWD ×25 W, 1.1 NA, Nikon), filtered through a 440/521/607/700 nm BrightLine quad-band bandpass filter (Semrock) and N-BK7 Mounted Plano-Convex Round cylindrical lens (*f* = 1,000 mm, Ø 1’, Thorlabs, cat. no. LJ1516RM) and recorded by an ORCA-Flash 4.0 sCMOS camera (Hamamatsu, cat. no. C13440-20CU). The cells were imaged under sample scanning mode and the dithered light sheet at 500-nm step size, thereby capturing a volume of ~25 × 51 µm × (~27–54) µm, considering the 32.8° angle between the excitation direction and the stage moving plane. The stained samples were initially photobleached by scanning the whole imaging volume with a 2 W 640 nm laser (MPB Communications) to achieve sparse labeling. For the photoactivatable PA-JF_646_ label, the samples were imaged by iteratively photoactivating each plane with low-intensity 405 nm light (<0.05 mW power at the rear aperture of the excitation objective; 6 W cm^−2^ power at the sample) for 8 ms and by alternatively exciting each plane with a 2 W 640 nm laser at its full power (26 mW power at the rear aperture of the excitation objective and 3466 W cm^−2^ power at the sample) for a 20-ms exposure time. For the spontaneously blinking JF_635_b label, the samples were imaged using the same settings: 640 nm laser at full power with 20-ms light exposure for each plane; the activation light was not used with this label.

To analyze the 3D super-resolution ATAC data, nano-gold fiducials were embedded within the coverslips for drift correction according to established protocols^23^. Super-resolution ATAC images were taken to construct a 3D volume when the sample was moving along the ‘*s*’ axis. Individual volumes per acquisition were automatically stored as TIFF stacks, which were then analyzed by in-house developed scripts in MATLAB. The cylindrical lens introduced astigmatism in the detection path and recorded each isolated single molecule with its ellipticity, thereby encoding the 3D position of each molecule relative to the microscope focal plane. All processing was performed by converting all dimensions to units of *x*–*y* pixels, which were 100 × 100 nm after transformation due to the magnification of the detection objective and tube lens. Localization precision was estimated by calculating the standard deviation of all the localizations coordinates (*x*, *y* and *z*) after the nano-gold fiducial correction. The localization precision was 26 ± 3 nm and 53 ± 5 nm for *x*–*y* and *z*, respectively.

### Expression and purification of FUS variants

pMBP-*tev*-FUS-*tev*-His_6_ (Addgene #242384) and pMBP-*tev*-FUS^A2C^-*tev*-His_6_ (Addgene, #242385) were generated by PCR amplifying the FUS coding sequence in pMBP-*tev*-FUS–EGFP-*tev*-His_6_ (*aka* pMal-*Tev*-FUS(WT)–EGFP-*Tev*-His_6_ as described by Hofweber et al.^37^) with SalI/HindIII ends without or with the A2C mutation and ligating the digested fragment into the original plasmid digested with SalI/HindIII, which removes the FUS–EGFP coding sequence. Inserted sequences were confirmed by DNA sequencing. Plasmid constructions are detailed in the history of the SnapGene files available on Addgene. Plasmids pMBP-*tev*-FUS-*tev*-His_6_ and pMBP-*tev*-FUS^A2C^-*tev*-His_6_ were used to overproduce MBP–FUS and MBP–FUS^A2C^, respectively, which were purified identically as MBP–FUS–EGFP^37^.

The FUS protein was overproduced in *Escherichia* *coli* strain Rosetta 2(DE3)pLysS (Novagen) transformed with the appropriate plasmid. Cells were grown in lysogeny broth with 50 µg ml^−1^ ampicillin and 34 μg ml^−1^ chloramphenicol. After incubating a 5-ml starter culture overnight at 37 °C, it was centrifuged (4,000*g*, 5 min, 25 °C) and resuspended in 1 l fresh medium, which was incubated at 37 °C. At an OD_600_ of ~0.8, protein overproduction was induced with 1 mM isopropyl β-D-1-thiogalactopyranoside (IPTG). Cultures were incubated for 24 h at 20 °C with shaking at 250 rpm before pelleting (4,000*g*, 20 min, 4 °C). Cells were recovered (4,000*g*, 20 min, 4 °C) and cell pellets were resuspended in 25 ml resuspension buffer consisting of 50 mM Na_2_HPO_4_/NaH_2_PO_4_, 500 mM NaCl, 10 μM ZnCl_2_, 10 mM imidazole, 4 mM β-mercaptoethanol (βME), 10% glycerol, pH 7.5, and protease inhibitors 10 mM phenylmethyl sulfonyl fluoride (PMSF), 100 µg ml^−1^ trypsin inhibitor, 20 µg ml^−1^ leupeptin and 100 µg ml^−1^ pepstatin. Cells were lysed using a French press (3× at 16,000 psi), the lysate was centrifuged (15,000*g*, 20 min, 4 °C), and the supernatant was incubated with 500 µl of Ni-NTA resin (QIAGEN) for 30 min at 25 °C. The suspension was transferred to a gravity column. The resin was washed with ten column volumes (CVs) of resuspension buffer with 2 mM PMSF, followed by five CVs of wash buffer A consisting of 50 mM Na_2_HPO_4_/NaH_2_PO_4_, 500 mM NaCl, 10 μM ZnCl_2_, 10 mM imidazole, 4 mM βME and 10% glycerol, pH 7.5. Fractions (1 ml) were eluted with elution buffer A consisting of 50 mM Na_2_HPO_4_/NaH_2_PO_4_, 500 mM NaCl, 10 μM ZnCl_2_, 400 mM imidazole, 4 mM βME and 10% (v/v) glycerol, pH 7.5. Protein-containing fractions (<15 ml) were combined and diluted with 100 ml dilution buffer consisting of 50 mM Na_2_HPO_4_/NaH_2_PO_4_, 150 mM NaCl, 10 μM ZnCl_2_, 10 mM imidazole, 10% (v/v) glycerol and 4 mM βME, pH 7.5. After gentle shaking at ambient temperature for 5 min, the protein mixture was loaded onto a gravity flow column with 2 ml amylose resin (New England Biolabs, E8021). The resin was washed with five CVs of wash buffer B consisting of 50 mM Na_2_HPO_4_/NaH_2_PO_4_, 200 mM NaCl, 10 μM ZnCl_2_, 10 mM imidazole and 10% (v/v) glycerol, pH 7.5, and the protein was eluted with amylose elution buffer consisting of 20 mM Tris, 150 mM NaCl, 20 mM maltose, 1 mM EDTA, 5% (v/v) glycerol, pH 7.5. For MBP–FUS^A2C^, 2 mM Tris(2-carboxyethyl)phosphine (TCEP) was added before storage. The eluted protein was typically ~20–30 µM, and it was flash frozen in liquid nitrogen and stored at −80 °C in 10 µl aliquots until use. Protein concentrations were determined by the densitometry of bands on SDS–PAGE gels stained with Coomassie blue R-250 using carbonic anhydrase as a standard and a ChemiDoc MP imaging system (Bio-Rad Laboratories). The purity of dye-labeled proteins was assayed by direct in-gel fluorescence imaging using the same ChemiDoc imaging system and was determined to be >95%.

### Labeling of FUS variants

MBP–FUS^A2C^ was reacted with a 15-fold molar excess of the spontaneously blinking dye HM-SiR-maleimide^10^ (**1**_**MAL**_; SaraFluor 650B-maleimide; Goryo Chemical, A209-01) or JF_635_b-maleimide (**5**_**MAL**_) at ambient temperature for 15 min. The reaction was quenched with 10 mM βME. The dye-protein mixture was incubated with 0.2 ml Ni-NTA resin for 15 min and loaded into a gravity flow column. Excess dye was removed by washing the resin-bound protein with 100 CVs of wash buffer C consisting of 50 mM Na_2_HPO_4_/NaH_2_PO_4_, 10 mM imidazole, 10 µM ZnCl_2_, 0.5 M NaCl, 0.2% (v/v) Triton X-100, 1 mM dithiothreitol (DTT) and 10% (v/v) glycerol, pH 7.5, and 50 CVs of wash buffer D consisting of 50 mM Na_2_HPO_4_/NaH_2_PO_4_, 10 mM imidazole, 10 µM ZnCl_2_, 150 mM NaCl, 2 M urea, 1 mM DTT and 10% glycerol, pH 7.5. To assess free dye removal, Ni-NTA resin (10 µl) was incubated with reducing SDS sample buffer and proteins were analyzed by electrophoresis. The acrylamide gel was incubated in ~5% (v/v) acetic acid (pH 2–3) for 5 min to convert the blinking dye molecules into the protonated fluorescent state, and the sample was analyzed by direct in-gel fluorescence imaging (Epi Red, *λ*_ex_ = 625–650 nm). If the free dye was still present, the resin was washed again and re-assayed. Once free dye was no longer detected in the gel image (<2%), the protein was eluted with elution buffer B consisting of 50 mM Na_2_HPO_4_/NaH_2_PO_4_, 250 mM imidazole, 10 µM ZnCl_2_, 150 mM NaCl and 10% (v/v) glycerol, pH 7.5. The eluted protein was typically 1–2 µM, and it was flash frozen in liquid nitrogen and stored at −80 °C in 3-µl aliquots until use.

### FUS condensate formation and sample preparation

FUS protein stock solutions were thawed at ambient temperature and centrifuged (14,000*g*, 10 min, 25 °C). Supernatants were used immediately. MBP–FUS solutions (diluted to 14 µM) were mixed with MBP–FUS^A2C^-JF_635_b (40 nM) in droplet buffer A consisting of 20 mM Na_2_HPO_4_/NaH_2_PO_4_, 2.5% (v/v) glycerol, 1 mM DTT and 150 mM NaCl, pH 7.5. This protein mixture was then diluted twofold with no-salt droplet buffer B consisting of 20 mM Na_2_HPO_4_/NaH_2_PO_4_, 2.5% (v/v) glycerol and 1 mM DTT, pH 7.5 to yield a final salt concentration of 75 mM and 7 µM MBP–FUS. Phase separation was induced by the addition of 0.3 µl (3 U) of acTEV protease (10 U µl^−1^; Invitrogen, 12575015) to 10 µl of the FUS protein mixture, which resulted in proteolytical removal of the MBP solubility tag and the 6×His-tag on the FUS constructs^37^. After incubation at ambient temperature for 10 min, the solution was added to a microscope slide flow chamber (~10 µl), and sealed with clear nail polish to prevent evaporation. Flow chambers were made from large coverslips (Gold Seal 3243; 24 × 60 mm; Thermo Fisher Scientific, 50-189-9138) that were argon plasma cleaned and pretreated with 1.5% mPEG-silane (LaysanBio, MPEG-SIL-5000) for 30 min at ambient temperature to passivate the surface, followed by 3×10 µL washes with droplet buffer. Small coverslips (10.5 × 22 mm; Electron Microscopy Sciences, 72191-22) with double-sided tape on their short edges were adhered on top of a large coverslip to construct flow chambers.

### 3D super-resolution microscopy of FUS condensates

The 3D imaging of BMCs followed established protocols^13,38^. A Zeiss Axiovert 200 M equipped with an oil-immersion objective (Zeiss Alpha Plan-Apochromat, 1.40 NA) and sCMOS camera (Teledyne Photometrics, Prime 95B, square pixel dimension of 120 nm at the camera plane) was used to capture the fluorescence emission from individual HM-SiR or JF_635_b molecules during their rare on-time events within FUS condensates. Laser excitation was converted to circularly polarized light with half (*λ*/2) and a quarter (*λ*/4) waveplates (Newport, 10RP42-1 and 10RP44-1) before entering the microscope as a slightly converging illumination beam. A self-configured adaptive optics system (Imagine Optic, AOKit Bio) in the emission path generated the *z*-dependent spot ellipticity needed for 3D information using 60 nm root mean square (rms) astigmatism. The red emission channel was collected with a double-bandpass filter set (Chroma, ZT532/640/NIR-RPC-UF2). A self-configured TIRF-Lock system (Mad City Labs) provided a *z*-stability for the coverslip of <3 nm for the duration of the experiment.

### Density maps from 3D localizations in FUS condensates

To generate 3D density maps, condensates were formed with 2 nM FUS^A2C^-JF_635_b. Localizations (one localization/blink) were acquired with 647 nm diode laser illumination (Coherent, OBIS 647, 120 mW) with ~10 kW cm^−1^ at the sample and 30 ms per frame for 20 min. Image sequences were analyzed using the ThunderSTORM plugin^39^ for Fiji^40^. The maximum likelihood method was used to determine all single-molecule spot centroids (*x*, *y*) and the ‘elliptical Gaussian’ fitting option was used to fit the astigmatic point spread functions (PSFs), which yielded the *z*-positions. The calibration file required for the *z*-dependent PSF was obtained from *z*-stacks (−500 nm to +500 nm; 25-nm steps) of 0.1-µm fluorescent microspheres (Thermo Fisher Scientific, T7279) and were fit as described by Huang et al.^41^ and implemented in the ThunderSTORM plugin. The post-processing option ‘remove duplicates’ was used to eliminate overlapping molecules, ‘merging’ was used to eliminate repeat localizations from the same blink, and ‘average shifted histograms’ (magnification of 3; lateral shift of 1) was used for visualization.

### Endoplasmic reticulum imaging via super-resolution optical fluctuation imaging using HaloTag ligands 1_HTL_–9_HTL_ in live and fixed cells

U2OS cells stably expressing Sec61b–HaloTag fusion protein were plated on plated in 35-mm glass-bottom MatTek dishes and allowed to grow to appropriate density (5 × 10^4^ cells per cm^2^) in phenol-free DMEM containing 10% (v/v) FBS for imaging. Cells were labeled with 100 nM of one of the nine HaloTag ligand-containing blinking dyes (**1**_**HTL**_**–9**_**HTL**_) for 30 min. For fixed-cell experiments, cells were washed 3× with medium and then fixed with a solution of 4% (w/v) paraformaldehyde in PBS buffer, pH 7.5. The fixed cells were imaged in PBS at ambient temperature. For live-cell imaging experiments, cells were maintained at 37 °C and 5% (v/v) CO_2_ using a Tokai-hit stage top incubator and objective heater. All cells were imaged using an Olympus ×100 1.5 NA TIRF objective on a RAMM frame microscope (ASI) equipped with a tube lens (LAO-300.0, Melles Griot), resulting in ×166.66 overall magnification^42^. An ASI CRISP autofocus system based on the reflection of the 940 nm diode provided continuous and simultaneous positional stabilization along the optical axis. Emitted light was collected on a Andor iXon Ultra EMCCD camera with increased sensitivity in the NIR (model DU-897UCS0-EXF) that was operated using Micro-Manager^43^ v.1.4.20 with the following settings: gain 400, cooled to −60 °C, 17 MHz EM amplifiers, preamp setting of 3. The sample was illuminated using a Stradus 637-140 laser (Vortran) illuminated the sample in a highly inclined and laminated optical sheet (HILO) configuration with an illumination power density of 400 W cm^−2^. The 10,000 frames were recorded at 100 frames per second and were analyzed with Igor Pro v.9.05 (WaveMetrics) using the Localizer plugin^44^ to reconstruct second-order SOFI images.

### Lysosomal imaging *via* SOFI using HaloTag ligands 4_HTL_ and 9_HTL_ in live and fixed cells

U2OS cells were transfected (Amaxa) with 500 ng plasmid per 1 × 10^6^ cells encoding the lysosomal marker HaloTag–CD63 (pPB_UCOE_EF1a_Halo_CD63_IRES_HygR). The cells were plated on plated in 35-mm glass-bottom MatTek dishes and allowed to grow to appropriate density (5 × 10^4^ cells per cm^2^) in phenol-free DMEM containing 10% (v/v) FBS for imaging. Cells were labeled with 20 nM **4**_**HTL**_ or 100 nM **9**_**HTL**_ for 1.5 h. For fixed-cell experiments, cells were washed 3× with medium and then fixed with a solution of 4% (w/v) paraformaldehyde in PBS buffer, pH 7.5. The fixed cells were imaged in PBS buffer at ambient temperature. For live-cell imaging experiments, cells were maintained at 37 °C and 5% (v/v) CO_2_ using a Tokai-hit stage top incubator and objective heater. All lysosomal cells were imaged using the same settings on the RAMM frame microscope (ASI) as for endoplasmic reticulum imaging. Live and fixed-cell SOFI images were processed from 100 frames collected at 100 Hz processed as SOFI images at 1 Hz. Individual lysosomes were tracked from a dataset of 10,000 images using the software package Localizer^44^ with a minimum track length of five frames, and a maximal jump distance of 15 pixels.

### Labeling of oligonucleotides for RNA-FISH

The oligonucleotides used to prepare RNA-FISH probes were synthesized by Integrated DNA Technologies with a 5′ primary amino group and a six-carbon spacer arm (C6). The oligonucleotide sequences and their modifications are:

5′-amino-C6-TTTCTAGAGTCGACCTGCAG-3′

5′-amino-C6-CTAGGCAATTAGGTACCTTAG-3′

5′-amino-C6-CTAATGAACCCGGGAATACTG-3′

Oligonucleotides (500 nmol) were dissolved in 500 µl deionized water and extracted three times with chloroform. The aqueous solution was separated, and the oligonucleotide was precipitated by adding 50 µl of 3 M NaCl (aq) and 1,250 µl ethanol and incubating at −20 °C for 30 min. The sample was centrifuged (13,000*g*, 30 min) and the resulting precipitate was washed with 70% (v/v) ethanol and dried. Purified oligonucleotides (17 nmol) were dissolved in 0.1 M sodium tetraborate, pH 8.5 (36.5 µl) and **11**_**NHS**_, **5**_**NHS**_ or **6**_**NHS**_ (25 nmol) in DMSO (3.5 µl) were added. The reaction was incubated for 48 h at ambient temperature with constant shaking and protection from light. The oligonucleotide was precipitated by adding 10 µl of 3 M NaCl (aq) and 250 µl ethanol and incubating at −20 °C for 30 min. Samples were centrifuged (13,000*g*, 30 min) and the resulting precipitate was washed twice with 70% (v/v) ethanol and then dissolved in deionized water. Purification of labeled oligonucleotides was performed on an Agilent 1200 HPLC purification system equipped with an autosampler, diode array detector and fraction collector. Samples were introduced onto a 4.6 × 150 mm, 5 μm, Eclipse XDB-C18 column (Agilent) using the system autosampler and eluted with a linear gradient of 0 → 100% (v/v) CH_3_CN/H_2_O (10 min, 3 ml min^−1^) containing a constant 10 mM TEAA additive. The purification was monitored using the system diode array detector (*λ*_abs_ = 260 nm) and collected using the system fraction collector. Product-containing fractions were pooled and lyophilized to obtain the labeled oligonucleotide products as white solids.

### Cell culture and sample preparation for RNA-FISH experiments

The protocol for RNA-FISH using immortalized mouse embryonic fibroblasts (MEFs) was described previously^45^. MEFs derived from a transgenic mouse containing a MS2 binding site (MBS) cassette targeted to the 3′ UTR of the essential β-actin gene were cultured in growth medium comprising DMEM (Thermo Fisher Scientific, 11965118), supplemented with 10% (v/v) FBS (Avantor 1300-500H) and 100 U ml^−1^ penicillin–streptomycin (Thermo Fisher Scientific, 15140122). Cells were grown on MatTek dishes to 50% confluency, washed with PBSM consisting of PBS supplemented with 5 mM MgCl_2_, and then fixed in 4% (w/v) paraformaldehyde for 10 min at ambient temperature. The cells were then washed with PBSM (3 × 5 min) and then with PBSM supplemented with 50 mM glycine (1 × 5 min). Before hybridization, cells were permeabilized by incubation with 0.5% (w/v) Triton X-100 in PBS for 10 min, washed with PBSM (3 × 5 min), and then incubated for 10 min in prehybridization solution consisting of 10% (v/v) formamide, 10% (w/v) dextran sulfate, 2× saline-sodium citrate (SSC) buffer, 0.2 mg ml^−1^ BSA, 1 mg ml^−1^ *E.* *coli* tRNA, 2 mM ribonucleoside vanadyl complexes (New England Biolabs) and 10 U ml^−1^ Superase (Invitrogen). Cells were then incubated with 200 µl prehybridization solution supplemented with 20 ng DNA probe at 37 °C for 18 h. Cells were washed with a solution of 10% v/v formamide in 2× SSC buffer at 37 °C (2 × 15 min) and then washed with 2× SSC buffer at ambient temperature (3 × 10 min).

### Fluorescence microscopy for RNA-FISH experiments

All samples were imaged at ambient temperature in PBS buffer. Wide-field, SOFI and SMLM experiments were performed using an ELYRA system (Carl Zeiss) with a ×63/1.4 NA Plan-Apo objective augmented with a Zeiss ×1.6 magnification lens resulting in a pixel size of 158.7 nm. The system contained an EMCCD camera (Andor Ultra 897, Gain 300) capable of single-photon detection, and the samples were illuminated with a 642 nm excitation laser in a HILO configuration at a frame rate of 33.33 Hz with a constant illumination power density of 100 W cm^−2^ for wide-field and SOFI experiments and 300 W cm^−2^ for SMLM experiments. Second-order SOFI images were reconstructed from 2,000-frame movies using Igor Pro 7 software (WaveMetrics) with the Localizer plugin^44^. SMLM experiments were performed as previously described^6,11^. For correction of sample drift, 100-nm TetraSpeck Microspheres (Invitrogen) were affixed to the cover glass by incubation in the medium for 30 min at ambient temperature. SMLM images were generated using the ThunderSTORM plugin^39^ for Fiji^40^ from a 5,000–20,000-frame video with maximum likelihood integrated Gaussian fitting option; video length was chosen to generate datasets with approximately equal localizations. SMLM images were visualized using normalized Gaussians.

### SMLM and SOFI of mitochondria using JFX_650_b–HaloTag ligand (2_HTL_) in live cells

U2OS cells were transfected with HaloTag–TOMM20 (Addgene, #123284) with an Amaxa Nucleofector II and plated on plasma-cleaned, silanized coverslips that had been coated overnight at 4 °C with 5 mg ml^−1^ human plasma fibronectin (EMD Millipore, FC010). After 18–24 h, cells were labeled with 200 nM JF_650_b-HaloTag ligand (**2**_**HTL**_) for 30 min, using a freshly thawed aliquot of DMSO stock solution for each experiment. Cells were rinsed in 37 °C complete McCoy’s medium and allowed to recover for at least 1 h before imaging. Imaging was performed using a ×60 1.49 NA objective on an Olympus IX71 custom TIRF system equipped with a 37 °C air curtain. The 16,000 frames were acquired with 8-ms exposure using an Andor iXon 897 EMCCD at a 111-nm pixel resolution and a 637 nm laser for excitation, with a Semrock Penta set, Di01-FF409/49/57/652/759, 432/514/595/681/809. The TIRF angle was controlled by a programmable Thorlabs motor (Z812B) and custom LabView software. The 16,000 frames were parsed into 250-frame image stacks for further processing. Second-order linear SOFI images were created from these 250-frame stacks using MATLAB software (https://github.com/kgrussmayer/sofipackage)^5^. TIRF images were created by summing the individual stacks in Fiji^40^, and SMLM images were generated using the ThunderSTORM plugin^39^ in Fiji, with 20 nm localization and 20 nm Gaussian rendering. The 500-frame stacks were also processed to ensure that features were not undersampled.

### SMLM and SOFI of paxillin using JF_639_b–HaloTag ligand (4_HTL_) in live cells

CHOK1 cells were stably transfected with a sequence-verified HaloTag–paxillin construct created by ligating the sequence encoding the HaloTag protein from Promega N-Halo vector into mEmerald Paxillin-N22 (Addgene, #54219) using XhoI and NotI. Cells were labeled with 200 nM JF_639_b–HaloTag ligand (**4**_**HTL**_) for 30 min, using a freshly thawed aliquot for each experiment. Cells were then trypsinized and replated onto plasma-cleaned, silanized coverslips that had been coated overnight at 4 °C with 5 μg ml^−1^ human plasma fibronectin (EMD Millipore, FC010). Cells were allowed to spread 2–4 h previous in growth medium (DMEM-F12 containing 15 mM HEPES; Gibco 11039-021) supplemented with 10% (v/v) fetal calf serum (Hyclone SH30396.03). Imaging was performed using a ×60 1.49 NA objective on an Olympus IX71 homemade TIRF system equipped with a 37 °C air curtain. The 16,000 frames were acquired with 15-ms exposure using an Andor iXon 897 EMCCD at a 111-nm pixel resolution and a 637 nm laser for excitation. The 16,000 frames were parsed into 500-frame image stacks for further processing. Second-order linear SOFI images were created from these 500-frame stacks using MATLAB software (https://github.com/kgrussmayer/sofipackage)^5^. TIRF images were created by summing the individual stacks in Fiji^40^, and SMLM images were generated using the ThunderSTORM plugin^39^ in Fiji, with Gaussian 30 nm localization. Analysis was also performed using 1,000-frame image stacks to ensure that features were not undersampled.

### Reporting summary

Further information on research design is available in the Nature Portfolio Reporting Summary linked to this article.

## Online content

Any methods, additional references, Nature Portfolio reporting summaries, source data, extended data, supplementary information, acknowledgements, peer review information; details of author contributions and competing interests; and statements of data and code availability are available at 10.1038/s41592-026-03062-5.

## Supplementary information

Supplementary Information Supplementary Notes 1 and 2.

Reporting Summary

Supplementary Video 1 **Performance of 1**_**HTL**_**–9**_**HTL**_ **in SMLM**. Initial clips from SMLM imaging sessions of paraformaldehyde-fixed COS-7 cells expressing HaloTag–histone H2B fusion proteins and labeled to saturation with HaloTag ligands **1**_**HTL**_–**9**_**HTL**_ (25-ms exposure; 37 frames per second). The intensity of the JFX_650_b–HaloTag ligand (**2**_**HTL**_) movie was adjusted with the Bleaching Correction ImageJ plugin (Simple Ratio, v.2.1.0)^29^.

Supplementary Video 2 **Live-cell SMLM of the endoplasmic reticulum using JF**_**630**_**b**. Live-cell SMLM imaging of U2OS cells expressing Sec61β–HaloTag and labeled with JF_630_b–HaloTag ligand (**6**_**HTL**_). SMLM images were rendered with an effective frame rate of 0.2 Hz.

Supplementary Video 3 **Comparison of HM-SiR and JF**_**635**_**b for imaging bimolecular condensates**. Initial clips from an SMLM imaging session of FUS BMCs doped with 0.03% mole fraction of FUS^A2C^ labeled with HM-SiR-maleimide (**1**_**MAL**_; **a**) or JF_635_b-maleimide (**5**_**MAL**_; **b**).

Supplementary Video 4 **Live-cell super-resolution imaging of paxillin using SOFI and SMLM with JF**_**639**_**b**. CHOK1 cells expressing HaloTag–paxillin labeled with JF_639_b–HaloTag ligand (**4**_**HTL**_) were imaged for 4 min continuously with 15-ms exposure times. Each video frame was created by processing 500 single-molecule images.

## Source data

Source Data Fig. 1 Statistical Source Data.

Source Data Fig. 2 Statistical Source Data.

Source Data Extended Data Fig. 2 Statistical Source Data.

Source Data Extended Data Fig. 4 Statistical Source Data.

Source Data Extended Data Fig. 5 Statistical Source Data.

Source Data Extended Data Fig. 6 Statistical Source Data.

Source Data Extended Data Fig. 8 Statistical Source Data.

Source Data Extended Data Fig. 9 Statistical Source Data.

## Data Availability

The authors declare that the data supporting the findings of this study are available within the paper, its Supplementary Information files and its Source Data files. Should any raw data files be needed in another format they are available from the corresponding authors upon reasonable request. Source data are provided with this paper.
