# Supplementary material for: A series of spontaneously blinking dyes for super-resolution microscopy
Source: Nat Methods. 2026 Apr 15;23(5):909–13. doi: 10.1038/s41592-026-03062-5 (PMC13167466; doi:10.1038/s41592-026-03062-5)

nature methods

Supplementary information https://doi.org/10.1038/s41592-026-03062-5

A series of spontaneously blinking dyes for super-resolution microscopy

In the format provided by the authors and unedited

SUPPLEMENTARY NOTE 1

Synthetic Efforts Towards HM-SiFl Ditriflates

Page	Contents
2	Schemes S1–S4 and Table S1
4	Summary: Development of a Concise Synthetic Route to HM-Xanthenes
8	General Experimental Information for Synthesis
9–20	Experimentals and Characterization Data for All Compounds
9	Ketone and Aryl Iodide Intermediates
13	Xanthenes via Optimized Grignard Additions
18	HM-Si-Fluorescein Ditriflates **S4** and **10**
21	References
24–39	NMR and HPLC/MS

SUPPLEMENTARY NOTE 2

Synthesis and Characterization of JFb Dyes

Page	Contents
40–59	Experimentals and Characterization Data for All Compounds
40	JFb Synthesis via Pd-Catalyzed C–N Cross-Coupling
48	Deprotection of JFb *tert*-Butyl Esters
52	Synthesis of JFb–HaloTag Ligands and Other Derivatives
60–118	NMR and HPLC/MS

SUPPLEMENTARY NOTE 1

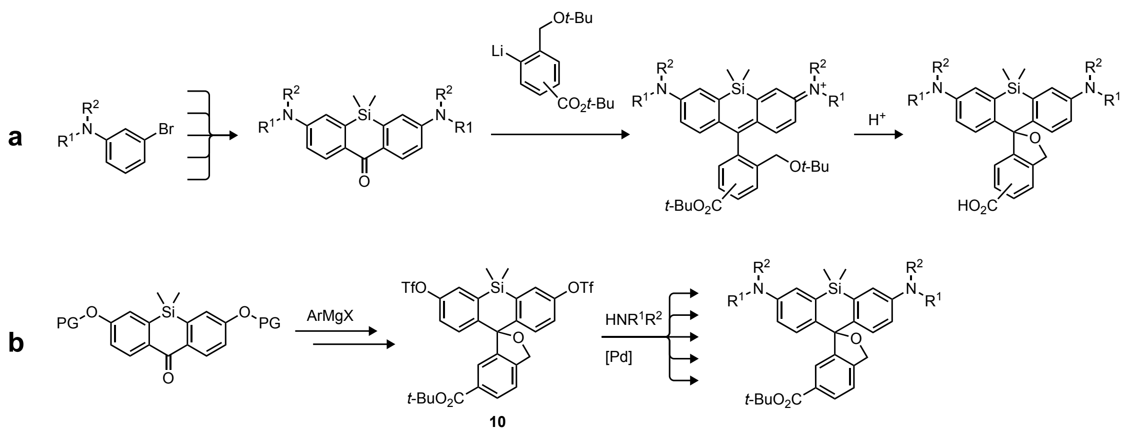

Scheme S1. Synthetic approaches to hydroxymethyl-Si-rhodamines (HM-SiR). (a) Existing strategy for HM-SiR synthesis via aryllithium addition to diamino-Si-xanthones. (b) Divergent C–N cross-coupling approach to HM-SiRs employing a common, late-stage ditriflate intermediate **10**.

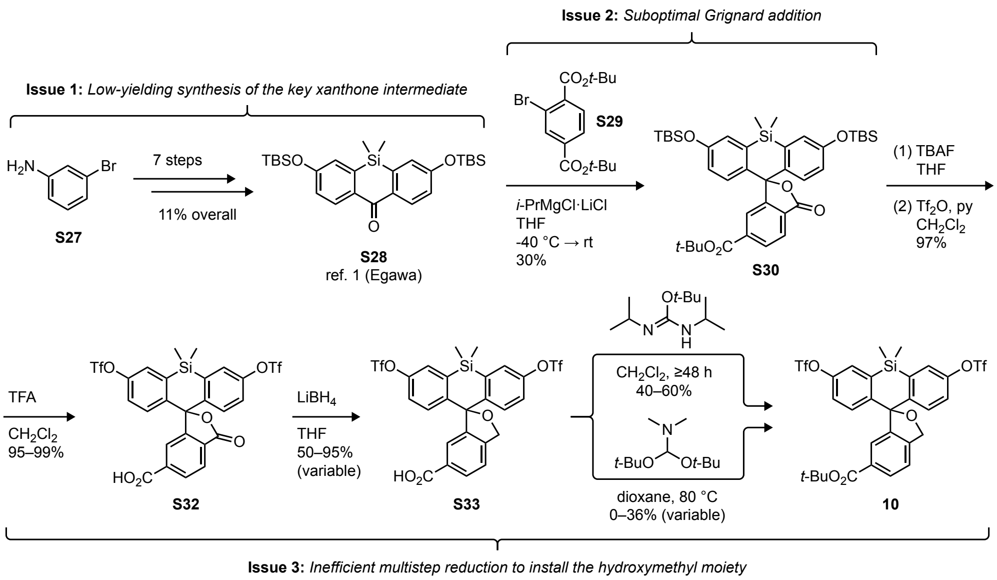

Scheme S2. First-generation synthesis of ditriflate intermediate **10**, annotated to highlight shortcomings targeted for optimization: (1) xanthone synthesis[1]; (2) Grignard addition; and (3) hydroxymethyl installation.

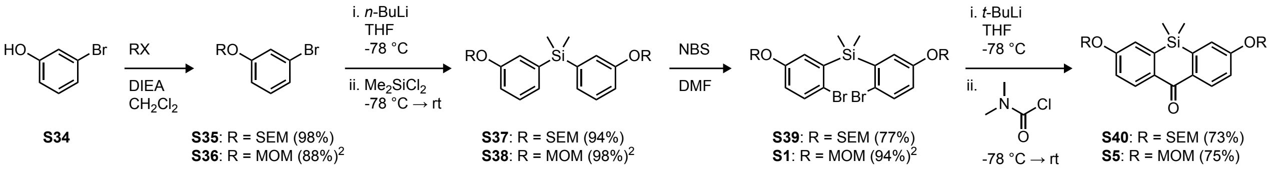

Scheme S3. Four-step synthesis of acetal-protected Si-xanthones **S5** and **S40** from 3-bromophenol (**S34**); synthesis of intermediates **S36**, **S38**, and **S1** from previous work.[2]

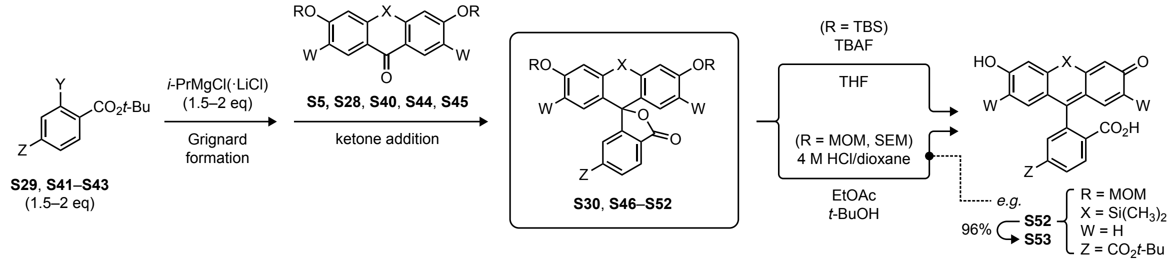

entry	halide	Y	Z	ketone	X	R	W	Grignard formation	ketone addition	product	yield (%)	
1	**S41**	Br	H	**S44**	$C(CH_3)_2$	TBS	H	*i*-PrMgCl·LiCl, -15 °C; -5 °C, 6 h	-5 °C; rt, 1 h	**S46**	56 ref 3	"Turbo Grignard" + ArBr
2	**S29**	Br	CO_2*t*-Bu	**S44**	$C(CH_3)_2$	TBS	H	*i*-PrMgCl·LiCl, -15 °C; -10 °C, 4 h	-10 °C; rt, 2 h	**S47**	17 ref 4	
3	**S41**	Br	H	**S45**	$C(CH_3)_2$	TBS	F	*i*-PrMgCl·LiCl, -15 °C; -5 °C, 6 h	-5 °C; rt, 0.5 h	**S48**	50 ref 5	
4	**S29**	Br	CO_2*t*-Bu	**S45**	$C(CH_3)_2$	TBS	F	*i*-PrMgCl·LiCl, -50 °C; -40 °C, 4 h	-40 °C; rt, 2 h	**S49**	31 ref 6	
5	**S41**	Br	H	**S28**	$Si(CH_3)_2$	TBS	H	*i*-PrMgCl·LiCl, -15 °C; -5 °C, 5 h	-5 °C; rt, 0.5 h	**S50**	56 ref 7	
6	**S29**	Br	CO_2*t*-Bu	**S28**	$Si(CH_3)_2$	TBS	H	*i*-PrMgCl·LiCl, -50 °C; -40 °C, 2 h	-40 °C; rt, 2 h	**S30**	30 ref 7	
7	**S42**	I	H	**S44**	$C(CH_3)_2$	TBS	H	*i*-PrMgCl, -40 °C; ↑ -25 °C, 2 h	-25 °C; rt, 1 h	**S46**	72	standard Grignard + ArI
8	**S43**	I	CO_2*t*-Bu	**S44**	$C(CH_3)_2$	TBS	H	*i*-PrMgCl, -40 °C; ↑ -25 °C, 2 h	-25 °C; rt, 3 h	**S47**	50	
9	**S42**	I	H	**S45**	$C(CH_3)_2$	TBS	F	*i*-PrMgCl, -40 °C; ↑ -25 °C, 2 h	-25 °C; rt, 0.5 h	**S48**	80	
10	**S43**	I	CO_2*t*-Bu	**S45**	$C(CH_3)_2$	TBS	F	*i*-PrMgCl, -40 °C; ↑ -25 °C, 2 h	-25 °C; rt, 2 h	**S49**	68	
11	**S42**	I	H	**S28**	$Si(CH_3)_2$	TBS	H	*i*-PrMgCl, -40 °C; ↑ -25 °C, 2 h	-25 °C; rt, 1 h	**S50**	94	
12	**S43**	I	CO_2*t*-Bu	**S28**	$Si(CH_3)_2$	TBS	H	*i*-PrMgCl, -40 °C; ↑ -25 °C, 2 h	-25 °C; rt, 3 h	**S30**	79	
13	**S43**	I	CO_2*t*-Bu	**S40**	$Si(CH_3)_2$	SEM	H	*i*-PrMgCl, -40 °C; ↑ -25 °C, 2 h	-25 °C; rt, 2 h	**S51**	81	
14	**S43**	I	CO_2*t*-Bu	**S5**	$Si(CH_3)_2$	MOM	H	*i*-PrMgCl, -40 °C; ↑ -25 °C, 2 h	-25 °C; rt, 2 h	**S52**	85	

Table S1. Optimization of RMgX addition to anthrones and Si-xanthones for the synthesis of carbo- and Si-fluoresceins. Entries 1–6 summarize our prior work[3-7] using the Turbo Grignard reagent with aryl bromides; entries 7–14 illustrate the substantial improvement seen in this report with standard *i*-PrMgCl and the analogous aryl iodides.

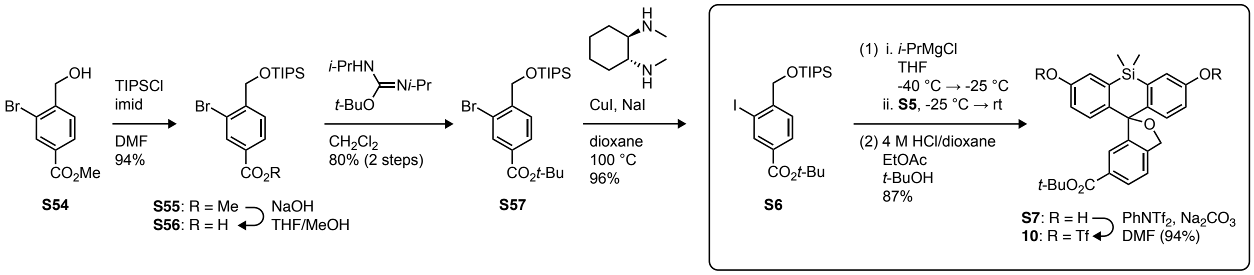

Scheme S4. Second-generation route to ditriflate **10** featuring direct installation of the hydroxymethyl functionality using the improved Grignard protocol with iodide **S6** and subsequent addition to the readily accessible ketone **S5**.

Summary: Development of a Concise Synthetic Route to HM-Xanthenes

Fine-tuning the blinking properties of the JFb scaffold was contingent on our ability to efficiently synthesize a variety of hydroxymethyl-Si-rhodamines (HM-SiRs) containing diversely substituted azetidine and pyrrolidine auxochromes. To that end, we sought a synthetic route that would enable incorporation of the diversity element—the 3′/6′ amine auxochrome substituent—at a late stage (**Scheme S1**). Unfortunately, the previously reported HM-SiR syntheses begin with a bromoaniline (**Scheme S1a**), such that the auxochrome NR^1R^2 is set during the first step of the synthesis.[8-11] The bromoaniline is typically converted into a diamino-Si-xanthone through a 3–4 step synthesis, and installation of the pendant phenyl ring is generally achieved, often in modest yield, via addition of an aryllithium reagent bearing an *ortho*-hydroxymethyl substituent protected as a *tert*-butyl ether. Deprotection of the *tert*-butyl ether to give the hydroxymethyl dye requires strong acid and extended reaction time (*e.g.*, neat TFA, >24 h; **Scheme S1a**). This was problematic for our purposes due to the propensity of azetidinyl rhodamines—particularly those with electron-withdrawing substituents on the 4-membered ring—to undergo ring-opening and other side reactions under harshly acidic conditions.

We instead pursued a more divergent synthetic strategy focusing on late-stage incorporation of the amine substituents through Pd-catalyzed C–N cross-coupling (**Scheme S1b**). In previous reports, we have extensively shown that the cross-coupling of fluorescein ditriflates with nitrogen nucleophiles is an efficient method for the synthesis of traditional lactone (*o*-carboxyl) rhodamines. Based on this—as well as preliminary work[12] describing the synthesis of a spontaneously blinking green dye (HM-JF_{526})—we surmised that hydroxymethyl-Si-fluorescein (HM-SiFl) ditriflate **10** could serve as the common intermediate for cross-coupling with various amines, thereby setting the auxochrome at the latest possible stage. This intermediate also incorporates the essential function handle on the bottom ring for ligand attachment and bioconjugation (*i.e.*, a 6-carboxylic acid protected as a *tert*-butyl ester). We expected that **10** would be most efficiently accessed by reaction of a protected dihydroxy-Si-xanthone with an appropriately functionalized aryl Grignard (**Scheme S1b**), which would better accommodate a wider range of protective functionality than an aryllithium reagent. We note that the analogous triflate **S4** for synthesis of the unsubstituted ("free") dyes **1**–**9** was more straightforward to synthesize due to the absence of the 6-ester substituent; its synthesis is summarized in **Extended Data Figure 1** and reported in **HM-Si-Fluorescein Ditriflates S4 and 10** (*vide infra*).

The route to ditriflate **10** underwent several optimizations, and the evolution of its synthesis is presented in **Schemes S2–S4** and **Table S1**. Our initial approach (**Scheme S2**) proceeded through the previously reported[7] Si-fluorescein ditriflate **S31**, which is itself prepared through addition of the Grignard derived from di-*tert*-butyl 2-bromoterephalate (**S29**) to the known[1] Si-xanthone **S28**. Consistent with our previous synthesis of an HM-rhodamine,[12] we found that the lactone could be selectively reduced (in modest and variable yield) to the cyclic ether with $LiBH_4$, but only by temporarily removing the *tert*-butyl protection on the 6-carboxyl group (**S31→S32→S33**). This then required reinstallation of the *tert*-butyl ester in the presence of the hydrolytically sensitive triflates to give the desired intermediate (**10**). Although this relatively long, modest-yielding synthesis was initially adequate for accessing small amounts of select JFb dyes, more substantial SAR exploration necessitated a better route. Hence, we optimized this route by addressing three distinct issues (**Schemes S3–S4**, **Table S1**) summarized below.

(1) **Low-yielding synthesis of the key xanthone intermediate (Scheme S3).** Si-xanthone **S28** was a crucial intermediate in our previous synthetic efforts towards azetidinyl-Si-rhodamines. To access this material, we made use of what was, at the time, the only published synthesis: Nagano's 2011 report of a 7-step route with an overall yield of 11% (**Scheme S2**).[1] Since that time, several shorter and more efficient syntheses have been reported.[13-16] Most of these rely on an approach similar to one first described by our laboratory[2] for the synthesis of Si-fluoresceins and Si-rhodamines; namely, the double lithium-bromide exchange of bis(2-bromophenyl)silanes and addition to a phosgene equivalent such as dimethylcarbamoyl chloride (**Scheme S3**). However, the use of silyl ether protecting groups on the phenols—particularly TBS—often results in diminished yields[15] or requires reprotection of the crude product following ketone installation[16] (*i.e.*, the lithium-bromide exchange and electrophile addition step).

With this in mind, we chose to protect the phenols with more robust acetal protecting groups. As shown in **Scheme S3**, the acetals (MOM and SEM) were installed in the first step through facile alkylation of 3-bromophenol with MOM-Br or SEM-Cl. Lithium-bromide exchange with *n*-butyllithium and addition to Me_2SiCl_2 afforded effectively quantitative yields (94–98%) of silanes **S37** and **S38**, which were regioselectively brominated with NBS/DMF to give the corresponding dibromides (**S39** and **S1**). Finally, lithium-bromide exchange with *tert*-butyllithium and addition to dimethylcarbamoyl chloride yielded the Si-xanthones **S40** and **S5** in excellent yield (73–75%). The choice of alkyllithium reagent was important, as *tert*-butyllithium gave substantially higher yields and cleaner reactions than *n*-butyllithium. This route to Si-xanthones represents a substantial improvement over the original synthesis[1] in both length (7 steps → 4 steps) and overall yield (SEM: 11% → 52%; MOM: 11% → 61%). Although the syntheses of both acetal-protected ketones were straightforward, we ultimately found MOM_2-Si-xanthone **S5** to be the more convenient choice due to easier chromatography, the smaller size of the protecting group, and the solid/crystalline states of the MOM dibromide **S1** and ketone **S5** (as compared to the SEM-protected viscous oils **S39** and **S40**).

(2) **Suboptimal Grignard addition (Table S1).** The key step in most existing syntheses of carbo- and Si-fluoresceins is the installation of the bottom (pendant) phenyl ring through addition of an arylmetal species to a protected dihydroxy anthrone/Si-xanthone. Although both aryllithium and aryl Grignard reagents will add to these ketones, the milder, less nucleophilic Grignards are safer and more tolerant of the *tert*-butyl esters commonly used to protect the necessary carboxyl substituents. In contrast, aryllithiums routinely require either (a) careful selection of alkyllithium reagent (*n*-/*sec*-/*tert*-butyllithium) with oftentimes extreme, inconvenient temperatures (-100 °C to -150 °C) to tolerate *t*-butyl protection,[14,17-19] or (b) more robust protecting groups (*e.g.*, oxazoline, orthoester) that can be challenging to remove.[2,18,20-22] (It is important to note that aryllithiums are typically *required* when using this approach for the synthesis of *rhodamines*, as most aryl Grignards are not reactive enough to add to diaminoanthrones or -xanthones.[22,23]) As a result, we have typically favored the use of Grignards for the synthesis of carbo- and Si-fluoresceins.[3-7] We previously accessed the necessary Grignard reagents via magnesium-bromide exchange of aryl bromides such as *tert*-butyl 2-bromobenzoate (**S41**) or di-*tert*-butyl 2-bromoterephthalate (**S29**) with Knochel's "Turbo Grignard" reagent *i*-PrMgCl·LiCl (**Scheme S2**, **Table S1**), as Mg/Br-exchange of aryl bromides with standard *i*-PrMgCl is often very slow.[24] We used these aryl Grignards with several dihydroxyanthrones and dihydroxy-Si-

xanthones (**S28**, **S44**, **S45**) over the past decade to synthesize a number of carbofluoresceins[3,5,6] (**Table S1**, entries 1–4) and Si-fluoresceins[4,7] (**Table S1**, entries 5–6).

This method, however, presented several opportunities for improvement. Firstly, the yields obtained from the Mg/Br-exchange and ketone addition were quite variable (**Table S1**, entries 1-6), ranging from low (17%, entry 2) to moderate (56%, entries 1, 5). In our hands, the commercial Turbo Grignard reagent displayed limited and inconsistent shelf stability once opened (as compared to a standard Grignard or organolithium). Perhaps consequently, the Mg/Br-exchange rarely consumed the bromide in its entirety, regardless of temperature or extended reaction time. The addition step also seldom proceeded to completion, with significant ketone starting material recovered even when using a substantial excess of Grignard (>2 eq). In the case of the present work, the modest yield of Si-fluorescein **S30** obtained via the Turbo Grignard route (30%, entry 6) was a significant bottleneck in the synthesis of ditriflate **10** (**Scheme S2**).

Given these results, we considered whether switching to an aryl halide with more facile reactivity in the magnesium-halide exchange could significantly improve yield and conversion. It is well-established that aryl iodides undergo metal-halide exchange with Grignard reagents (*e.g, i*-PrMgCl) faster than aryl bromides; in fact, Mg/I-exchange of iodides in the *presence* of bromides is a reliable method to prepare bromoaryl Grignards from bromoiodoarenes.[25-27] Indeed, when we substituted *tert*-butyl iodobenzoate (**S42**) or di-*tert*-butyl iodoterephthalate (**S43**) for the corresponding bromides and used *i*-PrMgCl in place of *i*-PrMgCl·LiCl, we observed a dramatic improvement across all six examples (**Table S1**, entries 7–12). The Mg/I-exchanges were cleaner and faster (-40 °C → -25 °C, ≤ 2 h), negligible amounts of unreacted ketone were recovered, and the yields of the fluorescein products were greatly enhanced (up to 3× higher). Importantly, the yield of Si-fluorescein **S30**, the key intermediate in our initial route to ditriflate **10** (**Scheme S2**), was increased to 79% by this modification (entry 6 → entry 12). TBS-protected ketones were used in entries 7–12 to be most consistent with our previous work[3-7] (entries 1–6) and enable direct comparison of the efficiencies of the two Grignard protocols.

Given the improved Si-xanthone synthesis described in (1) above, we then applied the same method to the SEM- and MOM-protected Si-xanthones **S40** and **S5** (**Table S1**, entries 13–14). Both smoothly reacted with the Grignard reagent derived from di-*tert*-butyl 2-iodoterephthalate (**S43**) to afford even better yields of Si-fluoresceins **S51** (81%) and **S52** (85%). Since acetal (*e.g.*, MOM) deprotection is commonly achieved with acid, we were initially concerned that removal of the MOM group in the presence of the *tert*-butyl ester—which was required for subsequent ditriflate formation and, ultimately, the cross-coupling—might prove difficult. Gratifyingly, several conditions were found that could effectively remove the acetal without disturbing the ester, including *p*-TsOH in MeOH (24–48 h, 25 °C)[28,29] and 4 M HCl/dioxane in 2:1 EtOAc/*t*-BuOH (< 2 h, 25 °C).[30] For example, the faster HCl/dioxane conditions promoted rapid deprotection of MOM_2-Si-fluorescein **S52** to afford a nearly quantitative (96%) yield of 6-*tert*-butoxycarbonyl-Si-fluorescein **S53** (**Table S1**; see **Xanthenes via Optimized Grignard Addition**). Curiously, our efforts to effect a more orthogonal and chemospecific deprotection of SEM_2-Si-fluorescein **S51** with fluoride (*e.g.*, TBAF) were unsuccessful; nonetheless, the SEM acetals could be easily removed under the same acid-mediated conditions used for the MOM analog (see **S53** in **Xanthenes via Optimized Grignard Addition**).

(3) **Inefficient multistep reduction to install the hydroxymethyl moiety (Scheme S4).** With a dramatically improved method for installation of the bottom phenyl ring in hand, we then turned our focus to the final transformation in our initial synthesis of **10** (**Scheme S2**): the circuitous, 3-step reduction protocol used to convert Si-fluorescein **S31** to its hydroxymethyl (cyclic ether) congener **10**. In order the selectively reduce the lactone to the cyclic ether, it was essential to remove the *tert*-butyl protecting group from the 6-carboxyl group. Unfortunately, this then required post-reduction reinstallation of the *tert*-butyl ester in the presence of two labile aryl triflates. Even the most mild reagents for carboxyl *tert*-butylation—most notably *tert*-butyl *N*,*N'*-diisopropylcarbamimidate[31] and *N*,*N*-dimethylformamide di-*tert*-butyl acetal[32]—required elevated temperature and/or extended reaction time to achieve reasonable conversion to **10**, and yields were variable and moderate at best (due in no small part to triflate hydrolysis, particularly with the DMF acetal). Moreover, the $LiBH_4$ reduction itself also exhibited variability in conversion and yield, sometimes providing ≤ 50% of the reduced intermediate **S33**.

In light of these issues, we considered whether we might eschew the suboptimal reduction and protecting group shuffling entirely by directly installing the *o*-hydroxymethyl substituent during the Grignard addition (**Scheme S4**). To that end, we synthesized a candidate aryl iodide (**S6**, Scheme S4) through a simple 4-step route from the commercially available benzoate **S54**. Straightforward TIPS protection of the free hydroxymethyl group afforded a 94% yield of silyl ether **S55**; the methyl ester was converted to a *tert*-butyl ester via simple 2-step saponification and *tert*-butylation with *tert*-butyl *N*,*N'*-diisopropylcarbamimidate (**S57**; 80% yield, 2 steps). Lastly, halogen exchange of **S57** using Buchwald's Cu(I)-catalyzed aromatic Finkelstein reaction[33] cleanly provided the desired iodide intermediate **S6** in excellent yield (96%).

When subjected to the optimized Grignard conditions, iodide **S6** underwent facile magnesium-iodide exchange with *i*-PrMgCl and efficient addition to MOM_2-Si-xanthone **S5**. Rather than purify the initial addition product and remove the TIPS and MOM protecting groups in a stepwise fashion, we opted to directly subject the crude Grignard addition product to the MOM deprotection conditions discussed above (4 M HCl/dioxane in EtOAc/*t*-BuOH). Conveniently, all the protecting groups save the *tert*-butyl ester were cleanly removed to provide, following purification, an 87% yield of HM-Si-fluorescein **S7**. Finally, trivial installation of the triflates with *N*-phenyl-bis(trifluoromethanesulfonimide) afforded the long-sought HM-SiFl ditriflate **10** (94%). **Scheme S4**, then, represents our current and most optimized route to this key cross-coupling substrate (see **Supplementary Note 2**).

In comparing our original (**Scheme S2**) and final (**Scheme S4**) syntheses, we note that systematically addressing three major flaws of the initial route to ditriflate **10** has achieved both a dramatic improvement in efficiency (13 steps → 6 steps, longest linear sequence) and overall yield (< 2% → 50%). We expect that these new and modified synthetic approaches will be of significant interest and use to the many fluorophore chemists investigating not only HM-xanthenes specifically, but also the ever-widening variety of xanthene dyes in general.

General Experimental Information for Synthesis

Commercial reagents were obtained from reputable suppliers and used as received. All solvents were purchased in septum-sealed bottles stored under an inert atmosphere. All reactions were sealed with septa through which a nitrogen atmosphere was introduced unless otherwise noted. Reactions were conducted in round-bottomed flasks or septum-capped crimp-top vials containing Teflon-coated magnetic stir bars. Heating of reactions was accomplished with a silicon oil bath or an aluminum reaction block on top of a stirring hotplate equipped with an electronic contact thermometer to maintain the indicated temperatures.

Reactions were monitored by thin layer chromatography (TLC) on precoated TLC glass plates (silica gel 60 F_{254}, 250 µm thickness) or by LC/MS (Phenomenex Kinetex 2.1 mm × 30 mm 2.6 µm C18 column; 5 µL injection; 5–98% $MeCN/H_2O$, linear gradient, with constant 0.1% v/v HCO_2H additive; 6 min run; 0.5 mL/min flow; ESI; positive ion mode). TLC chromatograms were visualized by UV illumination or developed with *p*-anisaldehyde, ceric ammonium molybdate, or $KMnO_4$ stain. Reaction products were purified by flash chromatography on an automated purification system using pre-packed silica gel columns or by preparative HPLC (Phenomenex Gemini–NX 30 × 150 mm 5 µm C18 column). Analytical HPLC analysis was performed with an Agilent Eclipse XDB 4.6 × 150 mm 5 µm C18 column under the indicated conditions. High-resolution mass spectrometry was performed by the High Resolution Mass Spectrometry Facility at the University of Iowa.

NMR spectra were recorded on a 400 MHz spectrometer. ^{1}H and ^{13}C chemical shifts were referenced to TMS or residual solvent peaks, and ^{19}F chemical shifts were referenced to $CFCl_3$. Data for ^{1}H NMR spectra are reported as follows: chemical shift (δ ppm), multiplicity (s = singlet, d = doublet, t = triplet, q = quartet, dd = doublet of doublets, m = multiplet), coupling constant (Hz), integration. Data for ^{13}C NMR spectra are reported by chemical shift (δ ppm) with hydrogen multiplicity (C, CH, CH_2, CH_3) information obtained from DEPT spectra.

Ketone and Aryl Iodide Intermediates

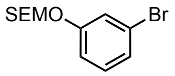

(2-((3-Bromophenoxy)methoxy)ethyl)trimethylsilane (S35): 3-Bromophenol (**S34**; 7.50 g, 43.35 mmol) was taken up in CH_2Cl_2 (100 mL); DIEA (11.33 mL, 65.03 mmol, 1.5 eq) and 2-(trimethylsilyl)ethoxymethyl chloride (11.51 mL, 65.03 mmol, 1.5 eq) were successively added, and the reaction was stirred at room temperature for 2 h. It was subsequently diluted with water and extracted with CH_2Cl_2 (2×). The combined organic extracts were washed with saturated $NaHCO_3$ and brine, dried over anhydrous $MgSO_4$, filtered, and evaporated. Flash chromatography (0–20% Et_2O/hexanes, linear gradient) afforded **S35** as a colorless oil (12.92 g, 98%). ^{1}H NMR ($CDCl_3$, 400 MHz) δ 7.24 – 7.20 (m, 1H), 7.17 – 7.10 (m, 2H), 7.02 – 6.91 (m, 1H), 5.20 (s, 2H), 3.78 – 3.71 (m, 2H), 0.99 – 0.92 (m, 2H), 0.00 (s, 9H); ^{13}C NMR ($CDCl_3$, 101 MHz) δ 158.4 (C), 130.7 (CH), 124.9 (CH), 122.8 (C), 119.7 (CH), 115.2 (CH), 93.1 (CH_2), 66.6 (CH_2), 18.2 (CH_2), -1.3 (CH_3); HRMS (EI) calcd for $C_{12}H_{19}BrO_2Si$ $[M]^{+\bullet}$ 302.0333/304.0312, found 302.0332/304.0311.

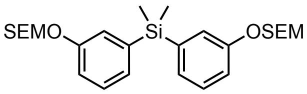

Dimethylbis(3-((2-(trimethylsilyl)ethoxy)methoxy)phenyl)silane (S37): A solution of (2-((3-bromophenoxy)methoxy)ethyl)trimethylsilane (**S35**; 12.50 g, 41.22 mmol, 2.4 eq) in THF (100 mL) was cooled to -78 °C under nitrogen. *n*-Butyllithium (1.6 M in hexanes, 25.76 mL, 41.22 mmol, 2.4 eq) was added, and the reaction was stirred at -78 °C for 30 min. Dichlorodimethylsilane (2.07 mL, 17.17 mmol) was then added. The dry ice bath was removed, and the reaction was stirred at room temperature for 2 h. It was subsequently quenched with saturated NH_4Cl, diluted with water, and extracted with EtOAc (2×). The combined organic extracts were washed with brine, dried over anhydrous $MgSO_4$, filtered, and concentrated *in vacuo*. Purification by flash chromatography on silica gel (0–10% Et_2O/hexanes, linear gradient) afforded 8.12 g (94%) of **S37** as a colorless oil. ^{1}H NMR ($CDCl_3$, 400 MHz) δ 7.27 (ddd, J = 8.2, 7.2, 0.5 Hz, 2H), 7.18 (ddd, J = 2.7, 1.1, 0.4 Hz, 2H), 7.14 (dt, J = 7.2, 1.1 Hz, 2H), 7.06 (ddd, J = 8.2, 2.6, 1.1 Hz, 2H), 5.20 (s, 4H), 3.78 – 3.72 (m, 4H), 0.98 – 0.92 (m, 4H), 0.53 (s, 6H), 0.00 (s, 18H); ^{13}C NMR ($CDCl_3$, 101 MHz) δ 157.2 (C), 140.0 (C), 129.2 (CH), 127.7 (CH), 122.3 (CH), 116.8 (CH), 93.2 (CH_2), 66.3 (CH_2), 18.2 (CH_2), -1.3 (CH_3), -2.3 (CH_3); HRMS (ESI) calcd for $C_{26}H_{44}O_4Si_3Na$ $[M+Na]^+$ 527.2440, found 527.2438.

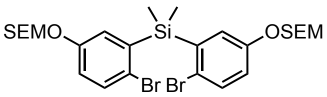

Bis(2-bromo-5-((2-(trimethylsilyl)ethoxy)methoxy)phenyl)dimethylsilane (S39): Dimethylbis(3-((2-(trimethylsilyl)ethoxy)methoxy)phenyl)silane (**S37**; 8.00 g, 15.85 mmol) was taken up in DMF (100 mL). *N*-Bromosuccinimide (5.64 g, 31.69 mmol, 2 eq) was added portion-wise over 5 min, and the reaction was then stirred at room temperature for 96 h. The reaction mixture was concentrated *in vacuo*; the resulting residue was diluted with water and extracted with EtOAc (2×). The combined organic extracts were washed with water and brine, dried over

anhydrous $MgSO_4$, filtered, and concentrated *in vacuo*. Silica gel chromatography (0–10% Et_2O/hexanes, linear gradient) afforded 8.09 g (77%) of **S39** as a pale yellow oil. ^{1}H NMR ($CDCl_3$, 400 MHz) δ 7.40 (d, J = 8.7 Hz, 2H), 7.14 (d, J = 3.0 Hz, 2H), 6.95 (dd, J = 8.7, 3.1 Hz, 2H), 5.17 (s, 4H), 3.76 – 3.70 (m, 4H), 0.97 – 0.90 (m, 4H), 0.73 (s, 6H), 0.00 (s, 18H); ^{13}C NMR ($CDCl_3$, 101 MHz) δ 156.3 (C), 140.2 (C), 133.8 (CH), 125.8 (CH), 121.9 (C), 118.8 (CH), 93.3 (CH_2), 66.4 (CH_2), 18.2 (CH_2), -1.1 (CH_3), -1.2 (CH_3); HRMS (ESI) calcd for $C_{26}H_{42}Br_2O_4Si_3Na$ $[M+Na]^+$ 683.0650/685.0630/687.0609, found 683.0646/685.0625/687.0607.

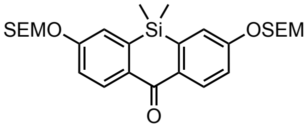

5,5-Dimethyl-3,7-bis((2-(trimethylsilyl)ethoxy)methoxy)dibenzo[*b*,*e*]silin-10(5*H*)-one (S40): A solution of bis(2-bromo-5-((2-(trimethylsilyl)ethoxy)methoxy)phenyl)dimethylsilane (**S39**; 5.50 g, 8.30 mmol) in THF (80 mL) was cooled to -78 °C under nitrogen. *tert*-Butyllithium (1.7 M in pentane, 21.48 mL, 36.52 mmol, 4.4 eq) was added, and the reaction was stirred at -78 °C for 30 min. Dimethylcarbamoyl chloride (841 µL, 9.13 mmol, 1.1 eq) was added; the reaction was then gradually warmed to room temperature over 4 h. It was subsequently quenched with saturated NH_4Cl, diluted with water, and extracted with EtOAc (2×). The combined organic extracts were washed with brine, dried over anhydrous $MgSO_4$, filtered, and concentrated *in vacuo*. Purification by flash chromatography on silica gel (0–20% Et_2O/hexanes, linear gradient) yielded 3.21 g (73%) of **S40** as a colorless gum. ^{1}H NMR ($CDCl_3$, 400 MHz) δ 8.42 (d, J = 8.8 Hz, 2H), 7.23 (d, J = 2.5 Hz, 2H), 7.20 (dd, J = 8.8, 2.6 Hz, 2H), 5.31 (s, 4H), 3.82 – 3.75 (m, 4H), 1.00 – 0.93 (m, 4H), 0.47 (s, 6H), 0.00 (s, 18H); ^{13}C NMR ($CDCl_3$, 101 MHz) δ 186.0 (C), 160.1 (C), 141.4 (C), 134.9 (C), 132.4 (CH), 119.7 (CH), 117.7 (CH), 92.8 (CH_2), 66.8 (CH_2), 18.2 (CH_2), -1.2 (CH_3), -1.3 (CH_3); HRMS (ESI) calcd for $C_{27}H_{43}O_5Si_3$ $[M+H]^+$ 531.2413, found 531.2412.

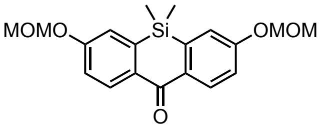

3,7-Bis(methoxymethoxy)-5,5-dimethyldibenzo[*b*,*e*]silin-10(5*H*)-one (S5): A solution of bis(2-bromo-5-(methoxymethoxy)phenyl)dimethylsilane[2] (**S1**; 6.00 g, 12.24 mmol) in THF (100 mL) was cooled to -78 °C under nitrogen. *tert*-Butyllithium (1.7 M in pentane, 31.68 mL, 53.85 mmol, 4.4 eq) was added, and the reaction was stirred at -78 °C for 30 min. Dimethylcarbamoyl chloride (1.24 mL, 13.46 mmol, 1.1 eq) was added; the reaction was then gradually warmed to room temperature over 4 h. It was subsequently quenched with saturated NH_4Cl, diluted with water, and extracted with EtOAc (2×). The combined organic extracts were washed with brine, dried over anhydrous $MgSO_4$, filtered, and concentrated *in vacuo*. Purification by flash chromatography on silica gel (0–40% EtOAc/hexanes, linear gradient) yielded 3.31 g (75%) of **S5** as a white solid. ^{1}H NMR ($CDCl_3$, 400 MHz) δ 8.43 (d, J = 8.7 Hz, 2H), 7.23 (d, J = 2.6 Hz, 2H), 7.20 (dd, J = 8.7, 2.7 Hz, 2H), 5.28 (s, 4H), 3.52 (s, 6H), 0.48 (s, 6H); ^{13}C NMR ($CDCl_3$, 101 MHz) δ 185.9 (C), 159.8 (C), 141.4 (C), 135.0 (C), 132.4 (CH), 119.8 (CH), 117.6 (CH), 94.3 (CH_2), 56.5 (CH_3), -1.3 (CH_3); HRMS (ESI) calcd for $C_{19}H_{23}O_5Si$ $[M+H]^+$ 359.1309, found 359.1308.

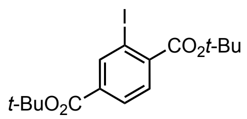

Di-*tert*-butyl 2-iodoterephthalate (S43): A solution of 2-iodoterephthalic acid (12.00 g, 41.1 mmol) in dioxane (80 mL) was heated to 80 °C, and *N,N*-dimethylformamide di-*tert*-butyl acetal (78.82 mL, 328.7 mmol, 8 eq) was added dropwise over 15 min. The reaction was stirred at 80 °C for an additional 1 h. After cooling the solution to room temperature, it was diluted with saturated $NaHCO_3$ and extracted with EtOAc (2×). The combined organic extracts were washed with water and brine, dried over anhydrous $MgSO_4$, filtered, and evaporated. Flash chromatography (0–10% Et_2O/hexanes, linear gradient) provided **S43** as a pale yellow, viscous oil (15.80 g, 95%). ^{1}H NMR ($CDCl_3$, 400 MHz) δ 8.49 (d, *J* = 1.6 Hz, 1H), 7.96 (dd, *J* = 8.0, 1.6 Hz, 1H), 7.66 (d, *J* = 8.0 Hz, 1H), 1.63 (s, 9H), 1.59 (s, 9H); ^{13}C NMR ($CDCl_3$, 101 MHz) δ 166.0 (C), 163.7 (C), 141.7 (CH), 141.1 (C), 134.9 (C), 130.0 (CH), 128.9 (CH), 92.7 (C), 83.4 (C), 82.3 (C), 28.3 (CH_3); HRMS (ESI) calcd for $C_{16}H_{21}IO_4Na$ $[M+Na]^+$ 427.0377, found 427.0381.

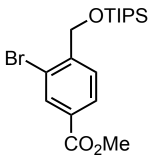

Methyl 3-bromo-4-(((triisopropylsilyl)oxy)methyl)benzoate (S55): To a solution of methyl 3-bromo-4-(hydroxymethyl)benzoate (**S54**; 7.95 g, 32.44 mmol) in DMF (100 mL) were added imidazole (4.42 g, 64.88 mmol, 2 eq) and TIPSCl (10.41 mL, 48.66 mmol, 1.5 eq). The reaction was stirred at room temperature for 4 h. It was subsequently diluted with water and extracted with Et_2O (2×). The combined organic extracts were washed with water and brine, dried over anhydrous $MgSO_4$, filtered, and concentrated *in vacuo*. Silica gel chromatography (0–10% Et_2O/hexanes, linear gradient) afforded 12.19 g (94%) of **S55** as a colorless solid. ^{1}H NMR ($CDCl_3$, 400 MHz) δ 8.17 (d, *J* = 1.6 Hz, 1H), 8.02 (dd, *J* = 8.1, 1.7 Hz, 1H), 7.72 (dt, *J* = 8.1, 1.1 Hz, 1H), 4.84 (d, *J* = 1.0 Hz, 2H), 3.92 (s, 3H), 1.27 – 1.17 (m, 3H), 1.11 (d, *J* = 7.0 Hz, 18H); ^{13}C NMR ($CDCl_3$, 101 MHz) δ 166.1 (C), 145.9 (C), 133.2 (CH), 130.2 (C), 128.7 (CH), 127.3 (CH), 120.6 (C), 65.0 (CH_2), 52.4 (CH_3), 18.2 (CH_3), 12.1 (CH); HRMS (ESI) calcd for $C_{18}H_{30}BrO_3Si$ $[M+H]^+$ 401.1142/403.1122, found 401.1139/403.1117.

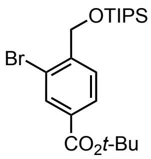

***tert*-Butyl 3-bromo-4-(((triisopropylsilyl)oxy)methyl)benzoate (S57):** Methyl 3-bromo-4-(((triisopropylsilyl)oxy)methyl)benzoate (**S55**; 11.90 g, 29.64 mmol) was dissolved in 3:1 THF/MeOH (160 mL), and 1 M NaOH (44.47 mL, 44.47 mmol, 1.5 eq) was added. The reaction was stirred at room temperature for 4 h. It was then acidified with 1 M HCl (50 mL), diluted with water, and extracted with EtOAc (2×). The combined organics were washed with brine, dried over anhydrous $MgSO_4$, filtered, and concentrated *in vacuo*. The crude carboxylic acid **S56**

was taken back up in CH_2Cl_2 (300 mL) under nitrogen, and *tert*-butyl *N*,*N'*-diisopropylcarbamimidate (29.69 g, 148.2 mmol, 5 eq) was added. After stirring the reaction at room temperature for 18 h, the resulting white suspension was diluted with hexanes (300 mL) and concentrated to half-volume. The mixture was filtered through Celite with hexanes and concentrated *in vacuo*. Purification by flash chromatography (0–50% CH_2Cl_2/hexanes, linear gradient) yielded 10.48 g (80%) of **S57** as a colorless oil. ^{1}H NMR ($CDCl_3$, 400 MHz) δ 8.10 (d, J = 1.6 Hz, 1H), 7.96 (dd, J = 8.0, 1.7 Hz, 1H), 7.70 (dt, J = 8.1, 1.1 Hz, 1H), 4.84 (d, J = 1.1 Hz, 2H), 1.59 (s, 9H), 1.26 – 1.17 (m, 3H), 1.10 (d, J = 6.9 Hz, 18H); ^{13}C NMR ($CDCl_3$, 101 MHz) δ 164.7 (C), 145.3 (C), 133.0 (CH), 132.1 (C), 128.5 (CH), 127.1 (CH), 120.4 (C), 81.6 (C), 65.0 (CH_2), 28.3 (CH_3), 18.2 (CH_3), 12.1 (CH); HRMS (ESI) calcd for $C_{21}H_{36}BrO_3Si$ $[M+H]^+$ 443.1612/445.1592, found 443.1606/445.1584.

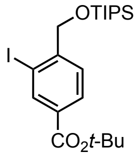

***tert*-Butyl 3-iodo-4-(((triisopropylsilyl)oxy)methyl)benzoate (S6):** An oven-dried round-bottom flask was charged with CuI (129 mg, 0.676 mmol, 0.1 eq) and NaI (2.03 g, 13.53 mmol, 2 eq). The flask was sealed and evacuated/backfilled with nitrogen (3×). A solution of *tert*-butyl 3-bromo-4-(((triisopropylsilyl)oxy)methyl)benzoate (**S57**; 3.00 g, 6.76 mmol) in dioxane (17 mL) was added, and the reaction was flushed again with nitrogen (3×). Following the addition of *trans*-*N*,*N'*-dimethylcyclohexane-1,2-diamine (213 µL, 1.35 mmol, 0.2 eq), the reaction was stirred at 110 °C for 24 h. It was then cooled to room temperature, diluted with saturated NH_4Cl and water, and extracted with EtOAc (2×). The combined organic extracts were washed with brine, dried over anhydrous $MgSO_4$, filtered, and concentrated *in vacuo*. Silica gel chromatography (0–50% CH_2Cl_2/hexanes, linear gradient) afforded **S6** (3.19 g, 96%) as a pale yellow gum. ^{1}H NMR ($CDCl_3$, 400 MHz) δ 8.36 (d, J = 1.7 Hz, 1H), 8.00 (dd, J = 8.1, 1.7 Hz, 1H), 7.64 (dt, J = 8.1, 1.1 Hz, 1H), 4.72 (d, J = 1.0 Hz, 2H), 1.59 (s, 9H), 1.26 – 1.17 (m, 3H), 1.11 (d, J = 7.0 Hz, 18H); ^{13}C NMR ($CDCl_3$, 101 MHz) δ 164.5 (C), 147.8 (C), 139.6 (CH), 132.2 (C), 129.3 (CH), 126.8 (CH), 94.7 (C), 81.6 (C), 69.9 (CH_2), 28.3 (CH_3), 18.2 (CH_3), 12.1 (CH); HRMS (ESI) calcd for $C_{21}H_{36}IO_3Si$ $[M+H]^+$ 491.1473, found 491.1470.

Xanthenes via Optimized Grignard Additions

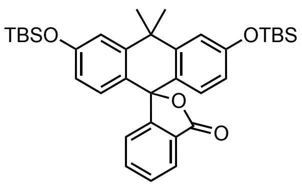

Carbofluorescein bis(*tert*-butyldimethylsilyl ether) (S46): To a solution of *tert*-butyl 2-iodobenzoate (**S42**; 1.65 g, 5.44 mmol, 1.5 eq) in THF (15 mL) at -40 °C was added *i*-PrMgCl (2.0 M in THF; 2.72 mL, 5.44 mmol, 1.5 eq). The reaction was then gradually warmed to -25 °C over 2 h while stirring. A solution of 3,6-bis((*tert*-butyldimethylsilyl)oxy)-10,10-dimethylanthracen-9(10*H*)-one[3] (**S44**; 1.75 g, 3.62 mmol) in THF (10 mL) was added dropwise; the reaction was then warmed to room temperature and stirred for 1 h. It was subsequently quenched with saturated NH_4Cl, diluted with water, and extracted with EtOAc (2×). The combined organic extracts were washed with brine, dried over anhydrous $MgSO_4$, filtered, and concentrated *in vacuo*. Flash chromatography on silica gel (0–20% Et_2O/hexanes, linear gradient) afforded 1.53 g (72%) of **S46** as a white solid. The characterization data for **S46** matched the previously reported spectra.[3]

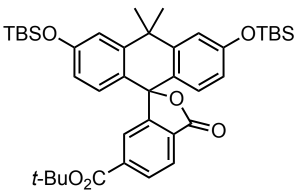

6-(*tert*-Butoxycarbonyl)carbofluorescein bis(*tert*-butyldimethylsilyl ether) (S47): To a solution of di-*tert*-butyl 2-iodoterephthalate (**S43**; 2.26 g, 5.59 mmol, 1.5 eq) in THF (15 mL) at -40 °C was added *i*-PrMgCl (2.0 M in THF; 2.80 mL, 5.59 mmol, 1.5 eq). The reaction was then gradually warmed to -25 °C over 2 h while stirring. A solution of 3,7-bis((*tert*-butyldimethylsilyl)oxy)-5,5-dimethyldibenzo[*b*,*e*]silin-10(5*H*)-one[3] (**S44**; 1.80 g, 3.73 mmol) in THF (10 mL) was added dropwise; the reaction was then warmed to room temperature and stirred for 3 h. It was subsequently quenched with saturated NH_4Cl, diluted with water, and extracted with EtOAc (2×). The combined organic extracts were washed with brine, dried over anhydrous $MgSO_4$, filtered, and concentrated *in vacuo*. Flash chromatography on silica gel (0–10% Et_2O/hexanes, linear gradient) afforded 1.28 g (50%) of **S47** as a white solid. The characterization data for **S47** matched the previously reported spectra.[4]

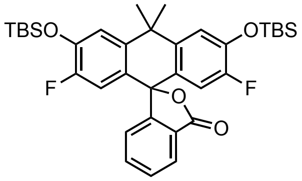

Virginia Orange bis(*tert*-butyldimethylsilyl ether) (S48): To a solution of *tert*-butyl 2-iodobenzoate (**S42**; 440 mg, 1.45 mmol, 1.5 eq) in THF (4 mL) at -40 °C was added *i*-PrMgCl (2.0 M in THF; 723 µL, 1.45 mmol, 1.5 eq). The reaction was then gradually warmed to -25 °C over 2 h while stirring. A solution of 3,6-bis((*tert*-

butyldimethylsilyl)oxy)-2,7-difluoro-10,10-dimethylanthracen-9(10*H*)-one[5] (**S45**; 500 mg, 0.964 mmol) in THF (2.5 mL) was added dropwise; the reaction was then warmed to room temperature and stirred for 30 min. It was subsequently quenched with saturated NH_4Cl, diluted with water, and extracted with EtOAc (2×). The combined organic extracts were washed with brine, dried over anhydrous $MgSO_4$, filtered, and concentrated *in vacuo*. Flash chromatography on silica gel (0–20% Et_2O/hexanes, linear gradient) afforded 479 mg (80%) of **S48** as a white foam. The characterization data for **S48** matched the previously reported spectra.[5]

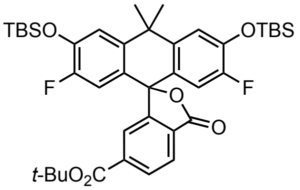

6-(*tert*-Butoxycarbonyl)-Virginia Orange bis(*tert*-butyldimethylsilyl ether) (S49): To a solution of di-*tert*-butyl 2-iodoterephthalate (**S43**; 2.34 g, 5.78 mmol, 1.5 eq) in THF (15 mL) at -40 °C was added *i*-PrMgCl (2.0 M in THF; 2.89 mL, 5.78 mmol, 1.5 eq). The reaction was then gradually warmed to -25 °C over 2 h while stirring. A solution of 3,6-bis((*tert*-butyldimethylsilyl)oxy)-2,7-difluoro-10,10-dimethylanthracen-9(10*H*)-one[5] (**S45**; 2.00 g, 3.86 mmol) in THF (10 mL) was added dropwise; the reaction was then warmed to room temperature and stirred for 2 h. It was subsequently quenched with saturated NH_4Cl, diluted with water, and extracted with EtOAc (2×). The combined organic extracts were washed with brine, dried over anhydrous $MgSO_4$, filtered, and concentrated *in vacuo*. Flash chromatography on silica gel (0–20% Et_2O/hexanes, linear gradient) afforded 1.90 g (68%) of **S49** as a white solid. The characterization data for **S49** matched the previously reported spectra.[6]

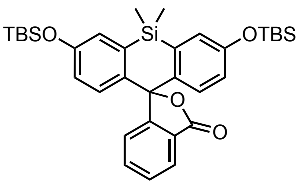

Si-Fluorescein bis(*tert*-butyldimethylsilyl ether) (S50): To a solution of *tert*-butyl 2-iodobenzoate (**S42**; 457 mg, 1.50 mmol, 1.5 eq) in THF (4 mL) at -40 °C was added *i*-PrMgCl (2.0 M in THF; 752 µL, 1.50 mmol, 1.5 eq). The reaction was then gradually warmed to -25 °C over 2 h while stirring. A solution of 3,7-bis((*tert*-butyldimethylsilyl)oxy)-5,5-dimethyldibenzo[*b*,*e*]silin-10(5*H*)-one[1] (**S28**; 500 mg, 1.00 mmol) in THF (2.5 mL) was added dropwise; the reaction was then warmed to room temperature and stirred for 1 h. It was subsequently quenched with saturated NH_4Cl, diluted with water, and extracted with EtOAc (2×). The combined organic extracts were washed with brine, dried over anhydrous $MgSO_4$, filtered, and concentrated *in vacuo*. Flash chromatography on silica gel (0–20% Et_2O/hexanes, linear gradient) afforded 566 mg (94%) of **S50** as a white solid. The characterization data for **S50** matched the previously reported spectra.[7]

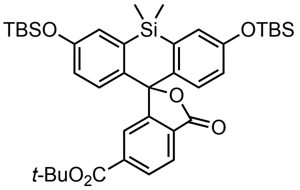

6-(*tert*-Butoxycarbonyl)-Si-fluorescein bis(*tert*-butyldimethylsilyl ether) (S30): To a solution of di-*tert*-butyl 2-iodoterephthalate (**S43**; 2.43 g, 6.01 mmol, 1.5 eq) in THF (15 mL) at -40 °C was added *i*-PrMgCl (2.0 M in THF; 3.01 mL, 6.01 mmol, 1.5 eq). The reaction was then gradually warmed to -25 °C over 2 h while stirring. A solution of 3,7-bis((*tert*-butyldimethylsilyl)oxy)-5,5-dimethyldibenzo[*b*,*e*]silin-10(5*H*)-one[1] (**S28**; 2.00 g, 4.01 mmol) in THF (10 mL) was added dropwise; the reaction was then warmed to room temperature and stirred for 3 h. It was subsequently quenched with saturated NH_4Cl, diluted with water, and extracted with EtOAc (2×). The combined organic extracts were washed with brine, dried over anhydrous $MgSO_4$, filtered, and concentrated *in vacuo*. Flash chromatography on silica gel (0–10% Et_2O/hexanes, linear gradient) afforded 2.22 g (79%) of **S30** as a white solid. The characterization data for **S30** matched the previously reported spectra.[7]

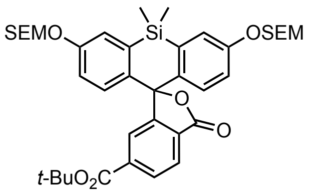

6-(*tert*-Butoxycarbonyl)-Si-fluorescein bis(trimethylsilylethoxymethyl ether) (S51): To a solution of di-*tert*-butyl 2-iodoterephthalate (**S43**; 3.05 g, 7.53 mmol, 2 eq) in THF (15 mL) at -40 °C was added *i*-PrMgCl (2.0 M in THF; 3.77 mL, 7.53 mmol, 2 eq). The reaction was then gradually warmed to -25 °C over 2 h while stirring. A solution of 5,5-dimethyl-3,7-bis((2-(trimethylsilyl)ethoxy)methoxy)dibenzo[*b*,*e*]silin-10(5*H*)-one (**S40**; 2.00 g, 3.77 mmol) in THF (10 mL) was added dropwise; the reaction was then warmed to room temperature and stirred for 2 h. It was subsequently quenched with saturated NH_4Cl, diluted with water, and extracted with EtOAc (2×). The combined organic extracts were washed with brine, dried over anhydrous $MgSO_4$, filtered, and concentrated *in vacuo*. Flash chromatography on silica gel (0–50% Et_2O/hexanes, linear gradient) afforded 2.24 g (81%) of **S51** as a white foam. ^{1}H NMR ($CDCl_3$, 400 MHz) δ 8.14 (dd, J = 8.0, 1.3 Hz, 1H), 7.99 (dd, J = 8.0, 0.8 Hz, 1H), 7.85 (dd, J = 1.3, 0.8 Hz, 1H), 7.33 (d, J = 2.6 Hz, 2H), 7.00 (d, J = 8.8 Hz, 2H), 6.95 (dd, J = 8.9, 2.6 Hz, 2H), 5.23 (AB quartet, ν_A = 2092.3 Hz, ν_B = 2089.1 Hz, J_{AB} = 7.0 Hz, 4H), 3.79 – 3.70 (m, 4H), 1.56 (s, 9H), 0.97 – 0.89 (m, 4H), 0.70 (s, 3H), 0.61 (s, 3H), -0.02 (s, 18H); ^{13}C NMR ($CDCl_3$, 101 MHz) δ 170.0 (C), 164.3 (C), 157.0 (C), 155.0 (C), 137.5 (C), 137.2 (C), 136.9 (C), 130.3 (CH), 128.6 (C), 128.2 (CH), 126.0 (CH), 125.0 (CH), 121.4 (CH), 117.6 (CH), 93.0 (CH_2), 90.4 (C), 82.6 (C), 66.5 (CH_2), 28.2 (CH_3), 18.2 (CH_2), 0.0 (CH_3), -0.7 (CH_3), -1.3 (CH_3); HRMS (ESI) calcd for $C_{39}H_{55}O_8Si_3$ $[M+H]^+$ 735.3199, found 735.3188.

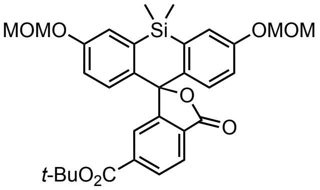

6-(*tert*-Butoxycarbonyl)-Si-fluorescein bis(methoxymethyl ether) (S52): To a solution of di-*tert*-butyl 2-iodoterephthalate (**S43**; 1.35 g, 3.35 mmol, 2 eq) in THF (8 mL) at -40 °C was added *i*-PrMgCl (2.0 M in THF; 1.67 mL, 3.35 mmol, 2 eq). The reaction was then gradually warmed to -25 °C over 2 h while stirring. A solution of 3,7-bis(methoxymethoxy)-5,5-dimethyldibenzo[*b*,*e*]silin-10(5*H*)-one (**S5**; 600 mg, 1.67 mmol) in THF (4 mL) was added dropwise; the reaction was then warmed to room temperature and stirred for 2 h. It was subsequently quenched with saturated NH_4Cl, diluted with water, and extracted with EtOAc (2×). The combined organic extracts were washed with brine, dried over anhydrous $MgSO_4$, filtered, and concentrated *in vacuo*. Flash chromatography on silica gel (0–70% Et_2O/hexanes, linear gradient) afforded 801 mg (85%) of **S52** as a white foam. ^{1}H NMR ($CDCl_3$, 400 MHz) δ 8.13 (dd, J = 8.0, 1.3 Hz, 1H), 7.98 (dd, J = 8.0, 0.7 Hz, 1H), 7.85 (dd, J = 1.3, 0.8 Hz, 1H), 7.32 (d, J = 2.6 Hz, 2H), 7.00 (d, J = 8.8 Hz, 2H), 6.94 (dd, J = 8.9, 2.7 Hz, 2H), 5.18 (AB quartet, ν_A = 2076.4 Hz, ν_B = 2069.2 Hz, J_{AB} = 6.9 Hz, 4H), 3.47 (s, 6H), 1.56 (s, 9H), 0.70 (s, 3H), 0.62 (s, 3H); ^{13}C NMR ($CDCl_3$, 101 MHz) δ 170.0 (C), 164.3 (C), 156.8 (C), 154.9 (C), 137.5 (C), 137.3 (C), 137.0 (C), 130.3 (CH), 128.6 (C), 128.3 (CH), 126.0 (CH), 125.0 (CH), 121.4 (CH), 117.5 (CH), 94.5 (CH_2), 90.4 (C), 82.6 (C), 56.3 (CH_3), 28.2 (CH_3), 0.0 (CH_3), -0.7 (CH_3); HRMS (ESI) calcd for $C_{31}H_{35}O_8Si$ $[M+H]^+$ 563.2096, found 563.2091.

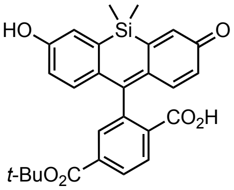

6-(*tert*-Butoxycarbonyl)-Si-fluorescein (S53): *Via bis-(trimethylsilylethoxymethyl ether) deprotection:* To a solution of 6-(*tert*-butoxycarbonyl)-Si-fluorescein bis(trimethylsilylethoxymethyl ether) (**S51**; 147 mg, 0.200 mmol) in 2:1 EtOAc/*t*-BuOH (3 mL) was added 4 M HCl in dioxane (1 mL), and the resulting red-pink solution was stirred at room temperature for 1 h. It was subsequently diluted with water and extracted with EtOAc (2×). The organic extracts were washed with brine, dried over anhydrous $MgSO_4$, filtered, and concentrated *in vacuo*. Silica gel chromatography (5–100% EtOAc/hexanes, linear gradient) afforded **S53** (90 mg, 95%) as an off-white solid.

Via bis-(methoxymethyl ether) deprotection: To a solution of 6-(*tert*-butoxycarbonyl)-Si-fluorescein bis(methoxymethyl ether) (**S52**; 270 mg, 0.480 mmol) in 2:1 EtOAc/*t*-BuOH (7.5 mL) was added 4 M HCl in dioxane (2.5 mL), and the resulting red-pink solution was stirred at room temperature for 2 h. It was subsequently diluted with water and extracted with EtOAc (2×). The organic extracts were washed with brine, dried over anhydrous $MgSO_4$, filtered, and concentrated *in vacuo*. Silica gel chromatography (5–100% EtOAc/hexanes, linear gradient) afforded **S53** (218 mg, 96%) as a white solid.

^{1}H NMR (DMSO-d_6, 400 MHz) δ 9.75 (s, 2H), 8.07 (AB of ABX, ν_A = 3233.3 Hz, J_{AX} = 1.3 Hz, ν_B = 3226.5 Hz, J_{BX} = 0.7 Hz, J_{AB} = 8.0 Hz, 2H), 7.59 (dd, J = 1.4, 0.7 Hz, 1H), 7.14 (d, J = 2.6 Hz, 2H), 6.82 (d, J = 8.8 Hz, 2H), 6.75 (dd, J = 8.8, 2.7 Hz, 2H), 1.49 (s, 9H), 0.62 (s, 3H), 0.52 (s, 3H); ^{13}C NMR (DMSO-d_6, 101 MHz) δ 169.3 (C), 163.4 (C), 156.9 (C), 155.6 (C), 136.9 (C), 135.5 (C), 133.7 (C), 129.9 (CH), 127.9 (CH), 127.2 (C), 126.2 (CH), 123.4 (CH), 120.1 (CH), 117.5 (CH), 89.8 (C), 82.2 (C), 27.6 (CH_3), -0.6 (CH3), -0.7 (CH_3); HRMS (ESI) calcd for $C_{27}H_{27}O_6Si$ $[M+H]^+$ 475.1571, found 475.1569.

HM-Si-Fluorescein Ditriflates S4 and 10

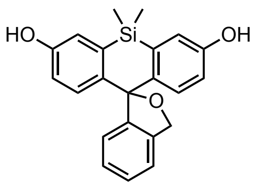

Hydroxymethyl-Si-fluorescein (HM-SiFl, S3): A solution of bis(2-bromo-5-(methoxymethoxy)phenyl)-dimethylsilane (**S1**; 2.00 g, 4.08 mmol) in THF (50 mL) was cooled to -78 °C under nitrogen. *tert*-Butyllithium (1.7 M in pentane, 10.56 mL, 17.95 mmol, 4.4 eq) was added, and the reaction was stirred at -78 °C for 30 min. It was then warmed to -20 °C, and a solution of phthalide (**S2**; 1.20 g, 8.97 mmol, 2.2 eq) in THF (50 mL) was added dropwise over 30 min via addition funnel. The reaction was then allowed to warm to room temperature overnight (18 h). It was subsequently diluted with saturated NH_4Cl and water and extracted with EtOAc (2×). The combined organic extracts were washed with brine, dried over anhydrous $MgSO_4$, filtered, and concentrated *in vacuo*. The crude residue was redissolved in 2:1 EtOAc/*t*-BuOH (45 mL). Following the addition of 4 M HCl in dioxane (15 mL), the resulting red solution was stirred at room temperature for 4 h. It was then diluted with water and extracted with EtOAc (2×). The organic extracts were washed with brine, dried over anhydrous $MgSO_4$, filtered, and evaporated. Silica gel chromatography (0–60% EtOAc/hexanes, linear gradient) afforded **S3** (1.16 g, 79%) as a pale yellow-orange solid. ^{1}H NMR (CD_3OD, 400 MHz) δ 7.35 (dt, J = 7.6, 1.0 Hz, 1H), 7.28 (td, J = 7.4, 1.1 Hz, 1H), 7.21 (td, J = 7.4, 1.2 Hz, 1H), 7.04 (d, J = 2.7 Hz, 2H), 7.00 (d, J = 8.7 Hz, 2H), 6.88 (dt, J = 7.6, 1.0 Hz, 1H), 6.68 (dd, J = 8.7, 2.8 Hz, 2H), 5.32 (s, 2H), 0.56 (s, 3H), 0.47 (s, 3H); ^{13}C NMR (CD_3OD, 101 MHz) δ 157.0 (C), 148.2 (C), 142.8 (C), 139.4 (C), 136.5 (C), 130.5 (CH), 128.53 (CH), 128.45 (CH), 124.8 (CH), 122.4 (CH), 120.1 (CH), 117.7 (CH), 94.0 (C), 73.9 (CH_2), 0.2 (CH_3), -0.6 (CH_3); HRMS (ESI) calcd for $C_{22}H_{21}O_3Si$ $[M+H]^+$ 361.1254, found 361.1247.

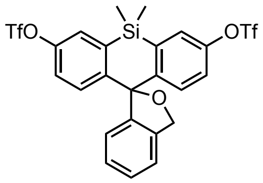

Hydroxymethyl-Si-fluorescein ditriflate (Tf_2-HM-SiFl, S4): Hydroxymethyl-Si-fluorescein (**S3**; 1.10 g, 3.05 mmol) was taken up in DMF (30 mL). Na_2CO_3 (1.94 g, 18.31 mmol, 6 eq) and *N*-phenyl-bis(trifluoromethanesulfonimide) (3.27 g, 9.15 mmol, 3 eq) were added in succession, and the reaction was stirred at room temperature for 18 h. It was then diluted with water and extracted with EtOAc (2×). The combined organic extracts were washed with water and brine, dried over anhydrous $MgSO_4$, filtered, and concentrated *in vacuo*. Flash chromatography (0–30% Et_2O/hexanes, linear gradient) yielded 1.77 g (93%) of **S4** as a white foam. ^{1}H NMR ($CDCl_3$, 400 MHz) δ 7.49 (d, J = 2.7 Hz, 2H), 7.41 – 7.30 (m, 3H), 7.35 (d, J = 8.9 Hz, 2H), 7.16 (dd, J = 8.9, 2.8 Hz, 2H), 7.09 (dt, J = 7.5, 1.0 Hz, 1H), 5.33 (s, 2H), 0.70 (s, 3H), 0.61 (s, 3H); ^{19}F NMR ($CDCl_3$, 376 MHz) δ -73.35 (s); ^{13}C NMR ($CDCl_3$, 101 MHz) δ 150.0 (C), 148.8 (C), 143.5 (C), 139.0 (C), 137.9 (C), 129.7 (CH), 128.6 (CH), 128.1

(CH), 125.8 (CH), 124.4 (CH), 122.4 (CH), 122.0 (CH), 118.9 (q, $^{1}J_{CF}$ = 320.8 Hz, CF_3), 91.9 (C), 73.6 (CH_2), 0.3 (CH_3), -1.3 (CH_3); HRMS (ESI) calcd for $C_{24}H_{19}F_6O_7S_2Si$ $[M+H]^+$ 625.0240, found 625.0232.

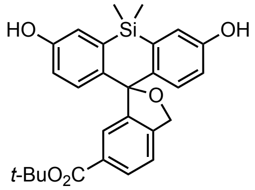

6-*tert*-Butoxycarbonyl-hydroxymethyl-Si-fluorescein (HM-SiFl-CO$_2$*t*-Bu, S7): To a solution of *tert*-butyl 3-iodo-4-(((triisopropylsilyl)oxy)methyl)benzoate (**S6**; 4.98 g, 10.15 mmol, 1.5 eq) in THF (25 mL) at -40 °C was added *i*-PrMgCl (2.0 M in THF; 5.08 mL, 10.15 mmol, 1.5 eq). The reaction was then gradually warmed to -25 °C over 2 h while stirring. A solution of 3,7-bis(methoxymethoxy)-5,5-dimethyldibenzo[*b*,*e*]silin-10(5*H*)-one (**S5**; 2.43 g, 6.77 mmol) in THF (10 mL) was added dropwise; the reaction was then warmed to room temperature and stirred for 2 h. It was subsequently quenched with saturated NH_4Cl, diluted with water, and extracted with EtOAc (2×). The combined organic extracts were washed with brine, dried over anhydrous $MgSO_4$, filtered, and concentrated *in vacuo*. The crude residue was redissolved in 2:1 EtOAc/*t*-BuOH (75 mL). Following the addition of 4 M HCl in dioxane (25 mL), the resulting red solution was stirred at room temperature for 6 h. It was then diluted with water and extracted with EtOAc (2×). The organic extracts were washed with brine, dried over anhydrous $MgSO_4$, filtered, and evaporated. Silica gel chromatography (10–75% EtOAc/hexanes, linear gradient) afforded **S7** (2.70 g, 87%) as a pale orange foam. ^{1}H NMR (DMSO-d_6, 400 MHz) δ 9.46 (s, 2H), 7.75 (dd, J = 7.9, 1.5 Hz, 1H), 7.49 (d, J = 8.0 Hz, 1H), 7.24 (d, J = 1.4 Hz, 1H), 7.22 (d, J = 8.8 Hz, 2H), 7.01 (d, J = 2.7 Hz, 2H), 6.74 (dd, J = 8.8, 2.7 Hz, 2H), 5.62 (s, 2H), 1.43 (s, 9H), 0.61 (s, 3H), 0.45 (s, 3H); ^{13}C NMR ($CDCl_3$, 101 MHz) δ 164.3 (C), 155.8 (C), 149.1 (C), 141.9 (C), 141.3 (C), 132.8 (C), 130.8 (C), 128.7 (CH), 128.2 (CH), 122.7 (CH), 122.3 (CH), 119.0 (CH), 117.5 (CH), 91.4 (C), 81.0 (C), 74.0 (CH_2), 27.7 (CH_3), 0.7 (CH_3), -0.6 (CH_3); HRMS (ESI) calcd for $C_{27}H_{29}O_5Si$ $[M+H]^+$ 461.1779, found 461.1779.

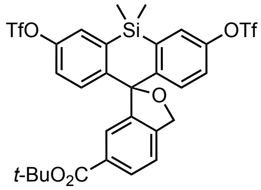

6-*tert*-Butoxycarbonyl-hydroxymethyl-Si-fluorescein ditriflate (Tf$_2$-HM-SiFl-CO$_2$*t*-Bu, 10): 6-*tert*-Butoxycarbonyl-hydroxymethyl-Si-fluorescein (**S7**; 2.75 g, 5.97 mmol) was taken up in DMF (60 mL). Na_2CO_3 (5.06 g, 47.76 mmol, 8 eq) and *N*-phenyl-bis(trifluoromethanesulfonimide) (8.53 g, 23.88 mmol, 4 eq) were added in succession, and the reaction was stirred at room temperature for 18 h. The resulting off-white suspension was diluted with water and extracted with EtOAc (2×). The combined organic extracts were washed with water and brine, dried over anhydrous $MgSO_4$, filtered, and concentrated *in vacuo*. Flash chromatography (0–20% EtOAc/hexanes, linear gradient) yielded 4.05 g (94%) of **10** as a white foam. ^{1}H NMR ($CDCl_3$, 400 MHz) δ 7.99 (dd, J = 8.0, 1.4 Hz, 1H), 7.65 (d, J = 1.4 Hz, 1H), 7.50 (d, J = 2.7 Hz, 2H), 7.44 (d, J = 8.9 Hz, 2H), 7.39 (d, J = 8.0 Hz, 1H), 7.20 (dd, J = 8.9,

2.7 Hz, 2H), 5.49 (s, 2H), 1.55 (s, 9H), 0.78 (s, 3H), 0.61 (s, 3H); ^{19}F NMR ($CDCl_3$, 376 MHz) δ -73.32 (s); ^{13}C NMR ($CDCl_3$, 101 MHz) δ 165.0 (C), 150.1 (C), 148.9 (C), 144.8 (C), 142.4 (C), 137.0 (C), 132.6 (C), 130.1 (CH), 129.5 (CH), 126.0 (CH), 124.8 (CH), 122.8 (CH), 121.9 (CH), 118.9 (q, $^{1}J_{CF}$ = 320.9 Hz, CF_3), 91.8 (C), 81.8 (C), 74.1 (CH_2), 28.3 (CH_3), 0.0 (CH_3), -0.5 (CH_3); HRMS (ESI) calcd for $C_{29}H_{27}F_6O_9S_2Si$ $[M+H]^+$ 725.0764, found 725.0755.

References

(1) Egawa, T.; Koide, Y.; Hanaoka, K.; Komatsu, T.; Terai, T.; Nagano, T. Development of a fluorescein analogue, TokyoMagenta, as a novel scaffold for fluorescence probes in red region. *Chem. Commun.* **2011**, *47*, 4162-4164.

(2) Grimm, J. B.; Brown, T. A.; Tkachuk, A. N.; Lavis, L. D. General synthetic method for Si-fluoresceins and Si-rhodamines. *ACS Cent. Sci.* **2017**, *3*, 975-985.

(3) Grimm, J. B.; Sung, A. J.; Legant, W. R.; Hulamm, P.; Matlosz, S. M.; Betzig, E.; Lavis, L. D. Carbofluoresceins and carborhodamines as scaffolds for high-contrast fluorogenic probes. *ACS Chem. Biol.* **2013**, *8*, 1303-1310.

(4) Grimm, J. B.; Muthusamy, A. K.; Liang, Y.; Brown, T. A.; Lemon, W. C.; Patel, R.; Lu, R.; Macklin, J. J.; Keller, P. J.; Ji, N.; Lavis, L. D. A general method to fine-tune fluorophores for live-cell and in vivo imaging. *Nat. Methods* **2017**, *14*, 987-994.

(5) Grimm, J. B.; Gruber, T. D.; Ortiz, G.; Brown, T. A.; Lavis, L. D. Virginia Orange: A versatile, red-shifted fluorescein scaffold for single-and dual-input fluorogenic probes. *Bioconjugate Chem.* **2016**, *27*, 474–480.

(6) Martineau, M.; Somasundaram, A.; Grimm, J. B.; Gruber, T. D.; Choquet, D.; Taraska, J. W.; Lavis, L. D.; Perrais, D. Semisynthetic fluorescent pH sensors for imaging exocytosis and endocytosis. *Nat. Commun.* **2017**, *8*, 1412.

(7) Grimm, J. B.; English, B. P.; Chen, J.; Slaughter, J. P.; Zhang, Z.; Revyakin, A.; Patel, R.; Macklin, J. J.; Normanno, D.; Singer, R. H.; Lionnet, T.; Lavis, L. D. A general method to improve fluorophores for live-cell and single-molecule microscopy. *Nat. Methods* **2015**, *12*, 244–250.

(8) Uno, S.-n.; Kamiya, M.; Yoshihara, T.; Sugawara, K.; Okabe, K.; Tarhan, M. C.; Fujita, H.; Funatsu, T.; Okada, Y.; Tobita, S.; Urano, Y. A spontaneously blinking fluorophore based on intramolecular spirocyclization for live-cell super-resolution imaging. *Nat. Chem.* **2014**, *6*, 681-689.

(9) Iwatate, R. J.; Kamiya, M.; Umezawa, K.; Kashima, H.; Nakadate, M.; Kojima, R.; Urano, Y. Silicon rhodamine-based near-infrared fluorescent probe for γ-glutamyltransferase. *Bioconjugate Chem.* **2018**, *29*, 241-244.

(10) Bucevičius, J.; Gilat, T.; Lukinavičius, G. Far-red switching DNA probes for live cell nanoscopy. *Chem. Commun.* **2020**, *56*, 14797-14800.

(11) Chi, W.; Qiao, Q.; Wang, C.; Zheng, J.; Zhou, W.; Xu, N.; Wu, X.; Jiang, X.; Tan, D.; Xu, Z.; Liu, X. Descriptor ΔG(C-O) enables the quantitative design of spontaneously blinking rhodamines for live-cell super-resolution imaging. *Angew. Chem. Int. Edit.* **2020**, *59*, 20215-20223.

(12) Zheng, Q.; Ayala, A. X.; Chung, I.; Weigel, A. V.; Ranjan, A.; Falco, N.; Grimm, J. B.; Tkachuk, A. N.; Wu, C.; Lippincott-Schwartz, J.; Singer, R. H.; Lavis, L. D. Rational design of fluorogenic and spontaneously blinking labels for super-resolution imaging. *ACS Cent. Sci.* **2019**, *5*, 1602-1613.

(13) Best, Q. A.; Sattenapally, N.; Dyer, D. J.; Scott, C. N.; McCarroll, M. E. pH-Dependent Si-fluorescein hypochlorous acid fluorescent probe: spirocycle ring-opening and excess hypochlorous acid-induced chlorination. *J. Am. Chem. Soc* **2013**, *135*, 13365-13370.

(14) Butkevich, A. N.; Belov, V. N.; Kolmakov, K.; Sokolov, V. V.; Shojaei, H.; Sidenstein, S. C.; Kamin, D.; Matthias, J.; Vlijm, R.; Engelhardt, J.; Hell, S. W. Hydroxylated fluorescent dyes for live-cell labeling: synthesis, spectra and super-resolution STED. *Chem. Eur. J.* **2017**, *23*, 12114-12119.

(15) Smaga, L. P.; Pino, N. W.; Ibarra, G. E.; Krishnamurthy, V.; Chan, J. A photoactivatable formaldehyde donor with fluorescence monitoring reveals threshold to arrest cell migration. *J. Am. Chem. Soc* **2020**, *142*, 680-684.

(16) Bearrood, T. E.; Aguirre-Figueroa, G.; Chan, J. Rational design of a red fluorescent sensor for ALDH1A1 displaying enhanced cellular uptake and reactivity. *Bioconjugate Chem.* **2020**, *31*, 224-228.

(17) Minta, A.; Kao, J. P.; Tsien, R. Y. Fluorescent indicators for cytosolic calcium based on rhodamine and fluorescein chromophores. *J. Biol. Chem.* **1989**, *264*, 8171–8178.

(18) Butkevich, A. N.; Mitronova, G. Y.; Sidenstein, S. C.; Klocke, J. L.; Kamin, D.; Meineke, D. N.; D'Este, E.; Kraemer, P. T.; Danzl, J. G.; Belov, V. N.; Hell, S. W. Fluorescent rhodamines and fluorogenic carbopyronines for super-resolution STED microscopy in living cells. *Angew. Chem. Int. Edit.* **2016**, *55*, 3290-3294.

(19) Bucevičius, J.; Kostiuk, G.; Gerasimaitė, R.; Gilat, T.; Lukinavičius, G. Enhancing the biocompatibility of rhodamine fluorescent probes by a neighbouring group effect. *Chem. Sci.* **2020**, *11*, 7313-7323.

(20) Kolmakov, K.; Belov, V. N.; Wurm, C. A.; Harke, B.; Leutenegger, M.; Eggeling, C.; Hell, S. W. A versatile route to red-emitting carbopyronine dyes for optical microscopy and nanoscopy. *Eur. J. Org. Chem.* **2010**, *2010*, 3593-3610.

(21) Lukinavičius, G.; Umezawa, K.; Olivier, N.; Honigmann, A.; Yang, G.; Plass, T.; Mueller, V.; Reymond, L.; Corrêa Jr, I. R.; Luo, Z. G.; Schultz, C.; Lemke, E. A.; Heppenstall, P.; Eggeling, C.; Manley, S.; Johnsson, K. A near-infrared fluorophore for live-cell super-resolution microscopy of cellular proteins. *Nat. Chem.* **2013**, *5*, 132–139.

(22) Grimm, J. B.; Klein, T.; Kopek, B. G.; Shtengel, G.; Hess, H. F.; Sauer, M.; Lavis, L. D. Synthesis of a far-red photoactivatable silicon-containing rhodamine for super-resolution microscopy. *Angew. Chem. Int. Edit.* **2016**, *55*, 1723-1727.

(23) Butkevich, A. N.; Ta, H.; Ratz, M.; Stoldt, S.; Jakobs, S.; Belov, V. N.; Hell, S. W. Two-color 810 nm STED nanoscopy of living cells with endogenous SNAP-tagged fusion proteins. *ACS Chem. Biol.* **2018**, *13*, 475-480.

(24) Krasovskiy, A.; Knochel, P. A LiCl-mediated Br/Mg exchange reaction for the preparation of functionalized aryl- and heteroarylmagnesium compounds from organic bromides. *Angew. Chem. Int. Edit.* **2004**, *43*, 3333-3336.

(25) Boymond, L.; Rottländer, M.; Cahiez, G.; Knochel, P. Preparation of highly functionalized Grignard reagents by an iodine-magnesium exchange reaction and its application in solid-phase synthesis. *Angew. Chem. Int. Edit.* **1998**, *37*, 1701-1703.

(26) Abarbri, M.; Thibonnet, J.; Bérillon, L.; Dehmel, F.; Rottländer, M.; Knochel, P. Preparation of new polyfunctional magnesiated heterocycles using a chlorine-, bromine-, or iodine-magnesium exchange. *J. Org. Chem.* **2000**, *65*, 4618-4634.

(27) Knochel, P.; Dohle, W.; Gommermann, N.; Kneisel, F. F.; Kopp, F.; Korn, T.; Sapountzis, I.; Vu, V. A. Highly functionalized organomagnesium reagents prepared through halogen-metal exchange. *Angew. Chem. Int. Edit.* **2003**, *42*, 4302-4320.

(28) Hari, Y.; Kondo, R.; Date, K.; Aoyama, T. Facile synthesis of 2-unsubstituted benzofuran-3-carboxylates using diazo(trimethylsilyl)methylmagnesium bromide. *Tetrahedron* **2009**, *65*, 8708-8713.

(29) Cheng, H. G.; Zhang, R. M.; Wang, M.; Zeng, X. F.; Xie, C. S. Convergent assembly of enantioenriched tetrahydrobenzofuro[2,3-b]pyrrole scaffolds by Ag-catalyzed asymmetric domino reaction of isocyanoacetates. *Asian J Org Chem* **2018**, *7*, 1075-1079.

(30) Kuribayashi, T.; Kubota, H.; Fukuda, T.; Takano, R.; Tsuji, T.; Sasaki, K.; Tanaka, N. 5-Hydroxypyrimidine-4-carboxamide compound. US 2011/0112103 A1, May 12, 2011.

(31) Mathias, L. J. Esterification and alkylation reactions employing isoureas. *Synthesis* **1979**, 561-576.

(32) Widmer, U. A convenient preparation of t-butyl esters. *Synthesis* **1983**, *1983*, 135-136.

(33) Klapars, A.; Buchwald, S. L. Copper-catalyzed halogen exchange in aryl halides: an aromatic Finkelstein reaction. *J. Am. Chem. Soc.* **2002**, *124*, 14844-14845.

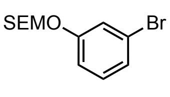

S35

Origin	Bruker BioSpin GmbH
Solvent	CDCl3
Temperature	300.0
Pulse Sequence	zg30
Experiment	1D
Number of Scans	16
Acquisition Date	2023-04-21T15:56:44
Spectrometer Frequency	400.13
Spectral Width	8012.8
Lowest Frequency	-1545.4
Nucleus	1H
Acquired Size	32768
Spectral Size	65536

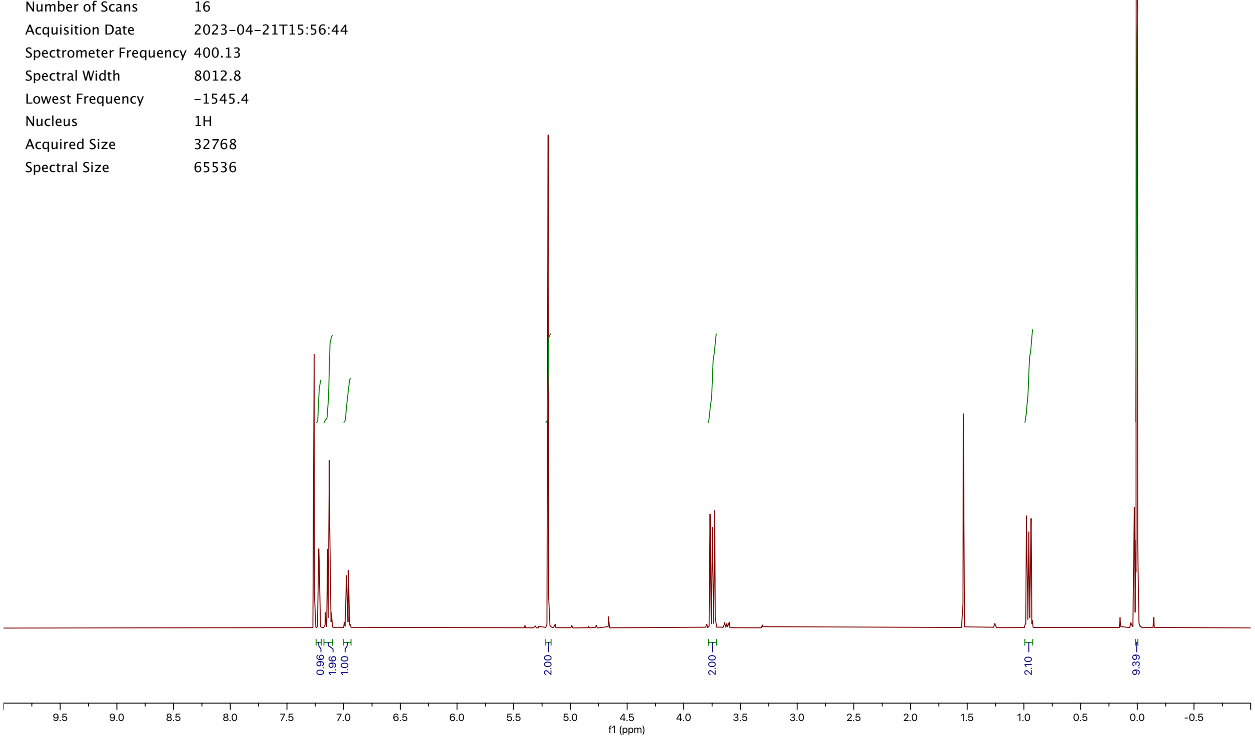

Origin	Bruker BioSpin GmbH
Solvent	CDCl3
Temperature	298.0
Pulse Sequence	zgpg30
Experiment	1D
Number of Scans	1024
Acquisition Date	2023-11-30T17:57:28
Spectrometer Frequency	100.62
Spectral Width	23809.5
Lowest Frequency	-1830.4
Nucleus	13C
Acquired Size	32768
Spectral Size	65536

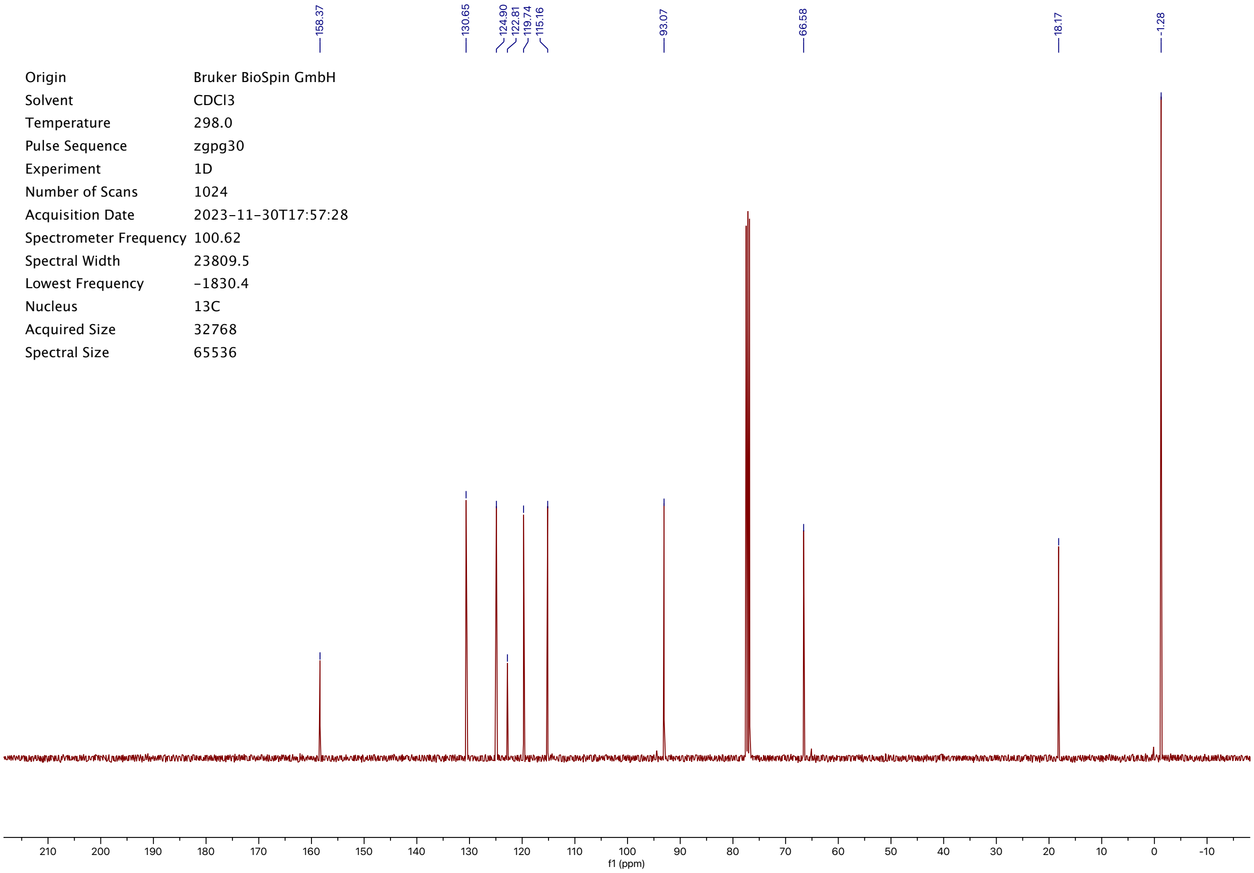

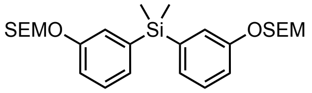

S37

Origin	Bruker BioSpin GmbH
Solvent	CDCl3
Temperature	298.0
Pulse Sequence	zg30
Experiment	1D
Number of Scans	16
Acquisition Date	2023-12-01T15:05:28
Spectrometer Frequency	400.13
Spectral Width	8196.7
Lowest Frequency	-1637.5
Nucleus	1H
Acquired Size	32768
Spectral Size	65536

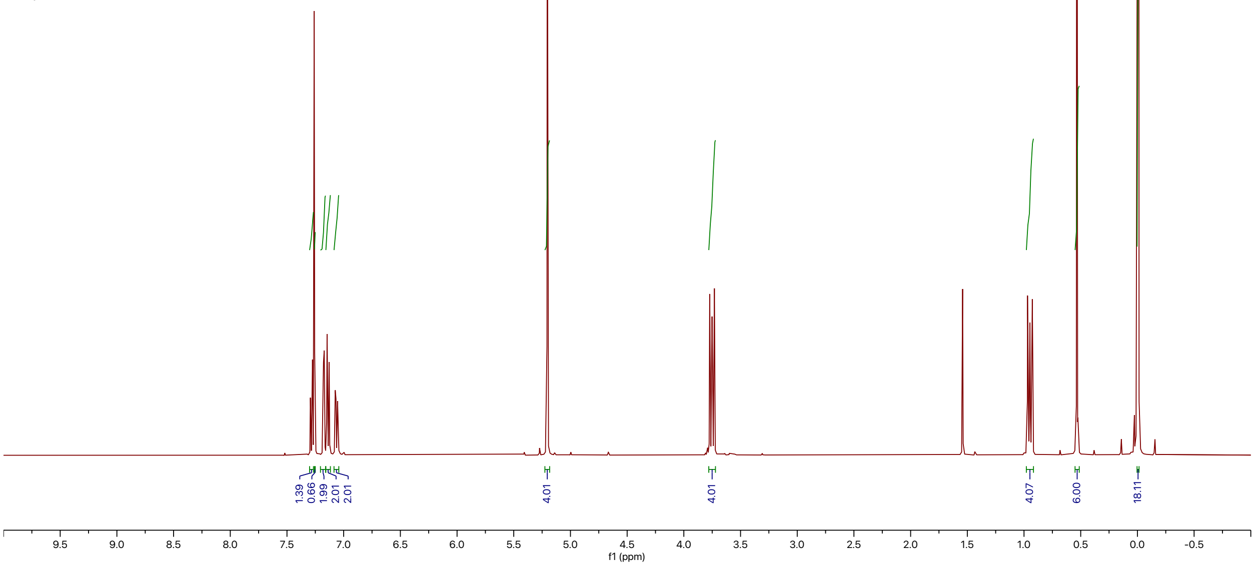

Origin	Bruker BioSpin GmbH
Solvent	CDCl3
Temperature	298.0
Pulse Sequence	zgpg30
Experiment	1D
Number of Scans	512
Acquisition Date	2023-12-01T16:18:25
Spectrometer Frequency	100.62
Spectral Width	23809.5
Lowest Frequency	-1831.1
Nucleus	13C
Acquired Size	32768
Spectral Size	65536

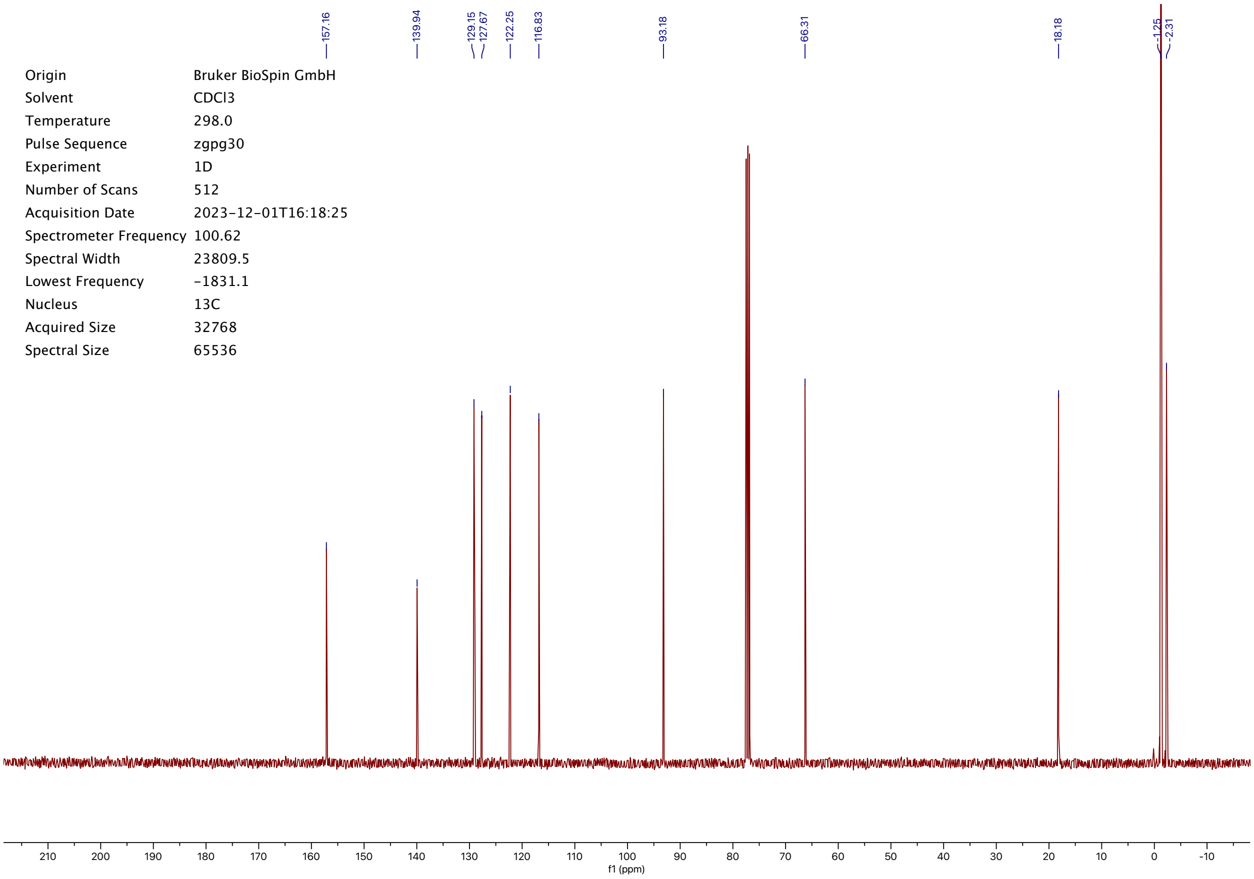

Origin	Bruker BioSpin GmbH
Solvent	CDCl3
Temperature	298.0
Pulse Sequence	zg30
Experiment	1D
Number of Scans	16
Acquisition Date	2023-12-01T15:12:16
Spectrometer Frequency	400.13
Spectral Width	8196.7
Lowest Frequency	-1637.5
Nucleus	1H
Acquired Size	32768
Spectral Size	65536

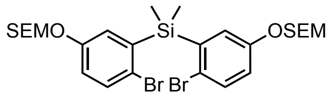

S39

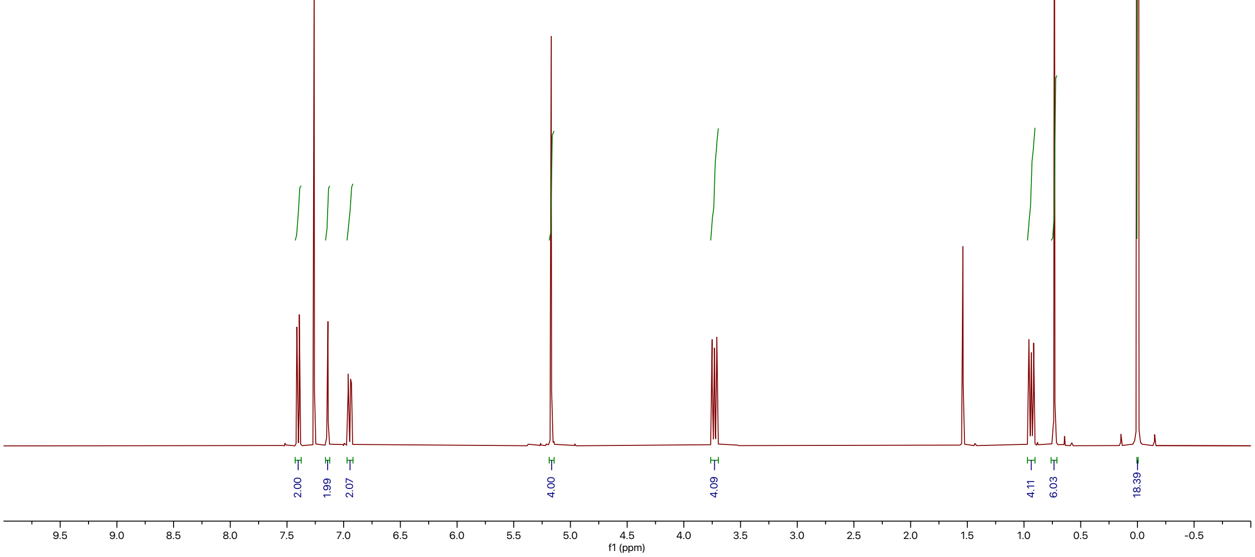

Origin	Bruker BioSpin GmbH
Solvent	CDCl3
Temperature	298.0
Pulse Sequence	zgpg30
Experiment	1D
Number of Scans	512
Acquisition Date	2023-12-01T18:15:35
Spectrometer Frequency	100.62
Spectral Width	23809.5
Lowest Frequency	-1830.9
Nucleus	13C
Acquired Size	32768
Spectral Size	65536

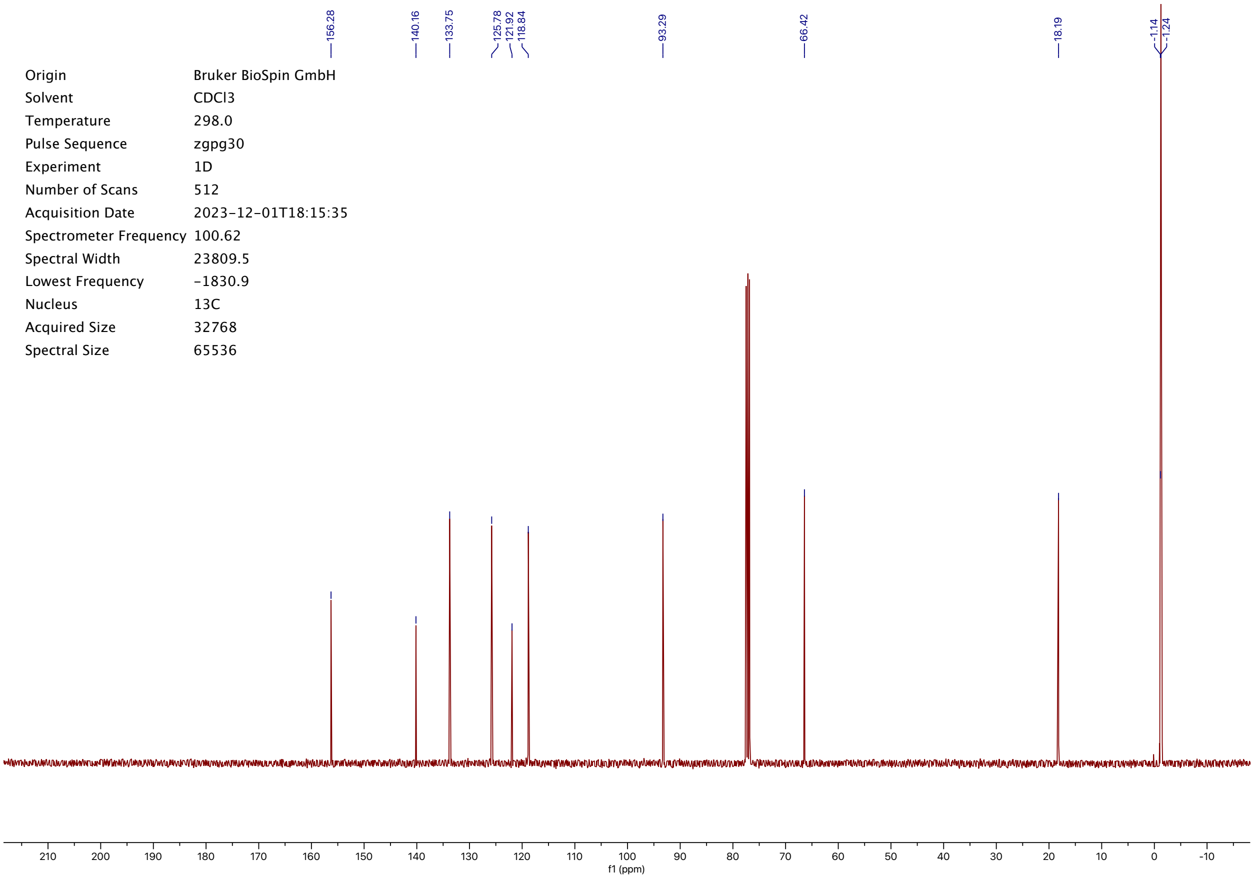

Origin	Bruker BioSpin GmbH
Solvent	CDCl3
Temperature	298.0
Pulse Sequence	zg30
Experiment	1D
Number of Scans	16
Acquisition Date	2023-12-01T15:19:13
Spectrometer Frequency	400.13
Spectral Width	8196.7
Lowest Frequency	-1639.1
Nucleus	1H
Acquired Size	32768
Spectral Size	65536

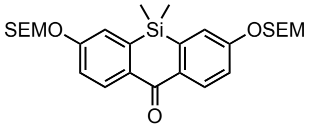

S40

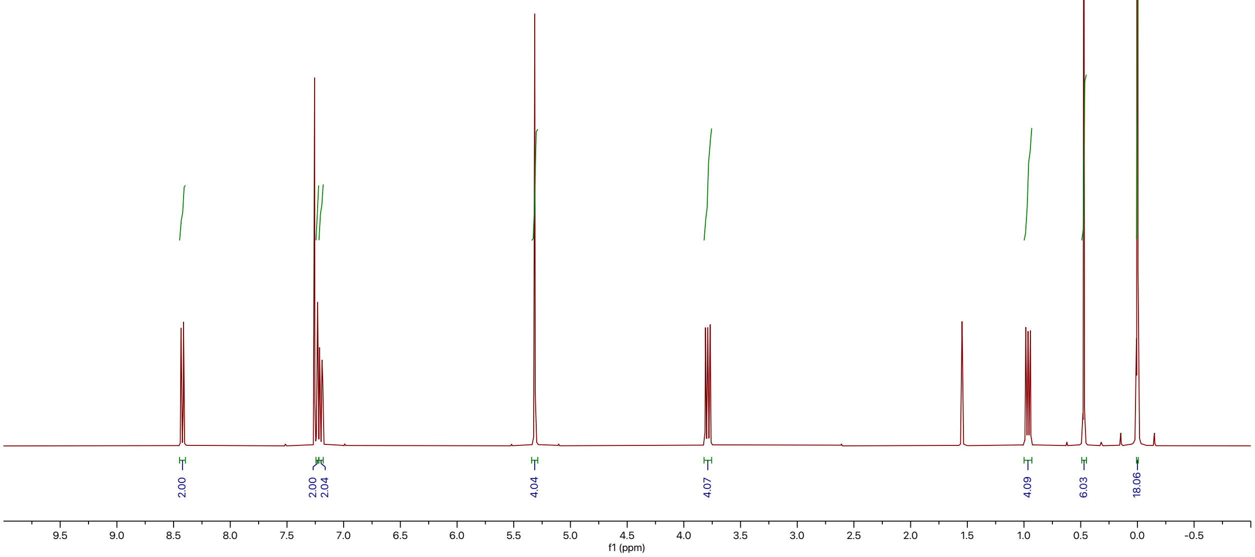

Origin	Bruker BioSpin GmbH
Solvent	CDCl3
Temperature	298.0
Pulse Sequence	zgpg30
Experiment	1D
Number of Scans	512
Acquisition Date	2023-12-01T20:12:50
Spectrometer Frequency	100.62
Spectral Width	23809.5
Lowest Frequency	-1831.2
Nucleus	13C
Acquired Size	32768
Spectral Size	65536

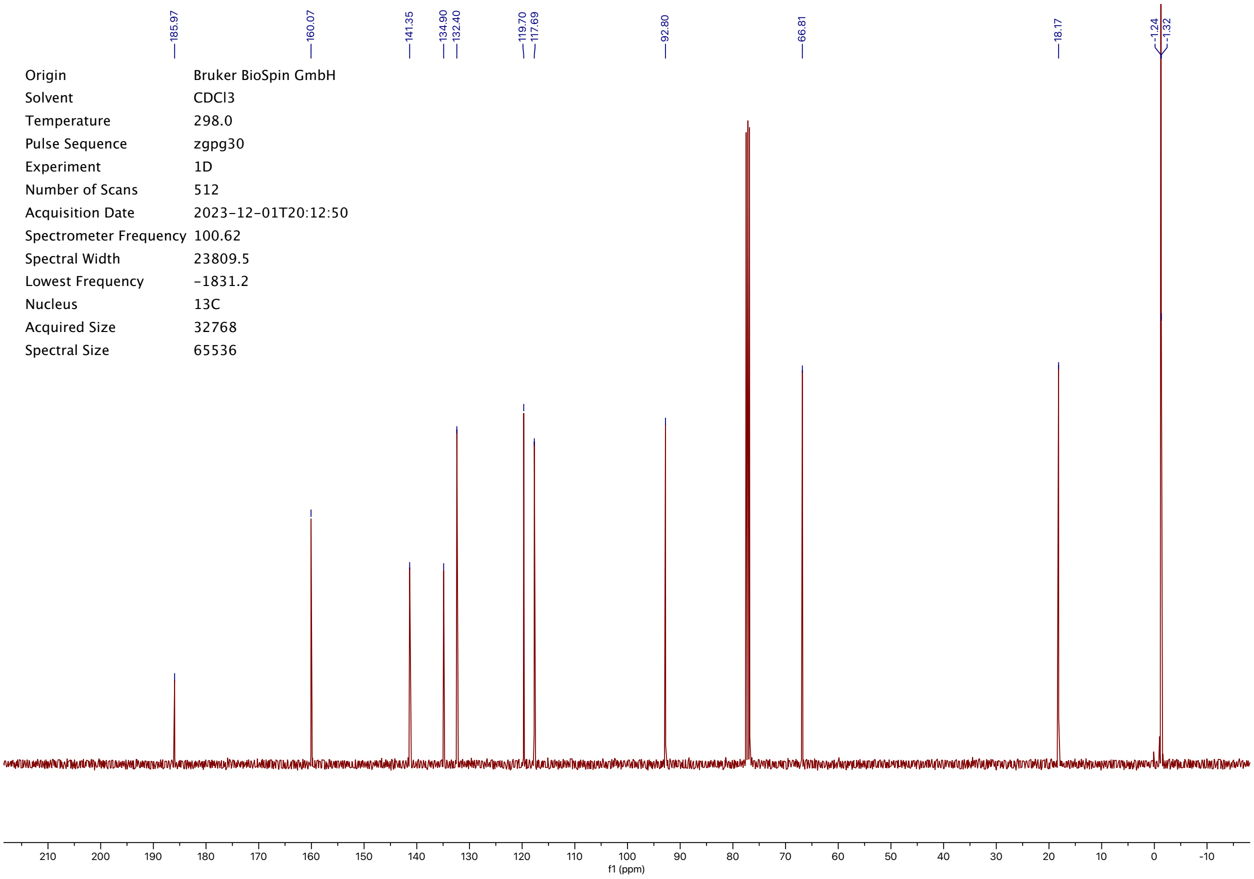

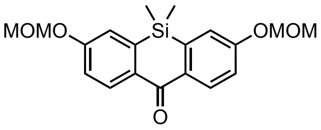

S5

Origin	Bruker BioSpin GmbH
Solvent	CDCl3
Temperature	295.1
Pulse Sequence	zg30
Experiment	1D
Number of Scans	16
Acquisition Date	2023-09-08T15:21:27
Spectrometer Frequency	400.13
Spectral Width	8012.8
Lowest Frequency	-1544.8
Nucleus	1H
Acquired Size	32768
Spectral Size	65536

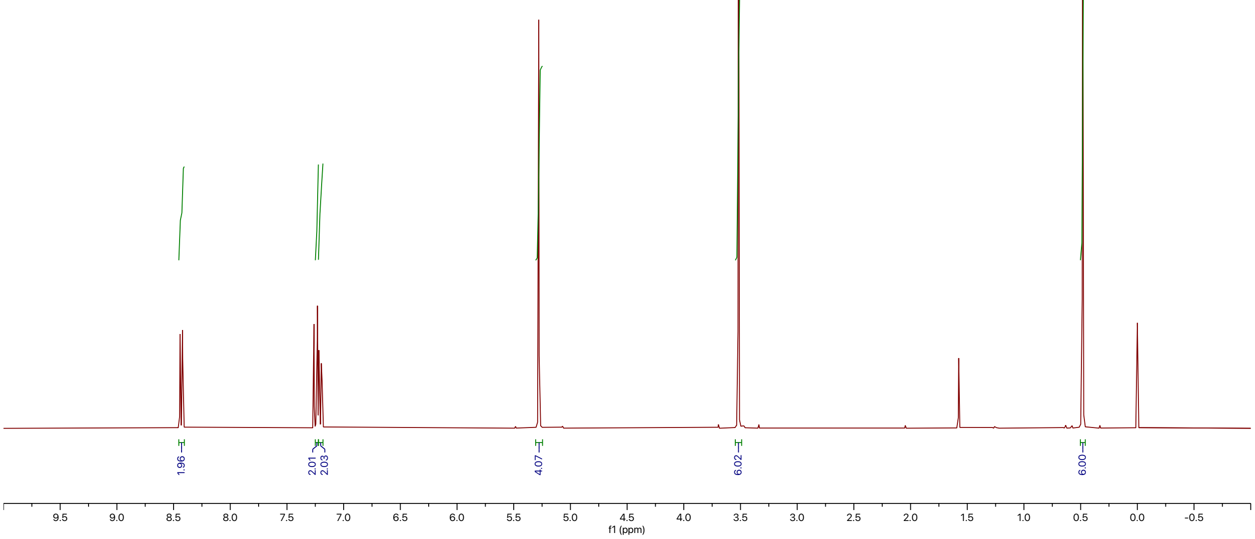

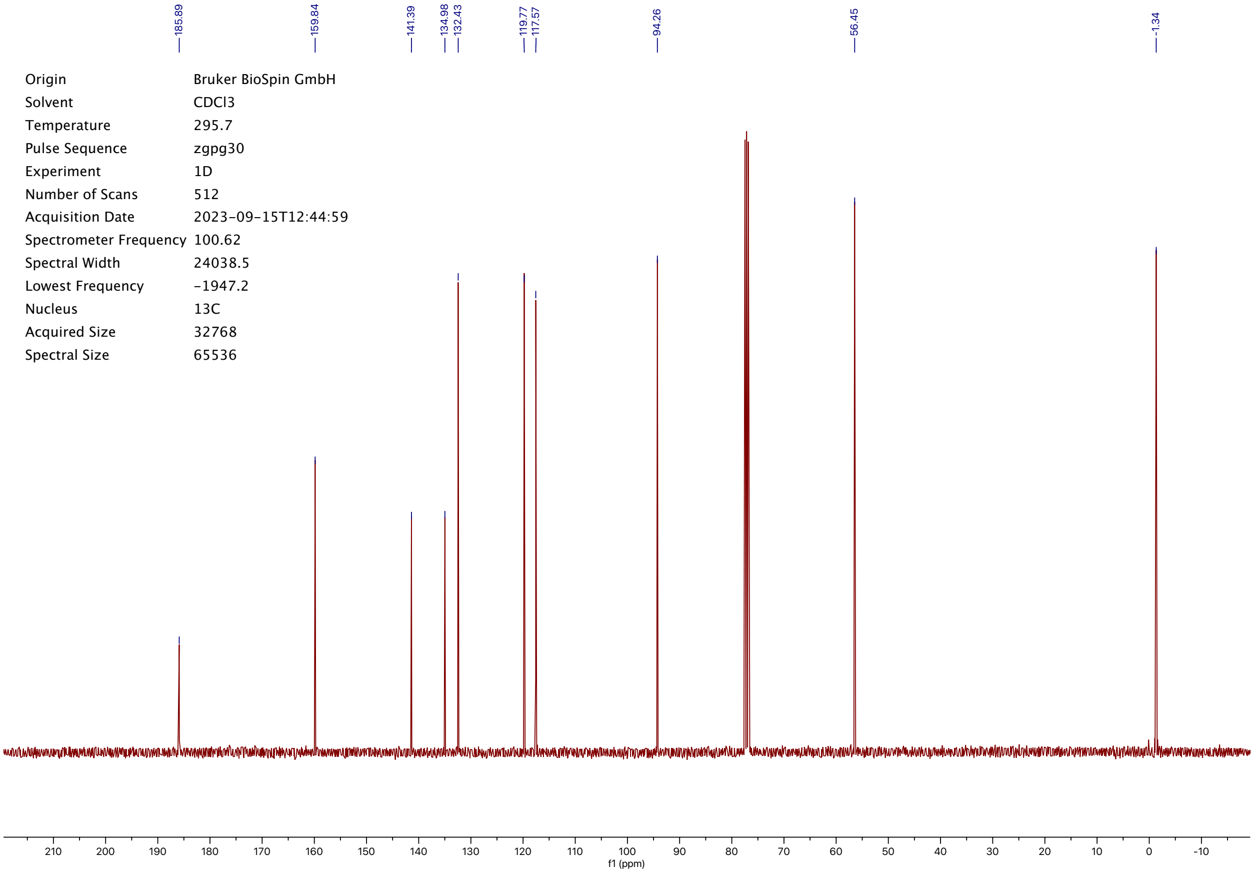

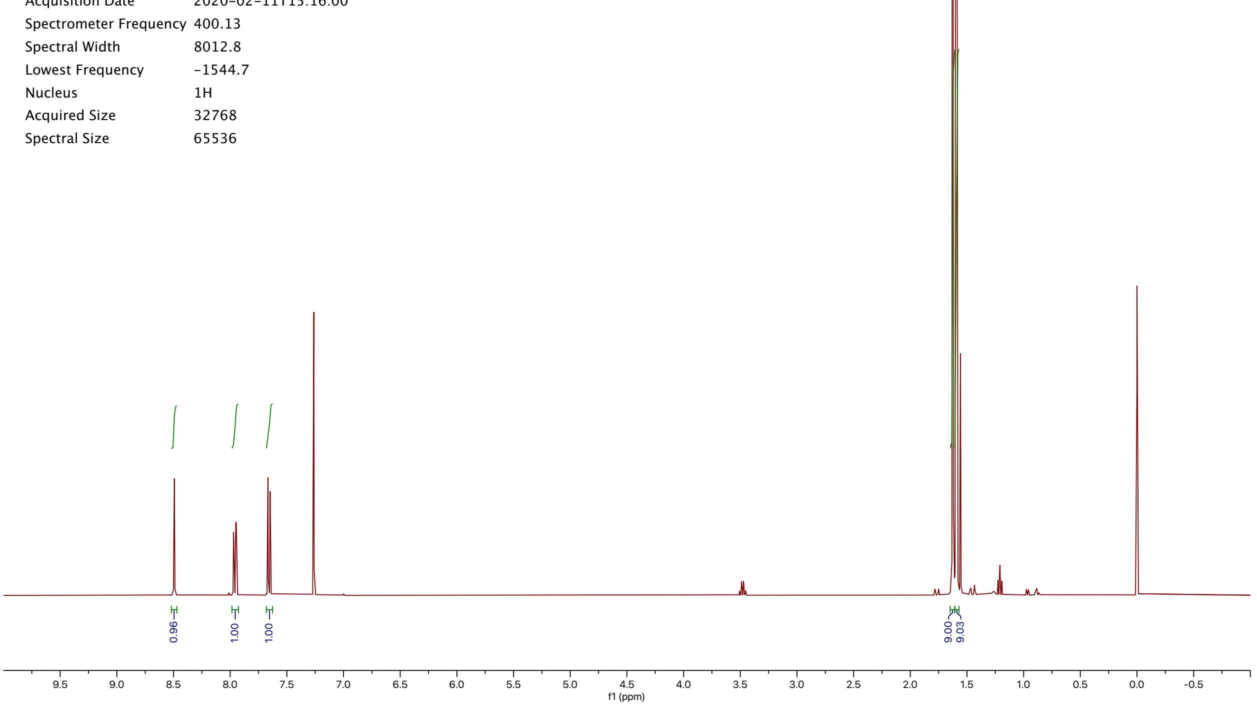
Origin Bruker BioSpin GmbH
Solvent CDCl3
Temperature 295.5
Pulse Sequence zg30
Experiment 1D
Number of Scans 16
Acquisition Date 2020-02-11T15:16:00
Spectrometer Frequency 400.13
Spectral Width 8012.8
Lowest Frequency -1544.7
Nucleus 1H
Acquired Size 32768
Spectral Size 65536
0.96
1.00
1.00
9.00
9.03
f1 (ppm)

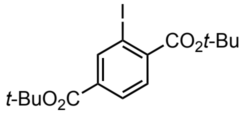
CO2t-Bu
t-BuO2C
I

S43

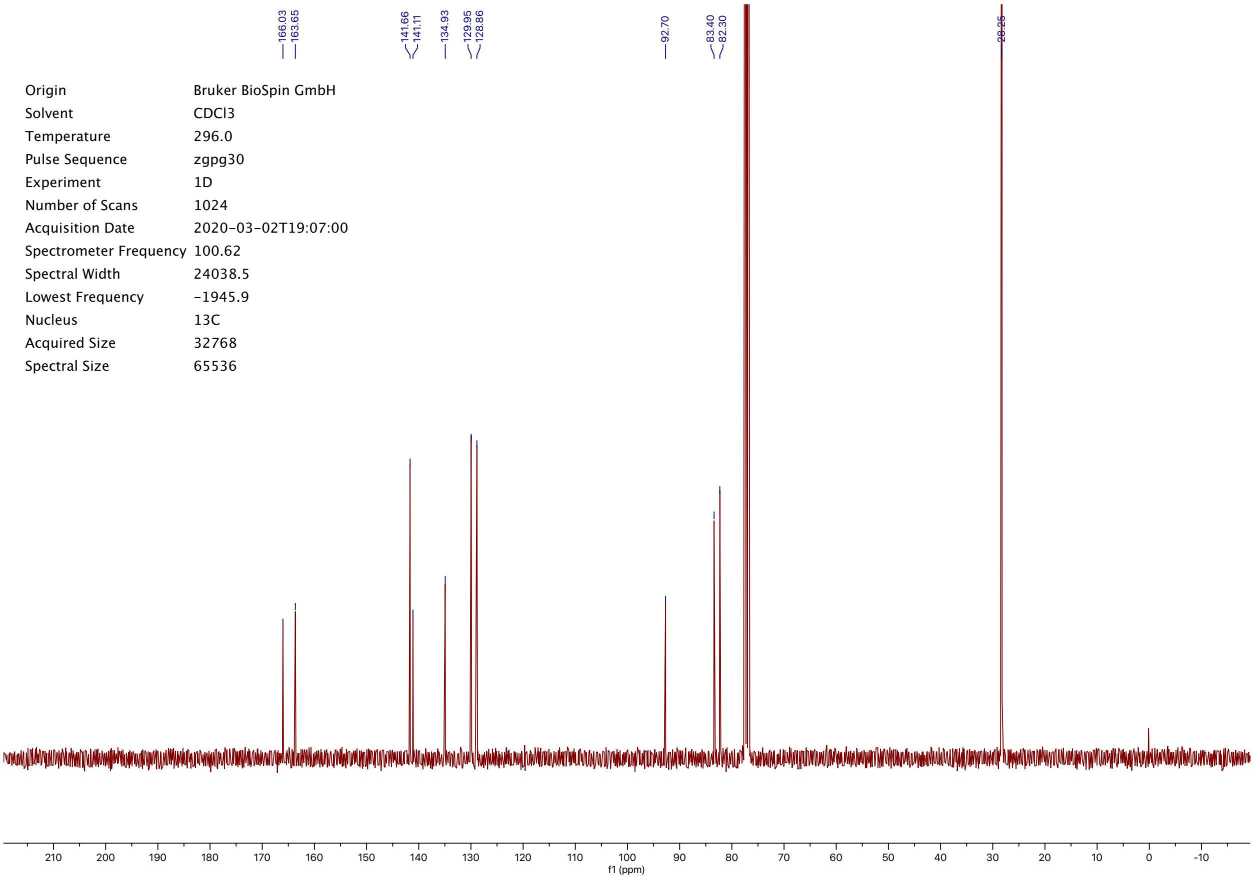
166.03
163.65
141.66
141.11
134.93
129.95
128.86
92.70
83.40
82.30
28.25
Origin Bruker BioSpin GmbH
Solvent CDCl3
Temperature 296.0
Pulse Sequence zgpg30
Experiment 1D
Number of Scans 1024
Acquisition Date 2020-03-02T19:07:00
Spectrometer Frequency 100.62
Spectral Width 24038.5
Lowest Frequency -1945.9
Nucleus 13C
Acquired Size 32768
Spectral Size 65536
f1 (ppm)

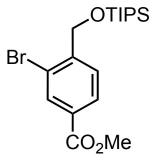
OTIPS
Br
CO_2Me

S55

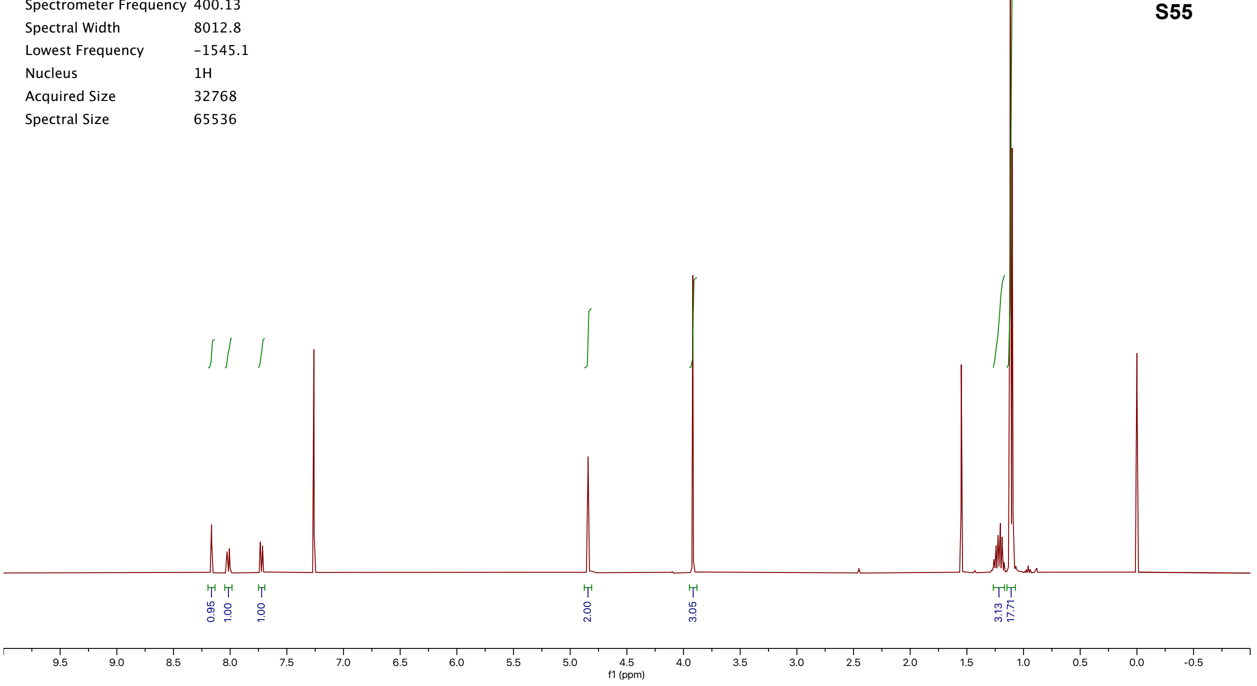
Origin Bruker BioSpin GmbH
Solvent CDCl3
Temperature 295.7
Pulse Sequence zg30
Experiment 1D
Number of Scans 16
Acquisition Date 2023-05-31T09:30:41
Spectrometer Frequency 400.13
Spectral Width 8012.8
Lowest Frequency -1545.1
Nucleus 1H
Acquired Size 32768
Spectral Size 65536
0.95
1.00
1.00
2.00
3.05
3.13
17.71
f1 (ppm)

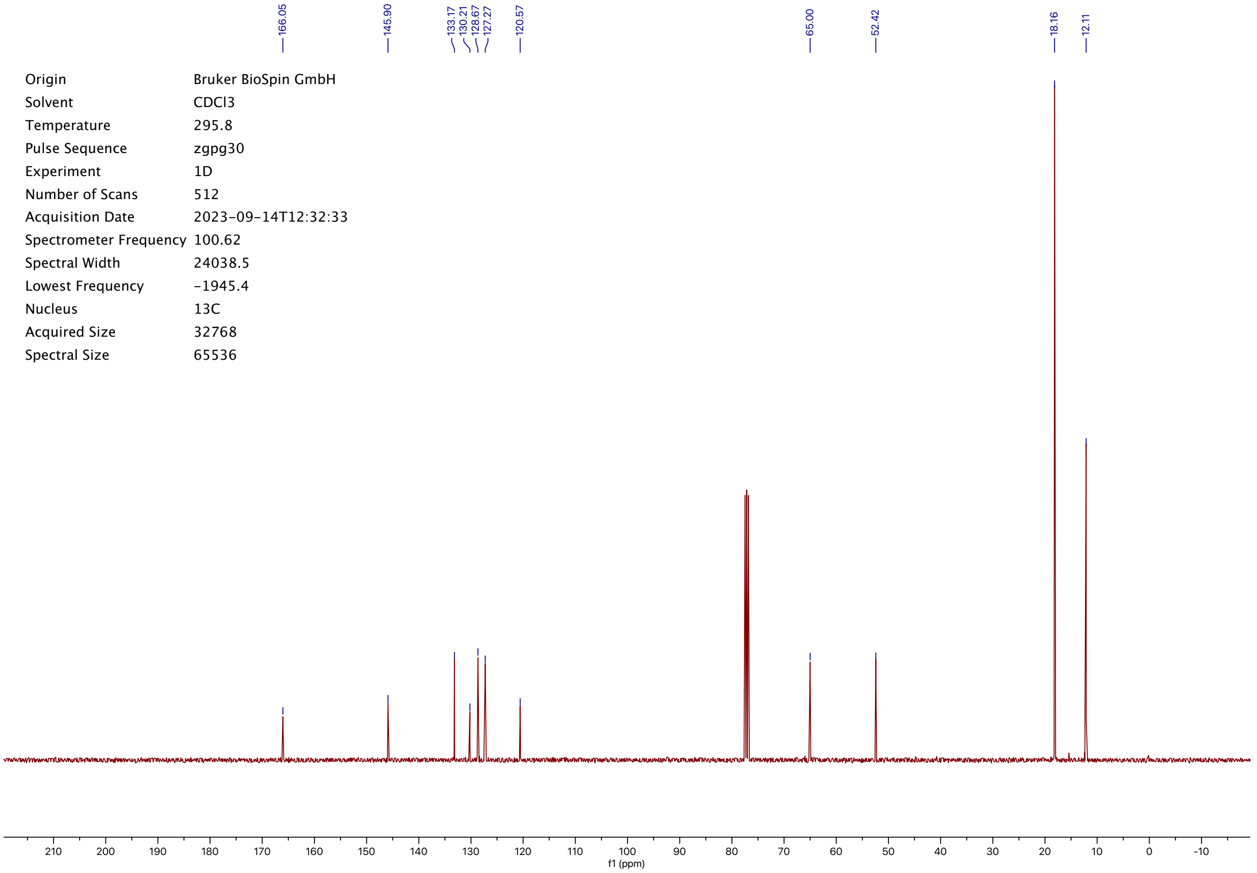
166.05
145.90
133.17
130.21
128.67
127.27
120.57
65.00
52.42
18.16
12.11
Origin Bruker BioSpin GmbH
Solvent CDCl3
Temperature 295.8
Pulse Sequence zgpg30
Experiment 1D
Number of Scans 512
Acquisition Date 2023-09-14T12:32:33
Spectrometer Frequency 100.62
Spectral Width 24038.5
Lowest Frequency -1945.4
Nucleus 13C
Acquired Size 32768
Spectral Size 65536
f1 (ppm)

S57

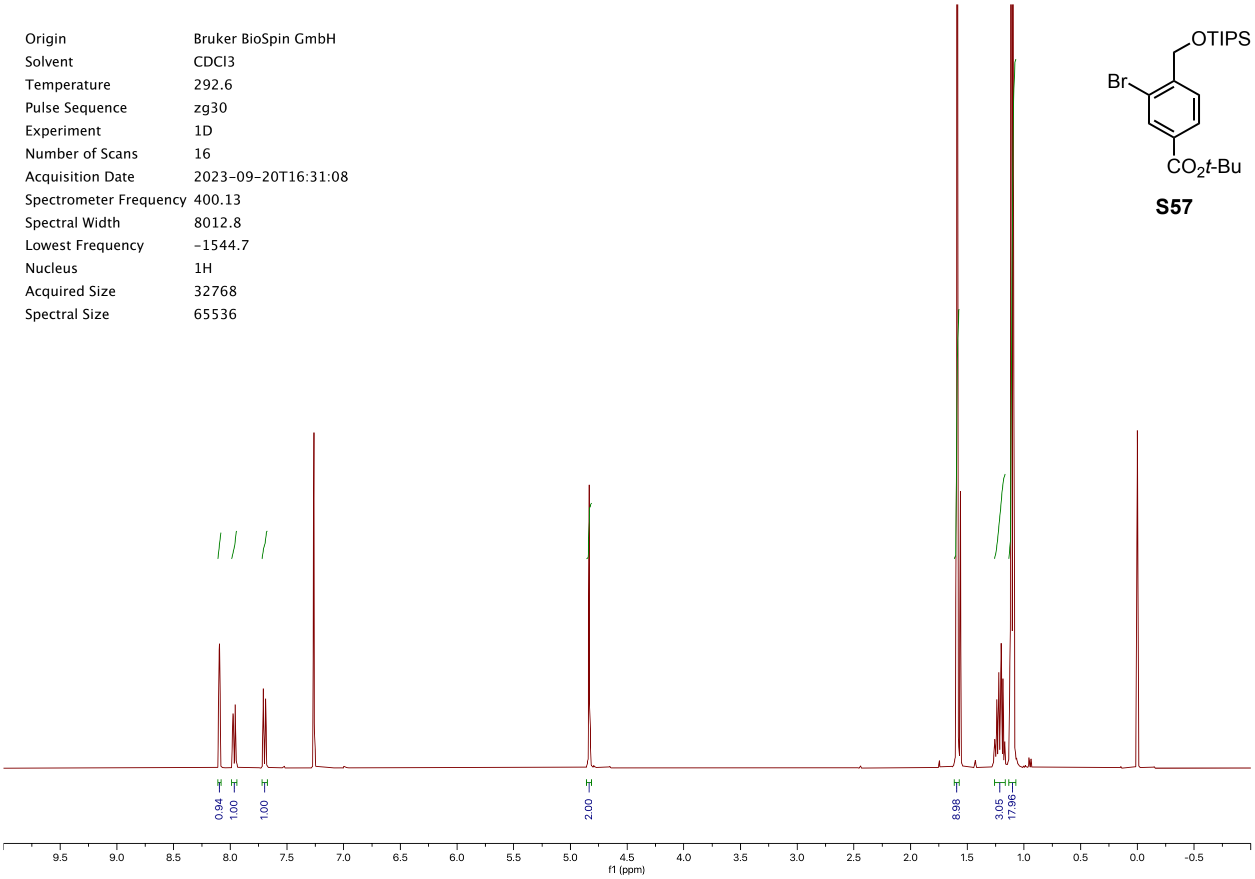
Origin Bruker BioSpin GmbH
Solvent CDCl3
Temperature 292.6
Pulse Sequence zg30
Experiment 1D
Number of Scans 16
Acquisition Date 2023-09-20T16:31:08
Spectrometer Frequency 400.13
Spectral Width 8012.8
Lowest Frequency -1544.7
Nucleus 1H
Acquired Size 32768
Spectral Size 65536
0.94
1.00
1.00
2.00
8.98
3.05
17.96
f1 (ppm)

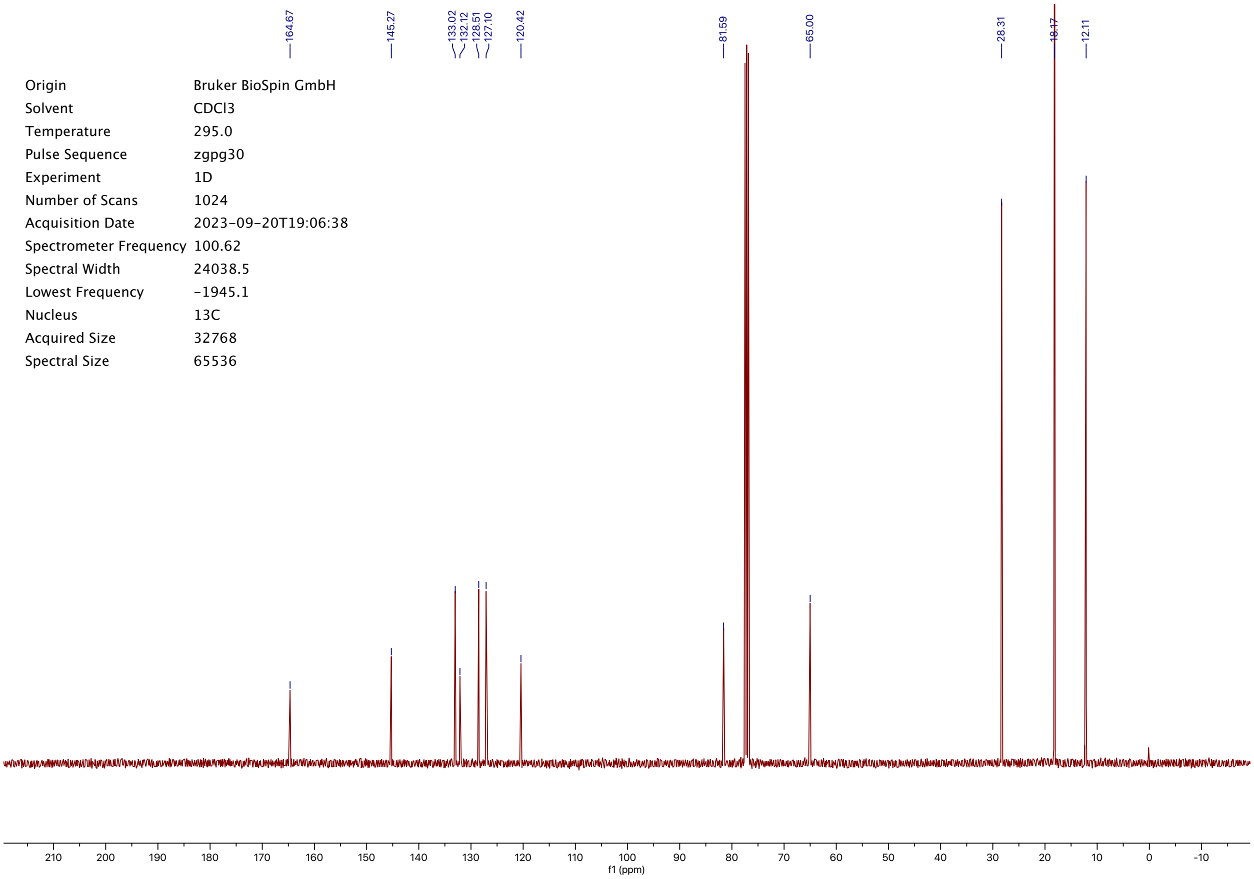
164.67
145.27
133.02
132.12
128.51
127.10
120.42
81.59
65.00
28.31
18.17
12.11
Origin Bruker BioSpin GmbH
Solvent CDCl3
Temperature 295.0
Pulse Sequence zgpg30
Experiment 1D
Number of Scans 1024
Acquisition Date 2023-09-20T19:06:38
Spectrometer Frequency 100.62
Spectral Width 24038.5
Lowest Frequency -1945.1
Nucleus 13C
Acquired Size 32768
Spectral Size 65536
f1 (ppm)

Origin	Bruker BioSpin GmbH
Solvent	CDCl3
Temperature	295.1
Pulse Sequence	zg30
Experiment	1D
Number of Scans	16
Acquisition Date	2023-09-28T10:08:45
Spectrometer Frequency	400.13
Spectral Width	8012.8
Lowest Frequency	-1544.9
Nucleus	1H
Acquired Size	32768
Spectral Size	65536

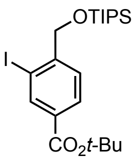

S6

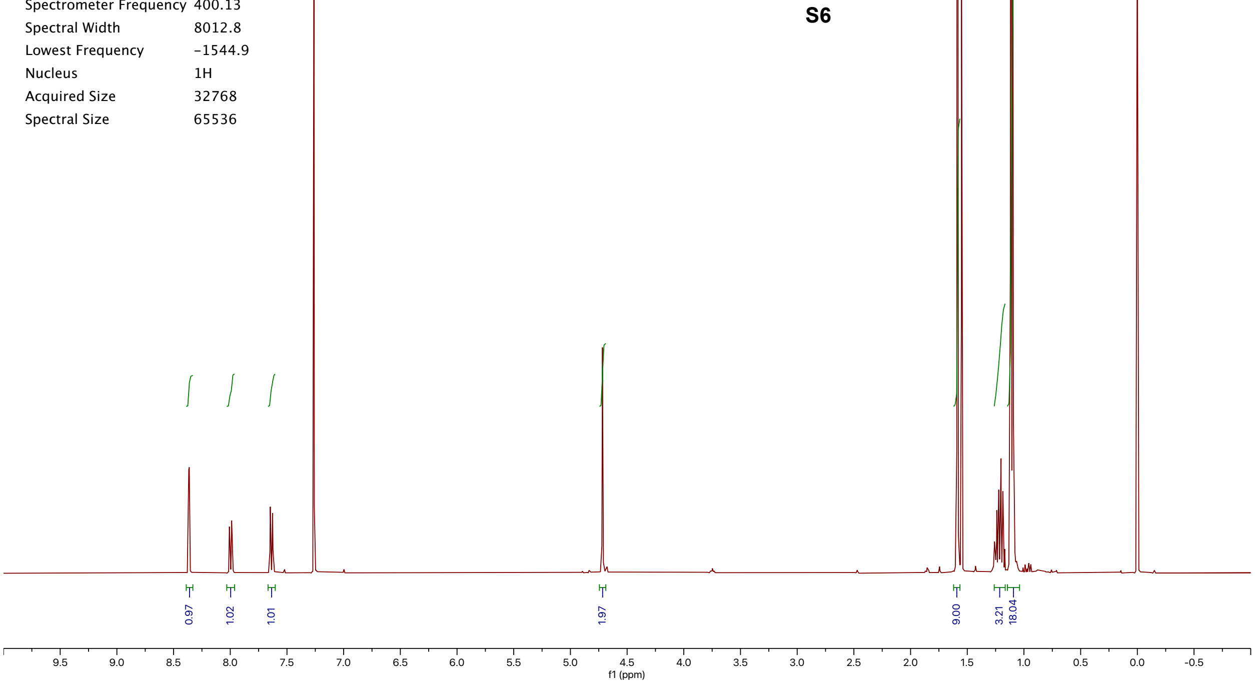

Origin	Bruker BioSpin GmbH
Solvent	CDCl3
Temperature	296.0
Pulse Sequence	zgpg30
Experiment	1D
Number of Scans	512
Acquisition Date	2023-09-22T18:37:48
Spectrometer Frequency	100.62
Spectral Width	24038.5
Lowest Frequency	-1945.4
Nucleus	13C
Acquired Size	32768
Spectral Size	65536

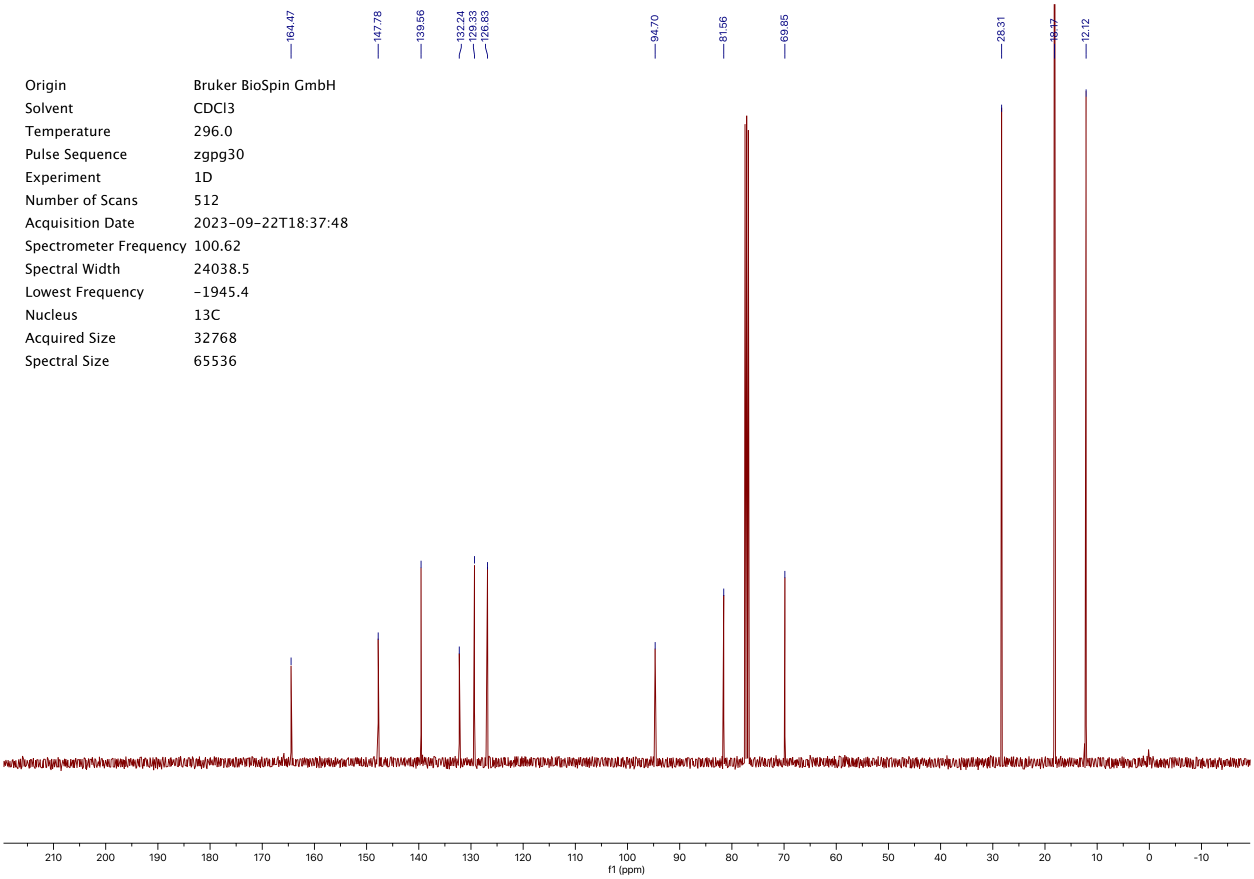

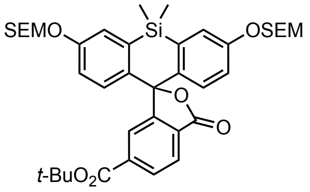

S51

Origin	Bruker BioSpin GmbH
Solvent	CDCl3
Temperature	298.0
Pulse Sequence	zg30
Experiment	1D
Number of Scans	16
Acquisition Date	2023-12-01T11:02:15
Spectrometer Frequency	400.13
Spectral Width	8196.7
Lowest Frequency	-1637.5
Nucleus	1H
Acquired Size	32768
Spectral Size	65536

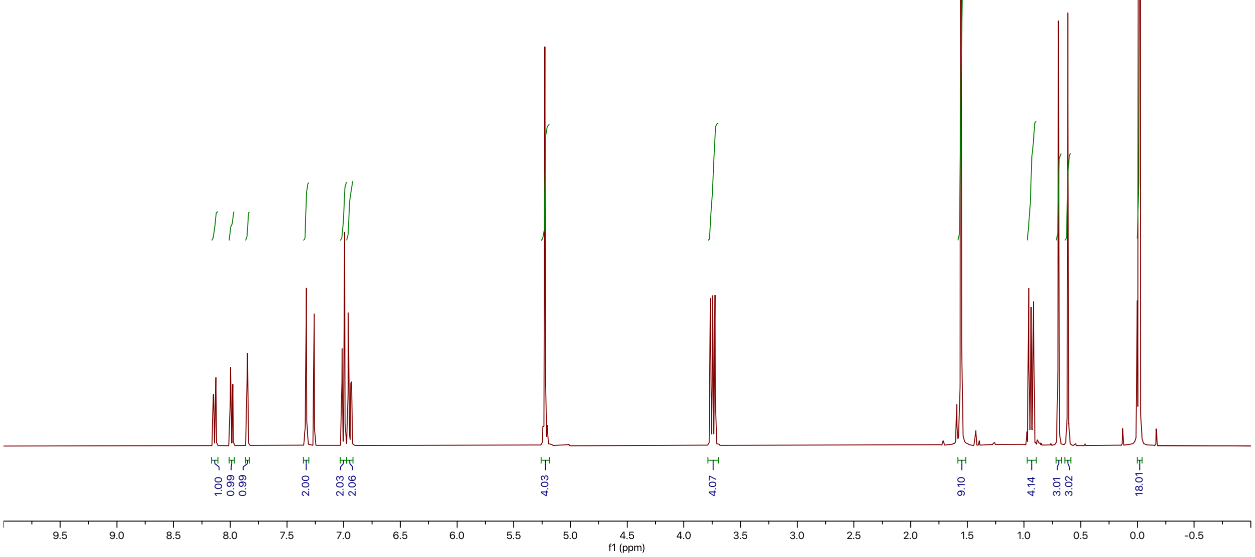

Origin	Bruker BioSpin GmbH
Solvent	CDCl3
Temperature	298.0
Pulse Sequence	zgpg30
Experiment	1D
Number of Scans	512
Acquisition Date	2023-12-01T11:32:45
Spectrometer Frequency	100.62
Spectral Width	23809.5
Lowest Frequency	-1830.9
Nucleus	13C
Acquired Size	32768
Spectral Size	65536

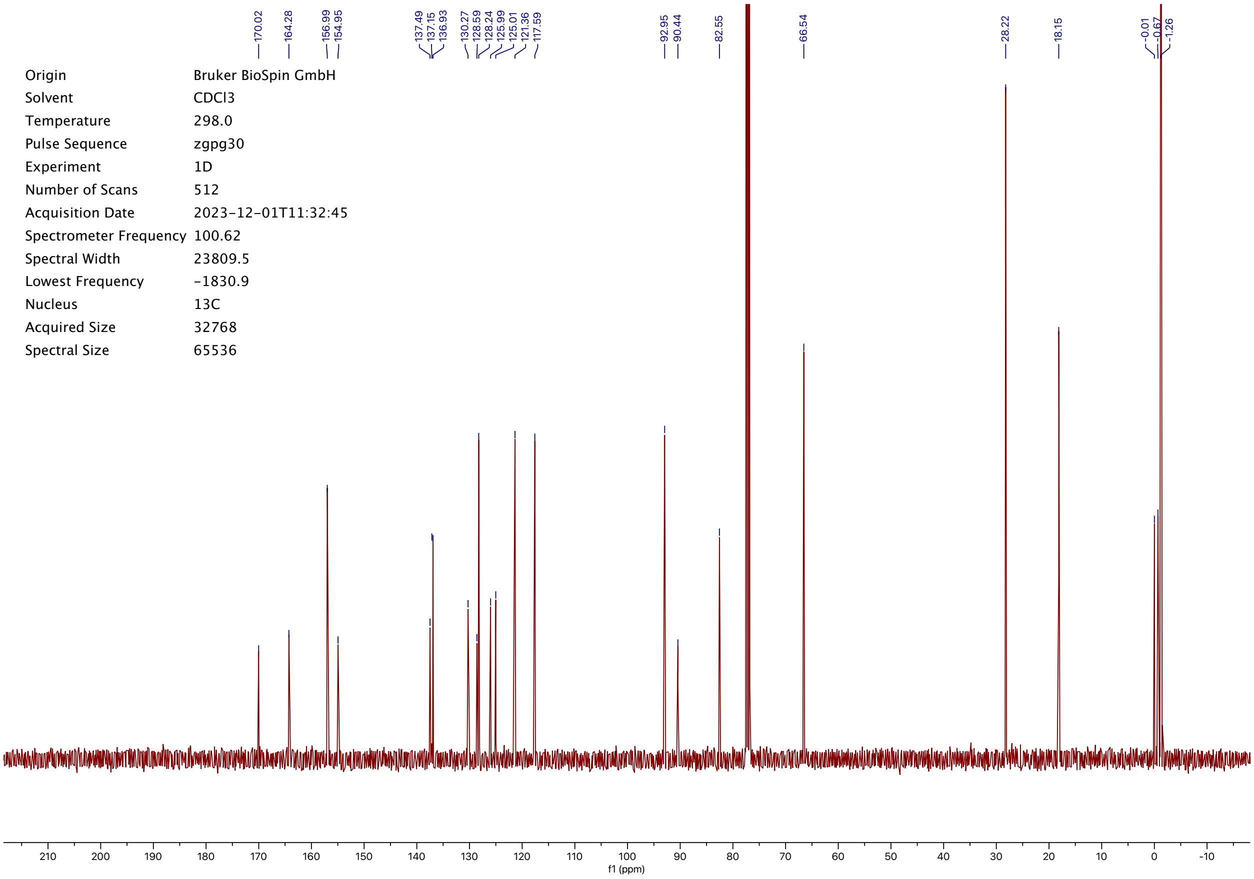

Origin	Bruker BioSpin GmbH
Solvent	CDCl3
Temperature	298.0
Pulse Sequence	zg30
Experiment	1D
Number of Scans	16
Acquisition Date	2023-12-04T09:51:56
Spectrometer Frequency	400.13
Spectral Width	8196.7
Lowest Frequency	-1637.5
Nucleus	1H
Acquired Size	32768
Spectral Size	65536

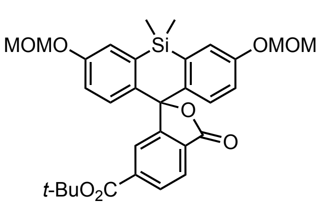

S52

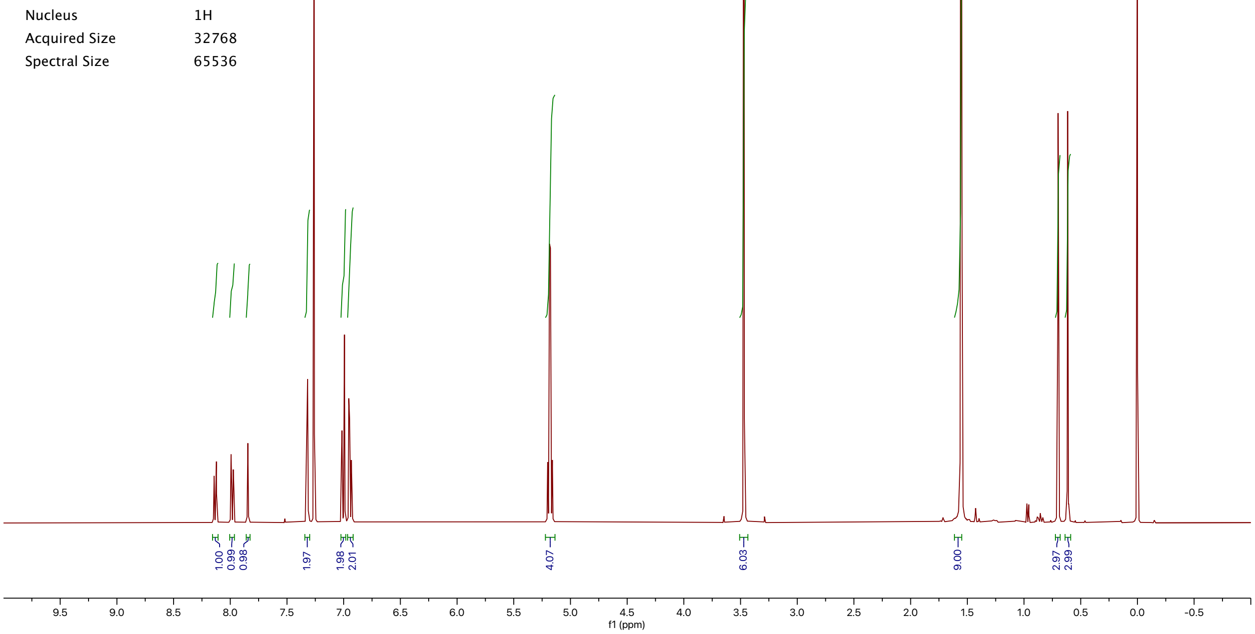

Origin	Bruker BioSpin GmbH
Solvent	CDCl3
Temperature	298.0
Pulse Sequence	zgpg30
Experiment	1D
Number of Scans	512
Acquisition Date	2023-12-01T12:29:14
Spectrometer Frequency	100.62
Spectral Width	23809.5
Lowest Frequency	-1831.2
Nucleus	13C
Acquired Size	32768
Spectral Size	65536

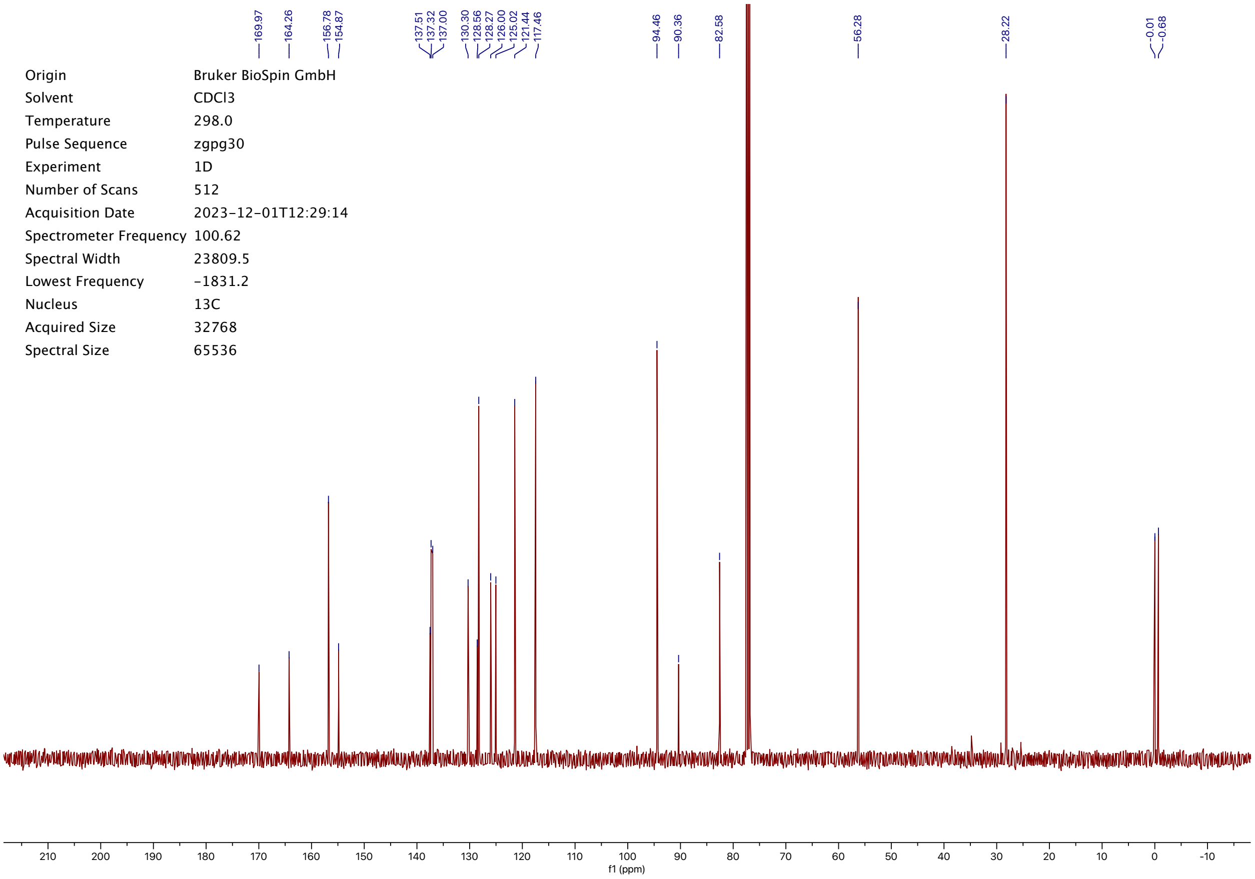

Origin	Bruker BioSpin GmbH
Solvent	DMSO
Temperature	298.0
Pulse Sequence	zg30
Experiment	1D
Number of Scans	16
Acquisition Date	2023-12-01T15:24:12
Spectrometer Frequency	400.13
Spectral Width	8196.7
Lowest Frequency	-1630.4
Nucleus	1H
Acquired Size	32768
Spectral Size	65536

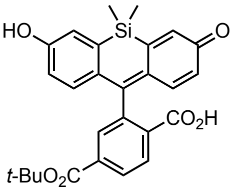

S53

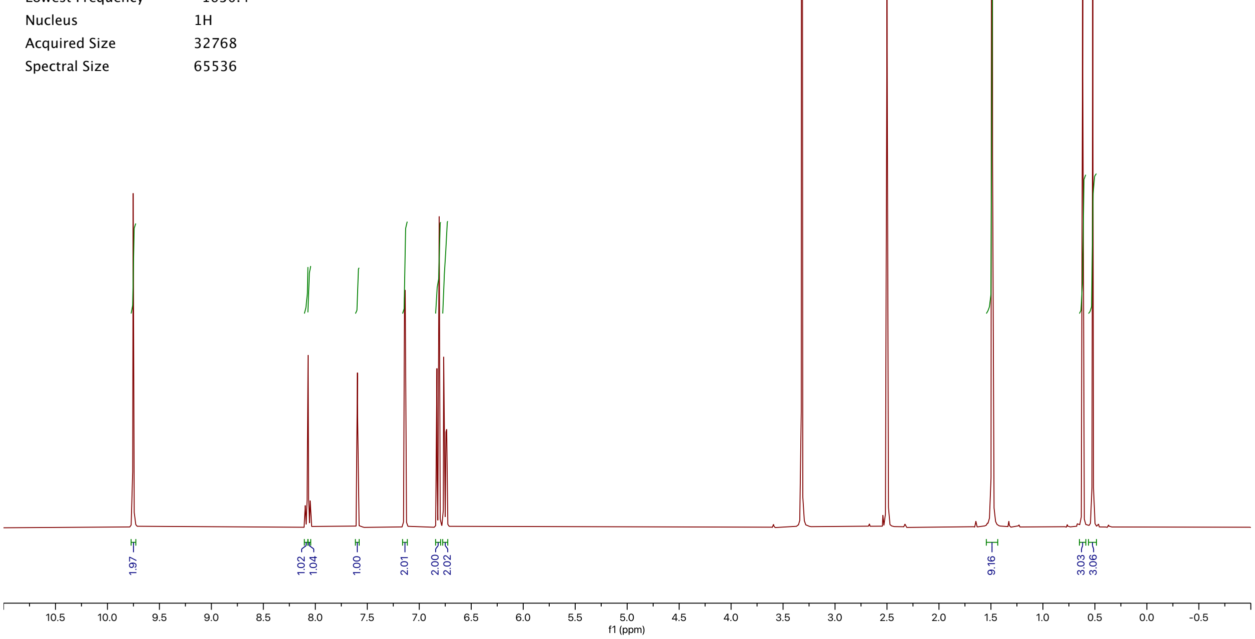

Origin	Bruker BioSpin GmbH
Solvent	DMSO
Temperature	298.0
Pulse Sequence	zgpg30
Experiment	1D
Number of Scans	1024
Acquisition Date	2023-12-01T23:07:03
Spectrometer Frequency	100.62
Spectral Width	23809.5
Lowest Frequency	-1891.1
Nucleus	13C
Acquired Size	32768
Spectral Size	65536

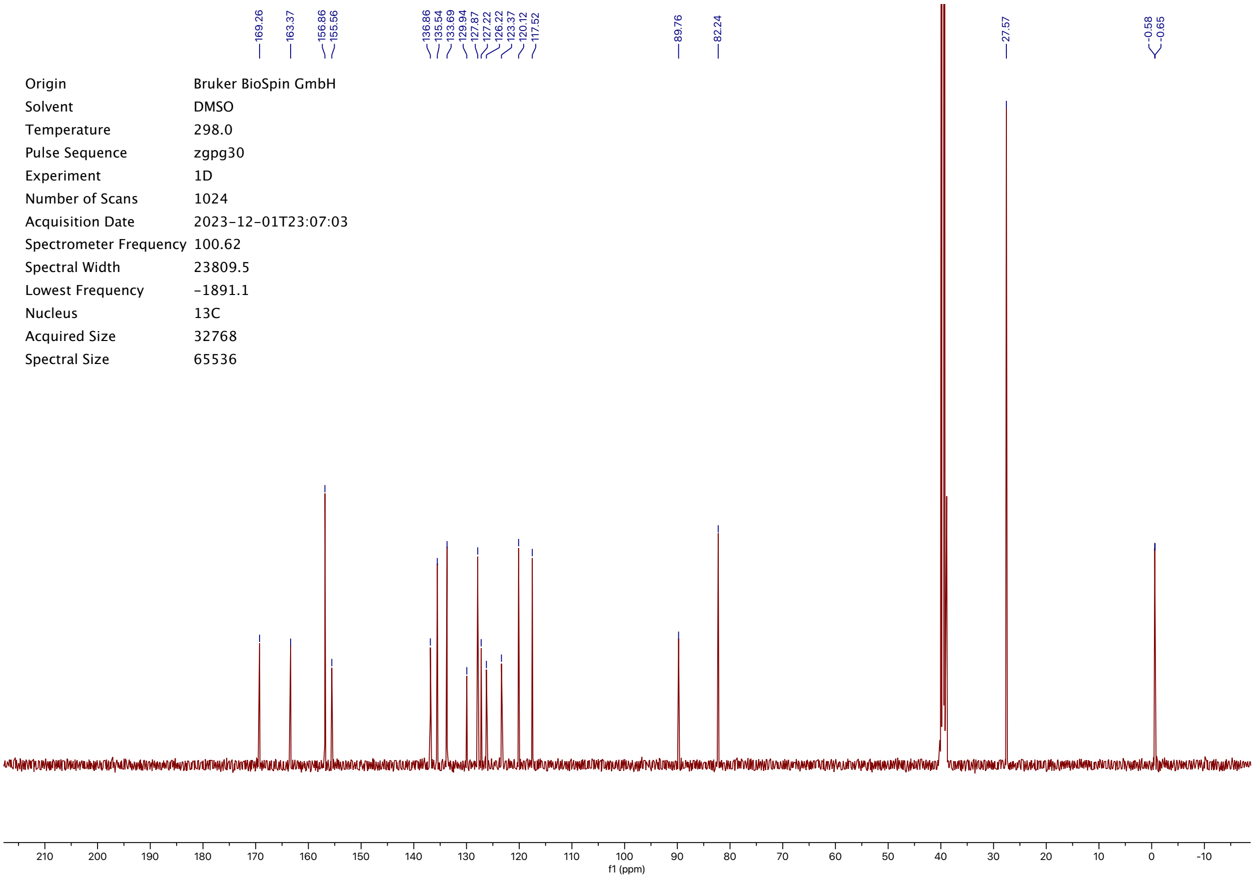

Origin	Bruker BioSpin GmbH
Solvent	MeOD
Temperature	303.0
Pulse Sequence	zg30
Experiment	1D
Number of Scans	16
Acquisition Date	2024-05-31T10:52:19
Spectrometer Frequency	400.13
Spectral Width	8196.7
Lowest Frequency	-1635.1
Nucleus	1H
Acquired Size	32768
Spectral Size	65536

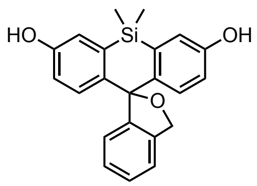

S3

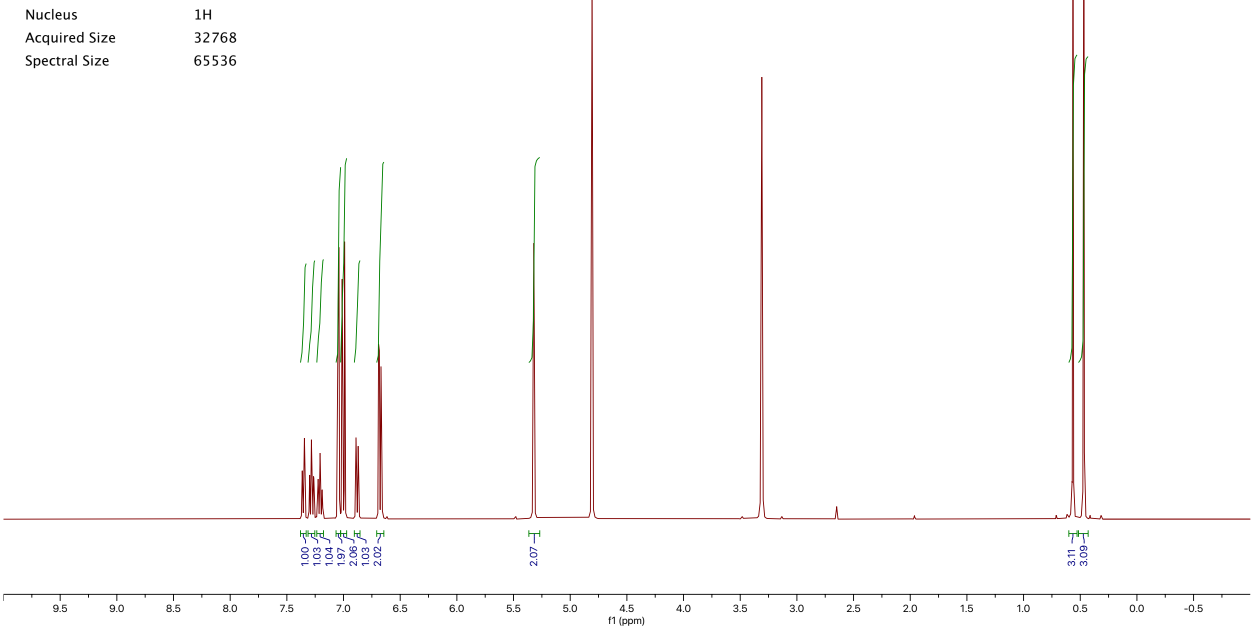

Origin	Bruker BioSpin GmbH
Solvent	MeOD
Temperature	303.0
Pulse Sequence	zgpg30
Experiment	1D
Number of Scans	1024
Acquisition Date	2024-05-31T21:06:41
Spectrometer Frequency	100.62
Spectral Width	23809.5
Lowest Frequency	-1704.6
Nucleus	13C
Acquired Size	32768
Spectral Size	65536

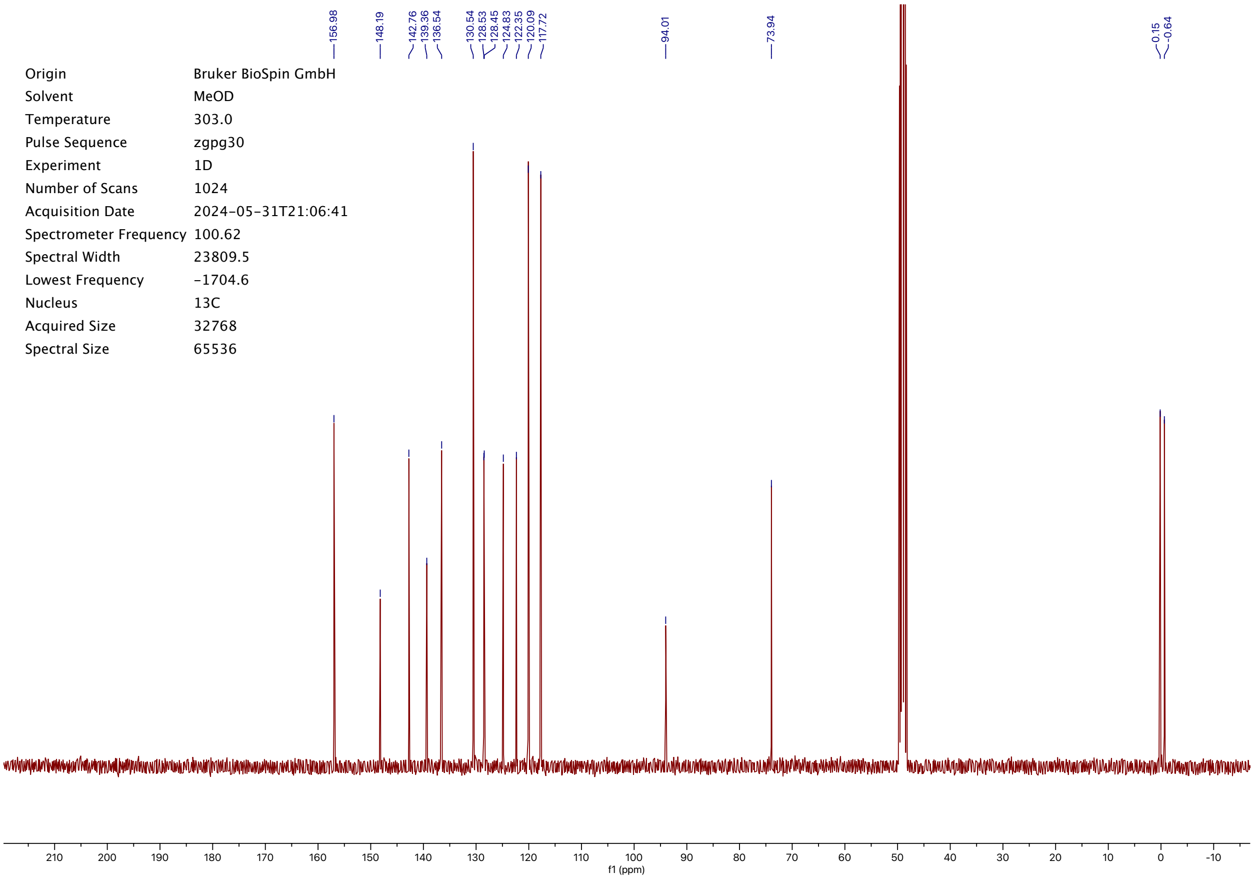

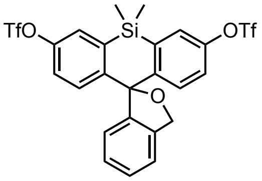
TfO
OTf
Si
O

S4

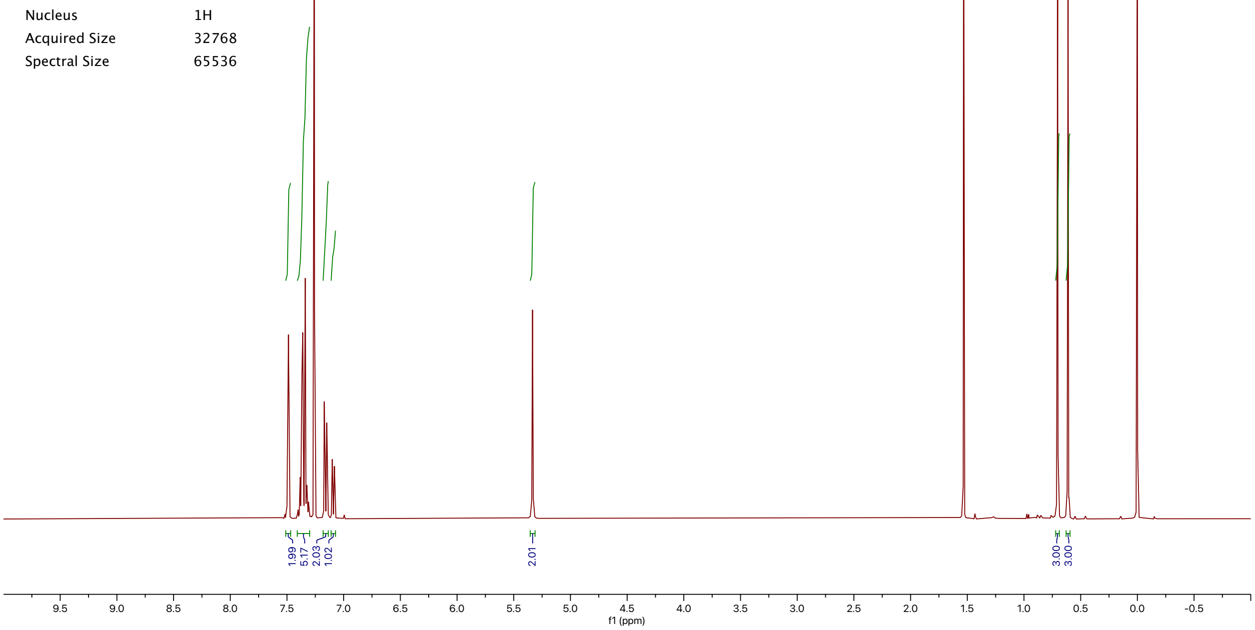
Origin Bruker BioSpin GmbH
Solvent CDCl3
Temperature 300.0
Pulse Sequence zg30
Experiment 1D
Number of Scans 16
Acquisition Date 2024-05-24T10:35:00
Spectrometer Frequency 400.13
Spectral Width 8196.7
Lowest Frequency -1637.8
Nucleus 1H
Acquired Size 32768
Spectral Size 65536
1.99
5.17
2.03
1.02
2.01
3.00
3.00
f1 (ppm)

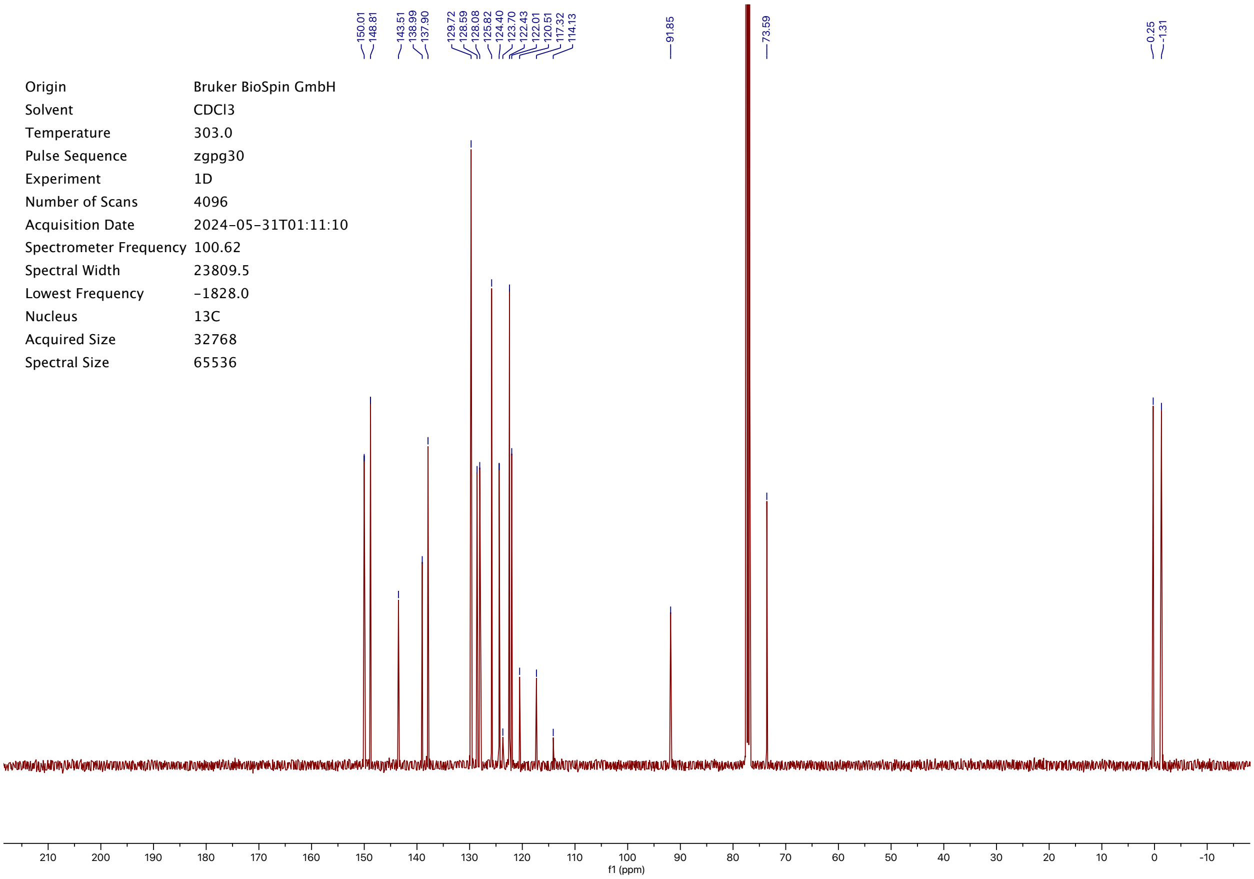
150.01
148.81
143.51
138.99
137.90
129.72
128.59
128.08
125.82
124.40
123.70
122.43
122.01
120.51
117.32
114.13
91.85
73.59
0.25
-1.31
Origin Bruker BioSpin GmbH
Solvent CDCl3
Temperature 303.0
Pulse Sequence zgpg30
Experiment 1D
Number of Scans 4096
Acquisition Date 2024-05-31T01:11:10
Spectrometer Frequency 100.62
Spectral Width 23809.5
Lowest Frequency -1828.0
Nucleus 13C
Acquired Size 32768
Spectral Size 65536
f1 (ppm)

Origin	Bruker BioSpin GmbH
Solvent	DMSO
Temperature	295.1
Pulse Sequence	zg30
Experiment	1D
Number of Scans	16
Acquisition Date	2023-10-02T16:16:18
Spectrometer Frequency	400.13
Spectral Width	8012.8
Lowest Frequency	-1535.6
Nucleus	1H
Acquired Size	32768
Spectral Size	65536

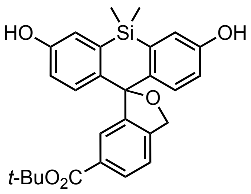

S7

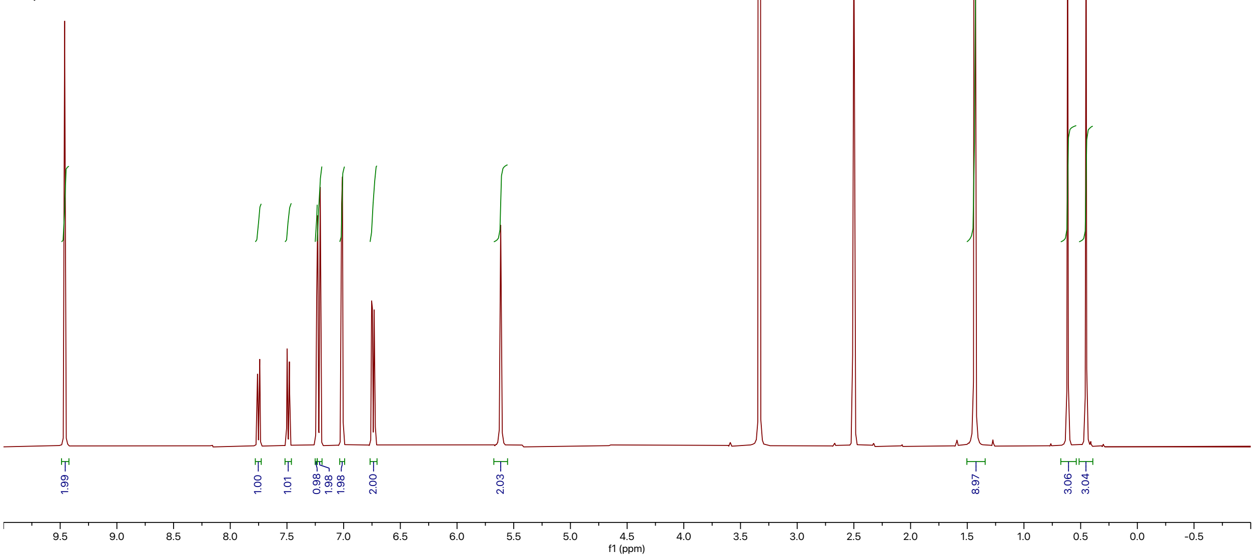

Origin	Bruker BioSpin GmbH
Solvent	DMSO
Temperature	296.0
Pulse Sequence	zgpg30
Experiment	1D
Number of Scans	2048
Acquisition Date	2023-10-02T21:04:59
Spectrometer Frequency	100.62
Spectral Width	24038.5
Lowest Frequency	-2004.8
Nucleus	13C
Acquired Size	32768
Spectral Size	65536

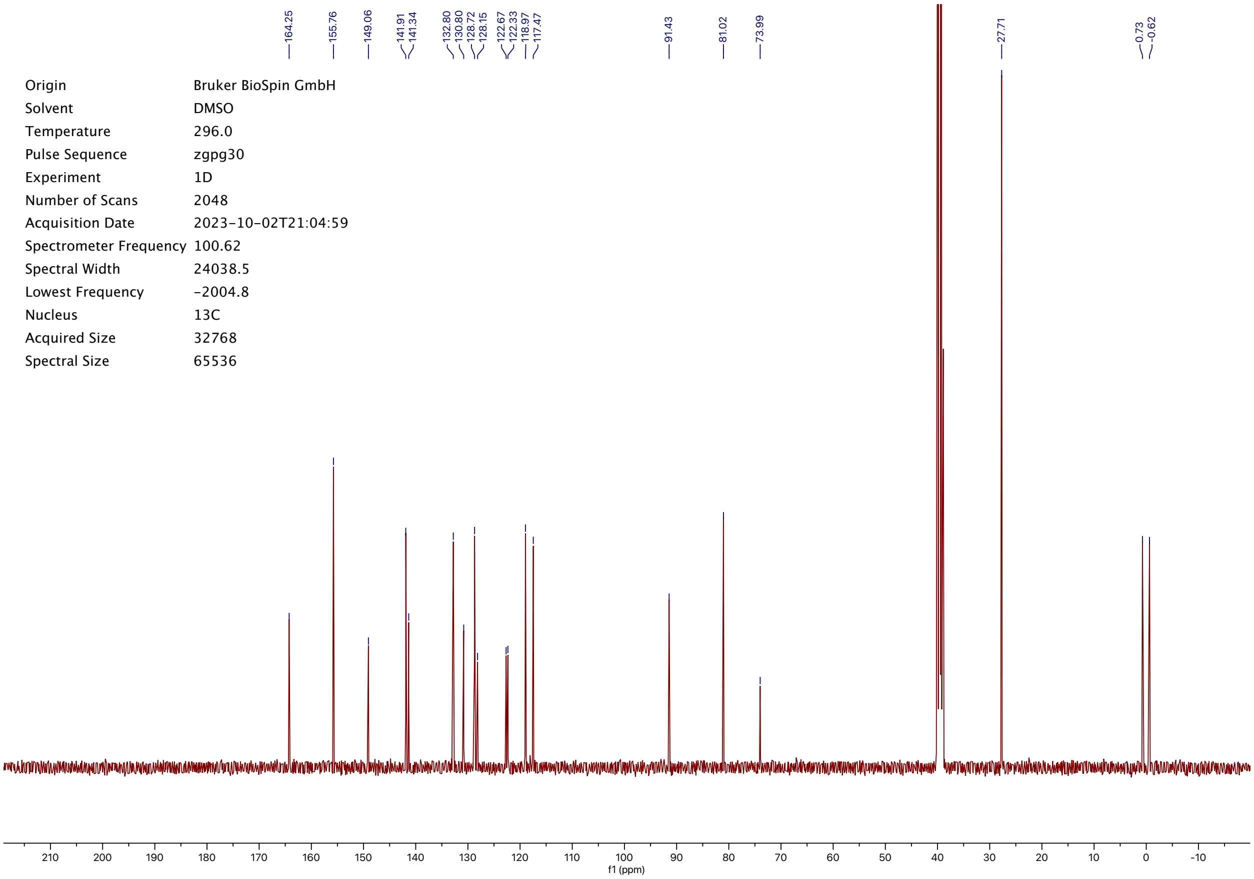

Origin	Bruker BioSpin GmbH
Solvent	CDCl3
Temperature	295.4
Pulse Sequence	zg30
Experiment	1D
Number of Scans	16
Acquisition Date	2023-09-15T12:03:08
Spectrometer Frequency	400.13
Spectral Width	8012.8
Lowest Frequency	-1545.5
Nucleus	1H
Acquired Size	32768
Spectral Size	65536

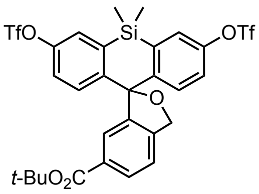

10

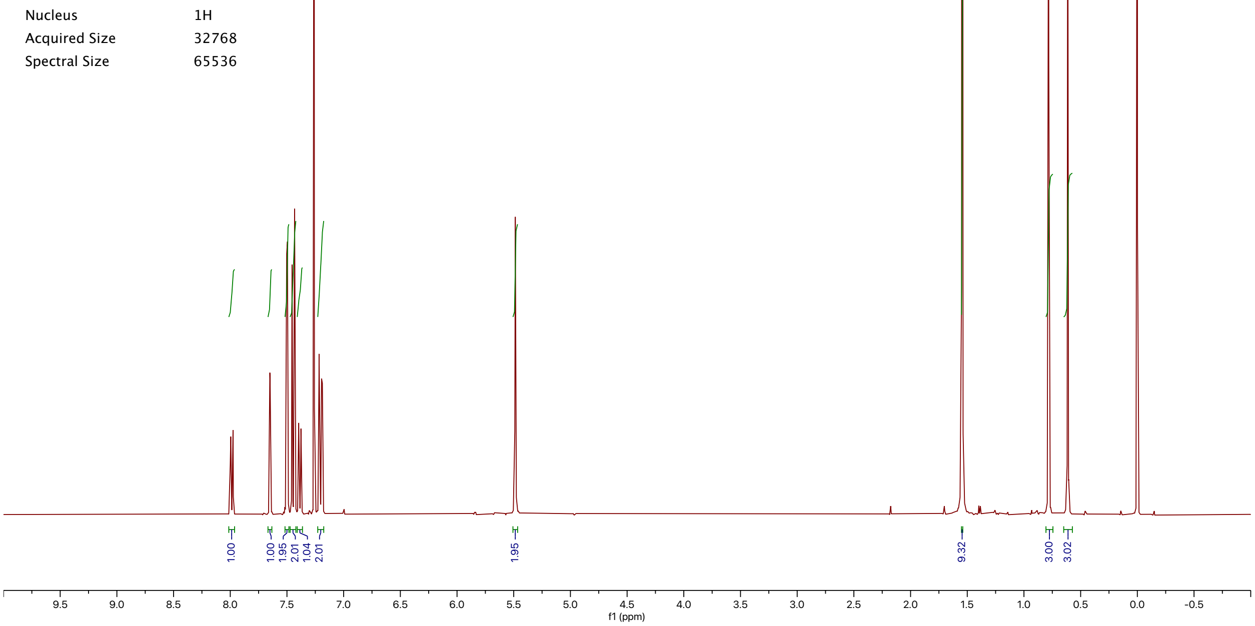

Origin	Bruker BioSpin GmbH
Solvent	CDCl3
Temperature	296.0
Pulse Sequence	zgpg30
Experiment	1D
Number of Scans	2048
Acquisition Date	2023-09-15T20:01:54
Spectrometer Frequency	100.62
Spectral Width	24038.5
Lowest Frequency	-1945.0
Nucleus	13C
Acquired Size	32768
Spectral Size	65536

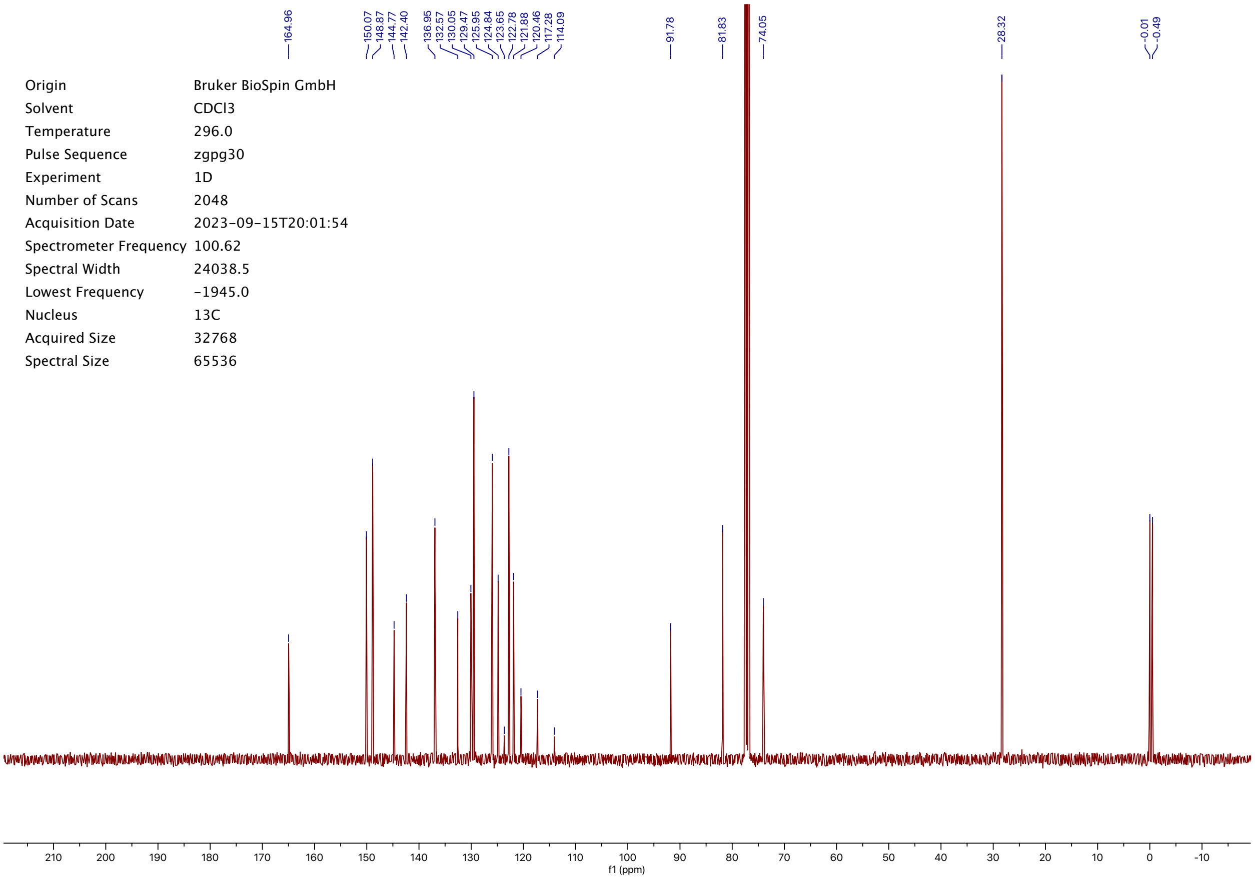

SUPPLEMENTARY NOTE 2

JFb Synthesis via Pd-Catalyzed C–N Cross-Couplings

General method S1: C–N cross-coupling of ditriflate. The following procedure for HM-SiR (**1**) is representative. A vial was charged with ditriflate **S4** (300 mg, 0.480 mmol), dimethylamine hydrochloride (94.0 mg, 1.15 mmol, 2.4 eq), RuPhos-G3-palladacycle (40.2 mg, 48.0 μmol, 0.1 eq), RuPhos (22.4 mg, 48.0 μmol, 0.1 eq), and Cs_2CO_3 (751 mg, 2.31 mmol, 4.8 eq). The vial was sealed and evacuated/backfilled with nitrogen (3×). Dioxane (3 mL) was added, and the reaction was flushed again with nitrogen (3×). The reaction was then stirred at 100 °C for 4 h. It was subsequently cooled to room temperature, filtered through Celite with CH_2Cl_2, and concentrated to dryness. Purification by silica gel chromatography (0–30% EtOAc/hexanes, linear gradient) afforded **1** (185 mg, 93%) as a white solid.

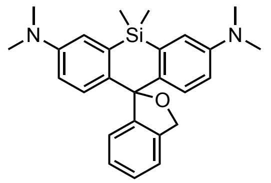

HM-SiR (1): The title compound (93%, white solid) was prepared from ditriflate **S4** and dimethylamine hydrochloride according to method S1. ^{1}H NMR ($CDCl_3$, 400 MHz) δ 7.31 – 7.26 (m, 2H), 7.25 – 7.21 (m, 1H), 7.06 (dt, *J* = 7.3, 1.0 Hz, 1H), 6.971 (d, *J* = 8.8 Hz, 2H), 6.970 (d, *J* = 2.9 Hz, 2H), 6.61 (dd, *J* = 8.9, 2.9 Hz, 2H), 5.23 (s, 2H), 2.94 (s, 12H), 0.61 (s, 3H), 0.54 (s, 3H); ^{13}C NMR ($CDCl_3$, 101 MHz) δ 148.8 (C), 146.3 (C), 139.9 (C), 138.6 (C), 135.7 (C), 128.7 (CH), 127.4 (CH), 127.2 (CH), 124.7 (CH), 121.1 (CH), 116.9 (CH), 114.0 (CH), 92.7 (C), 72.4 (CH_2), 40.7 (CH_3), 0.8 (CH_3), -1.1 (CH_3); Analytical HPLC: t_R = 10.7 min, >99% purity (10–95% MeCN/H_2O, linear gradient, with constant 0.1% v/v TFA additive; 20 min run; 1 mL/min flow; ESI; positive ion mode; detection at 650 nm); HRMS (ESI) calcd for $C_{26}H_{31}N_2OSi$ $[M+H]^+$ 415.2200, found 415.2192.

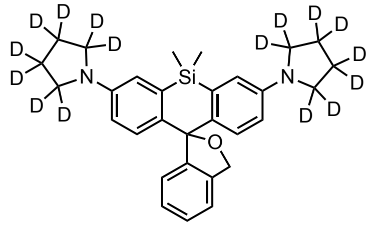

JFX$_{650}$b (2): The title compound (85%, light blue solid) was prepared from ditriflate **S4** and pyrrolidine-2,2,3,3,4,4,5,5-*d*$_8$ according to method S1. ^{1}H NMR ($CDCl_3$, 400 MHz) δ 7.31 – 7.24 (m, 2H), 7.24 – 7.19 (m, 1H), 7.04 (dt, *J* = 7.5, 1.1 Hz, 1H), 6.96 (d, *J* = 8.7 Hz, 2H), 6.78 (d, *J* = 2.8 Hz, 2H), 6.44 (dd, *J* = 8.8, 2.8 Hz, 2H), 5.24 (s, 2H), 0.61 (s, 3H), 0.54 (s, 3H); ^{13}C NMR ($CDCl_3$, 101 MHz) δ 146.9 (C), 146.3 (C), 139.7 (C), 137.5 (C), 135.6 (C), 128.9 (CH), 127.4 (CH), 127.0 (CH), 124.7 (CH), 121.1 (CH), 115.7 (CH), 113.0 (CH), 92.9 (C), 72.4 (CH_2), 0.7 (CH_3), -1.0 (CH_3); Analytical HPLC: t_R = 13.1 min, >99% purity (10–95% MeCN/H_2O, linear gradient, with constant

0.1% v/v TFA additive; 20 min run; 1 mL/min flow; ESI; positive ion mode; detection at 650 nm); HRMS (ESI) calcd for $C_{30}H_{19}D_{16}N_2OSi$ $[M+H]^+$ 483.3517, found 483.3518.

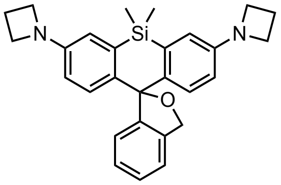

JF$_{646}$b (3): The title compound (72%, white solid) was prepared from ditriflate **S4** and azetidine according to method S1. ^{1}H NMR ($CDCl_3$, 400 MHz) δ 7.32 – 7.27 (m, 2H), 7.25 – 7.21 (m, 1H), 7.06 (dt, *J* = 7.4, 1.1 Hz, 1H), 6.95 (d, *J* = 8.6 Hz, 2H), 6.66 (d, *J* = 2.6 Hz, 2H), 6.31 (dd, *J* = 8.6, 2.6 Hz, 2H), 5.21 (s, 2H), 3.87 (t, *J* = 7.2 Hz, 8H), 2.34 (p, *J* = 7.2 Hz, 4H), 0.58 (s, 3H), 0.52 (s, 3H); ^{13}C NMR ($CDCl_3$, 101 MHz) δ 150.4 (C), 146.1 (C), 139.9 (C), 139.3 (C), 135.6 (C), 128.4 (CH), 127.3 (CH), 127.2 (CH), 124.8 (CH), 121.2 (CH), 115.6 (CH), 112.6 (CH), 92.9 (C), 72.4 (CH_2), 52.6 (CH_2), 17.2 (CH_2), 0.7 (CH_3), -1.2 (CH_3); Analytical HPLC: t_R = 12.2 min, >99% purity (10–95% MeCN/H_2O, linear gradient, with constant 0.1% v/v TFA additive; 20 min run; 1 mL/min flow; ESI; positive ion mode; detection at 650 nm); HRMS (ESI) calcd for $C_{28}H_{31}N_2OSi$ $[M+H]^+$ 439.2200, found 439.2191.

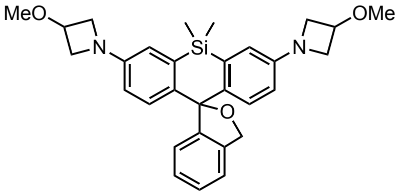

JF$_{639}$b (4): The title compound (85%, white solid) was prepared from ditriflate **S4** and 3-methoxyazetidine hydrochloride according to method S1. ^{1}H NMR ($CDCl_3$, 400 MHz) δ 7.31 – 7.27 (m, 2H), 7.25 – 7.22 (m, 1H), 7.05 (dt, *J* = 7.3, 1.1 Hz, 1H), 6.96 (d, *J* = 8.6 Hz, 2H), 6.68 (d, *J* = 2.6 Hz, 2H), 6.33 (dd, *J* = 8.6, 2.7 Hz, 2H), 5.22 (s, 2H), 4.32 (tt, *J* = 6.1, 4.5 Hz, 2H), 4.14 – 4.05 (m, 4H), 3.75 – 3.66 (m, 4H), 3.32 (s, 6H), 0.59 (s, 3H), 0.52 (s, 3H); ^{13}C NMR ($CDCl_3$, 101 MHz) δ 149.8 (C), 146.1 (C), 139.8 (C), 139.7 (C), 135.7 (C), 128.5 (CH), 127.4 (CH), 127.3 (CH), 124.8 (CH), 121.2 (CH), 116.0 (CH), 113.0 (CH), 92.8 (C), 72.5 (CH_2), 70.3 (CH), 59.0 (CH_2), 56.1 (CH_3), 0.7 (CH_3), -1.2 (CH_3); Analytical HPLC: t_R = 11.9 min, >99% purity (10–95% MeCN/H_2O, linear gradient, with constant 0.1% v/v TFA additive; 20 min run; 1 mL/min flow; ESI; positive ion mode; detection at 650 nm); HRMS (ESI) calcd for $C_{30}H_{35}N_2O_3Si$ $[M+H]^+$ 499.2411, found 499.2417.

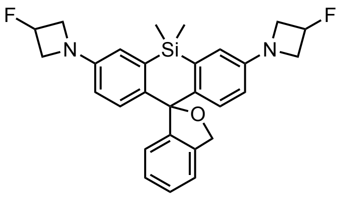

JF$_{635}$b (5): The title compound (89%, white solid) was prepared from ditriflate **S4** and 3-fluoroazetidine hydrochloride according to method S1. ^{1}H NMR ($CDCl_3$, 400 MHz) δ 7.32 – 7.28 (m, 2H), 7.27 – 7.22 (m, 1H), 7.05

(dt, J = 7.3, 1.1 Hz, 1H), 6.99 (d, J = 8.6 Hz, 2H), 6.69 (d, J = 2.7 Hz, 2H), 6.35 (dd, J = 8.6, 2.7 Hz, 2H), 5.40 (dtt, $^{2}J_{FH}$ = 57.1 Hz, J = 5.9, 3.8 Hz, 2H), 5.22 (s, 2H), 4.24 – 4.13 (m, 4H), 4.01 – 3.90 (m, 4H), 0.60 (s, 3H), 0.54 (s, 3H); ^{19}F NMR ($CDCl_3$, 376 MHz) δ -180.46 (dtt, J_{FH} = 57.0, 24.3, 18.0 Hz); ^{13}C NMR ($CDCl_3$, 101 MHz) δ 149.4 (C), 145.8 (C), 140.2 (C), 139.8 (C), 135.8 (C), 128.6 (CH), 127.4 (CH), 124.7 (CH), 121.3 (CH), 116.2 (CH), 113.2 (CH), 92.7 (C), 82.9 (d, $^{1}J_{CF}$ = 204.5 Hz, CHF), 72.5 (CH_2), 59.71 (d, $^{2}J_{CF}$ = 23.3 Hz, CH_2), 0.7 (CH_3), -1.2 (CH_3); Analytical HPLC: t_R = 13.1 min, >99% purity (10–95% MeCN/H_2O, linear gradient, with constant 0.1% v/v TFA additive; 20 min run; 1 mL/min flow; ESI; positive ion mode; detection at 650 nm); HRMS (ESI) calcd for $C_{28}H_{29}F_2N_2OSi$ [M+H]$^+$ 475.2012, found 475.2016.

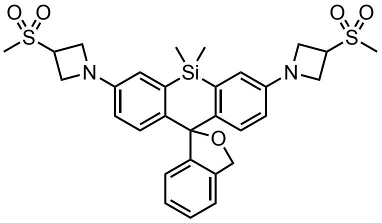

JF$_{630}$b (6): The title compound (57%, white solid) was prepared from ditriflate **S4** and 3-methylsulfonyl-azetidine hydrochloride according to method S1. ^{1}H NMR (DMSO-d_6, 400 MHz) δ 7.40 – 7.35 (m, 1H), 7.24 (td, J = 7.4, 1.1 Hz, 1H), 7.17 (td, J = 7.3, 1.0 Hz, 1H), 7.05 (d, J = 8.6 Hz, 2H), 6.80 – 6.76 (m, 1H), 6.71 (d, J = 2.6 Hz, 2H), 6.42 (dd, J = 8.7, 2.6 Hz, 2H), 5.37 (s, 2H), 4.40 (tt, J = 8.2, 5.4 Hz, 2H), 4.16 – 4.08 (m, 4H), 4.08 – 4.00 (m, 4H), 3.03 (s, 6H), 0.59 (s, 3H), 0.46 (s, 3H); ^{13}C NMR (DMSO-d_6, 101 MHz) δ 148.7 (C), 147.3 (C), 140.5 (C), 137.4 (C), 133.3 (C), 127.9 (CH), 127.4 (CH), 126.9 (CH), 122.9 (CH), 121.6 (CH), 115.1 (CH), 113.0 (CH), 91.8 (C), 73.0 (CH_2), 51.8 (CH_2), 50.2 (CH), 38.1 (CH_3), 0.0 (CH_3), -0.3 (CH_3); Analytical HPLC: t_R = 12.1 min, >99% purity (10–95% MeCN/H_2O, linear gradient, with constant 0.1% v/v TFA additive; 20 min run; 1 mL/min flow; ESI; positive ion mode; detection at 650 nm); HRMS (ESI) calcd for $C_{30}H_{35}N_2O_5S_2Si$ [M+H]$^+$ 595.1751, found 595.1758.

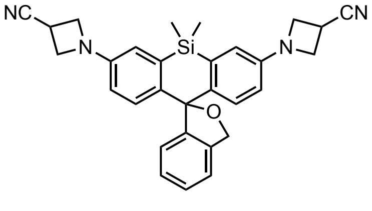

JF$_{629}$b (7): The title compound (92%, white solid) was prepared from ditriflate **S4** and azetidine-3-carbonitrile hydrochloride according to method S1. ^{1}H NMR ($CDCl_3$, 400 MHz) δ 7.33 – 7.28 (m, 2H), 7.28 – 7.23 (m, 1H), 7.04 (dt, J = 7.5, 1.0 Hz, 1H), 7.03 (d, J = 8.6 Hz, 2H), 6.67 (d, J = 2.7 Hz, 2H), 6.34 (dd, J = 8.6, 2.6 Hz, 2H), 5.23 (s, 2H), 4.23 – 4.14 (m, 4H), 4.11 – 4.02 (m, 4H), 3.57 (tt, J = 8.4, 6.4 Hz, 2H), 0.60 (s, 3H), 0.53 (s, 3H); ^{13}C NMR ($CDCl_3$, 101 MHz) δ 148.7 (C), 145.6 (C), 141.1 (C), 139.6 (C), 135.9 (C), 128.6 (CH), 127.6 (CH), 127.5 (CH), 124.6 (CH), 121.4 (CH), 119.9 (C), 115.9 (CH), 113.0 (CH), 92.5 (C), 72.7 (CH_2), 55.4 (CH_2), 18.6 (CH), 0.6 (CH_3), -1.1 (CH_3); Analytical HPLC: t_R = 14.2 min, >99% purity (10–95% MeCN/H_2O, linear gradient, with constant 0.1% v/v TFA additive; 20 min run; 1 mL/min flow; ESI; positive ion mode; detection at 650 nm); HRMS (ESI) calcd for $C_{30}H_{29}N_4OSi$ [M+H]$^+$ 489.2105, found 489.2099.

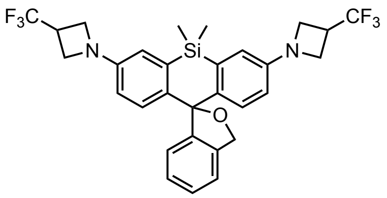

JF$_{626}$b (8): The title compound (92%, white solid) was prepared from ditriflate **S4** and 3-trifluoromethyl-azetidine hydrochloride according to method S1. ^{1}H NMR ($CDCl_3$, 400 MHz) δ 7.33 – 7.28 (m, 2H), 7.26 (s, 1H), 7.05 (dt, J = 7.4, 1.1 Hz, 1H), 7.00 (d, J = 8.6 Hz, 2H), 6.67 (d, J = 2.6 Hz, 2H), 6.33 (dd, J = 8.6, 2.7 Hz, 2H), 5.22 (s, 2H), 4.09 – 4.02 (m, 4H), 3.97 – 3.91 (m, 4H), 3.45 – 3.30 (m, 2H), 0.59 (s, 3H), 0.53 (s, 3H); ^{19}F NMR ($CDCl_3$, 376 MHz) δ -73.31 (d, $^{3}J_{FH}$ = 8.7 Hz); ^{13}C NMR ($CDCl_3$, 101 MHz) δ 148.9 (C), 145.7 (C), 140.3 (C), 139.8 (C), 135.9 (C), 128.6 (CH), 127.47 (CH), 127.43 (CH), 126.5 (q, $^{1}J_{CF}$ = 275.1 Hz, CF_3), 124.7 (CH), 121.3 (CH), 115.7 (CH), 112.6 (CH), 92.7 (C), 72.5 (CH_2), 51.4 (q, $^{3}J_{CF}$ = 3.6 Hz, CH_2), 33.3 (q, $^{2}J_{CF}$ = 32.0 Hz, CHF), 0.7 (CH_3), -1.2 (CH_3); Analytical HPLC: t_R = 16.1 min, >99% purity (10–95% MeCN/H_2O, linear gradient, with constant 0.1% v/v TFA additive; 20 min run; 1 mL/min flow; ESI; positive ion mode; detection at 650 nm); HRMS (ESI) calcd for $C_{30}H_{29}F_6N_2OSi$ $[M+H]^+$ 575.1948, found 575.1942.

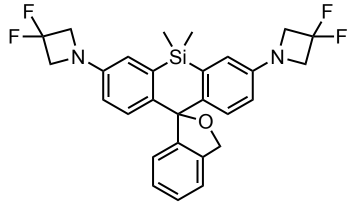

JF$_{614}$b (9): The title compound (70%, white solid) was prepared from ditriflate **S4** and 3,3-difluoroazetidine hydrochloride according to method S1. ^{1}H NMR ($CDCl_3$, 400 MHz) δ 7.34 – 7.29 (m, 2H), 7.29 – 7.24 (m, 1H), 7.05 (dt, J = 7.4, 1.0 Hz, 1H), 7.04 (d, J = 8.6 Hz, 2H), 6.71 (d, J = 2.7 Hz, 2H), 6.38 (dd, J = 8.7, 2.7 Hz, 2H), 5.24 (s, 2H), 4.22 (t, $^{3}J_{HF}$ = 11.8 Hz, 8H), 0.61 (s, 3H), 0.54 (s, 3H); ^{19}F NMR ($CDCl_3$, 376 MHz) δ -99.83 (p, $^{3}J_{FH}$ = 11.8 Hz); ^{13}C NMR ($CDCl_3$, 101 MHz) δ 148.1 (t, $^{4}J_{CF}$ = 2.6 Hz, C), 145.6 (C), 141.0 (C), 139.7 (C), 136.0 (C), 128.7 (CH), 127.6 (CH), 127.5 (CH), 124.6 (CH), 121.4 (CH), 116.6 (CH), 116.1 (t, $^{1}J_{CF}$ = 274.7 Hz, CF_2), 113.7 (CH), 92.5 (C), 72.7 (CH_2), 63.5 (t, $^{2}J_{CF}$ = 25.6 Hz, CH_2), 0.6 (CH_3), -1.2 (CH_3); Analytical HPLC: t_R = 17.6 min, 98.5% purity (10–95% MeCN/H_2O, linear gradient, with constant 0.1% v/v TFA additive; 20 min run; 1 mL/min flow; ESI; positive ion mode; detection at 254 nm); HRMS (ESI) calcd for $C_{28}H_{27}F_4N_2OSi$ $[M+H]^+$ 511.1823, found 511.1831.

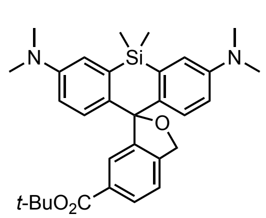

6-*tert*-Butoxycarbonyl-HM-SiR (S8): The title compound (60%, off-white solid) was prepared from ditriflate **10** and dimethylamine hydrochloride according to method S1. ^{1}H NMR ($CDCl_3$, 400 MHz) δ 7.91 (dd, J = 7.9, 1.5 Hz, 1H),

7.61 (dd, J = 1.4, 0.7 Hz, 1H), 7.31 (dd, J = 7.9, 0.8 Hz, 1H), 7.07 (d, J = 8.9 Hz, 2H), 6.96 (d, J = 2.9 Hz, 2H), 6.65 (dd, J = 8.9, 2.9 Hz, 2H), 5.38 (s, 2H), 2.95 (s, 12H), 1.51 (s, 9H), 0.67 (s, 3H), 0.55 (s, 3H); ^{13}C NMR ($CDCl_3$, 101 MHz) δ 165.6 (C), 148.9 (C), 147.6 (C), 143.4 (C), 138.8 (C), 134.9 (C), 131.9 (C), 128.9 (CH), 128.4 (CH), 125.3 (CH), 121.1 (CH), 116.8 (CH), 114.2 (CH), 92.6 (C), 81.1 (C), 73.0 (CH_2), 40.6 (CH_3), 28.3 (CH_3), 0.4 (CH_3), -0.2 (CH_3); HRMS (ESI) calcd for $C_{31}H_{39}N_2O_3Si$ [M+H]$^+$ 515.2724, found 515.2712.

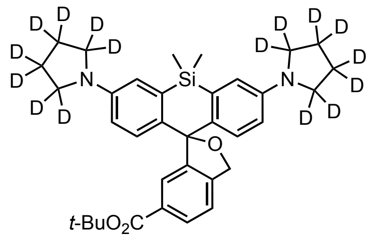

6-*tert*-Butoxycarbonyl-JFX$_{650}$b (S9): The title compound (27%, pale yellow solid) was prepared from ditriflate **10** and pyrrolidine-2,2,3,3,4,4,5,5-d_8 according to method S1. ^{1}H NMR ($CDCl_3$, 400 MHz) δ 7.89 (dd, J = 7.9, 1.5 Hz, 1H), 7.58 (d, J = 1.4 Hz, 1H), 7.30 (d, J = 7.9 Hz, 1H), 7.08 (d, J = 8.8 Hz, 2H), 6.77 (d, J = 2.8 Hz, 2H), 6.49 (dd, J = 8.8, 2.7 Hz, 2H), 5.40 (s, 2H), 1.50 (s, 9H), 0.66 (s, 3H), 0.54 (s, 3H); ^{13}C NMR ($CDCl_3$, 101 MHz) δ 165.6 (C), 148.1 (C), 146.3 (C), 143.1 (C), 137.8 (C), 134.7 (C), 131.8 (C), 128.7 (CH), 128.5 (CH), 125.1 (CH), 121.0 (CH), 115.6 (CH), 113.3 (CH), 92.8 (C), 81.0 (C), 73.1 (CH_2), 28.3 (CH_3), 0.3 (CH_3), -0.0 (CH_3); HRMS (ESI) calcd for $C_{35}H_{27}D_{16}N_2O_3Si$ [M+H]$^+$ 583.4042, found 583.4034.

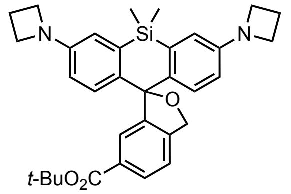

6-*tert*-Butoxycarbonyl-JF$_{646}$b (S10): The title compound (91%, off-white solid) was prepared from ditriflate **10** and azetidine according to method S1. ^{1}H NMR ($CDCl_3$, 400 MHz) δ 7.90 (dd, J = 7.9, 1.5 Hz, 1H), 7.60 (d, J = 1.4 Hz, 1H), 7.30 (d, J = 8.0 Hz, 1H), 7.05 (d, J = 8.6 Hz, 2H), 6.65 (d, J = 2.7 Hz, 2H), 6.35 (dd, J = 8.7, 2.7 Hz, 2H), 5.36 (s, 2H), 3.88 (t, J = 7.2 Hz, 8H), 2.34 (p, J = 7.2 Hz, 4H), 1.51 (s, 9H), 0.64 (s, 3H), 0.52 (s, 3H); ^{13}C NMR ($CDCl_3$, 101 MHz) δ 165.6 (C), 150.4 (C), 147.4 (C), 143.3 (C), 139.4 (C), 134.7 (C), 131.8 (C), 128.9 (CH), 128.1 (CH), 125.3 (CH), 121.1 (CH), 115.5 (CH), 112.9 (CH), 92.8 (C), 81.2 (C), 73.0 (CH_2), 52.5 (CH_2), 28.3 (CH_3), 17.1 (CH_2), 0.3 (CH_3), -0.3 (CH_3); HRMS (ESI) calcd for $C_{33}H_{39}N_2O_3Si$ [M+H]$^+$ 539.2724, found 539.2725.

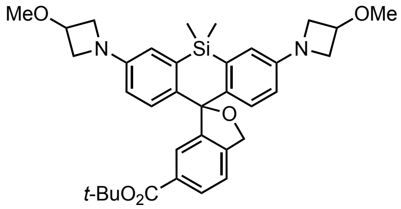

6-*tert*-Butoxycarbonyl-JF$_{639}$b (S11): The title compound (91%, white solid) was prepared from ditriflate **10** and 3-methoxyazetidine hydrochloride according to method S1. ^{1}H NMR ($CDCl_3$, 400 MHz) δ 7.91 (dd, J = 7.9, 1.5 Hz, 1H), 7.59 (d, J = 1.6 Hz, 1H), 7.31 (d, J = 8.0 Hz, 1H), 7.06 (d, J = 8.6 Hz, 2H), 6.67 (d, J = 2.7 Hz, 2H), 6.38 (dd, J = 8.6, 2.6 Hz, 2H), 5.37 (s, 2H), 4.33 (tt, J = 6.1, 4.5 Hz, 2H), 4.14 – 4.07 (m, 4H), 3.75 – 3.68 (m, 4H), 3.32 (s, 6H), 1.51 (s, 9H), 0.64 (s, 3H), 0.52 (s, 3H); ^{13}C NMR ($CDCl_3$, 101 MHz) δ 165.6 (C), 149.8 (C), 147.3 (C), 143.2 (C), 139.8 (C), 134.8 (C), 131.8 (C), 128.9 (CH), 128.2 (CH), 125.2 (CH), 121.2 (CH), 115.9 (CH), 113.3 (CH), 92.7 (C), 81.2 (C), 73.1 (CH_2), 70.2 (CH), 59.0 (CH_2), 56.2 (CH_3), 28.3 (CH_3), 0.3 (CH_3), -0.3 (CH_3); HRMS (ESI) calcd for $C_{35}H_{43}N_2O_5Si$ $[M+H]^+$ 599.2936, found 599.2933.

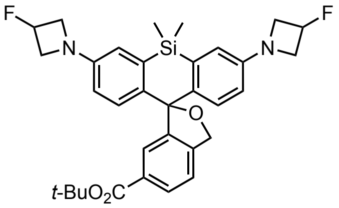

6-*tert*-Butoxycarbonyl-JF$_{635}$b (S12): The title compound (25%, light blue solid) was prepared from ditriflate **10** and 3-fluoroazetidine hydrochloride according to method S1. ^{1}H NMR ($CDCl_3$, 400 MHz) δ 7.92 (dd, J = 7.9, 1.5 Hz, 1H), 7.60 (d, J = 1.4 Hz, 1H), 7.32 (d, J = 8.0 Hz, 1H), 7.09 (d, J = 8.6 Hz, 2H), 6.68 (d, J = 2.7 Hz, 2H), 6.39 (dd, J = 8.7, 2.7 Hz, 2H), 5.41 (dtt, $^{2}J_{HF}$ = 57.0 Hz, J = 6.0, 3.7 Hz, 2H), 5.37 (s, 2H), 4.24 – 4.14 (m, 4H), 4.02 – 3.91 (m, 4H), 1.52 (s, 9H), 0.66 (s, 3H), 0.53 (s, 3H); ^{19}F NMR ($CDCl_3$, 376 MHz) δ -180.44 (dtt, J_{FH} = 56.9, 23.9, 18.0 Hz); ^{13}C NMR ($CDCl_3$, 101 MHz) δ 165.5 (C), 149.36 (d, $^{4}J_{CF}$ = 1.5 Hz, C), 147.1 (C), 143.2 (C), 140.3 (C), 134.9 (C), 131.9 (C), 129.0 (CH), 128.2 (CH), 125.2 (CH), 121.2 (CH), 116.1 (CH), 113.5 (CH), 92.6 (C), 82.94 (d, $^{1}J_{CF}$ = 204.5 Hz, CHF), 81.3 (C), 73.1 (CH_2), 59.68 (d, $^{2}J_{CF}$ = 23.5 Hz, CH_2), 28.3 (CH_3), 0.3 (CH_3), -0.3 (CH_3); HRMS (ESI) calcd for $C_{33}H_{37}F_2N_2O_3Si$ $[M+H]^+$ 575.2536, found 575.2535.

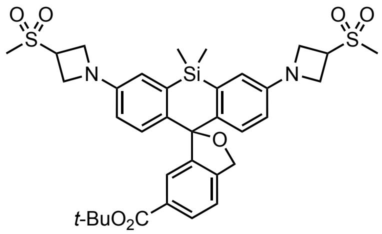

6-*tert*-Butoxycarbonyl-JF$_{630}$b (S13): The title compound (41%, light blue solid) was prepared from ditriflate **10** and 3-methylsulfonyl-azetidine hydrochloride according to method S1. ^{1}H NMR ($CDCl_3$, 400 MHz) δ 7.91 (dd, J = 7.9, 1.4 Hz, 1H), 7.57 (d, J = 1.7 Hz, 1H), 7.32 (d, J = 8.0 Hz, 1H), 7.13 (d, J = 8.6 Hz, 2H), 6.68 (d, J = 2.7 Hz, 2H), 6.41

(dd, J = 8.6, 2.7 Hz, 2H), 5.40 (s, 2H), 4.27 – 4.17 (m, 8H), 4.10 – 4.01 (m, 2H), 2.99 (s, 6H), 1.52 (s, 9H), 0.66 (s, 3H), 0.53 (s, 3H); ^{13}C NMR ($CDCl_3$, 101 MHz) δ 165.4 (C), 148.5 (C), 147.0 (C), 142.9 (C), 141.2 (C), 134.8 (C), 132.0 (C), 129.1 (CH), 128.3 (CH), 125.0 (CH), 121.3 (CH), 115.9 (CH), 113.4 (CH), 92.5 (C), 81.3 (C), 73.3 (CH_2), 52.6 (CH_2), 51.9 (CH), 38.1 (CH_3), 28.3 (CH_3), 0.3 (CH_3), -0.2 (CH_3); HRMS (ESI) calcd for $C_{35}H_{43}N_2O_7S_2Si$ $[M+H]^+$ 695.2275, found 695.2274.

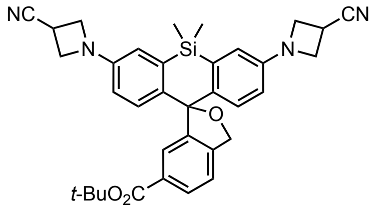

6-*tert*-Butoxycarbonyl-JF$_{629}$b (S14): The title compound (49%, pale green solid) was prepared from ditriflate **10** and azetidine-3-carbonitrile hydrochloride according to method S1. ^{1}H NMR ($CDCl_3$, 400 MHz) δ 7.92 (dd, J = 8.0, 1.5 Hz, 1H), 7.59 (d, J = 1.4 Hz, 1H), 7.33 (d, J = 7.9 Hz, 1H), 7.12 (d, J = 8.6 Hz, 2H), 6.66 (d, J = 2.6 Hz, 2H), 6.38 (dd, J = 8.6, 2.7 Hz, 2H), 5.39 (s, 2H), 4.23 – 4.17 (m, 4H), 4.11 – 4.06 (m, 4H), 3.58 (tt, J = 8.4, 6.4 Hz, 2H), 1.52 (s, 9H), 0.67 (s, 3H), 0.53 (s, 3H); ^{13}C NMR ($CDCl_3$, 101 MHz) δ 165.4 (C), 148.7 (C), 146.8 (C), 143.0 (C), 141.2 (C), 134.9 (C), 132.0 (C), 129.1 (CH), 128.3 (CH), 125.0 (CH), 121.3 (CH), 119.9 (C), 115.9 (CH), 113.4 (CH), 92.4 (C), 81.3 (C), 73.3 (CH_2), 55.4 (CH_2), 28.3 (CH_3), 18.5 (CH), 0.3 (CH_3), -0.3 (CH_3); HRMS (ESI) calcd for $C_{35}H_{37}N_4O_3Si$ $[M+H]^+$ 589.2629, found 589.2630.

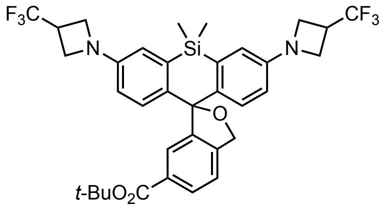

6-*tert*-Butoxycarbonyl-JF$_{626}$b (S15): The title compound (92%, white solid) was prepared from ditriflate **10** and 3-trifluoromethyl-azetidine hydrochloride according to method S1. ^{1}H NMR ($CDCl_3$, 400 MHz) δ 7.92 (dd, J = 7.9, 1.4 Hz, 1H), 7.60 (d, J = 1.4 Hz, 1H), 7.32 (d, J = 8.0 Hz, 1H), 7.10 (d, J = 8.6 Hz, 2H), 6.66 (d, J = 2.6 Hz, 2H), 6.37 (dd, J = 8.6, 2.7 Hz, 2H), 5.37 (s, 2H), 4.09 – 4.02 (m, 4H), 3.99 – 3.92 (m, 4H), 3.45 – 3.30 (m, 2H), 1.52 (s, 9H), 0.65 (s, 3H), 0.53 (s, 3H); ^{19}F NMR ($CDCl_3$, 376 MHz) δ -73.30 (d, $^3J_{FH}$ = 8.8 Hz); ^{13}C NMR ($CDCl_3$, 101 MHz) δ 165.5 (C), 148.9 (C), 147.0 (C), 143.2 (C), 140.4 (C), 135.0 (C), 131.9 (C), 129.0 (CH), 128.3 (CH), 126.5 (q, $^1J_{CF}$ = 275.2 Hz, CF_3), 125.2 (CH), 121.2 (CH), 115.6 (CH), 112.9 (CH), 92.6 (C), 81.3 (C), 73.1 (CH_2), 51.4 (q, $^3J_{CF}$ = 3.6 Hz, CH_2), 33.2 (q, $^2J_{CF}$ = 32.0 Hz, CH), 28.3 (CH_3), 0.3 (CH_3), -0.4 (CH_3); HRMS (ESI) calcd for $C_{35}H_{37}F_6N_2O_3Si$ $[M+H]^+$ 675.2472, found 675.2471.

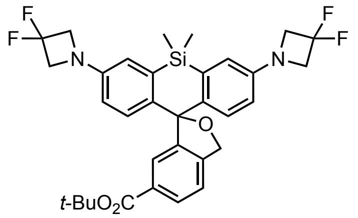

6-*tert*-Butoxycarbonyl-JF$_{614}$b (S16): The title compound (92%, white solid) was prepared from ditriflate **10** and 3,3-difluoroazetidine hydrochloride according to method S1. ^{1}H NMR ($CDCl_3$, 400 MHz) δ 7.93 (dd, J = 7.9, 1.4 Hz, 1H), 7.61 (d, J = 1.4 Hz, 1H), 7.33 (d, J = 7.9 Hz, 1H), 7.14 (d, J = 8.6 Hz, 2H), 6.71 (d, J = 2.7 Hz, 2H), 6.42 (dd, J = 8.6, 2.7 Hz, 2H), 5.39 (s, 2H), 4.23 (t, $^{3}J_{HF}$ = 11.8 Hz, 8H), 1.52 (s, 9H), 0.67 (s, 3H), 0.54 (s, 3H); ^{19}F NMR ($CDCl_3$, 376 MHz) δ -99.78 (p, $^{3}J_{FH}$ = 12.0 Hz); ^{13}C NMR ($CDCl_3$, 101 MHz) δ 165.5 (C), 148.1 (t, $^{4}J_{CF}$ = 2.6 Hz, C), 146.8 (C), 143.1 (C), 141.1 (C), 135.1 (C), 133.0 (C), 129.1 (CH), 128.3 (CH), 125.1 (CH), 121.3 (CH), 116.6 (CH), 116.10 (t, $^{1}J_{CF}$ = 274.6 Hz, CF_2), 114.0 (CH), 92.5 (C), 81.3 (C), 73.2 (CH_2), 63.5 (t, $^{2}J_{CF}$ = 25.6 Hz, CH_2), 28.3 (CH_3), 0.3 (CH_3), -0.3 (CH_3); HRMS (ESI) calcd for $C_{33}H_{35}F_4N_2O_3Si$ $[M+H]^+$ 611.2348, found 611.2342.

Deprotection of JFb *tert*-Butyl Esters

General method S2: *tert*-Butyl ester deprotection. The following procedure for 6-carboxy-JF_{614}b (**S25**) is representative. 6-*tert*-Butoxycarbonyl-JF_{614}b (**S16**; 215 mg, 0.352 mmol) was taken up in CH_2Cl_2 (5 mL), and trifluoroacetic acid (1 mL) was added. The reaction was stirred at room temperature for 4 h. Toluene (5 mL) was added; the reaction mixture was concentrated to dryness and then azeotroped with MeOH (3×). The residue was redissolved in 1:1 THF/MeOH (5 mL), and 1 M NaOH (1.06 mL, 1.06 mmol, 3 eq) was added. After stirring the reaction at room temperature for 1 h, it was acidified with 1 M HCl (1.1 mL), diluted with water, and extracted with EtOAc (2×). The combined organic extracts were washed with brine, dried over anhydrous $MgSO_4$, filtered, and concentrated *in vacuo* to afford 186 mg (95%) of **S25** as a blue solid. Analytical HPLC and NMR indicated that the material was >95% pure and did not require further purification prior to amide coupling.

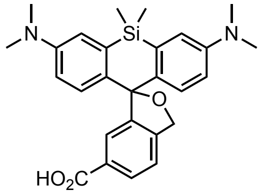

6-Carboxy-HM-SiR (S17): The title compound (45%, blue solid) was prepared from 6-*tert*-butoxycarbonyl-HM-SiR (**S8**) according to method S2, except that the crude product was purified by reverse phase HPLC (10–60% $MeCN/H_2O$, linear gradient, with constant 0.1% TFA additive). ^{1}H NMR ($CDCl_3$, 400 MHz) δ 8.01 (dd, J = 7.9, 1.5 Hz, 1H), 7.77 (dd, J = 1.5, 0.7 Hz, 1H), 7.37 (dd, J = 8.0, 0.8 Hz, 1H), 7.00 (d, J = 8.8 Hz, 2H), 6.97 (d, J = 2.9 Hz, 2H), 6.63 (dd, J = 8.9, 3.0 Hz, 2H), 5.33 (s, 2H), 2.94 (s, 12H), 0.66 (s, 3H), 0.54 (s, 3H); ^{13}C NMR ($CDCl_3$, 101 MHz) δ 171.2 (C), 149.0 (C), 147.5 (C), 145.4 (C), 138.4 (C), 135.4 (C), 129.5 (CH), 129.2 (C), 128.3 (CH), 126.5 (CH), 121.4 (CH), 117.2 (CH), 114.2 (CH), 92.6 (C), 72.6 (CH_2), 40.7 (CH_3), 0.7 (CH_3), -0.8 (CH_3); Analytical HPLC: t_R = 9.0 min, 96.9% purity (10–95% $MeCN/H_2O$, linear gradient, with constant 0.1% v/v TFA additive; 20 min run; 1 mL/min flow; ESI; positive ion mode; detection at 650 nm); HRMS (ESI) calcd for $C_{27}H_{31}N_2O_3Si$ $[M+H]^+$ 459.2098, found 459.2087.

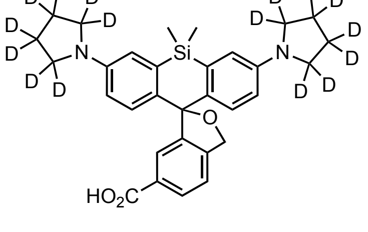

6-Carboxy-JFX_{650}b (S18): The title compound (67%, blue solid) was prepared from 6-*tert*-butoxycarbonyl-JFX_{650}b (**S9**) according to method S2. ^{1}H NMR ($CDCl_3$, 400 MHz) δ 7.92 (dd, J = 7.9, 1.5 Hz, 1H), 7.68 (d, J = 1.5 Hz, 1H), 7.30 (d, J = 8.0 Hz, 1H), 6.92 (d, J = 8.8 Hz, 2H), 6.71 (d, J = 2.8 Hz, 2H), 6.39 (dd, J = 8.8, 2.8 Hz, 2H), 5.27 (s, 2H), 0.58 (s, 3H), 0.47 (s, 3H); Analytical HPLC: t_R = 11.7 min, >99% purity (10–95% $MeCN/H_2O$, linear gradient,

with constant 0.1% v/v TFA additive; 20 min run; 1 mL/min flow; ESI; positive ion mode; detection at 650 nm); HRMS (ESI) calcd for $C_{31}H_{19}D_{16}N_2O_3Si$ [M+H]$^+$ 527.3416, found 527.3409.

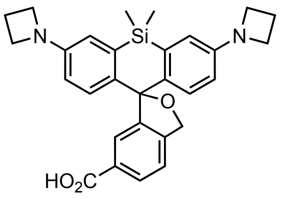

6-Carboxy-JF$_{646}$b (S19): The title compound (87%, dark blue solid) was prepared from 6-*tert*-butoxycarbonyl-JF$_{646}$b (**S10**) according to method S2. ^{1}H NMR ($CDCl_3$, 400 MHz) δ 7.93 (dd, *J* = 7.9, 1.4 Hz, 1H), 7.68 (d, *J* = 1.4 Hz, 1H), 7.29 (d, *J* = 8.0 Hz, 1H), 6.90 (d, *J* = 8.6 Hz, 2H), 6.60 (d, *J* = 2.6 Hz, 2H), 6.27 (dd, *J* = 8.7, 2.7 Hz, 2H), 5.25 (s, 2H), 3.81 (t, *J* = 7.2 Hz, 8H), 2.27 (p, *J* = 7.2 Hz, 4H), 0.56 (s, 3H), 0.45 (s, 3H); ^{13}C NMR ($CDCl_3$, 101 MHz) δ 170.8 (C), 150.5 (C), 147.3 (C), 145.2 (C), 139.1 (C), 135.2 (C), 129.5 (CH), 129.2 (C), 128.1 (CH), 126.5 (CH), 121.4 (CH), 116.0 (CH), 113.0 (CH), 92.7 (C), 72.6 (CH_2), 52.6 (CH_2), 17.1 (CH_2), 0.6 (CH_3), -0.9 (CH_3); Analytical HPLC: t_R = 10.8 min, 98.1% purity (10–95% MeCN/H_2O, linear gradient, with constant 0.1% v/v TFA additive; 20 min run; 1 mL/min flow; ESI; positive ion mode; detection at 650 nm); HRMS (ESI) calcd for $C_{29}H_{31}N_2O_3Si$ [M+H]$^+$ 483.2098, found 483.2105.

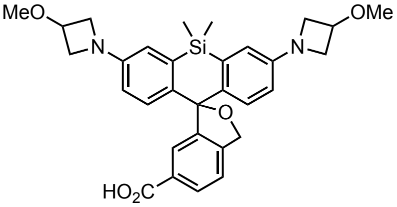

6-Carboxy-JF$_{639}$b (S20): The title compound (95%, dark blue solid) was prepared from 6-*tert*-butoxycarbonyl-JF$_{639}$b (**S11**) according to method S2. ^{1}H NMR ($CDCl_3$, 400 MHz) δ 8.00 (dd, *J* = 8.0, 1.5 Hz, 1H), 7.75 (d, *J* = 1.5 Hz, 1H), 7.37 (d, *J* = 8.0 Hz, 1H), 6.99 (d, *J* = 8.6 Hz, 2H), 6.69 (d, *J* = 2.7 Hz, 2H), 6.36 (dd, *J* = 8.6, 2.7 Hz, 2H), 5.32 (s, 2H), 4.37 – 4.28 (m, 2H), 4.15 – 4.05 (m, 4H), 3.71 (dd, *J* = 7.9, 4.6 Hz, 4H), 3.32 (s, 6H), 0.63 (s, 3H), 0.52 (s, 3H); ^{13}C NMR ($CDCl_3$, 101 MHz) δ 170.9 (C), 149.8 (C), 147.2 (C), 145.2 (C), 139.5 (C), 135.3 (C), 129.6 (CH), 129.2 (C), 128.1 (CH), 126.5 (CH), 121.5 (CH), 116.3 (CH), 113.3 (CH), 92.7 (C), 72.7 (CH_2), 70.2 (CH), 59.0 (CH_2), 56.2 (CH_3), 0.6 (CH_3), -0.9 (CH_3); Analytical HPLC: t_R = 10.5 min, >99% purity (10–95% MeCN/H_2O, linear gradient, with constant 0.1% v/v TFA additive; 20 min run; 1 mL/min flow; ESI; positive ion mode; detection at 650 nm); HRMS (ESI) calcd for $C_{31}H_{35}N_2O_5Si$ [M+H]$^+$ 543.2310, found 543.2316.

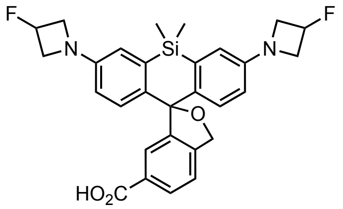

6-Carboxy-JF$_{635}$b (S21): The title compound (~100%, blue green solid) was prepared from 6-*tert*-butoxycarbonyl-JF$_{635}$b (**S12**) according to method S2. ^{1}H NMR ($CDCl_3$, 400 MHz) δ 8.02 (dd, J = 7.9, 1.5 Hz, 1H), 7.75 (dd, J = 1.4, 0.6 Hz, 1H), 7.38 (dd, J = 8.0, 0.6 Hz, 1H), 7.02 (d, J = 8.6 Hz, 2H), 6.69 (d, J = 2.6 Hz, 2H), 6.37 (dd, J = 8.6, 2.7 Hz, 2H), 5.40 (dtt, $^2J_{FH}$ = 57.1 Hz, J = 5.8, 3.8 Hz, 2H), 5.34 (s, 2H), 4.27 – 4.13 (m, 4H), 4.04 – 3.88 (m, 4H), 0.65 (s, 3H), 0.53 (s, 3H); ^{19}F NMR ($CDCl_3$, 376 MHz) δ -180.44 (dtt, J_{FH} = 56.9, 23.7, 18.0 Hz); Analytical HPLC: t_R = 12.2 min, >99% purity (10–95% MeCN/H_2O, linear gradient, with constant 0.1% v/v TFA additive; 20 min run; 1 mL/min flow; ESI; positive ion mode; detection at 650 nm); HRMS (ESI) calcd for $C_{29}H_{29}F_2N_2O_3Si$ $[M+H]^+$ 519.1910, found 519.1908.

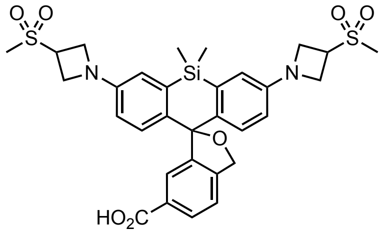

6-Carboxy-JF$_{630}$b (S22): The title compound (84%, blue solid) was prepared from 6-*tert*-butoxycarbonyl-JF$_{630}$b (**S13**) according to method S2. ^{1}H NMR ($CDCl_3$, 400 MHz) δ 8.01 (dd, J = 8.0, 1.5 Hz, 1H), 7.72 (d, J = 1.5 Hz, 1H), 7.38 (d, J = 8.0 Hz, 1H), 7.07 (d, J = 8.6 Hz, 2H), 6.69 (d, J = 2.7 Hz, 2H), 6.38 (dd, J = 8.6, 2.7 Hz, 2H), 5.37 (s, 2H), 4.26 – 4.14 (m, 8H), 4.12 – 4.02 (m, 2H), 2.98 (s, 6H), 0.65 (s, 3H), 0.52 (s, 3H); Analytical HPLC: t_R = 11.2 min, >99% purity (10–95% MeCN/H_2O, linear gradient, with constant 0.1% v/v TFA additive; 20 min run; 1 mL/min flow; ESI; positive ion mode; detection at 650 nm); HRMS (ESI) calcd for $C_{31}H_{35}N_2O_7S_2Si$ $[M+H]^+$ 639.1649, found 639.1644.

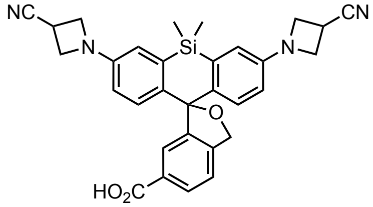

6-Carboxy-JF$_{629}$b (S23): The title compound (88%, pale blue solid) was prepared from 6-*tert*-butoxycarbonyl-JF$_{629}$b (**S14**) according to method S2. ^{1}H NMR (CD_3OD, 400 MHz) δ 7.91 (dd, J = 7.9, 1.5 Hz, 1H), 7.47 (d, J = 1.4 Hz, 1H), 7.44 (d, J = 8.0 Hz, 1H), 7.14 (d, J = 8.7 Hz, 2H), 6.74 (d, J = 2.6 Hz, 2H), 6.46 (dd, J = 8.7, 2.7 Hz, 2H), 5.49 (s, 2H), 4.18 – 4.12 (m, 4H), 4.04 – 3.98 (m, 4H), 3.70 (tt, J = 8.3, 5.6 Hz, 2H), 0.65 (s, 3H), 0.49 (s, 3H); Analytical HPLC: t_R = 12.4 min, >99% purity (10–95% MeCN/H_2O, linear gradient, with constant 0.1% v/v TFA additive; 20

min run; 1 mL/min flow; ESI; positive ion mode; detection at 650 nm); HRMS (ESI) calcd for $C_{31}H_{29}N_4O_3Si$ $[M+H]^+$ 533.2003, found 533.2007.

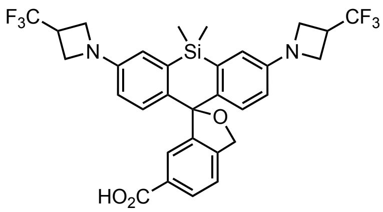

6-Carboxy-JF$_{626}$b (S24): The title compound (97%, blue solid) was prepared from 6-*tert*-butoxycarbonyl-JF$_{626}$b (**S15**) according to method S2. ^{1}H NMR ($CDCl_3$, 400 MHz) δ 8.02 (dd, J = 7.9, 1.5 Hz, 1H), 7.75 (d, J = 1.7 Hz, 1H), 7.38 (d, J = 7.9 Hz, 1H), 7.03 (d, J = 8.6 Hz, 2H), 6.67 (d, J = 2.7 Hz, 2H), 6.35 (dd, J = 8.7, 2.7 Hz, 2H), 5.34 (s, 2H), 4.11 – 4.00 (m, 4H), 3.99 – 3.91 (m, 4H), 3.44 – 3.31 (m, 2H), 0.64 (s, 3H), 0.52 (s, 3H); ^{19}F NMR ($CDCl_3$, 376 MHz) δ -73.31 (d, $^3J_{FH}$ = 8.5 Hz); ^{13}C NMR ($CDCl_3$, 101 MHz) δ 171.3 (C), 149.0 (C), 147.1 (C), 145.3 (C), 140.0 (C), 135.5 (C), 129.7 (CH), 129.1 (C), 128.2 (CH), 126.5 (q, $^1J_{CF}$ = 275.1 Hz, CF_3), 126.4 (CH), 121.6 (CH), 115.8 (CH), 112.8 (CH), 92.6 (C), 72.8 (CH_2), 51.4 (q, $^3J_{CF}$ = 3.2 Hz, CH_2), 33.2 (q, $^2J_{CF}$ = 32.0 Hz, CH), 0.5 (CH_3), -0.9 (CH_3); Analytical HPLC: t_R = 14.4 min, >99% purity (10–95% MeCN/H_2O, linear gradient, with constant 0.1% v/v TFA additive; 20 min run; 1 mL/min flow; ESI; positive ion mode; detection at 650 nm); HRMS (ESI) calcd for $C_{31}H_{29}F_6N_2O_3Si$ $[M+H]^+$ 619.1846, found 619.1849.

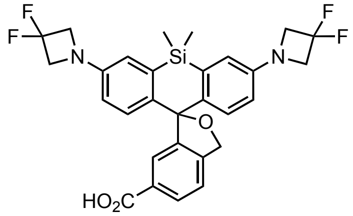

6-Carboxy-JF$_{614}$b (S25): The title compound (95%, blue solid) was prepared from 6-*tert*-butoxycarbonyl-JF$_{614}$b (**S16**) according to method S2. ^{1}H NMR ($CDCl_3$, 400 MHz) δ 8.03 (dd, J = 8.0, 1.5 Hz, 1H), 7.75 (d, J = 1.6 Hz, 1H), 7.39 (d, J = 8.0 Hz, 1H), 7.07 (d, J = 8.6 Hz, 2H), 6.71 (d, J = 2.7 Hz, 2H), 6.40 (dd, J = 8.6, 2.7 Hz, 2H), 5.36 (s, 2H), 4.22 (t, $^3J_{HF}$ = 11.8 Hz, 8H), 0.66 (s, 3H), 0.54 (s, 3H); ^{19}F NMR ($CDCl_3$, 376 MHz) δ -99.77 (p, $^3J_{FH}$ = 12.0 Hz); ^{13}C NMR ($CDCl_3$, 101 MHz) δ 171.4 (C), 148.2 (t, $^4J_{CF}$ = 2.5 Hz, C), 147.0 (C), 145.1 (C), 140.6 (C), 135.5 (C), 129.8 (CH), 129.2 (C), 128.3 (CH), 126.3 (CH), 121.7 (CH), 116.8 (CH), 116.1 (t, $^1J_{CF}$ = 274.7 Hz, CF_2), 113.9 (CH), 92.4 (C), 72.9 (CH_2), 63.5 (t, $^2J_{CF}$ = 25.7 Hz, CH_2), 0.5 (CH_3), -0.8 (CH_3); Analytical HPLC: t_R = 15.0 min, >99% purity (10–95% MeCN/H_2O, linear gradient, with constant 0.1% v/v TFA additive; 20 min run; 1 mL/min flow; ESI; positive ion mode; detection at 254 nm); HRMS (ESI) calcd for $C_{29}H_{27}F_4N_2O_3Si$ $[M+H]^+$ 555.1722, found 555.1734.

Synthesis of JFb–HaloTag Ligands and Other Derivatives

General method S3: Preparation of HaloTag ligands and maleimides. The following procedure for JF_{614}b–HaloTag ligand (**9_{HTL}**) is representative. 6-Carboxy-JF_{614}b (**S25**; 50 mg, 90.2 µmol) and HATU (51.4 mg, 0.135 mmol, 1.5 eq) were combined in DMF (4 mL); 2-(2-((6-chlorohexyl)oxy)ethoxy)ethanamine hydrochloride (**S26**; 35.2 mg, 0.135 mmol, 1.5 eq) and DIEA (47.1 µL, 0.270 mmol, 3 eq) were added, and the reaction was stirred at room temperature for 4 h. It was subsequently diluted with saturated $NaHCO_3$ and extracted with EtOAc (2×). The combined organic extracts were washed with brine, dried over anhydrous $MgSO_4$, filtered, and concentrated *in vacuo*. Silica gel chromatography (10–100% EtOAc/toluene, linear gradient) afforded 55.8 mg (81%) of **9_{HTL}** as a white solid.

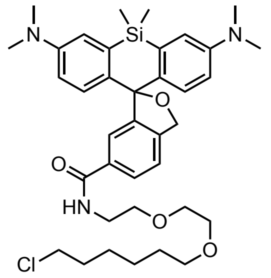

HM-SiR–HaloTag ligand (1_{HTL}): The title compound (52%, blue solid) was prepared from 6-carboxy-HM-SiR (**S17**) according to method S3. ^{1}H NMR ($CDCl_3$, 400 MHz) δ 7.75 (dd, *J* = 7.9, 1.6 Hz, 1H), 7.42 (dd, *J* = 1.7, 0.6 Hz, 1H), 7.35 (dd, *J* = 7.9, 0.6 Hz, 1H), 6.968 (d, *J* = 8.7 Hz, 2H), 6.967 (d, *J* = 2.9 Hz, 2H), 6.62 (dd, *J* = 8.9, 2.9 Hz, 2H), 6.58 (t, *J* = 4.7 Hz, 1H), 5.29 (s, 2H), 3.64 – 3.57 (m, 6H), 3.55 – 3.51 (m, 2H), 3.49 (t, *J* = 6.7 Hz, 2H), 3.37 (t, *J* = 6.6 Hz, 2H), 2.95 (s, 12H), 1.76 – 1.68 (m, 2H), 1.55 – 1.46 (m, 2H), 1.43 – 1.28 (m, 4H), 0.66 (s, 3H), 0.54 (s, 3H); Analytical HPLC: t_R = 11.8 min, 96.8% purity (10–95% MeCN/H_2O, linear gradient, with constant 0.1% v/v TFA additive; 20 min run; 1 mL/min flow; ESI; positive ion mode; detection at 650 nm); HRMS (ESI) calcd for $C_{37}H_{51}ClN_3O_4Si$ $[M+H]^+$ 664.3332, found 664.3326.

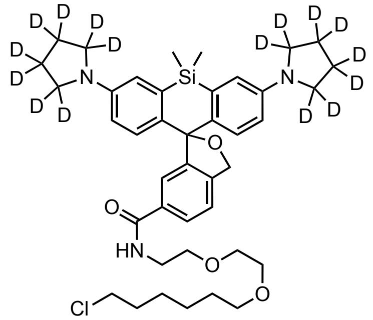

JFX_{650}b–HaloTag ligand (2_{HTL}): The title compound (63%, blue solid) was prepared from 6-carboxy-JFX_{650}b (**S18**) according to method S3. ^{1}H NMR ($CDCl_3$, 400 MHz) δ 7.75 (dd, *J* = 7.8, 1.6 Hz, 1H), 7.39 (d, *J* = 1.6 Hz, 1H), 7.34 (d, *J* = 7.9 Hz, 1H), 6.95 (d, *J* = 8.8 Hz, 2H), 6.77 (d, *J* = 2.8 Hz, 2H), 6.56 (t, *J* = 4.5 Hz, 1H), 6.45 (dd, *J* = 8.8, 2.8 Hz, 2H), 5.29 (s, 2H), 3.64 – 3.55 (m, 6H), 3.55 – 3.51 (m, 2H), 3.49 (t, *J* = 6.7 Hz, 2H), 3.37 (t, *J* = 6.6 Hz, 2H), 1.76 – 1.68 (m, 2H), 1.55 – 1.47 (m, 2H), 1.43 – 1.34 (m, 2H), 1.33 – 1.26 (m, 2H), 0.64 (s, 3H), 0.53 (s, 3H); Analytical

HPLC: t_R = 13.4 min, >99% purity (10–95% MeCN/H_2O, linear gradient, with constant 0.1% v/v TFA additive; 20 min run; 1 mL/min flow; ESI; positive ion mode; detection at 650 nm); HRMS (ESI) calcd for $C_{41}H_{39}D_{16}ClN_3O_4Si$ $[M+H]^+$ 732.4649, found 732.4638.

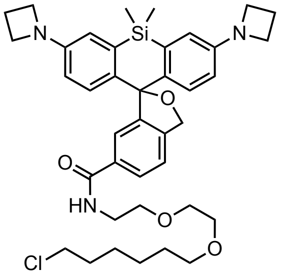

JF$_{646}$b–HaloTag ligand (3$_{HTL}$): The title compound (54%, blue solid) was prepared from 6-carboxy-JF$_{646}$b (**S19**) according to method S3. ^{1}H NMR ($CDCl_3$, 400 MHz) δ 7.75 (dd, *J* = 7.8, 1.5 Hz, 1H), 7.43 (d, *J* = 1.5 Hz, 1H), 7.35 (d, *J* = 7.9 Hz, 1H), 6.94 (d, *J* = 8.6 Hz, 2H), 6.66 (d, *J* = 2.6 Hz, 2H), 6.62 (t, *J* = 4.4 Hz, 1H), 6.32 (dd, *J* = 8.6, 2.7 Hz, 2H), 5.28 (s, 2H), 3.88 (t, *J* = 7.2 Hz, 8H), 3.66 – 3.55 (m, 6H), 3.56 – 3.52 (m, 2H), 3.50 (t, *J* = 6.7 Hz, 2H), 3.38 (t, *J* = 6.6 Hz, 2H), 2.35 (p, *J* = 7.2 Hz, 4H), 1.77 – 1.69 (m, 2H), 1.55 – 1.48 (m, 2H), 1.43 – 1.35 (m, 2H), 1.33 – 1.27 (m, 2H), 0.63 (s, 3H), 0.52 (s, 3H); Analytical HPLC: t_R = 12.8 min, >99% purity (10–95% MeCN/H_2O, linear gradient, with constant 0.1% v/v TFA additive; 20 min run; 1 mL/min flow; ESI; positive ion mode; detection at 650 nm); HRMS (ESI) calcd for $C_{39}H_{51}ClN_3O_4Si$ $[M+H]^+$ 688.3332, found 688.3330.

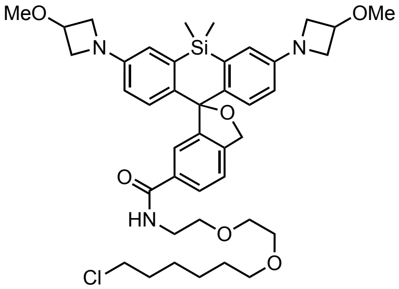

JF$_{639}$b–HaloTag ligand (4$_{HTL}$): The title compound (60%, pale blue solid) was prepared from 6-carboxy-JF$_{639}$b (**S20**) according to method S3. ^{1}H NMR ($CDCl_3$, 400 MHz) δ 7.62 (dd, *J* = 7.9, 1.5 Hz, 1H), 7.30 (d, *J* = 1.5 Hz, 1H), 7.21 (d, *J* = 7.9 Hz, 1H), 6.82 (d, *J* = 8.6 Hz, 2H), 6.55 (d, *J* = 2.6 Hz, 2H), 6.48 (t, *J* = 4.4 Hz, 1H), 6.21 (dd, *J* = 8.6, 2.7 Hz, 2H), 5.14 (s, 2H), 4.25 – 4.14 (m, 2H), 4.02 – 3.91 (m, 4H), 3.63 – 3.54 (m, 4H), 3.52 – 3.43 (m, 6H), 3.42 – 3.38 (m, 2H), 3.37 (t, *J* = 6.6 Hz, 2H), 3.24 (t, *J* = 6.6 Hz, 2H), 3.19 (s, 6H), 1.63 – 1.55 (m, 2H), 1.41 – 1.34 (m, 2H), 1.30 – 1.21 (m, 2H), 1.20 – 1.14 (m, 2H), 0.50 (s, 3H), 0.38 (s, 3H); Analytical HPLC: t_R = 12.6 min, >99% purity (10–95% MeCN/H_2O, linear gradient, with constant 0.1% v/v TFA additive; 20 min run; 1 mL/min flow; ESI; positive ion mode; detection at 650 nm); HRMS (ESI) calcd for $C_{41}H_{55}ClN_3O_6Si$ $[M+H]^+$ 748.3543, found 748.3548.

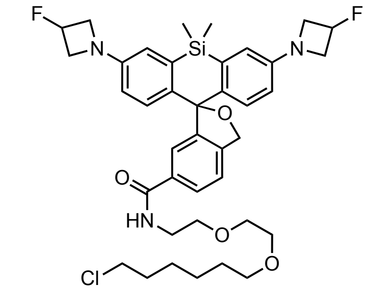

JF_{635}b–HaloTag ligand (5_{HTL}): The title compound (51%, pale blue solid) was prepared from 6-carboxy-JF_{635}b (**S21**) according to method S3. ^{1}H NMR ($CDCl_3$, 400 MHz) δ 7.66 (dd, J = 7.9, 1.5 Hz, 1H), 7.38 (d, J = 1.5 Hz, 1H), 7.28 (d, J = 7.9 Hz, 1H), 6.93 (d, J = 8.6 Hz, 2H), 6.62 (d, J = 2.7 Hz, 2H), 6.55 (t, J = 4.4 Hz, 1H), 6.29 (dd, J = 8.6, 2.7 Hz, 2H), 5.33 (dtt, $^{2}J_{HF}$ = 57.1 Hz, J = 5.9, 3.8 Hz, 2H), 5.23 (s, 2H), 4.19 – 4.05 (m, 4H), 3.96 – 3.82 (m, 4H), 3.58 – 3.50 (m, 6H), 3.49 – 3.45 (m, 2H), 3.43 (t, J = 6.7 Hz, 2H), 3.32 (t, J = 6.7 Hz, 2H), 1.70 – 1.61 (m, 2H), 1.48 – 1.41 (m, 2H), 1.36 – 1.28 (m, 2H), 1.27 – 1.20 (m, 2H), 0.58 (s, 3H), 0.46 (s, 3H); ^{19}F NMR ($CDCl_3$, 376 MHz) δ -180.43 (dtt, J_{FH} = 57.2, 24.0, 18.0 Hz); Analytical HPLC: t_R = 14.1 min, 98.1% purity (10–95% MeCN/H_2O, linear gradient, with constant 0.1% v/v TFA additive; 20 min run; 1 mL/min flow; ESI; positive ion mode; detection at 650 nm); HRMS (ESI) calcd for $C_{39}H_{49}ClF_2N_3O_4Si$ $[M+H]^+$ 724.3143, found 724.3141.

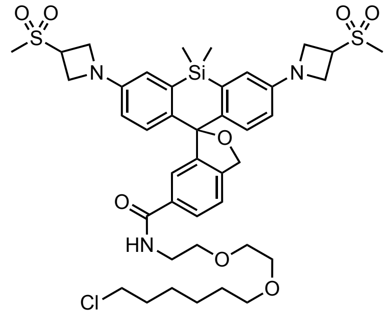

JF_{630}b–HaloTag ligand (6_{HTL}): The title compound (64%, pale blue solid) was prepared from 6-carboxy-JF_{630}b (**S22**) according to method S3. ^{1}H NMR ($CDCl_3$, 400 MHz) δ 7.58 (dd, J = 7.9, 1.5 Hz, 1H), 7.31 (d, J = 1.6 Hz, 1H), 7.22 (d, J = 7.9 Hz, 1H), 6.92 (d, J = 8.6 Hz, 2H), 6.56 (d, J = 2.7 Hz, 2H), 6.50 (t, J = 4.3 Hz, 1H), 6.24 (dd, J = 8.6, 2.7 Hz, 2H), 5.19 (s, 2H), 4.17 – 4.01 (m, 8H), 3.97 – 3.88 (m, 2H), 3.51 – 3.43 (m, 6H), 3.43 – 3.39 (m, 2H), 3.37 (t, J = 6.7 Hz, 2H), 3.26 (t, J = 6.6 Hz, 2H), 2.86 (s, 6H), 1.63 – 1.55 (m, 2H), 1.41 – 1.35 (m, 2H), 1.30 – 1.23 (m, 2H), 1.21 – 1.15 (m, 2H), 0.52 (s, 3H), 0.39 (s, 3H); Analytical HPLC: t_R = 12.7 min, >99% purity (10–95% MeCN/H_2O, linear gradient, with constant 0.1% v/v TFA additive; 20 min run; 1 mL/min flow; ESI; positive ion mode; detection at 650 nm); HRMS (ESI) calcd for $C_{41}H_{55}ClN_3O_8S_2SiNa$ $[M+Na]^+$ 866.2702, found 866.2715.

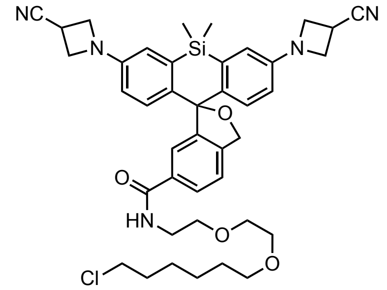

JF$_{629}$b–HaloTag ligand (7$_{HTL}$): The title compound (57%, pale blue solid) was prepared from 6-carboxy-JF$_{629}$b (**S23**) according to method S3. ^{1}H NMR ($CDCl_3$, 400 MHz) δ 7.70 (dd, *J* = 7.9, 1.5 Hz, 1H), 7.46 (d, *J* = 1.5 Hz, 1H), 7.36 (d, *J* = 7.9 Hz, 1H), 7.07 (d, *J* = 8.7 Hz, 2H), 6.71 (d, *J* = 2.6 Hz, 2H), 6.56 (t, *J* = 4.5 Hz, 1H), 6.40 (dd, *J* = 8.7, 2.7 Hz, 2H), 5.33 (s, 2H), 4.23 (ddd, *J* = 8.4, 7.1, 2.6 Hz, 4H), 4.10 (td, *J* = 6.8, 2.7 Hz, 4H), 3.65 – 3.53 (m, 10H), 3.50 (t, *J* = 6.7 Hz, 2H), 3.40 (t, *J* = 6.7 Hz, 2H), 1.77 – 1.68 (m, 2H), 1.57 – 1.49 (m, 2H), 1.44 – 1.35 (m, 2H), 1.35 – 1.25 (m, 2H), 0.66 (s, 3H), 0.53 (s, 3H); Analytical HPLC: t_R = 14.2 min, >99% purity (10–95% MeCN/H_2O, linear gradient, with constant 0.1% v/v TFA additive; 20 min run; 1 mL/min flow; ESI; positive ion mode; detection at 650 nm); HRMS (ESI) calcd for $C_{41}H_{49}ClN_5O_4Si$ $[M+H]^+$ 738.3237, found 738.3233.

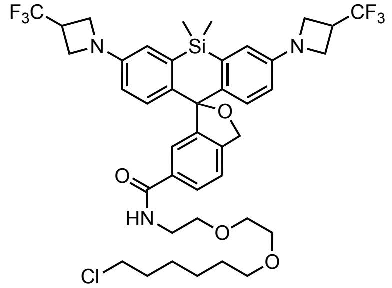

JF$_{626}$b–HaloTag ligand (8$_{HTL}$): The title compound (75%, white solid) was prepared from 6-carboxy-JF$_{626}$b (**S24**) according to method S3. ^{1}H NMR ($CDCl_3$, 400 MHz) δ 7.73 (dd, *J* = 7.9, 1.6 Hz, 1H), 7.45 (d, *J* = 1.7 Hz, 1H), 7.35 (d, *J* = 7.9 Hz, 1H), 7.00 (d, *J* = 8.6 Hz, 2H), 6.67 (d, *J* = 2.7 Hz, 2H), 6.60 (t, *J* = 4.8 Hz, 1H), 6.34 (dd, *J* = 8.7, 2.7 Hz, 2H), 5.29 (s, 2H), 4.09 – 4.02 (m, 4H), 3.99 – 3.91 (m, 4H), 3.65 – 3.57 (m, 6H), 3.56 – 3.52 (m, 2H), 3.49 (t, *J* = 6.7 Hz, 2H), 3.44 – 3.32 (m, 2H), 3.38 (t, *J* = 6.6 Hz, 2H), 1.77 – 1.68 (m, 2H), 1.54 – 1.48 (m, 2H), 1.43 – 1.35 (m, 2H), 1.33 – 1.25 (m, 2H), 0.64 (s, 3H), 0.52 (s, 3H); ^{19}F NMR ($CDCl_3$, 376 MHz) δ -73.30 (d, $^{3}J_{FH}$ = 8.6 Hz); Analytical HPLC: t_R = 15.9 min, >99% purity (10–95% MeCN/H_2O, linear gradient, with constant 0.1% v/v TFA additive; 20 min run; 1 mL/min flow; ESI; positive ion mode; detection at 650 nm); HRMS (ESI) calcd for $C_{41}H_{48}ClF_6N_3O_4SiNa$ $[M+Na]^+$ 846.2899, found 846.2908.

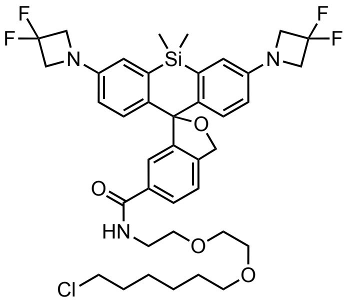

JF_{614}b–HaloTag ligand (9_{HTL}): The title compound (81%, white solid) was prepared from 6-carboxy-JF_{614}b (**S25**) according to method S3. ^{1}H NMR ($CDCl_3$, 400 MHz) δ 7.72 (dd, J = 7.9, 1.6 Hz, 1H), 7.46 (d, J = 1.5 Hz, 1H), 7.36 (d, J = 7.9 Hz, 1H), 7.05 (d, J = 8.6 Hz, 2H), 6.71 (d, J = 2.7 Hz, 2H), 6.61 (t, J = 4.8 Hz, 1H), 6.39 (dd, J = 8.6, 2.7 Hz, 2H), 5.32 (s, 2H), 4.22 (t, $^3J_{HF}$ = 11.8 Hz, 8H), 3.65 – 3.57 (m, 6H), 3.57 – 3.52 (m, 2H), 3.50 (t, J = 6.7 Hz, 2H), 3.39 (t, J = 6.6 Hz, 2H), 1.77 – 1.68 (m, 2H), 1.56 – 1.48 (m, 2H), 1.44 – 1.35 (m, 2H), 1.34 – 1.27 (m, 2H), 0.66 (s, 3H), 0.54 (s, 3H); ^{19}F NMR ($CDCl_3$, 376 MHz) δ -99.79 (p, $^3J_{FH}$ = 11.8 Hz); Analytical HPLC: t_R = 15.0 min, >99% purity (30–95% MeCN/H_2O, linear gradient, with constant 0.1% v/v TFA additive; 20 min run; 1 mL/min flow; ESI; positive ion mode; detection at 254 nm); HRMS (ESI) calcd for $C_{39}H_{46}ClF_4N_3O_4SiNa$ $[M+Na]^+$ 782.2774, found 782.2784.

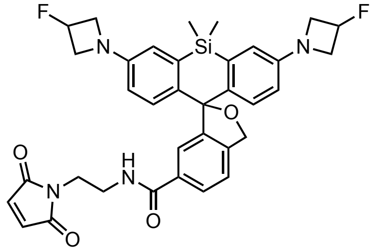

JF_{635}b–maleimide (5_{MAL}): The title compound (83%, yellow solid) was prepared from 6-carboxy-JF_{635}b (**S21**) and *N*-(2-aminoethyl)maleimide trifluoroacetate salt according to method S3. ^{1}H NMR ($CDCl_3$, 400 MHz) δ 7.66 (dd, J = 7.9, 1.6 Hz, 1H), 7.47 (dd, J = 1.6, 0.6 Hz, 1H), 7.35 (dd, J = 7.9, 0.5 Hz, 1H), 6.98 (d, J = 8.6 Hz, 2H), 6.69 (d, J = 2.6 Hz, 2H), 6.68 (s, 2H), 6.55 (t, J = 5.4 Hz, 1H), 6.35 (dd, J = 8.6, 2.6 Hz, 2H), 5.40 (dtt, $^2J_{HF}$ = 57.1 Hz, J = 5.9, 3.7 Hz, 2H), 5.27 (s, 2H), 4.24 – 4.13 (m, 4H), 4.01 – 3.89 (m, 4H), 3.80 – 3.74 (m, 2H), 3.64 – 3.57 (m, 2H), 0.64 (s, 3H), 0.53 (s, 3H); ^{19}F NMR ($CDCl_3$, 376 MHz) δ -180.43 (dtt, J_{FH} = 57.0, 24.2, 18.1 Hz); ^{13}C NMR ($CDCl_3$, 101 MHz) δ 171.1 (C), 167.5 (C), 149.5 (d, $^4J_{CF}$ = 1.1 Hz, C), 146.9 (C), 143.2 (C), 139.7 (C), 135.6 (C), 134.4 (CH), 133.9 (C), 128.3 (CH), 126.2 (CH), 123.6 (CH), 121.6 (CH), 116.3 (CH), 113.3 (CH), 92.6 (C), 82.9 (d, $^1J_{CF}$ = 204.5 Hz, CHF), 72.5 (CH_2), 59.7 (d, $^2J_{CF}$ = 23.5 Hz, CH_2), 39.8 (CH_2), 37.5 (CH_2), 0.6 (CH_3), -1.0 (CH_3); Analytical HPLC: t_R = 11.9 min, >99% purity (10–95% MeCN/H_2O, linear gradient, with constant 0.1% v/v TFA additive; 20 min run; 1 mL/min flow; ESI; positive ion mode; detection at 650 nm); HRMS (ESI) calcd for $C_{35}H_{35}F_2N_4O_4Si$ $[M+H]^+$ 641.2390, found 641.2385.

General method S4: NHS ester formation. The following procedure for JF_{630}b-NHS (**6_{NHS}**) is representative. 6-Carboxy-JF_{630}b (**S22**; 30 mg, 47.0 μmol) was combined with DSC (28.9 mg, 0.113 mmol, 2.4 eq) in DMF (1.5 mL).

After adding Et_3N (39.3 μL, 0.282 mmol, 6 eq) and DMAP (0.6 mg, 4.7 μmol, 0.1 eq), the reaction was stirred at room temperature for 1 h. It was subsequently concentrated *in vacuo* and purified by reverse phase HPLC (20–60% $MeCN/H_2O$, linear gradient, with constant 0.1% TFA additive). The pooled product fractions were partially concentrated to remove MeCN, neutralized with 1–2 mL saturated $NaHCO_3$, and extracted with CH_2Cl_2 (2×). The combined organic layers were washed with brine, dried over anhydrous $MgSO_4$, filtered, and evaporated to yield 17.8 mg (52%) of **6$_{NHS}$** as a pale blue solid.

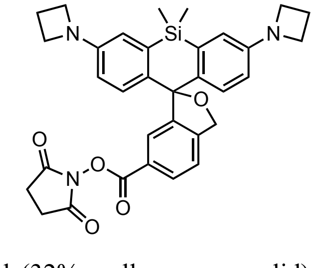

JF$_{646}$b–NHS (3$_{NHS}$): The title compound (32%, yellow-green solid) was prepared from 6-carboxy-JF$_{646}$b (**S19**) according to method S4. ^{1}H NMR ($CDCl_3$, 400 MHz) δ 7.88 (dd, *J* = 7.9, 1.5 Hz, 1H), 7.59 (d, *J* = 1.5 Hz, 1H), 7.24 (d, *J* = 8.0 Hz, 1H), 6.78 (d, *J* = 8.6 Hz, 2H), 6.47 (d, *J* = 2.6 Hz, 2H), 6.17 (dd, *J* = 8.6, 2.7 Hz, 2H), 5.17 (s, 2H), 3.71 (t, *J* = 7.2 Hz, 8H), 2.74 – 2.63 (m, 4H), 2.16 (p, *J* = 7.3 Hz, 4H), 0.43 (s, 3H), 0.32 (s, 3H); ^{13}C NMR ($CDCl_3$, 101 MHz) δ 169.4 (C), 161.8 (C), 150.7 (C), 148.1 (C), 146.8 (C), 138.5 (C), 135.0 (C), 129.8 (CH), 128.1 (CH), 126.9 (CH), 124.7 (C), 121.9 (CH), 115.8 (CH), 112.9 (CH), 92.8 (C), 72.7 (CH_2), 52.6 (CH_2), 25.8 (CH_2), 17.1 (CH_2), 0.5 (CH_3), -0.8 (CH_3); Analytical HPLC: t_R = 11.6 min, 96.5% purity (10–95% $MeCN/H_2O$, linear gradient, with constant 0.1% v/v TFA additive; 20 min run; 1 mL/min flow; ESI; positive ion mode; detection at 650 nm); HRMS (ESI) calcd for $C_{33}H_{34}N_3O_5Si$ $[M+H]^+$ 580.2262, found 580.2260.

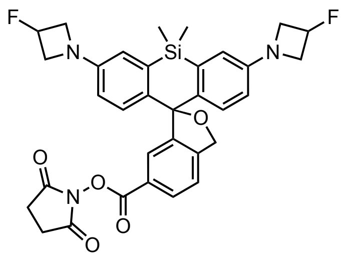

JF$_{635}$b–NHS (5$_{NHS}$): The title compound (56%, off-white solid) was prepared from 6-carboxy-JF$_{635}$b (**S21**) according to method S4. ^{1}H NMR ($CDCl_3$, 400 MHz) δ 8.10 (dd, *J* = 8.0, 1.6 Hz, 1H), 7.79 (d, *J* = 1.5 Hz, 1H), 7.46 (d, *J* = 8.0 Hz, 1H), 7.04 (d, *J* = 8.7 Hz, 2H), 6.71 (d, *J* = 2.7 Hz, 2H), 6.42 (dd, *J* = 8.7, 2.7 Hz, 2H), 5.43 (dtt, $^2J_{HF}$ = 57.1 Hz, *J* = 6.0, 3.7 Hz, 1H), 5.39 (s, 2H), 5.38 – 5.33 (m, 1H), 4.28 – 4.16 (m, 4H), 4.06 – 3.94 (m, 4H), 2.90 (s, 4H), 0.65 (s, 3H), 0.55 (s, 3H); ^{19}F NMR ($CDCl_3$, 376 MHz) δ -180.53 (dtt, J_{FH} = 57.4, 24.1, 18.1 Hz); ^{13}C NMR ($CDCl_3$, 101 MHz) δ 169.3 (C), 161.7 (C), 149.6 (d, $^4J_{CF}$ = 1.2 Hz, C), 147.9 (C), 146.5 (C), 139.4 (C), 135.2 (C), 129.9 (CH), 128.2 (CH), 126.8 (CH), 124.8 (C), 122.1 (CH), 116.3 (CH), 113.5 (CH), 92.6 (C), 83.0 (d, $^1J_{CF}$ = 204.5 Hz, CHF), 72.9 (CH_2), 59.7 (d, $^2J_{CF}$ = 23.6 Hz, CH_2), 25.8 (CH_2), 0.5 (CH_3), -0.8 (CH_3); Analytical HPLC: t_R = 12.6 min, >99%

purity (10–95% MeCN/H_2O, linear gradient, with constant 0.1% v/v TFA additive; 20 min run; 1 mL/min flow; ESI; positive ion mode; detection at 650 nm); HRMS (ESI) calcd for $C_{33}H_{32}F_2N_3O_5Si$ $[M+H]^+$ 616.2074, found 616.2073.

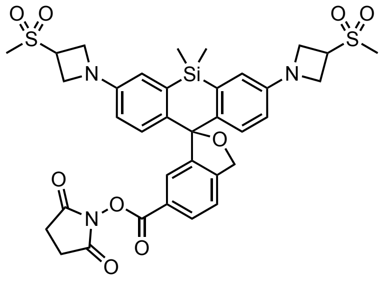

JF$_{630}$b–NHS (6$_{NHS}$): The title compound (52%, pale blue solid) was prepared from 6-carboxy-JF$_{630}$b (**S22**) according to method S4. ^{1}H NMR ($CDCl_3$, 400 MHz) δ 8.07 (dd, *J* = 8.0, 1.5 Hz, 1H), 7.74 (dd, *J* = 1.6, 0.7 Hz, 1H), 7.44 (dd, *J* = 8.0, 0.6 Hz, 1H), 7.06 (d, *J* = 8.6 Hz, 2H), 6.69 (d, *J* = 2.6 Hz, 2H), 6.41 (dd, *J* = 8.7, 2.7 Hz, 2H), 5.39 (s, 2H), 4.27 – 4.17 (m, 8H), 4.09 – 4.01 (m, 2H), 3.00 (s, 6H), 2.87 (s, 4H), 0.63 (s, 3H), 0.51 (s, 3H); ^{13}C NMR ($CDCl_3$, 101 MHz) δ 169.4 (C), 161.7 (C), 148.7 (C), 147.7 (C), 146.2 (C), 140.3 (C), 135.1 (C), 130.0 (CH), 128.2 (CH), 126.6 (CH), 124.8 (C), 122.2 (CH), 116.2 (CH), 113.4 (CH), 92.5 (C), 73.0 (CH_2), 52.7 (CH_2), 52.0 (CH), 37.9 (CH_3), 25.8 (CH_2), 0.4 (CH_3), -0.7 (CH_3); Analytical HPLC: t_R = 12.1 min, 98.3% purity (10–95% MeCN/H_2O, linear gradient, with constant 0.1% v/v TFA additive; 20 min run; 1 mL/min flow; ESI; positive ion mode; detection at 650 nm); HRMS (ESI) calcd for $C_{35}H_{38}N_3O_9S_2Si$ $[M+H]^+$ 736.1813, found 736.1814.

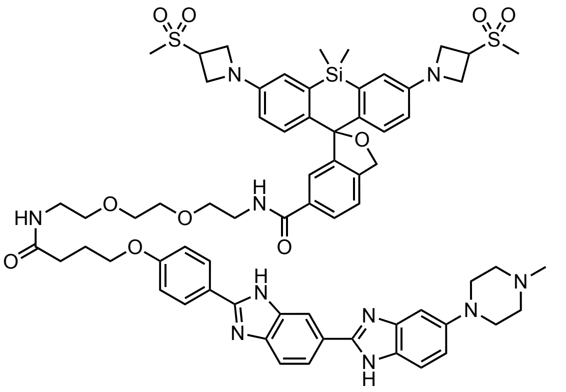

JF$_{630}$b–Hoechst (6$_{HST}$): JF$_{630}$b-NHS (**6$_{NHS}$**; 20 mg, 27.1 μmol), *N*-(2-(2-(2-aminoethoxy)ethoxy)ethyl)-4-(4-(5-(4-methylpiperazin-1-yl)-1*H*,3'*H*-[2,5'-bibenzo[*d*]imidazol]-2'-yl)phenoxy)butanamide[1] (4·TFA salt; 35.8 mg, 32.6 μmol, 1.2 eq), and DIEA (47.3 μL, 0.272 mmol, 10 eq) were combined in DMF (1 mL). The reaction was stirred for 4 h at room temperature, then concentrated *in vacuo*. The crude residue was purified by reverse phase HPLC (10–75% MeCN/H_2O, linear gradient, with constant 0.1% TFA additive). The pooled product fractions were partially concentrated to remove MeCN, neutralized with saturated $NaHCO_3$, and extracted with 10% MeOH/CH_2Cl_2 (2×). The combined organic layers were dried over anhydrous $MgSO_4$, filtered, and evaporated to yield 5.0 mg (15%) of **6$_{HST}$** as a yellow solid. ^{1}H NMR (CD_3OD, 400 MHz) δ 8.26 (s, 1H), 8.04 (d, *J* = 8.9 Hz, 2H), 7.96 (d, *J* = 8.4 Hz, 1H),

[1] Nakamura, A.; Takigawa, K.; Kurishita, Y.; Kuwata, K.; Ishida, M.; Shimoda, Y.; Hamachi, I.; Tsukiji, S. *Chem. Commun.* **2014**, *50*, 6149–6152.

7.70 (dd, J = 8.0, 1.6 Hz, 1H), 769 (bs, 1H), 7.52 (d, J = 8.8 Hz, 1H), 7.39 (d, J = 8.0 Hz, 1H), 7.33 (d, J = 1.5 Hz, 1H), 7.19 – 7.13 (m, 1H), 7.09 – 7.05 (m, 1H), 7.06 (d, J = 8.9 Hz, 2H), 7.03 (d, J = 8.7 Hz, 2H), 6.72 (d, J = 2.6 Hz, 2H), 6.41 (dd, J = 8.7, 2.7 Hz, 2H), 5.37 (s, 2H), 4.32 – 4.24 (m, 2H), 4.19 – 4.08 (m, 8H), 4.01 (t, J = 6.1 Hz, 2H), 3.58 – 3.49 (m, 6H), 3.48 – 3.41 (m, 4H), 3.29 – 3.23 (m, 6H), 2.98 (s, 6H), 2.81 – 2.72 (m, 4H), 2.44 (s, 3H), 2.34 (t, J = 7.3 Hz, 2H), 2.04 (p, J = 6.7 Hz, 2H), 0.61 (s, 3H), 0.46 (s, 3H); Analytical HPLC: t_R = 7.9 min, >99% purity (10–95% MeCN/H_2O, linear gradient, with constant 0.1% v/v TFA additive; 20 min run; 1 mL/min flow; ESI; positive ion mode; detection at 650 nm); HRMS (ESI) calcd for $C_{66}H_{77}N_{10}O_{10}S_2Si$ $[M+H]^+$ 1261.5029, found 1261.5026.

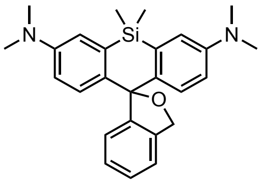

1

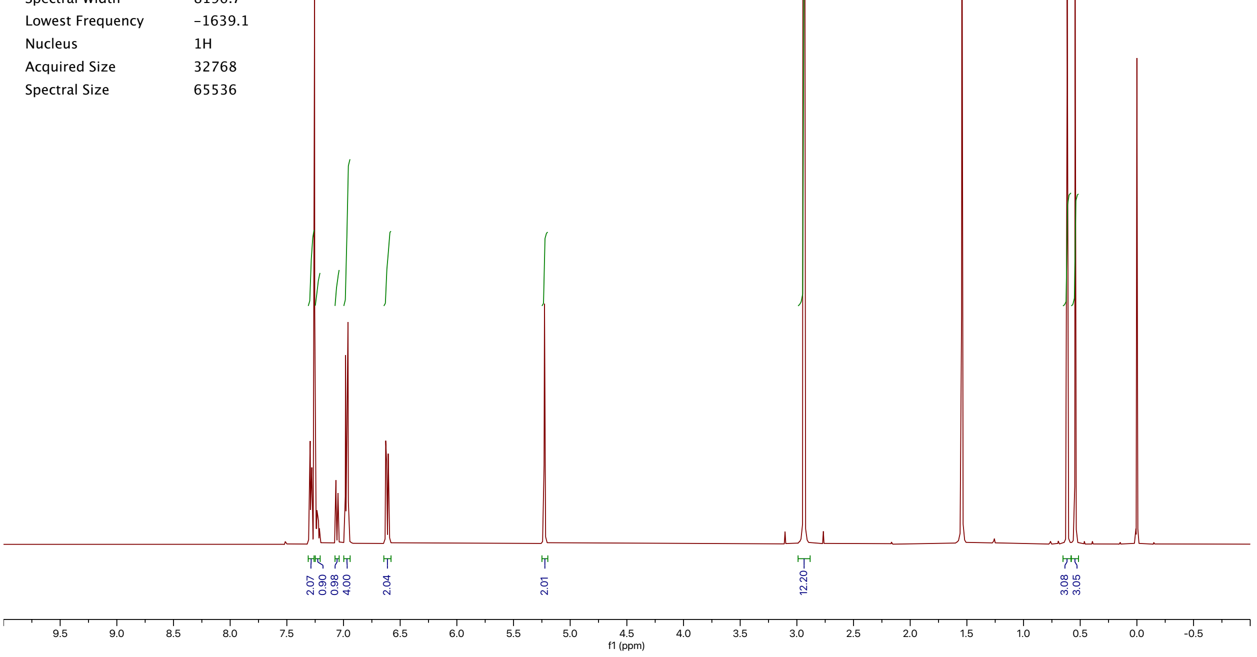

Origin Bruker BioSpin GmbH
Solvent CDCl3
Temperature 303.0
Pulse Sequence zg30
Experiment 1D
Number of Scans 16
Acquisition Date 2024-05-31T10:57:47
Spectrometer Frequency 400.13
Spectral Width 8196.7
Lowest Frequency -1639.1
Nucleus 1H
Acquired Size 32768
Spectral Size 65536
2.07
0.90
0.98
4.00
2.04
2.01
12.20
3.08
3.05
9.5 9.0 8.5 8.0 7.5 7.0 6.5 6.0 5.5 5.0 4.5 4.0 3.5 3.0 2.5 2.0 1.5 1.0 0.5 0.0 -0.5
f1 (ppm)

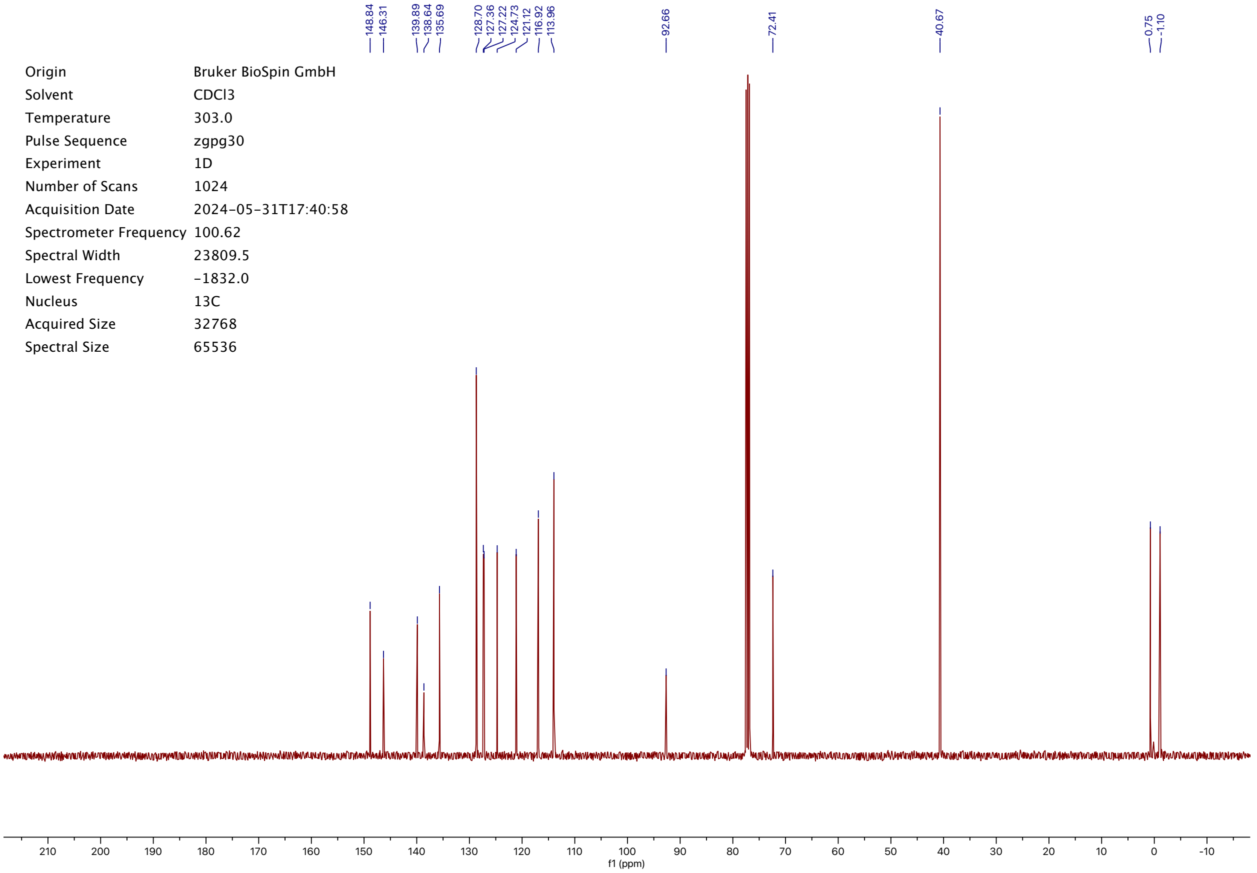

148.84
146.31
139.89
138.64
135.69
128.70
127.36
127.22
124.73
121.12
116.92
113.96
92.66
72.41
40.67
0.75
-1.10
Origin Bruker BioSpin GmbH
Solvent CDCl3
Temperature 303.0
Pulse Sequence zgpg30
Experiment 1D
Number of Scans 1024
Acquisition Date 2024-05-31T17:40:58
Spectrometer Frequency 100.62
Spectral Width 23809.5
Lowest Frequency -1832.0
Nucleus 13C
Acquired Size 32768
Spectral Size 65536
210 200 190 180 170 160 150 140 130 120 110 100 90 80 70 60 50 40 30 20 10 0 -10
f1 (ppm)

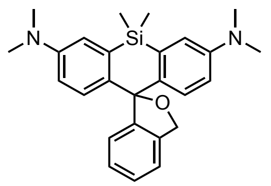

1

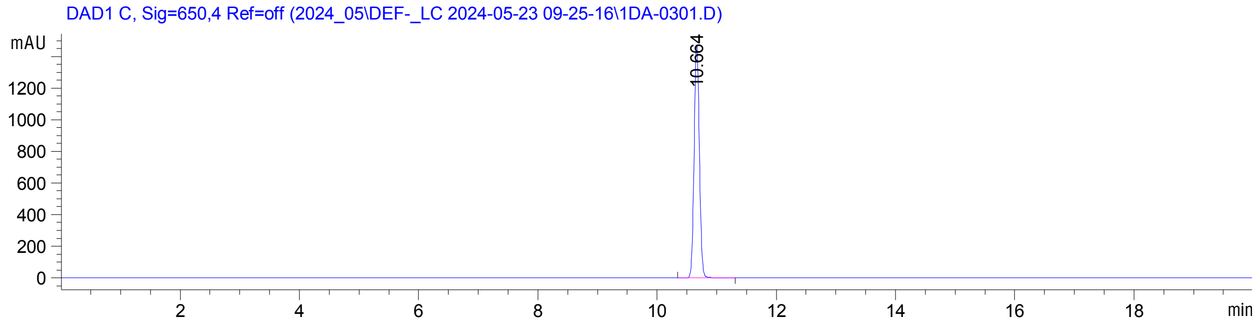

DAD1 C, Sig=650,4 Ref=off (2024_05\DEF-_LC 2024-05-23 09-25-16\1DA-0301.D)
mAU
10.664
min

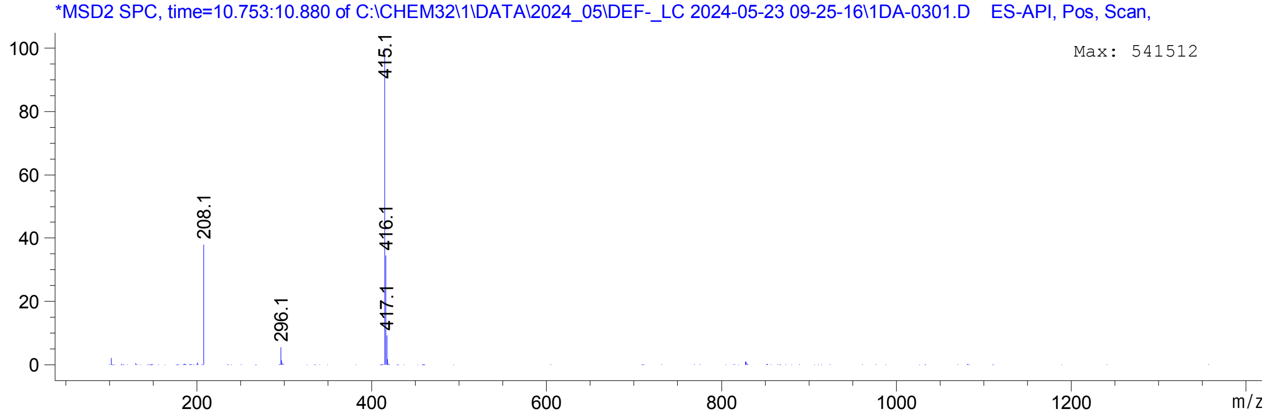

*MSD2 SPC, time=10.753:10.880 of C:\CHEM32\1\DATA\2024_05\DEF-_LC 2024-05-23 09-25-16\1DA-0301.D ES-API, Pos, Scan,
Max: 541512
415.1
416.1
417.1
208.1
296.1
m/z

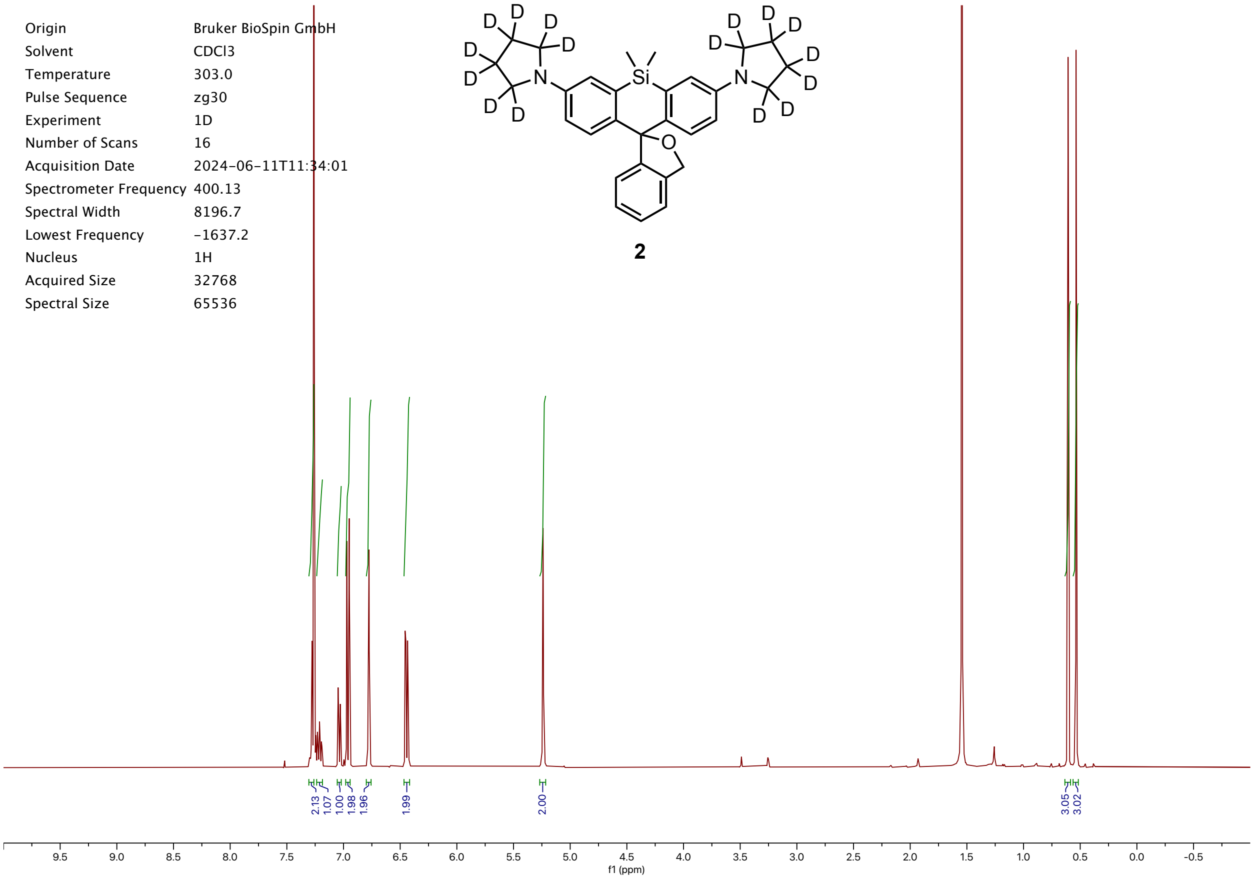
Origin Bruker BioSpin GmbH
Solvent CDCl3
Temperature 303.0
Pulse Sequence zg30
Experiment 1D
Number of Scans 16
Acquisition Date 2024-06-11T11:34:01
Spectrometer Frequency 400.13
Spectral Width 8196.7
Lowest Frequency -1637.2
Nucleus 1H
Acquired Size 32768
Spectral Size 65536
2
2.13 1.07 1.00 1.98 1.96 1.99 2.00 3.05 3.02
f1 (ppm)

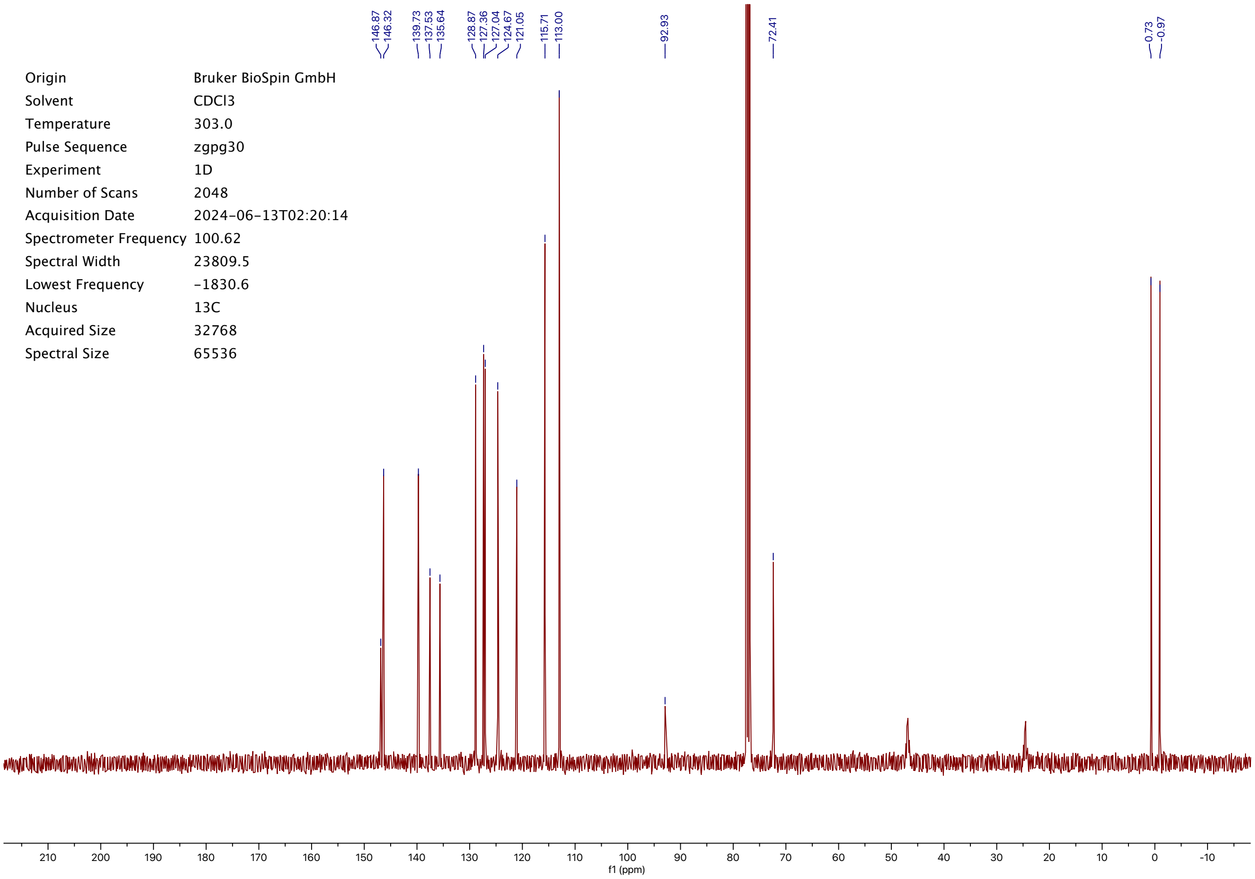
146.87 146.32 139.73 137.53 135.64 128.87 127.36 127.04 124.67 121.05 115.71 113.00 92.93 72.41 0.73 -0.97
Origin Bruker BioSpin GmbH
Solvent CDCl3
Temperature 303.0
Pulse Sequence zgpg30
Experiment 1D
Number of Scans 2048
Acquisition Date 2024-06-13T02:20:14
Spectrometer Frequency 100.62
Spectral Width 23809.5
Lowest Frequency -1830.6
Nucleus 13C
Acquired Size 32768
Spectral Size 65536
f1 (ppm)

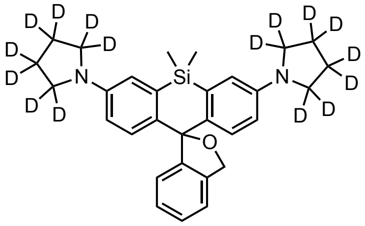

2

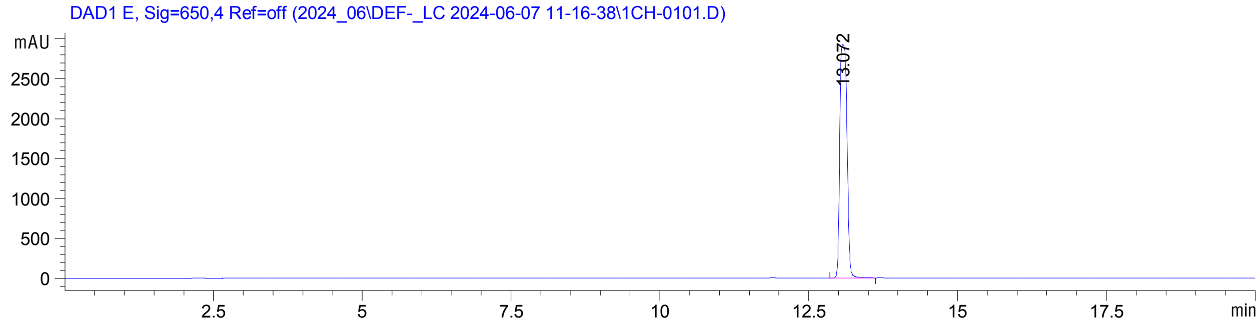
DAD1 E, Sig=650,4 Ref=off (2024_06\DEF-_LC 2024-06-07 11-16-38\1CH-0101.D)
mAU
2500
2000
1500
1000
500
0
13.072
2.5
5
7.5
10
12.5
15
17.5
min

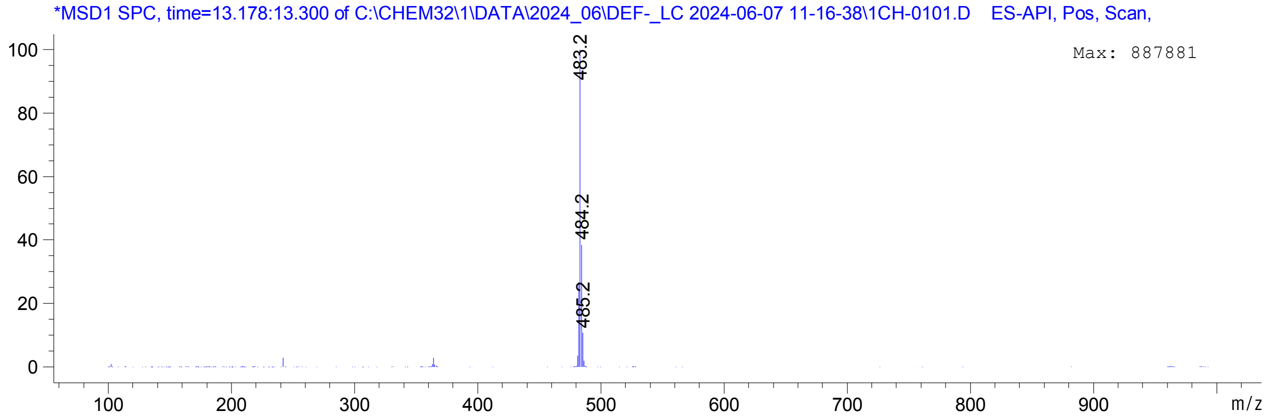
*MSD1 SPC, time=13.178:13.300 of C:\CHEM32\1\DATA\2024_06\DEF-_LC 2024-06-07 11-16-38\1CH-0101.D ES-API, Pos, Scan,
Max: 887881
100
80
60
40
20
0
483.2
484.2
485.2
100
200
300
400
500
600
700
800
900
m/z

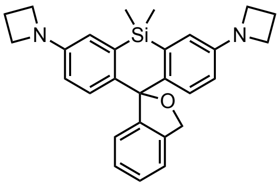

3

Origin	Bruker BioSpin GmbH
Solvent	CDCl3
Temperature	303.0
Pulse Sequence	zg30
Experiment	1D
Number of Scans	16
Acquisition Date	2024-06-11T11:28:51
Spectrometer Frequency	400.13
Spectral Width	8196.7
Lowest Frequency	-1637.2
Nucleus	1H
Acquired Size	32768
Spectral Size	65536

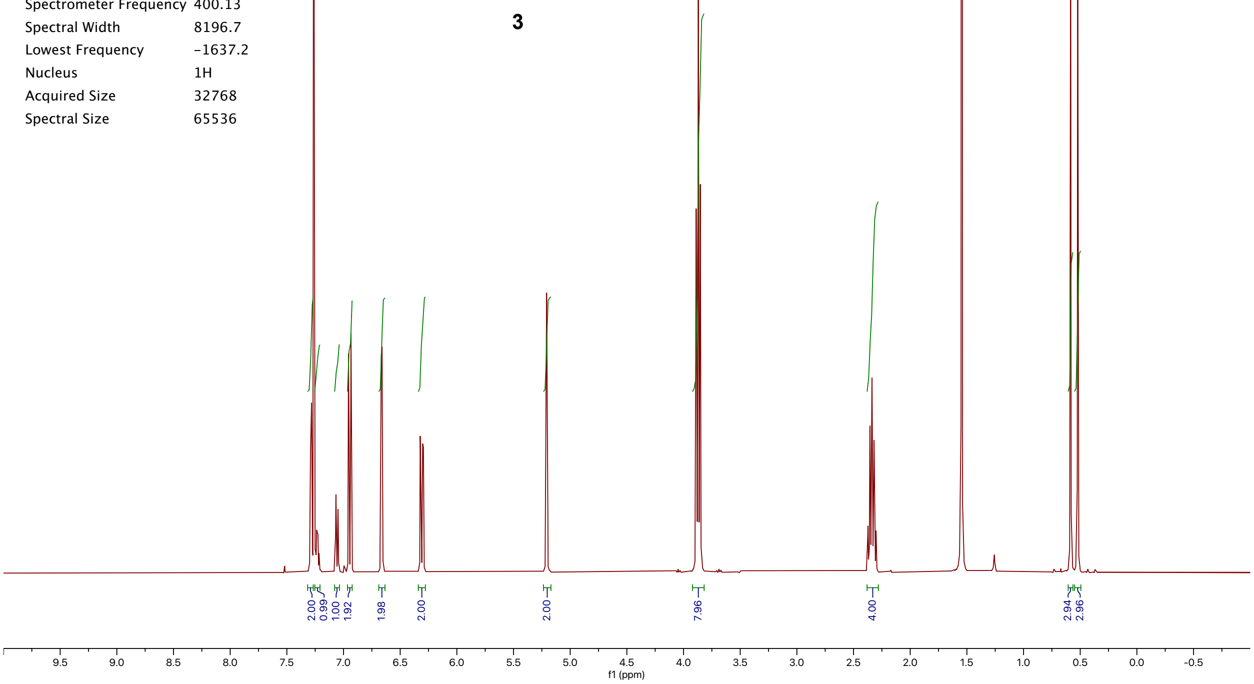

Origin	Bruker BioSpin GmbH
Solvent	CDCl3
Temperature	303.0
Pulse Sequence	zgpg30
Experiment	1D
Number of Scans	2048
Acquisition Date	2024-06-12T23:43:25
Spectrometer Frequency	100.62
Spectral Width	23809.5
Lowest Frequency	-1830.9
Nucleus	13C
Acquired Size	32768
Spectral Size	65536

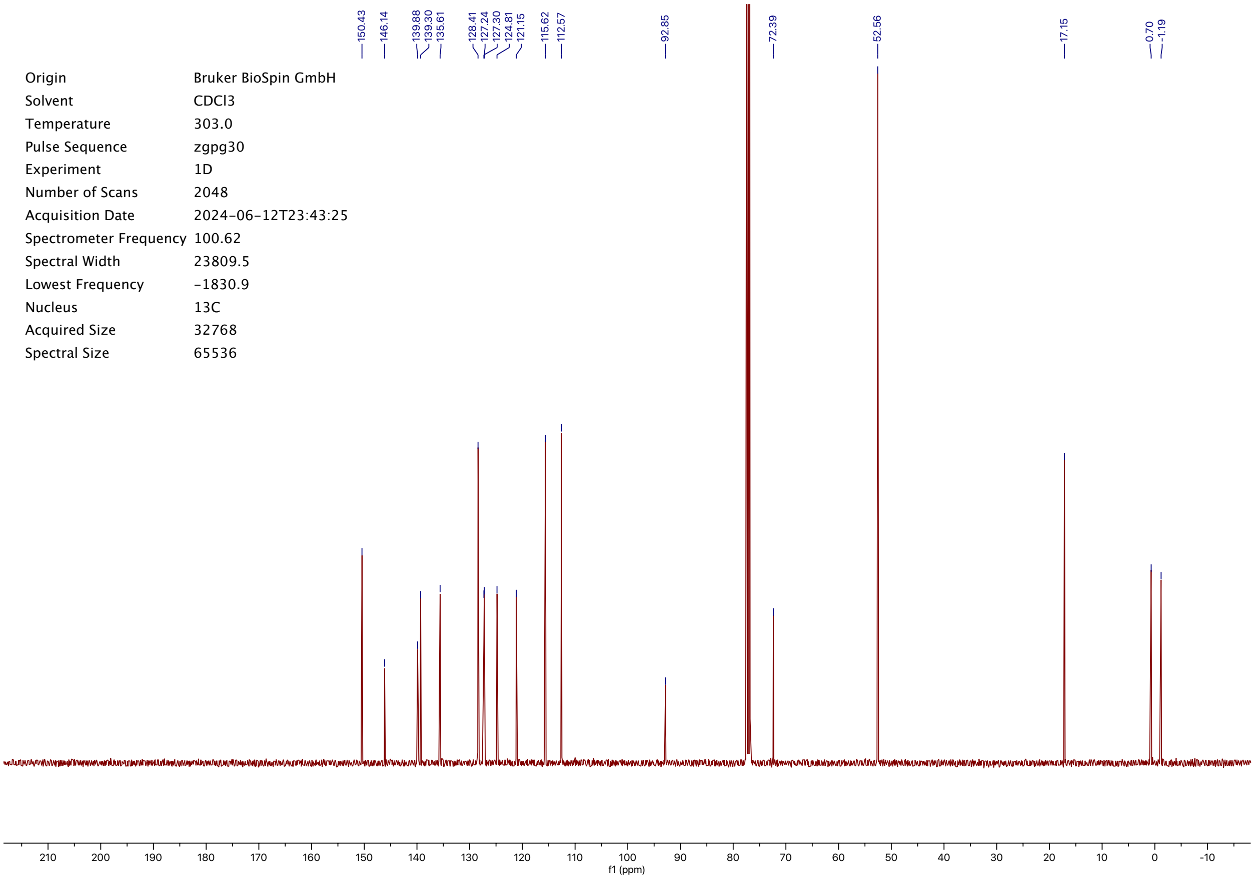

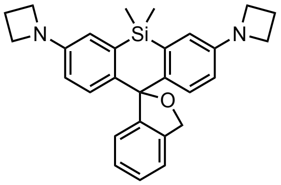

3

DAD1 E, Sig=650,4 Ref=off (2024_06\DEF-_LC 2024-06-06 11-02-42\1CF-0101.D)

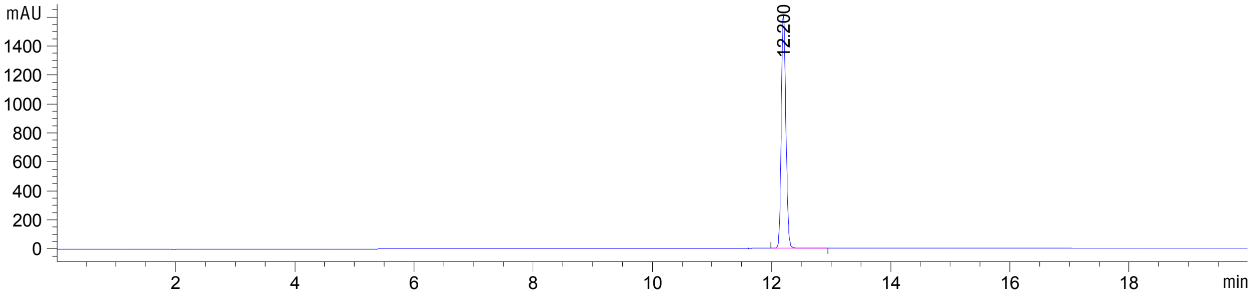

*MSD1 SPC, time=12.307:12.428 of C:\CHEM32\1\DATA\2024_06\DEF-_LC 2024-06-06 11-02-42\1CF-0101.D ES-API, Pos, Scan,

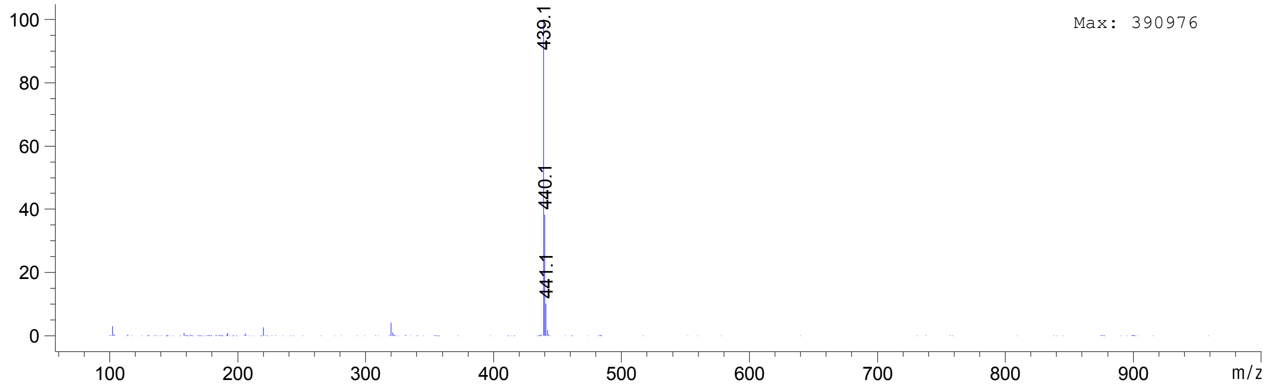

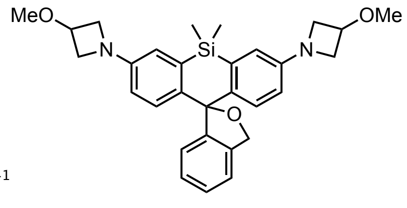

4

Origin	Bruker BioSpin GmbH
Solvent	CDCl3
Temperature	303.0
Pulse Sequence	zg30
Experiment	1D
Number of Scans	16
Acquisition Date	2024-06-11T11:23:41
Spectrometer Frequency	400.13
Spectral Width	8196.7
Lowest Frequency	-1637.1
Nucleus	1H
Acquired Size	32768
Spectral Size	65536

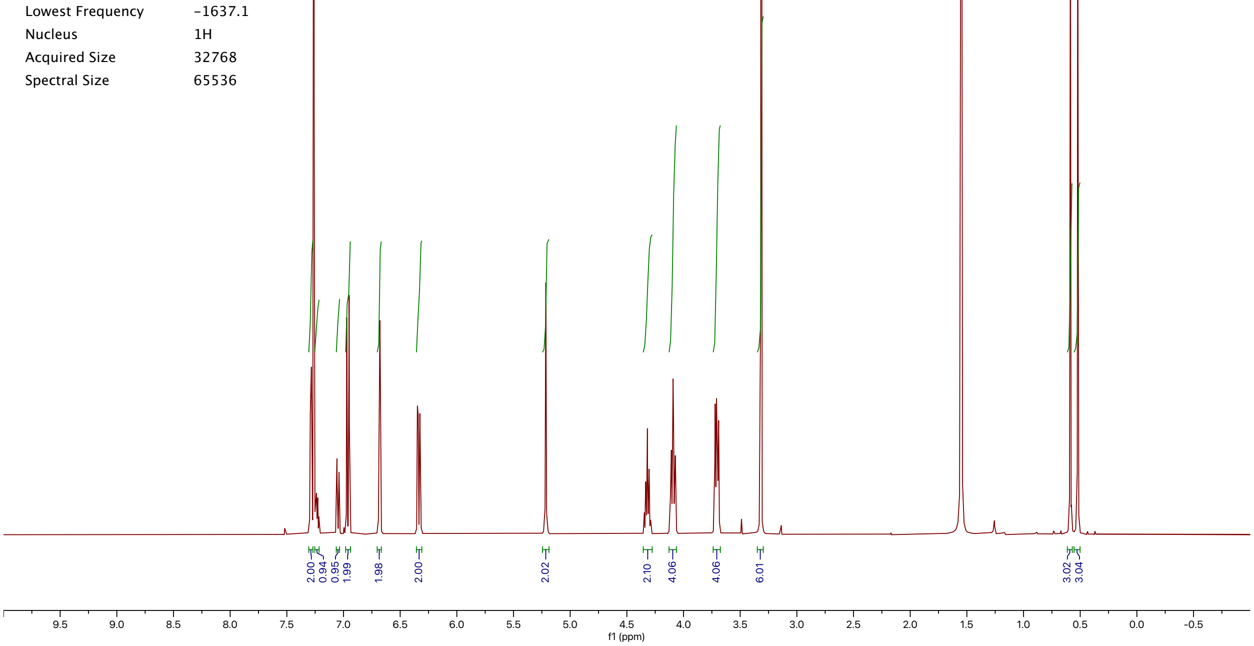

Origin	Bruker BioSpin GmbH
Solvent	CDCl3
Temperature	303.0
Pulse Sequence	zgpg30
Experiment	1D
Number of Scans	2048
Acquisition Date	2024-06-12T18:05:27
Spectrometer Frequency	100.62
Spectral Width	23809.5
Lowest Frequency	-1830.5
Nucleus	13C
Acquired Size	32768
Spectral Size	65536

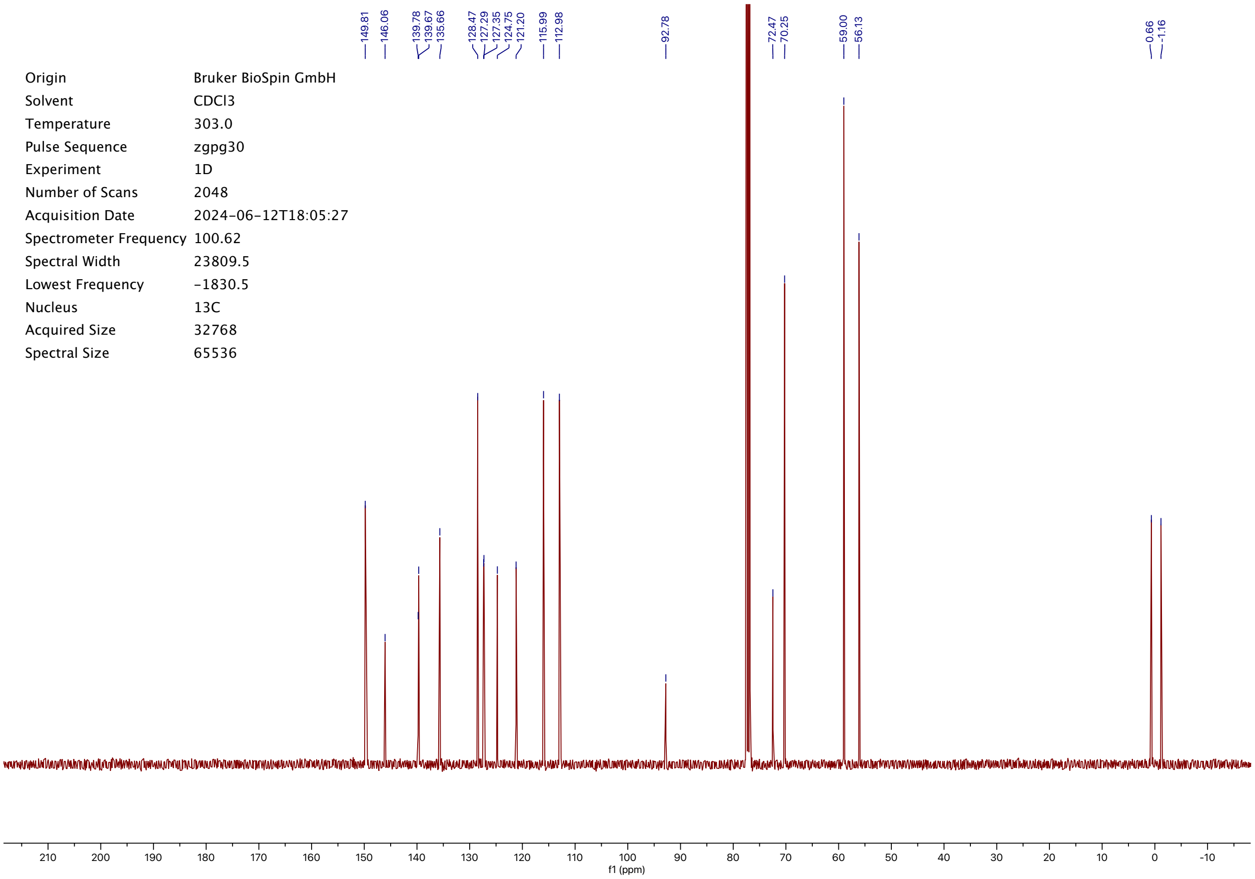

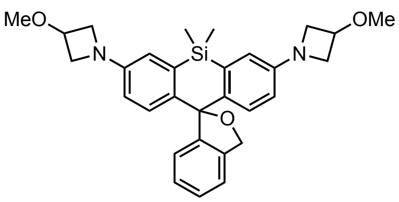

MeO
N
Si
N
OMe
O

4

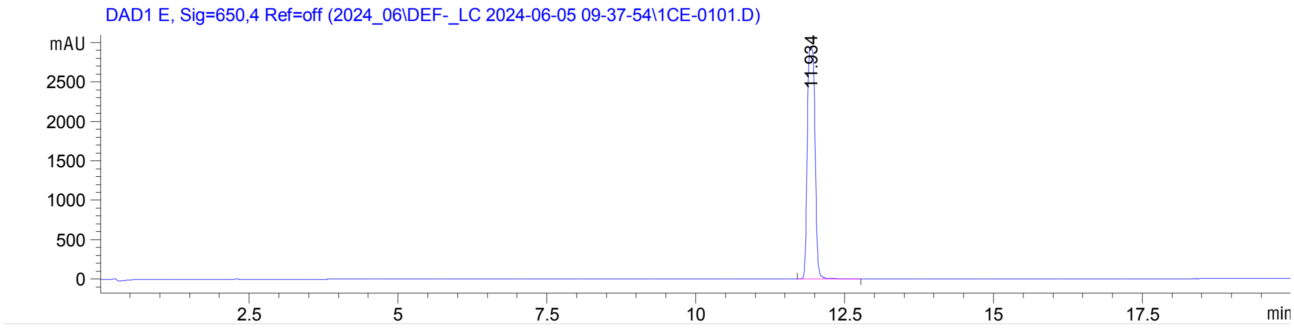

DAD1 E, Sig=650,4 Ref=off (2024_06\DEF-_LC 2024-06-05 09-37-54\1CE-0101.D)
mAU
2500
2000
1500
1000
500
0
11.934
2.5
5
7.5
10
12.5
15
17.5
min

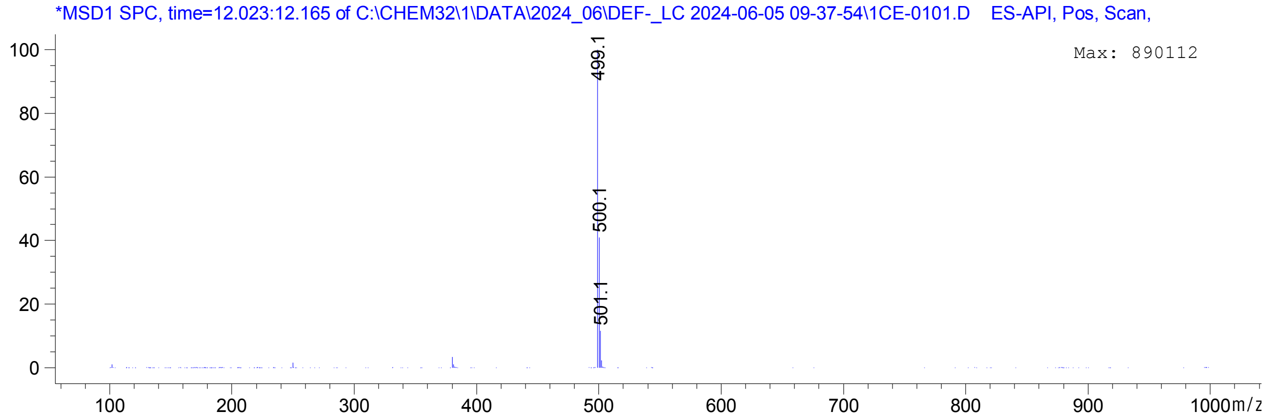

*MSD1 SPC, time=12.023:12.165 of C:\CHEM32\1\DATA\2024_06\DEF-_LC 2024-06-05 09-37-54\1CE-0101.D ES-API, Pos, Scan,
Max: 890112
100
80
60
40
20
0
499.1
500.1
501.1
100
200
300
400
500
600
700
800
900
1000
m/z

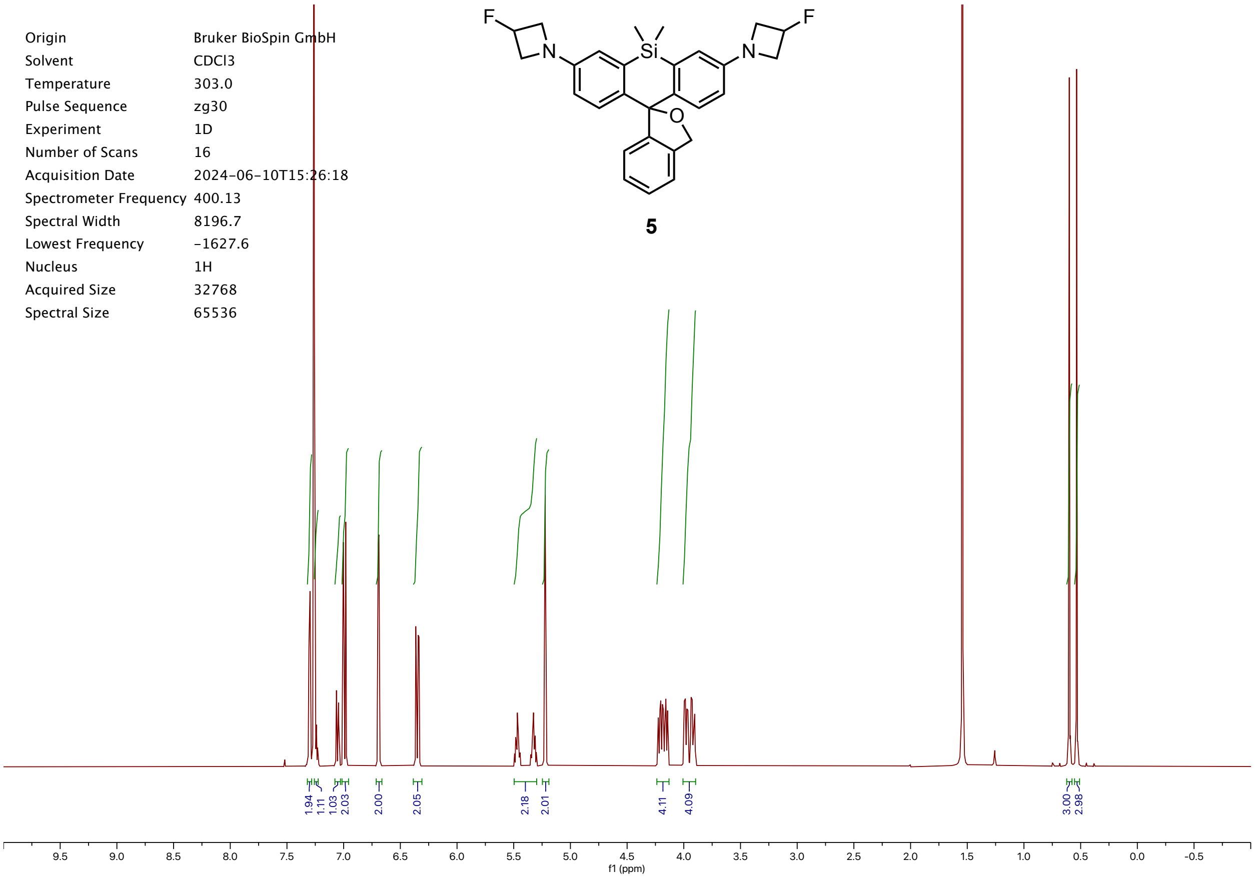
Origin Bruker BioSpin GmbH
Solvent CDCl3
Temperature 303.0
Pulse Sequence zg30
Experiment 1D
Number of Scans 16
Acquisition Date 2024-06-10T15:26:18
Spectrometer Frequency 400.13
Spectral Width 8196.7
Lowest Frequency -1627.6
Nucleus 1H
Acquired Size 32768
Spectral Size 65536
F
N
Si
O
5
1.94
1.11
1.03
2.03
2.00
2.05
2.18
2.01
4.11
4.09
3.00
2.98
f1 (ppm)

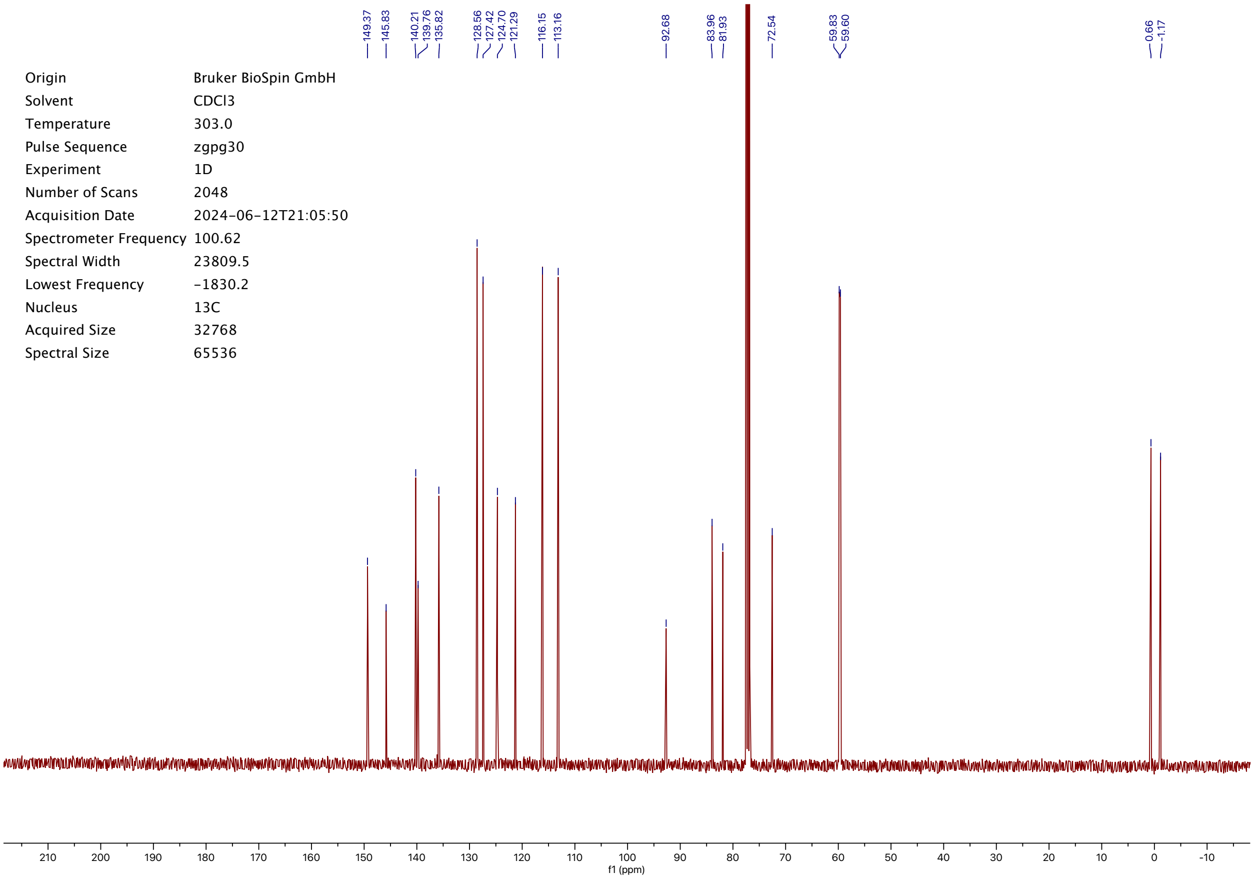
149.37
145.83
140.21
139.76
135.82
128.56
127.42
124.70
121.29
116.15
113.16
92.68
83.96
81.93
72.54
59.83
59.60
0.66
-1.17
Origin Bruker BioSpin GmbH
Solvent CDCl3
Temperature 303.0
Pulse Sequence zgpg30
Experiment 1D
Number of Scans 2048
Acquisition Date 2024-06-12T21:05:50
Spectrometer Frequency 100.62
Spectral Width 23809.5
Lowest Frequency -1830.2
Nucleus 13C
Acquired Size 32768
Spectral Size 65536
f1 (ppm)

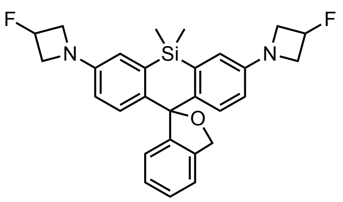

5

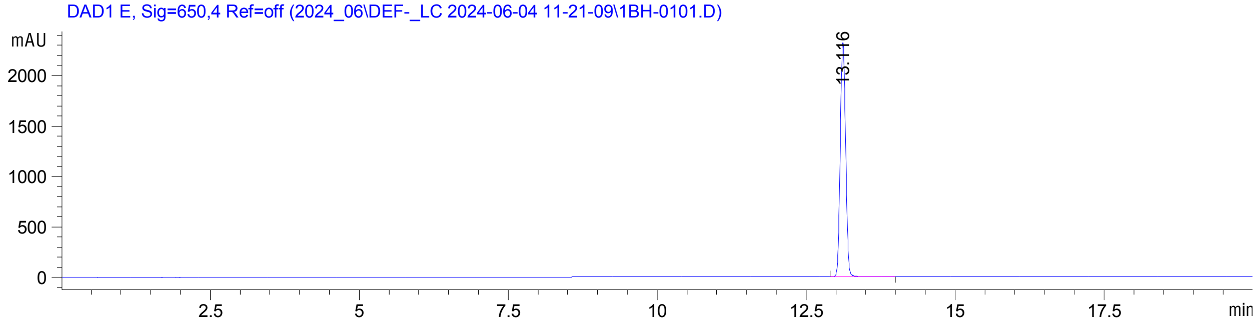

DAD1 E, Sig=650,4 Ref=off (2024_06\DEF-_LC 2024-06-04 11-21-09\1BH-0101.D)
mAU
2000
1500
1000
500
0
2.5
5
7.5
10
12.5
15
17.5
min
13.116

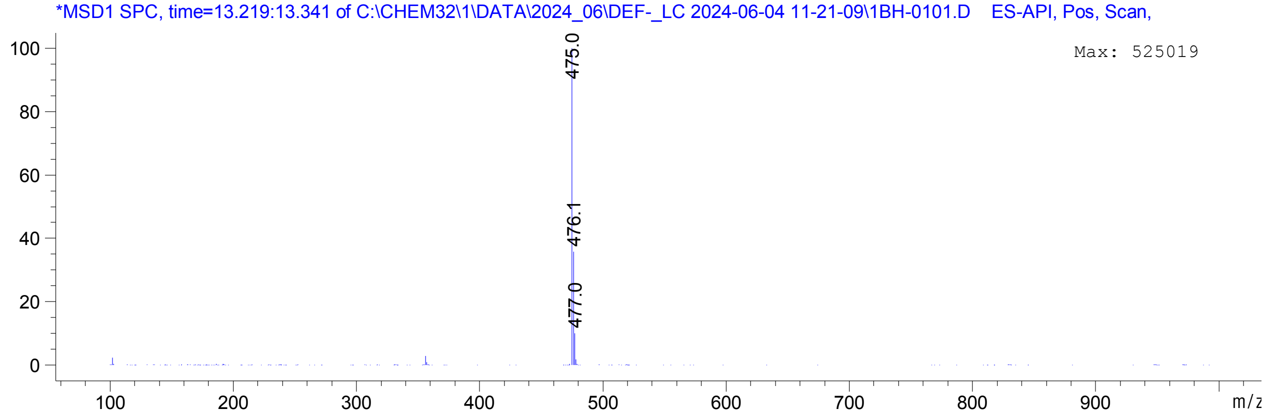

*MSD1 SPC, time=13.219:13.341 of C:\CHEM32\1\DATA\2024_06\DEF-_LC 2024-06-04 11-21-09\1BH-0101.D ES-API, Pos, Scan,
Max: 525019
100
80
60
40
20
0
100
200
300
400
500
600
700
800
900
m/z
475.0
476.1
477.0

Origin	Bruker BioSpin GmbH
Solvent	DMSO
Temperature	303.0
Pulse Sequence	zg30
Experiment	1D
Number of Scans	16
Acquisition Date	2024-06-14T13:17:08
Spectrometer Frequency	400.13
Spectral Width	8196.7
Lowest Frequency	-1630.0
Nucleus	1H
Acquired Size	32768
Spectral Size	65536

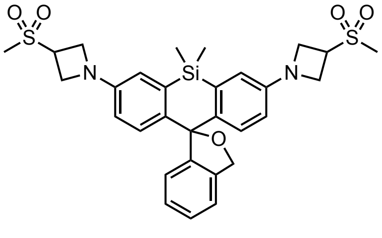

6

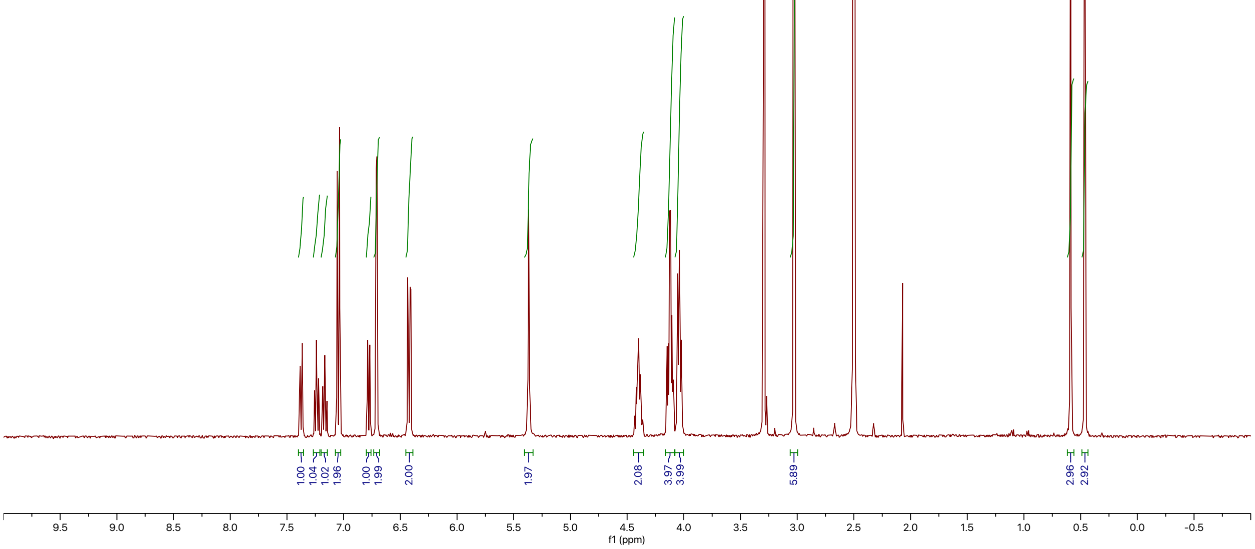

Origin	Bruker BioSpin GmbH
Solvent	DMSO
Temperature	303.0
Pulse Sequence	zgpg30
Experiment	1D
Number of Scans	2048
Acquisition Date	2024-06-15T12:02:06
Spectrometer Frequency	100.62
Spectral Width	23809.5
Lowest Frequency	-1895.8
Nucleus	13C
Acquired Size	32768
Spectral Size	65536

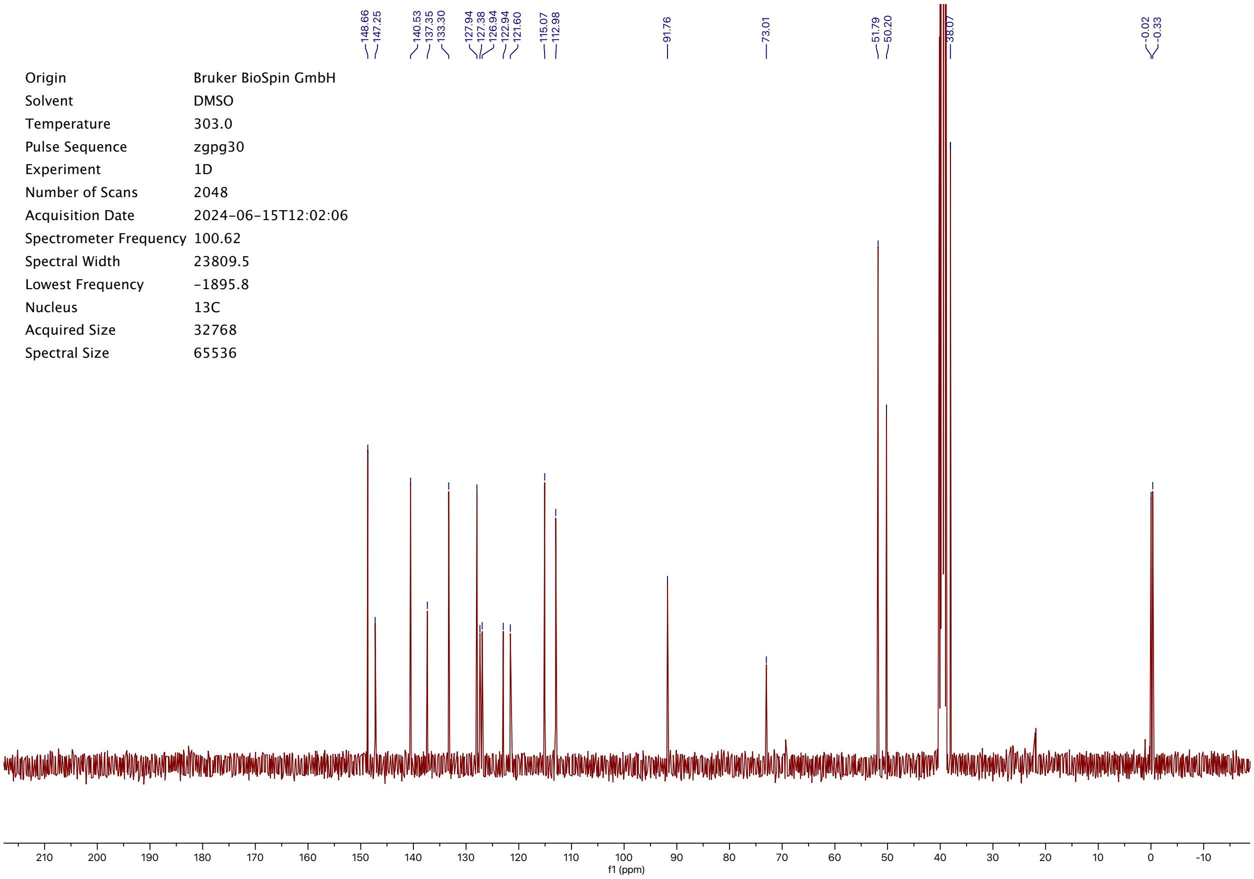

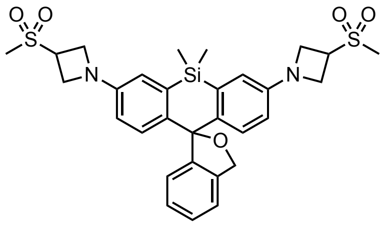

6

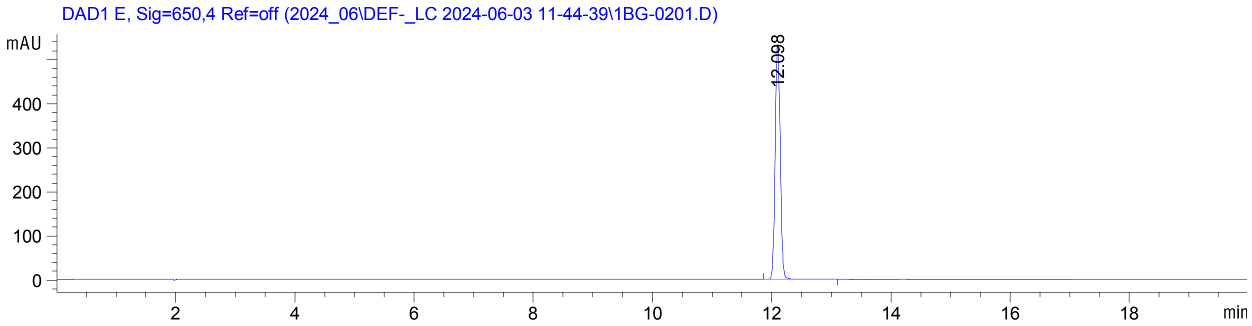

DAD1 E, Sig=650,4 Ref=off (2024_06\DEF-_LC 2024-06-03 11-44-39\1BG-0201.D)
mAU
400
300
200
100
0
12.098
2
4
6
8
10
12
14
16
18
min

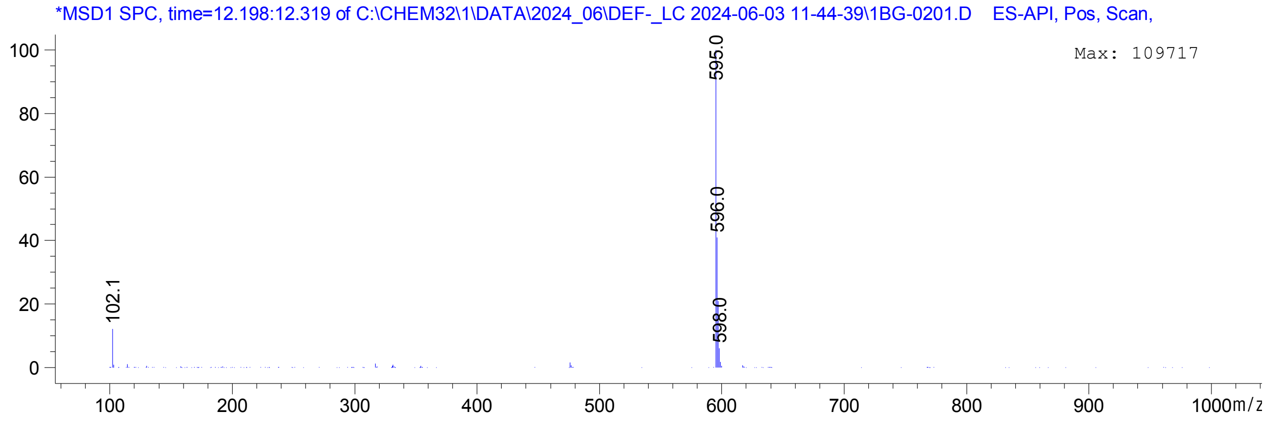

*MSD1 SPC, time=12.198:12.319 of C:\CHEM32\1\DATA\2024_06\DEF-_LC 2024-06-03 11-44-39\1BG-0201.D ES-API, Pos, Scan,
Max: 109717
100
80
60
40
20
0
595.0
596.0
598.0
102.1
100
200
300
400
500
600
700
800
900
1000
m/z

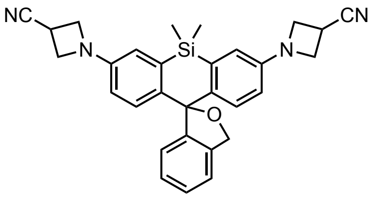

7

Origin	Bruker BioSpin GmbH
Solvent	CDCl3
Temperature	303.0
Pulse Sequence	zg30
Experiment	1D
Number of Scans	16
Acquisition Date	2024-06-10T15:15:11
Spectrometer Frequency	400.13
Spectral Width	8196.7
Lowest Frequency	-1637.2
Nucleus	1H
Acquired Size	32768
Spectral Size	65536

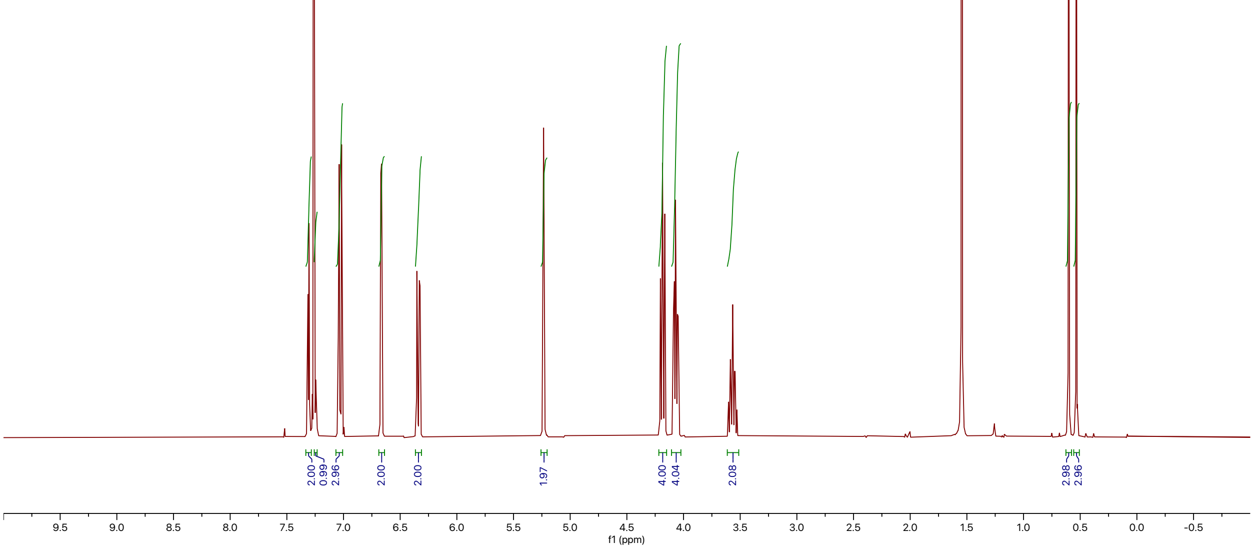

Origin	Bruker BioSpin GmbH
Solvent	CDCl3
Temperature	303.0
Pulse Sequence	zgpg30
Experiment	1D
Number of Scans	2048
Acquisition Date	2024-06-12T02:15:36
Spectrometer Frequency	100.62
Spectral Width	23809.5
Lowest Frequency	-1831.6
Nucleus	13C
Acquired Size	32768
Spectral Size	65536

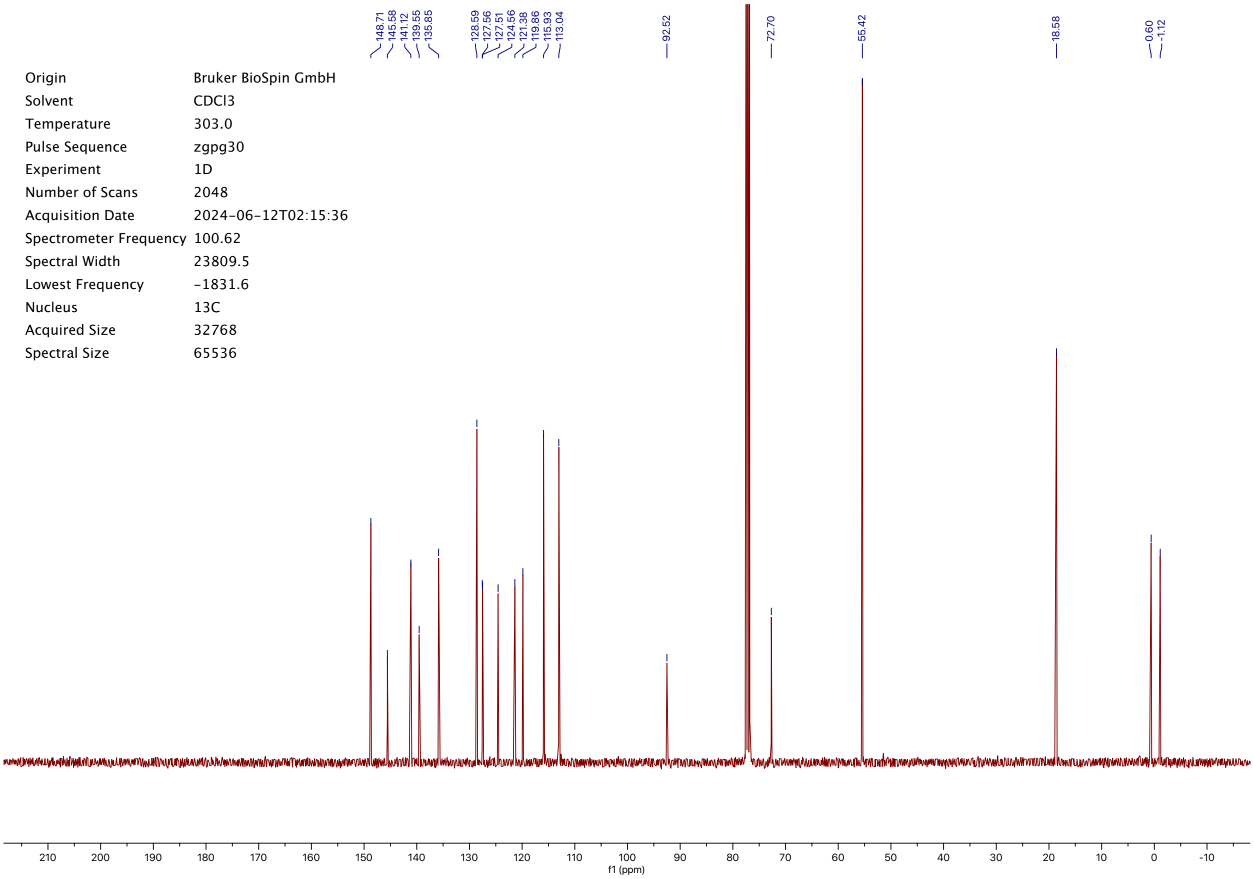

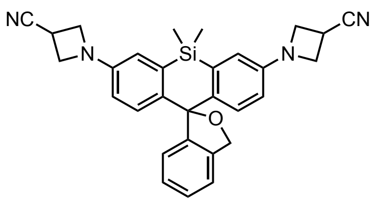

NC
CN
N
N
Si
O

7

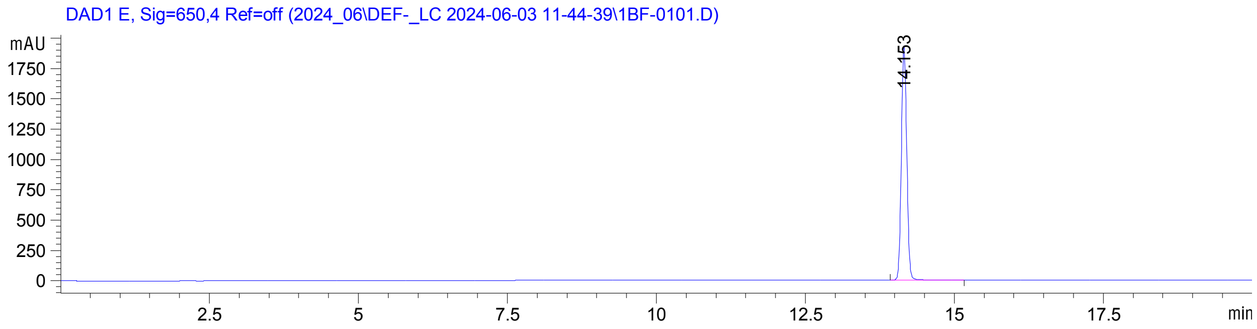

DAD1 E, Sig=650,4 Ref=off (2024_06\DEF-_LC 2024-06-03 11-44-39\1BF-0101.D)
mAU
1750
1500
1250
1000
750
500
250
0
14.153
2.5
5
7.5
10
12.5
15
17.5
min

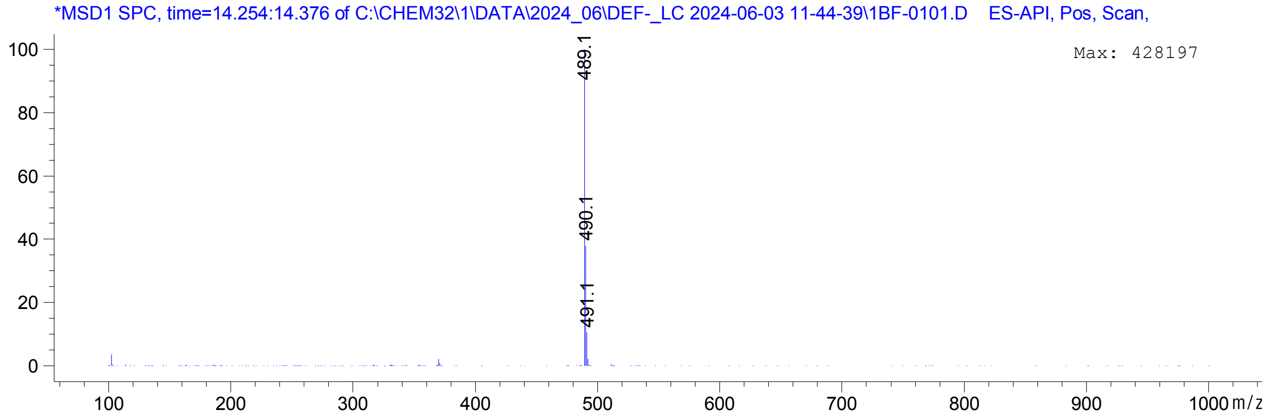

*MSD1 SPC, time=14.254:14.376 of C:\CHEM32\1\DATA\2024_06\DEF-_LC 2024-06-03 11-44-39\1BF-0101.D ES-API, Pos, Scan,
Max: 428197
100
80
60
40
20
0
489.1
490.1
491.1
100
200
300
400
500
600
700
800
900
1000
m/z

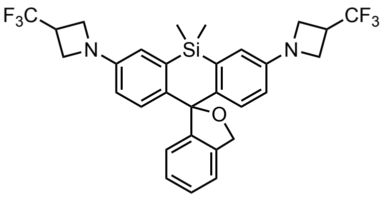

8

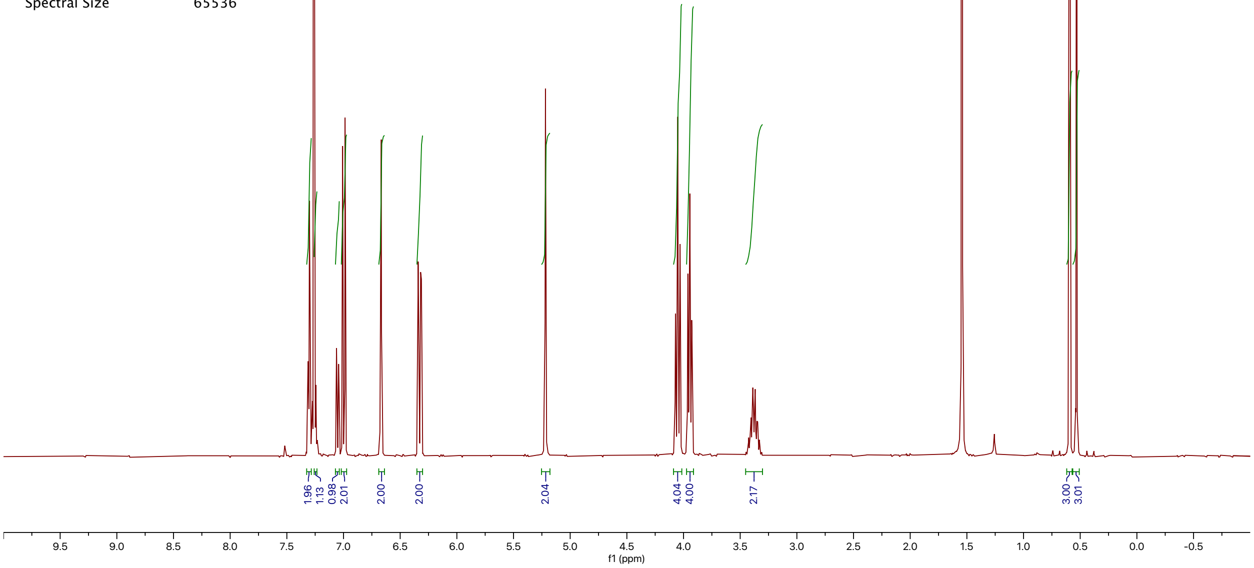

Origin Bruker BioSpin GmbH
Solvent CDCl3
Temperature 303.0
Pulse Sequence zg30
Experiment 1D
Number of Scans 16
Acquisition Date 2024-06-10T15:09:49
Spectrometer Frequency 400.13
Spectral Width 8196.7
Lowest Frequency -1637.1
Nucleus 1H
Acquired Size 32768
Spectral Size 65536
1.96
1.13
0.98
2.01
2.00
2.00
2.04
4.04
4.00
2.17
3.00
3.01
9.5 9.0 8.5 8.0 7.5 7.0 6.5 6.0 5.5 5.0 4.5 4.0 3.5 3.0 2.5 2.0 1.5 1.0 0.5 0.0 -0.5
f1 (ppm)

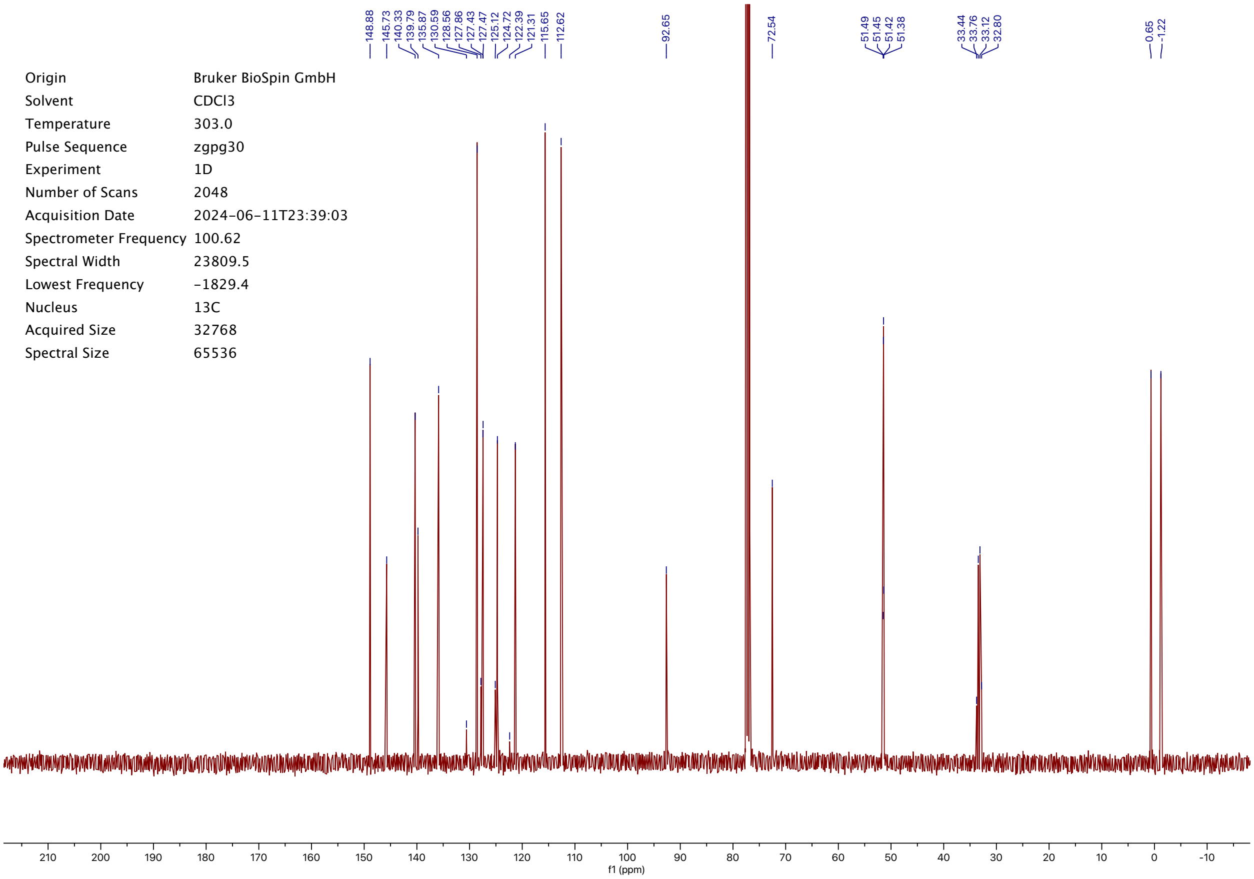

148.88
145.73
140.33
139.79
135.87
130.59
128.56
127.86
127.47
127.43
125.12
124.72
122.39
121.31
115.65
112.62
92.65
72.54
51.49
51.45
51.42
51.38
33.44
33.76
33.12
32.80
0.65
-1.22
Origin Bruker BioSpin GmbH
Solvent CDCl3
Temperature 303.0
Pulse Sequence zgpg30
Experiment 1D
Number of Scans 2048
Acquisition Date 2024-06-11T23:39:03
Spectrometer Frequency 100.62
Spectral Width 23809.5
Lowest Frequency -1829.4
Nucleus 13C
Acquired Size 32768
Spectral Size 65536
210 200 190 180 170 160 150 140 130 120 110 100 90 80 70 60 50 40 30 20 10 0 -10
f1 (ppm)

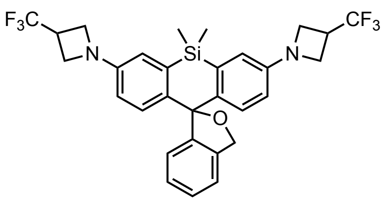
F3C
CF3
N
N
Si
O

8

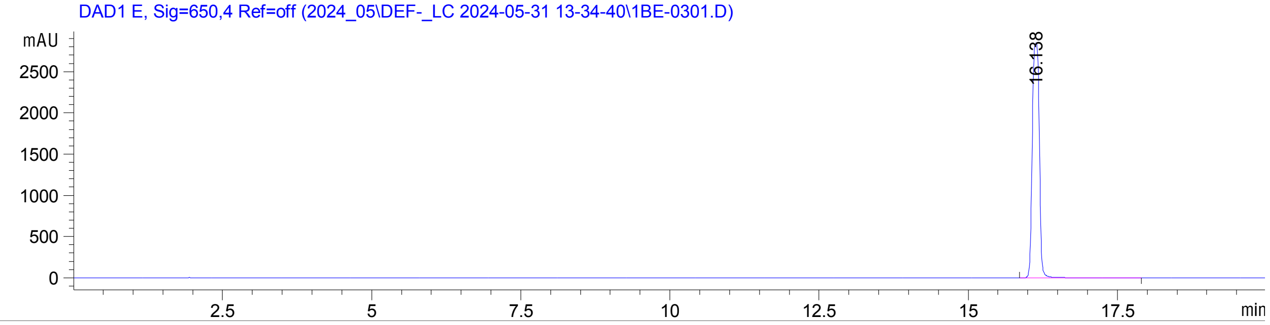
DAD1 E, Sig=650,4 Ref=off (2024_05\DEF-_LC 2024-05-31 13-34-40\1BE-0301.D)
mAU
2500
2000
1500
1000
500
0
16.138
2.5
5
7.5
10
12.5
15
17.5
min

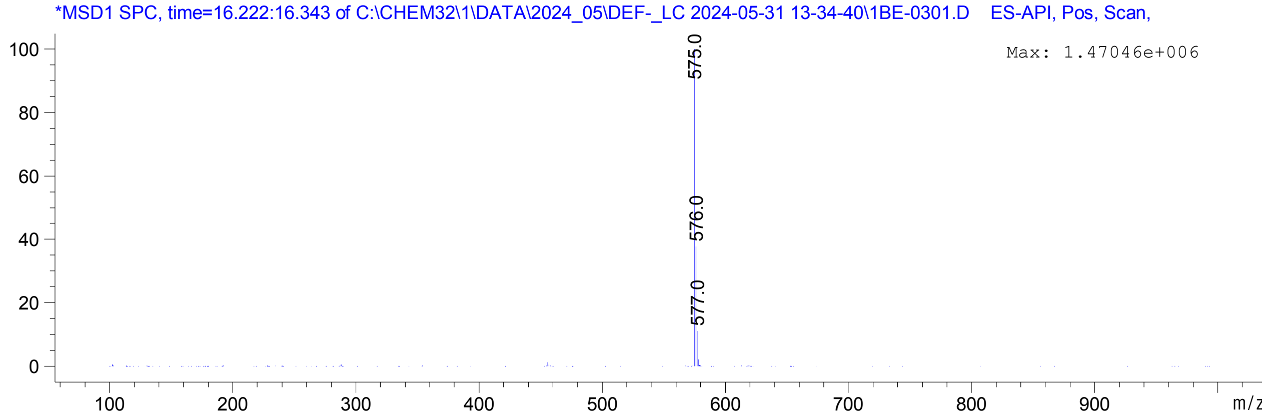
*MSD1 SPC, time=16.222:16.343 of C:\CHEM32\1\DATA\2024_05\DEF-_LC 2024-05-31 13-34-40\1BE-0301.D ES-API, Pos, Scan,
Max: 1.47046e+006
100
80
60
40
20
0
575.0
576.0
577.0
100
200
300
400
500
600
700
800
900
m/z

Origin	Bruker BioSpin GmbH
Solvent	CDCl3
Temperature	303.0
Pulse Sequence	zg30
Experiment	1D
Number of Scans	16
Acquisition Date	2024-06-10T15:04:32
Spectrometer Frequency	400.13
Spectral Width	8196.7
Lowest Frequency	-1637.2
Nucleus	1H
Acquired Size	32768
Spectral Size	65536

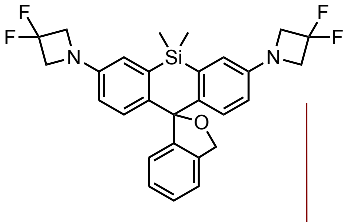

9

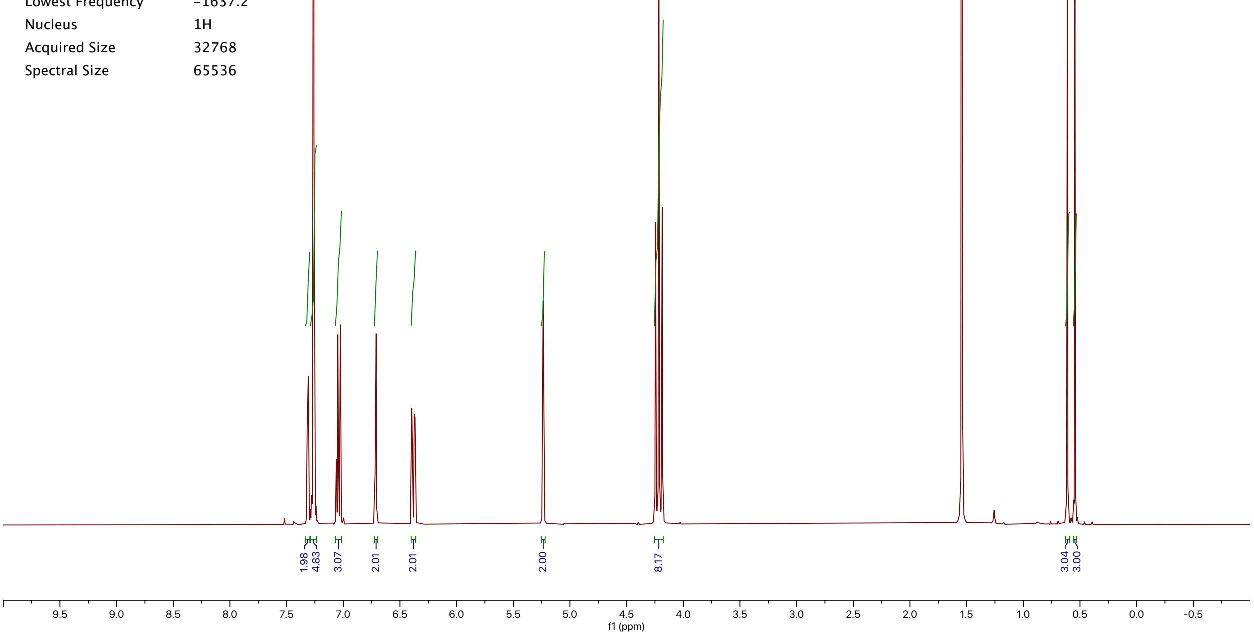

Origin	Bruker BioSpin GmbH
Solvent	CDCl3
Temperature	303.0
Pulse Sequence	zgpg30
Experiment	1D
Number of Scans	2048
Acquisition Date	2024-06-11T21:02:30
Spectrometer Frequency	100.62
Spectral Width	23809.5
Lowest Frequency	-1829.8
Nucleus	13C
Acquired Size	32768
Spectral Size	65536

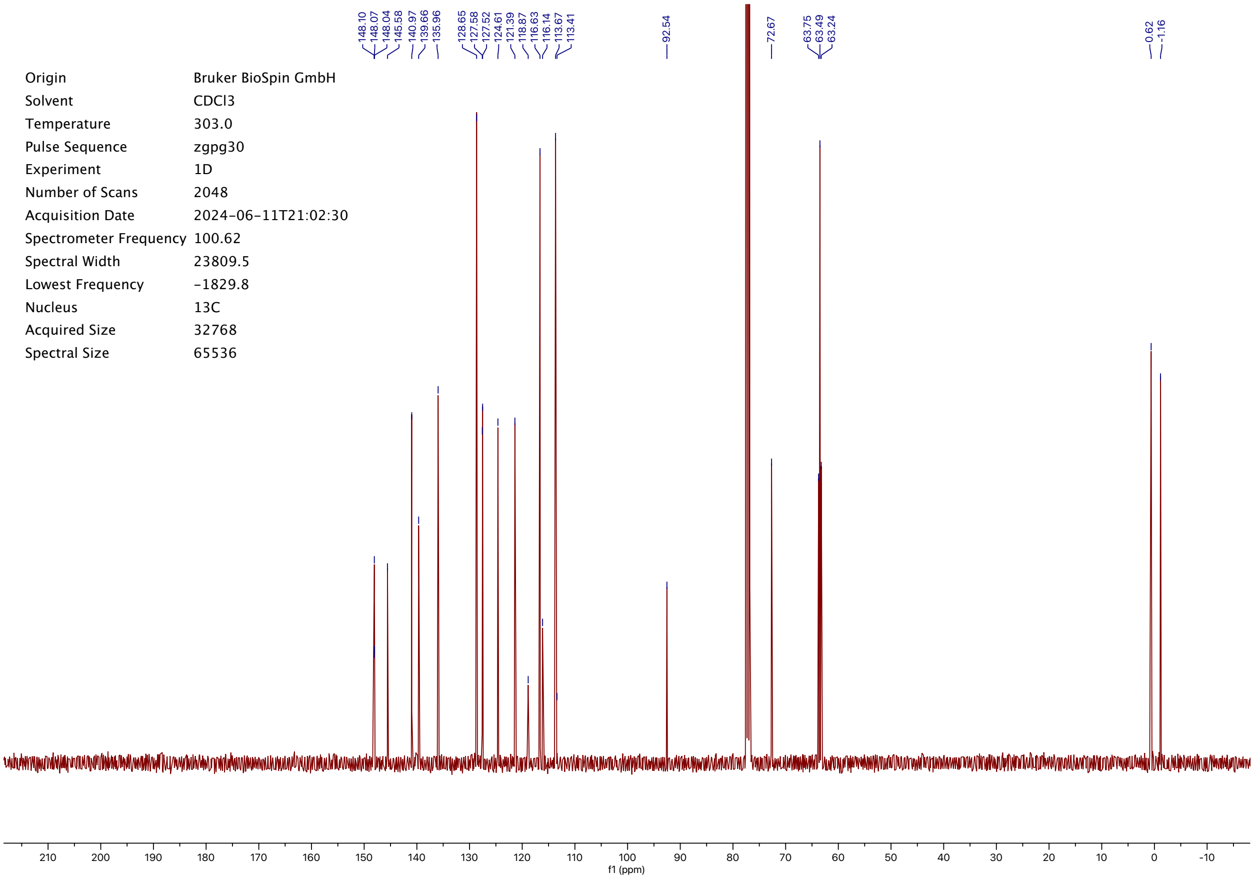

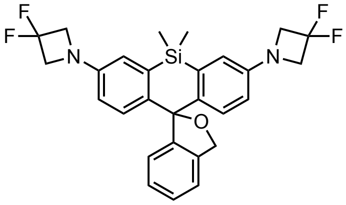

9

DAD1 A, Sig=254,4 Ref=off (2024_05\DEF-_LC 2024-05-30 11-32-35\1BD-0101.D)

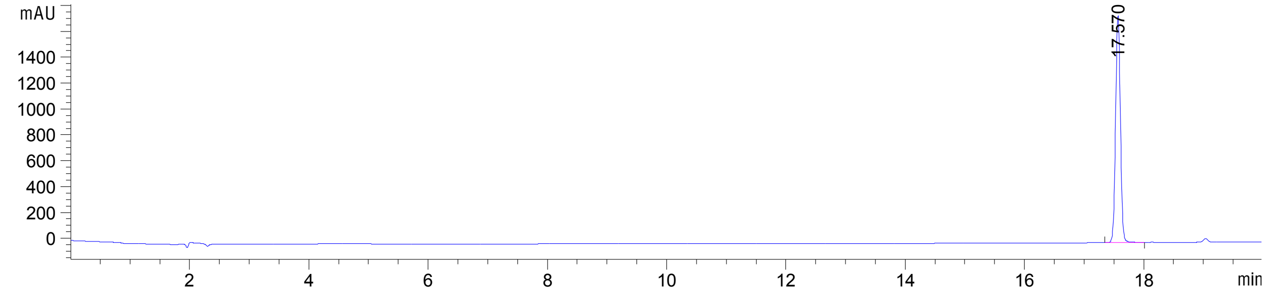

*MSD1 SPC, time=17.662:17.784 of C:\CHEM32\1\DATA\2024_05\DEF-_LC 2024-05-30 11-32-35\1BD-0101.D ES-API, Pos, Scan,

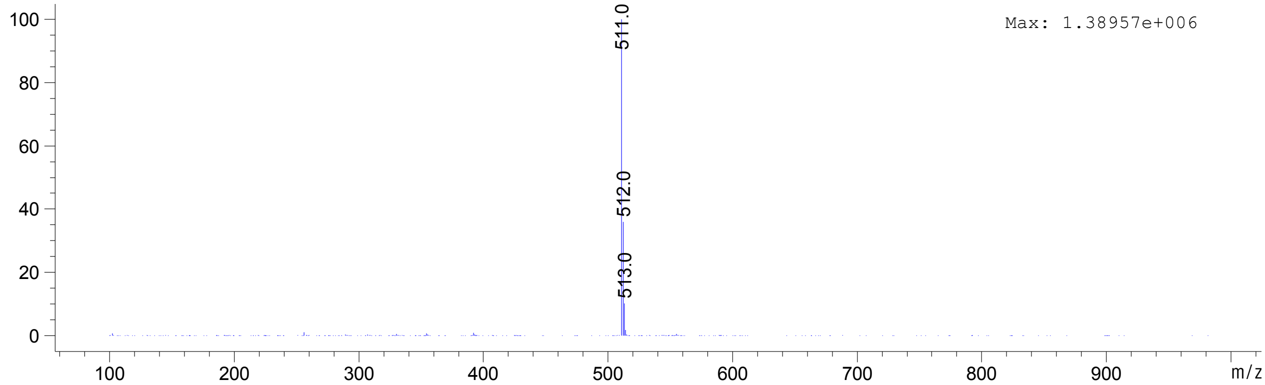

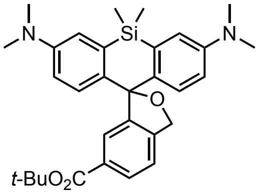

Si
N
N
O
t-BuO2C

S8

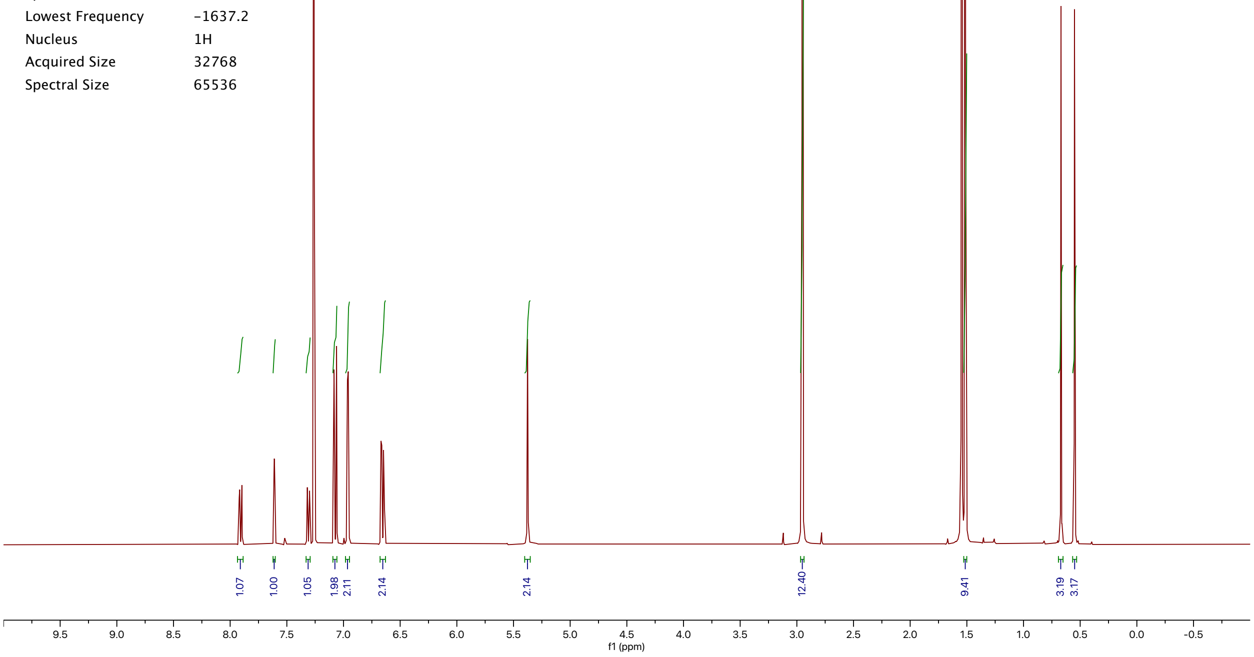

Origin Bruker BioSpin GmbH
Solvent CDCl3
Temperature 303.0
Pulse Sequence zg30
Experiment 1D
Number of Scans 16
Acquisition Date 2024-06-24T14:15:19
Spectrometer Frequency 400.13
Spectral Width 8196.7
Lowest Frequency -1637.2
Nucleus 1H
Acquired Size 32768
Spectral Size 65536
1.07
1.00
1.05
1.98
2.11
2.14
2.14
12.40
9.41
3.19
3.17
9.5 9.0 8.5 8.0 7.5 7.0 6.5 6.0 5.5 5.0 4.5 4.0 3.5 3.0 2.5 2.0 1.5 1.0 0.5 0.0 -0.5
f1 (ppm)

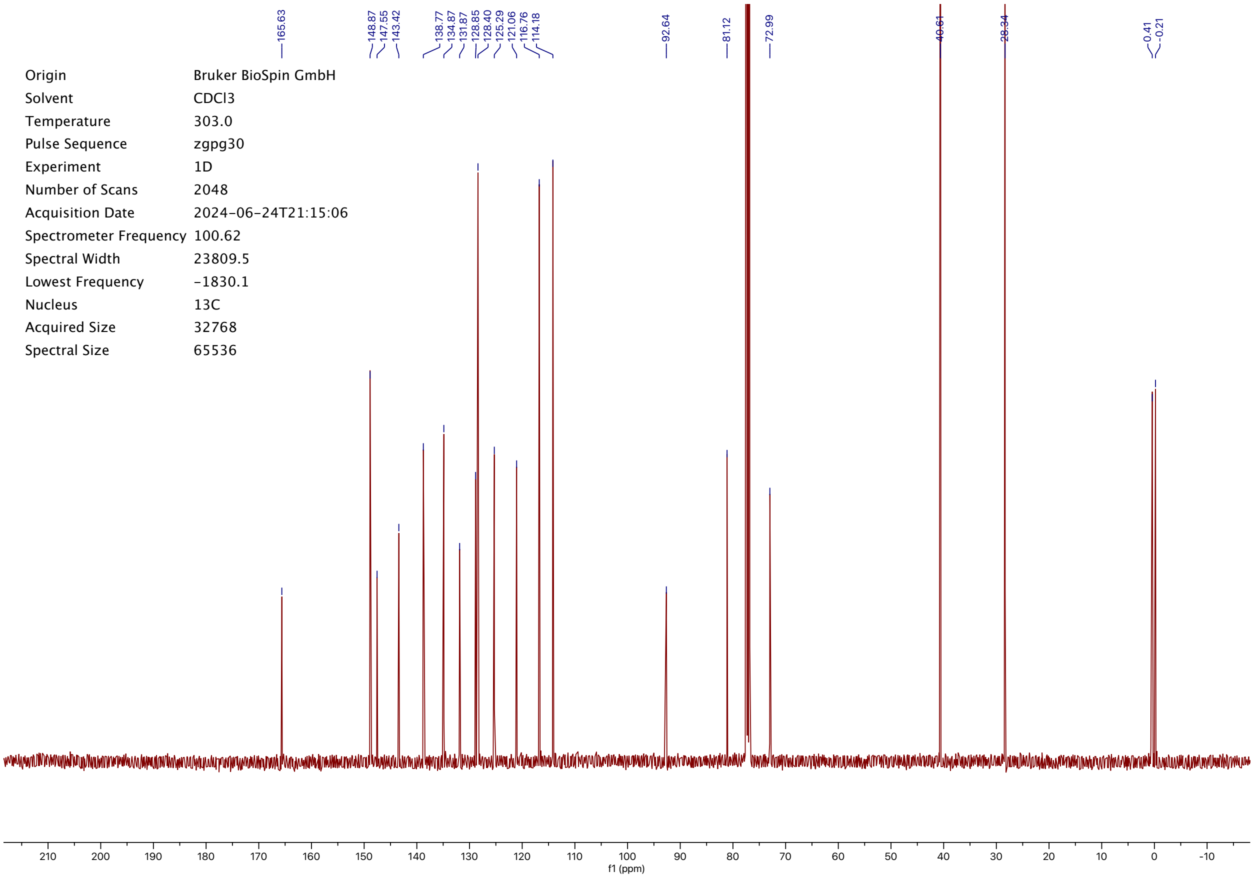

165.63
148.87
147.55
143.42
138.77
134.87
131.87
128.85
128.40
125.29
121.06
116.76
114.18
92.64
81.12
72.99
40.61
28.34
0.41
-0.21
Origin Bruker BioSpin GmbH
Solvent CDCl3
Temperature 303.0
Pulse Sequence zgpg30
Experiment 1D
Number of Scans 2048
Acquisition Date 2024-06-24T21:15:06
Spectrometer Frequency 100.62
Spectral Width 23809.5
Lowest Frequency -1830.1
Nucleus 13C
Acquired Size 32768
Spectral Size 65536
210 200 190 180 170 160 150 140 130 120 110 100 90 80 70 60 50 40 30 20 10 0 -10
f1 (ppm)

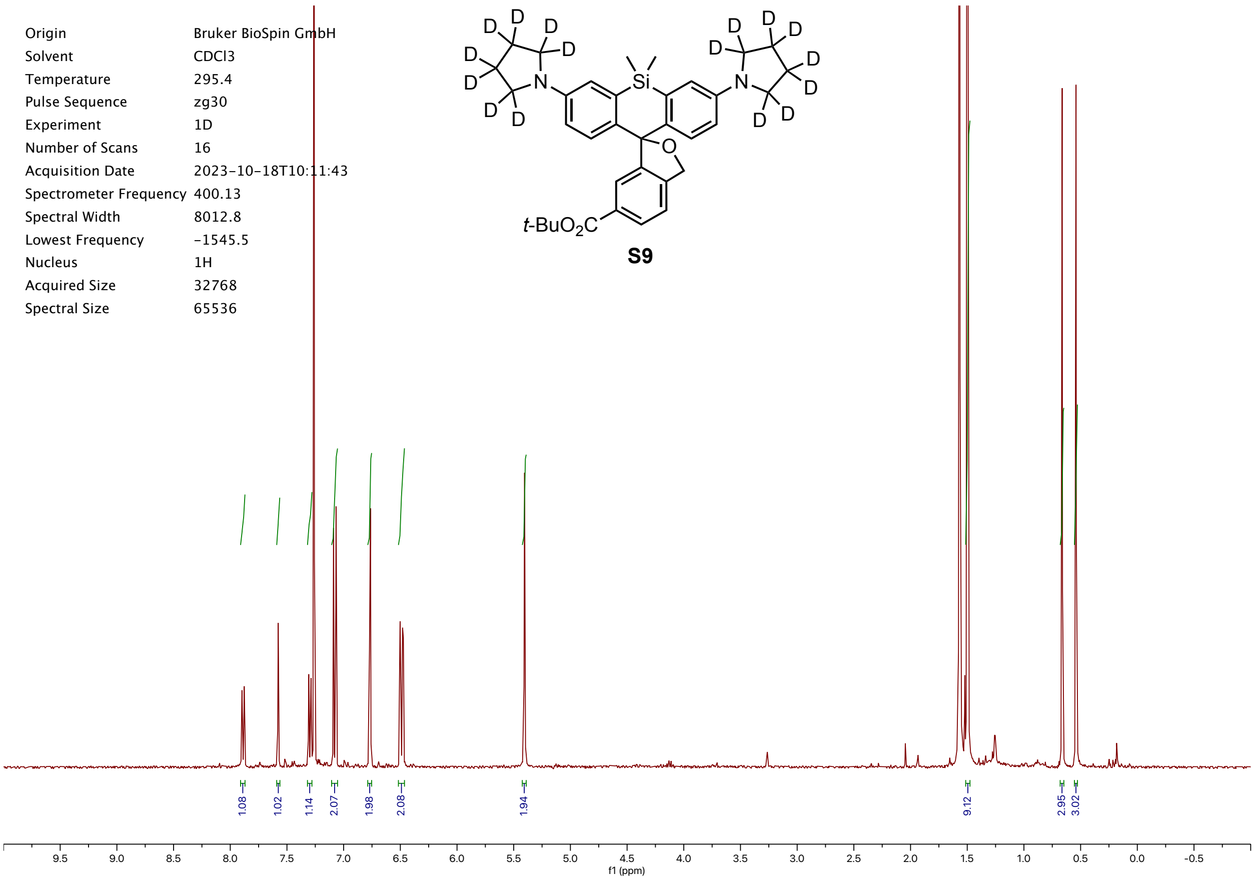
Origin Bruker BioSpin GmbH
Solvent CDCl3
Temperature 295.4
Pulse Sequence zg30
Experiment 1D
Number of Scans 16
Acquisition Date 2023-10-18T10:11:43
Spectrometer Frequency 400.13
Spectral Width 8012.8
Lowest Frequency -1545.5
Nucleus 1H
Acquired Size 32768
Spectral Size 65536
t-BuO2C
S9
1.08
1.02
1.14
2.07
1.98
2.08
1.94
9.12
2.95
3.02
f1 (ppm)

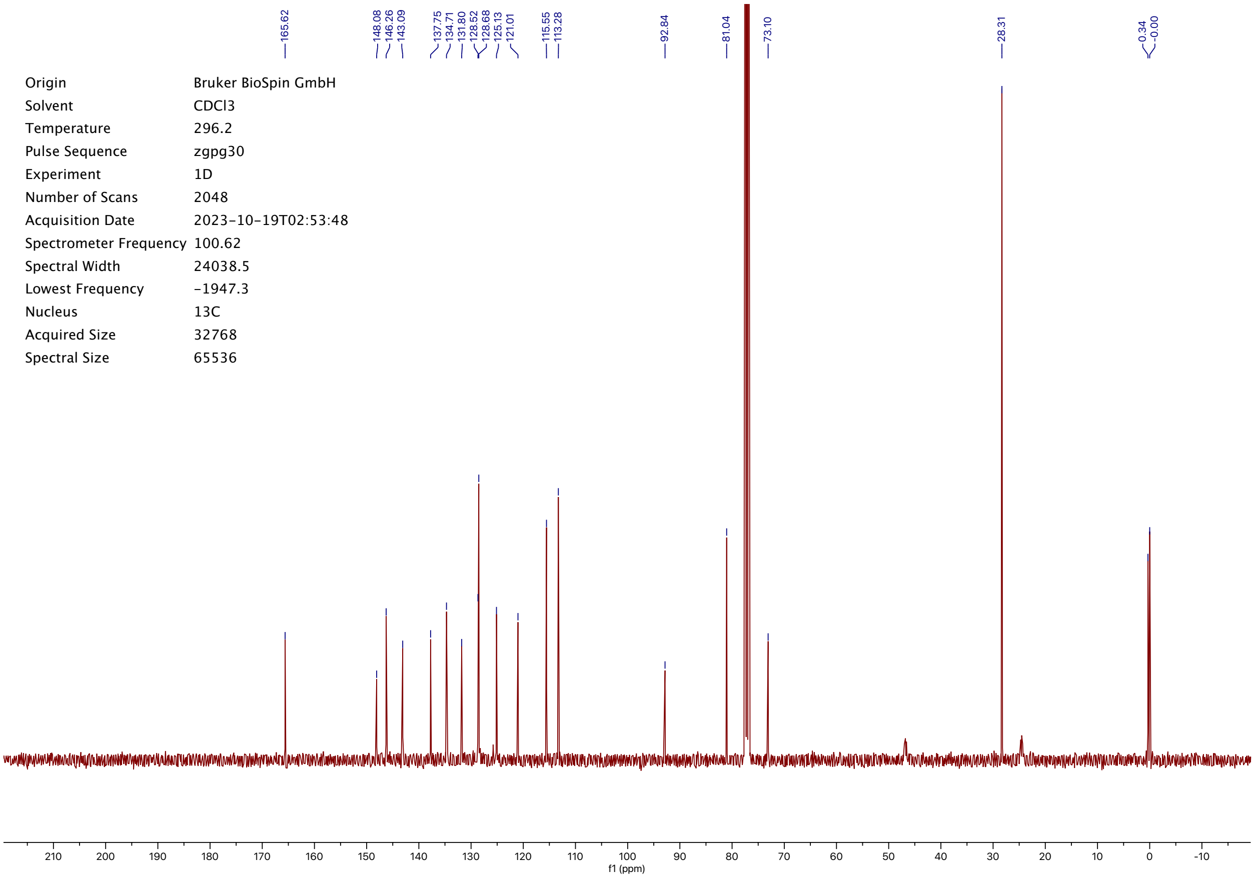
165.62
148.08
146.26
143.09
137.75
134.71
131.80
128.82
128.68
125.13
121.01
115.55
113.28
92.84
81.04
73.10
28.31
0.34
-0.00
Origin Bruker BioSpin GmbH
Solvent CDCl3
Temperature 296.2
Pulse Sequence zgpg30
Experiment 1D
Number of Scans 2048
Acquisition Date 2023-10-19T02:53:48
Spectrometer Frequency 100.62
Spectral Width 24038.5
Lowest Frequency -1947.3
Nucleus 13C
Acquired Size 32768
Spectral Size 65536
f1 (ppm)

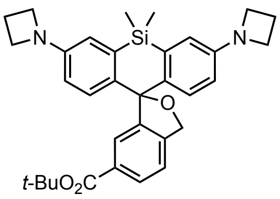

S10

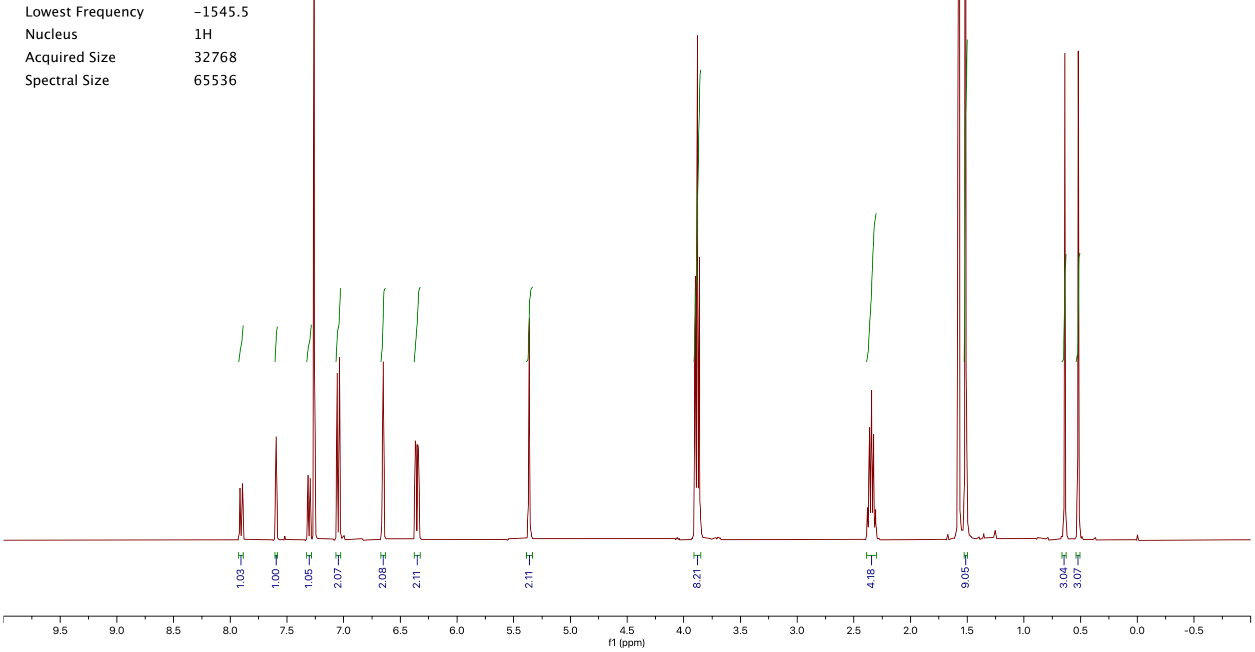

Origin Bruker BioSpin GmbH
Solvent CDCl3
Temperature 295.3
Pulse Sequence zg30
Experiment 1D
Number of Scans 16
Acquisition Date 2023-10-16T09:42:30
Spectrometer Frequency 400.13
Spectral Width 8012.8
Lowest Frequency -1545.5
Nucleus 1H
Acquired Size 32768
Spectral Size 65536
1.03 1.00 1.05 2.07 2.08 2.11 2.11 8.21 4.18 9.05 3.04 3.07
9.5 9.0 8.5 8.0 7.5 7.0 6.5 6.0 5.5 5.0 4.5 4.0 3.5 3.0 2.5 2.0 1.5 1.0 0.5 0.0 -0.5
f1 (ppm)

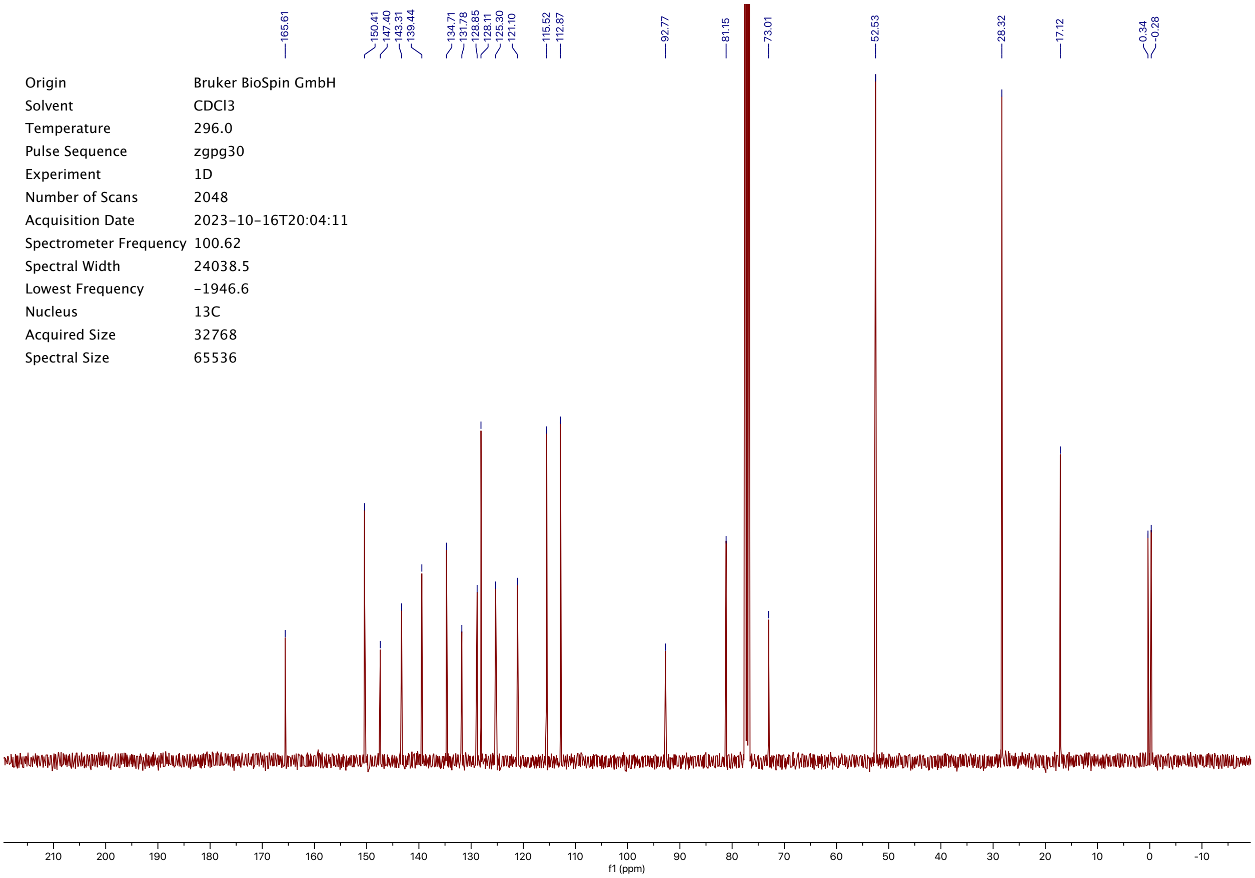

165.61
150.41 147.40 143.31 139.44
134.71 131.78 128.85 128.11 125.30 121.10
115.52 112.87
92.77
81.15
73.01
52.53
28.32
17.12
0.34 -0.28
Origin Bruker BioSpin GmbH
Solvent CDCl3
Temperature 296.0
Pulse Sequence zgpg30
Experiment 1D
Number of Scans 2048
Acquisition Date 2023-10-16T20:04:11
Spectrometer Frequency 100.62
Spectral Width 24038.5
Lowest Frequency -1946.6
Nucleus 13C
Acquired Size 32768
Spectral Size 65536
210 200 190 180 170 160 150 140 130 120 110 100 90 80 70 60 50 40 30 20 10 0 -10
f1 (ppm)

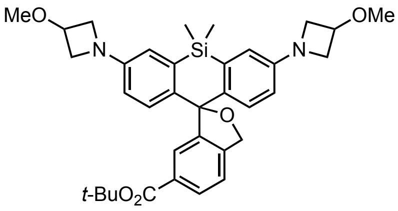

S11

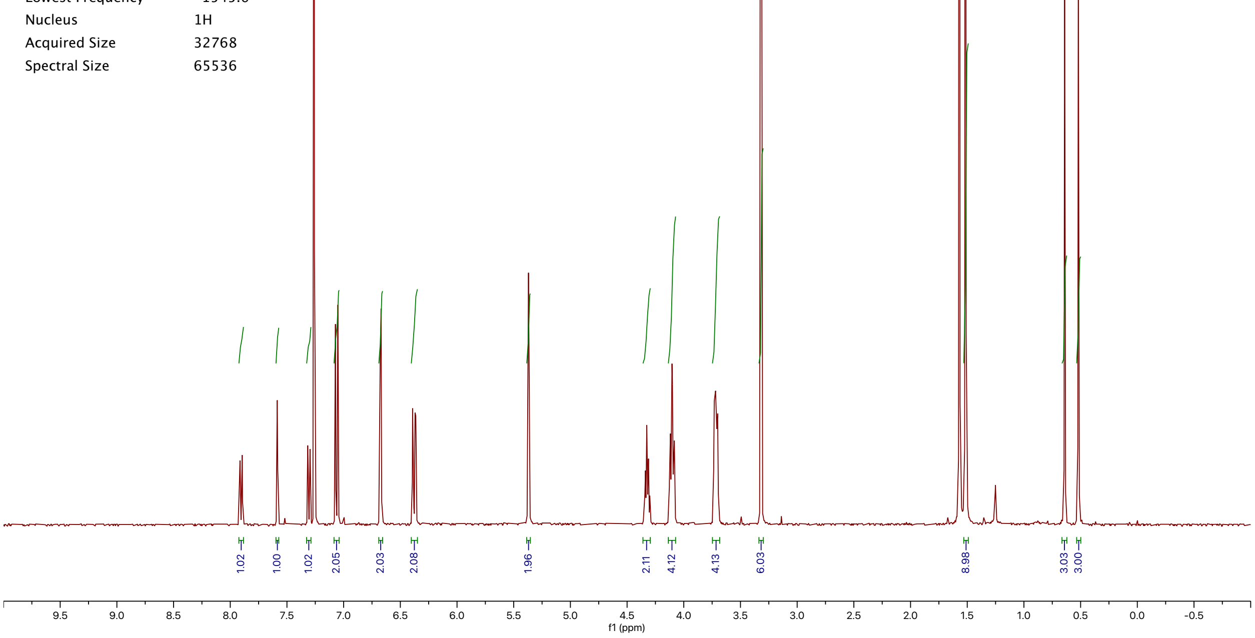

Origin	Bruker BioSpin GmbH
Solvent	CDCl3
Temperature	295.3
Pulse Sequence	zg30
Experiment	1D
Number of Scans	16
Acquisition Date	2023-10-17T10:56:00
Spectrometer Frequency	400.13
Spectral Width	8012.8
Lowest Frequency	-1545.6
Nucleus	1H
Acquired Size	32768
Spectral Size	65536

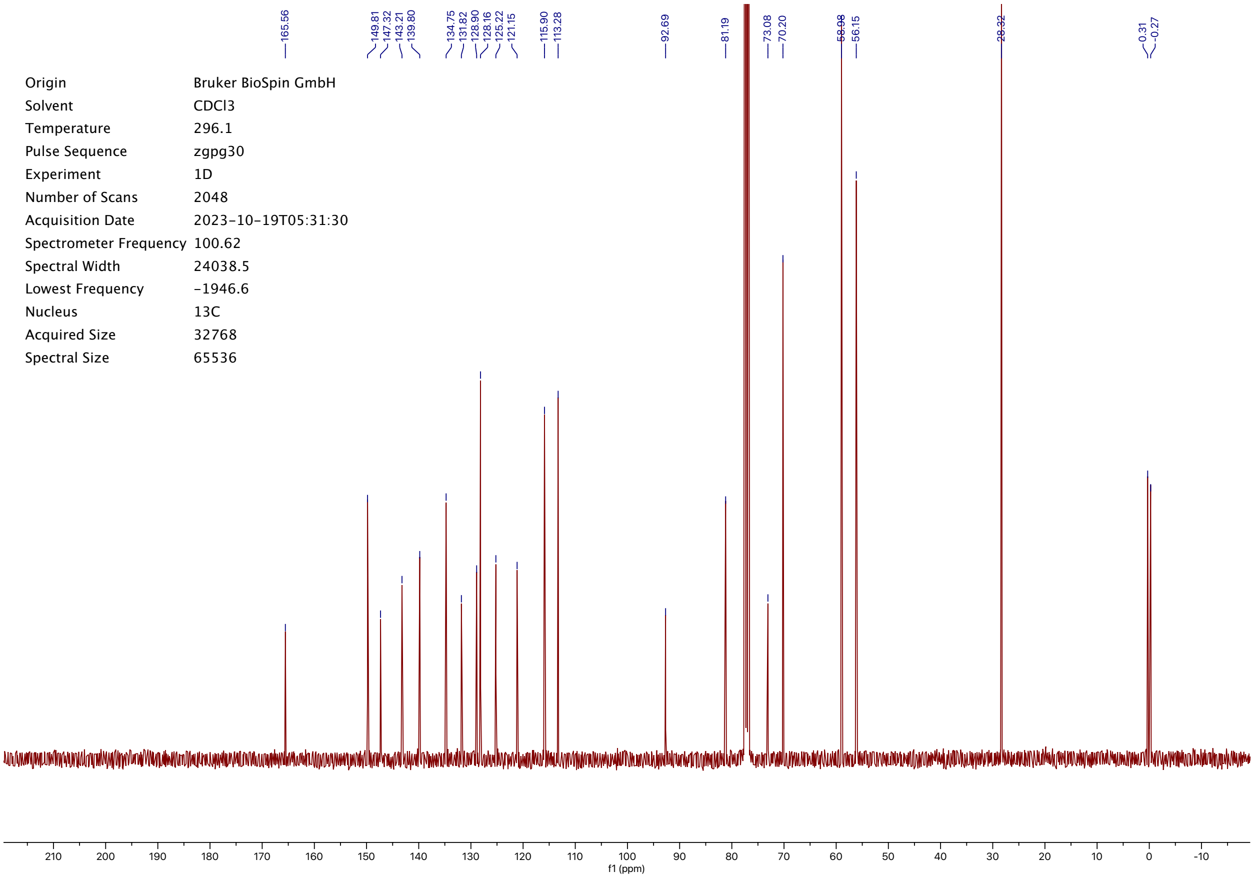

Origin	Bruker BioSpin GmbH
Solvent	CDCl3
Temperature	296.1
Pulse Sequence	zgpg30
Experiment	1D
Number of Scans	2048
Acquisition Date	2023-10-19T05:31:30
Spectrometer Frequency	100.62
Spectral Width	24038.5
Lowest Frequency	-1946.6
Nucleus	13C
Acquired Size	32768
Spectral Size	65536

Origin	Bruker BioSpin GmbH
Solvent	CDCl3
Temperature	295.4
Pulse Sequence	zg30
Experiment	1D
Number of Scans	16
Acquisition Date	2023-10-18T09:56:07
Spectrometer Frequency	400.13
Spectral Width	8012.8
Lowest Frequency	-1535.6
Nucleus	1H
Acquired Size	32768
Spectral Size	65536

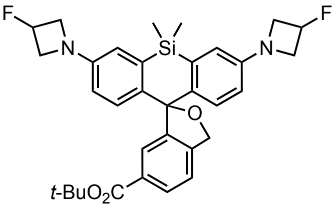

S12

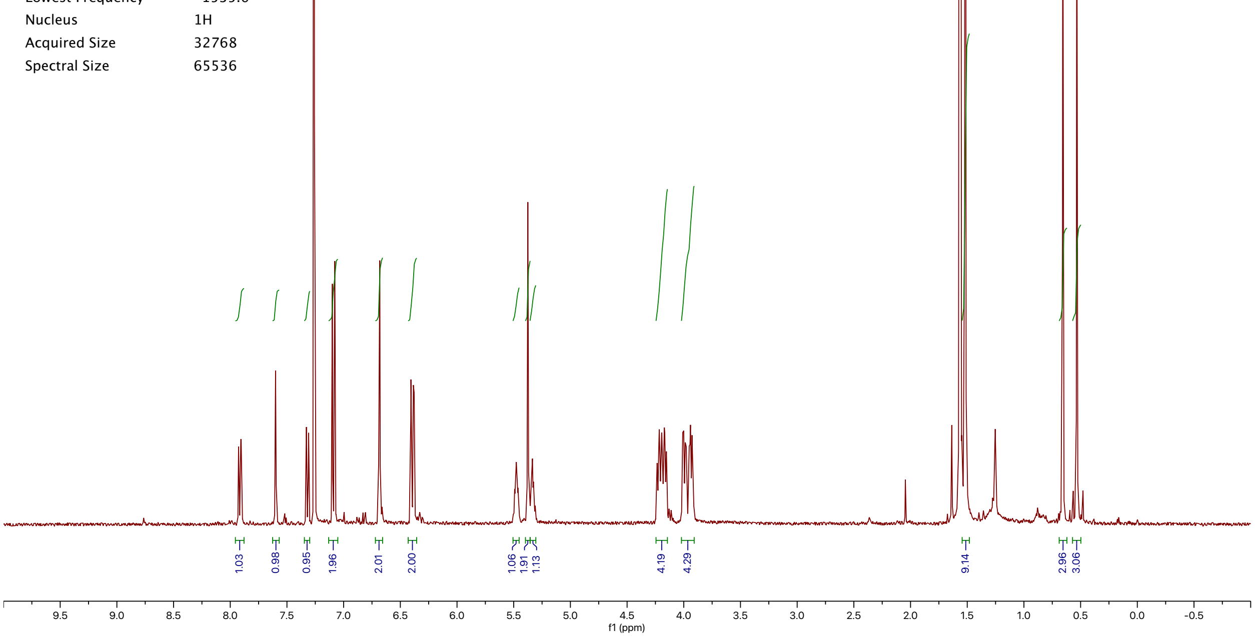

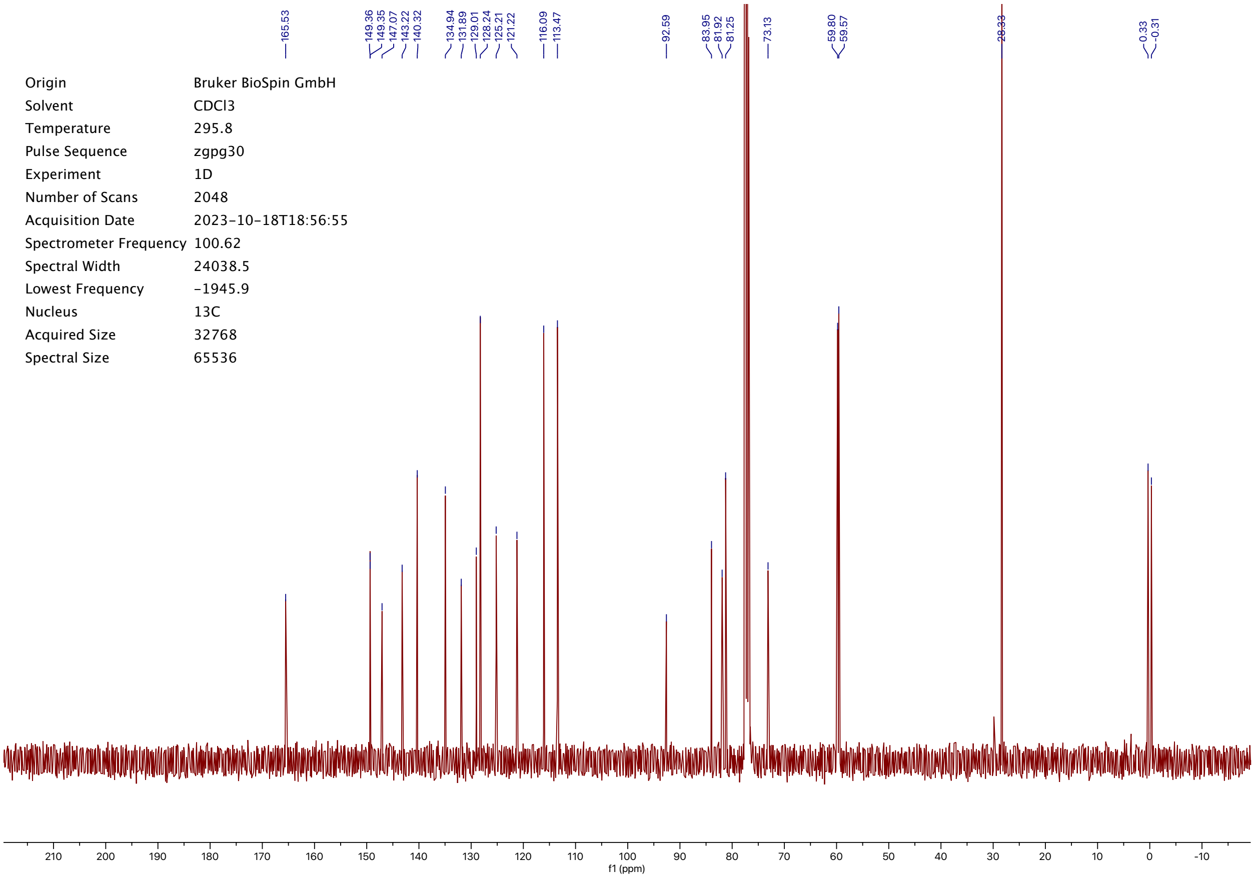

Origin	Bruker BioSpin GmbH
Solvent	CDCl3
Temperature	295.8
Pulse Sequence	zgpg30
Experiment	1D
Number of Scans	2048
Acquisition Date	2023-10-18T18:56:55
Spectrometer Frequency	100.62
Spectral Width	24038.5
Lowest Frequency	-1945.9
Nucleus	13C
Acquired Size	32768
Spectral Size	65536

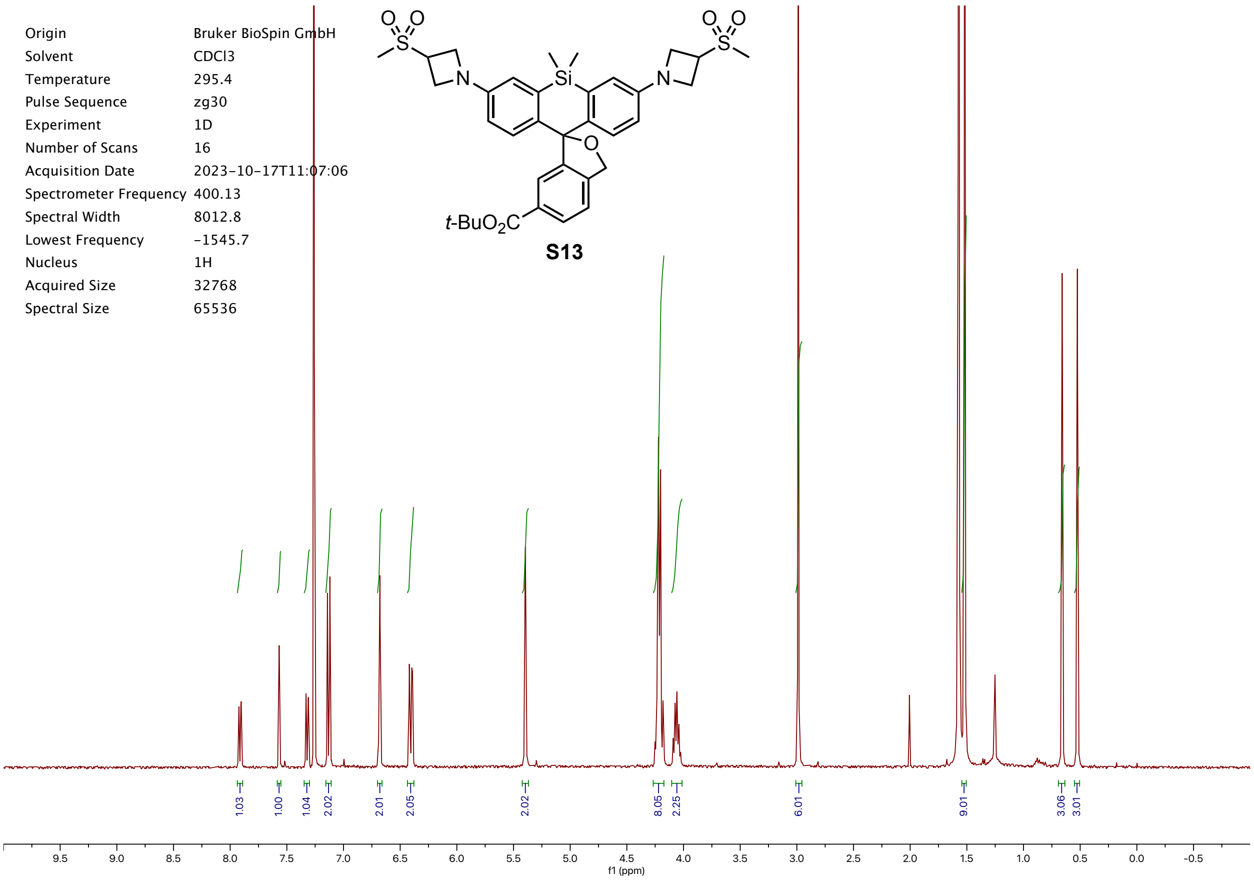
S13
Origin Bruker BioSpin GmbH
Solvent CDCl3
Temperature 295.4
Pulse Sequence zg30
Experiment 1D
Number of Scans 16
Acquisition Date 2023-10-17T11:07:06
Spectrometer Frequency 400.13
Spectral Width 8012.8
Lowest Frequency -1545.7
Nucleus 1H
Acquired Size 32768
Spectral Size 65536
1.03
1.00
1.04
2.02
2.01
2.05
2.02
8.05
2.25
6.01
9.01
3.06
3.01
9.5 9.0 8.5 8.0 7.5 7.0 6.5 6.0 5.5 5.0 4.5 4.0 3.5 3.0 2.5 2.0 1.5 1.0 0.5 0.0 -0.5
f1 (ppm)

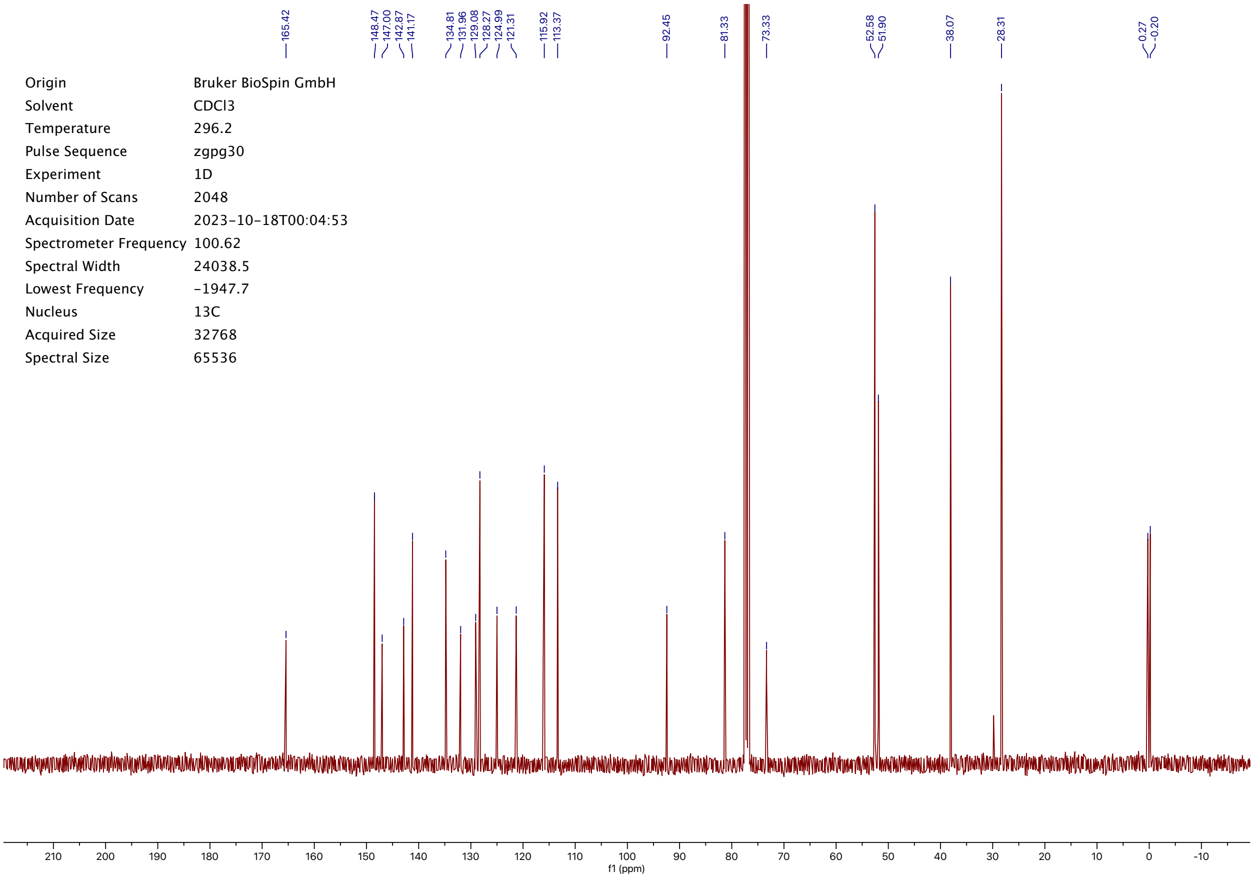
165.42
148.47
147.00
142.87
141.17
134.81
131.96
129.08
128.27
124.99
121.31
115.92
113.37
92.45
81.33
73.33
52.58
51.90
38.07
28.31
0.27
-0.20
Origin Bruker BioSpin GmbH
Solvent CDCl3
Temperature 296.2
Pulse Sequence zgpg30
Experiment 1D
Number of Scans 2048
Acquisition Date 2023-10-18T00:04:53
Spectrometer Frequency 100.62
Spectral Width 24038.5
Lowest Frequency -1947.7
Nucleus 13C
Acquired Size 32768
Spectral Size 65536
210 200 190 180 170 160 150 140 130 120 110 100 90 80 70 60 50 40 30 20 10 0 -10
f1 (ppm)

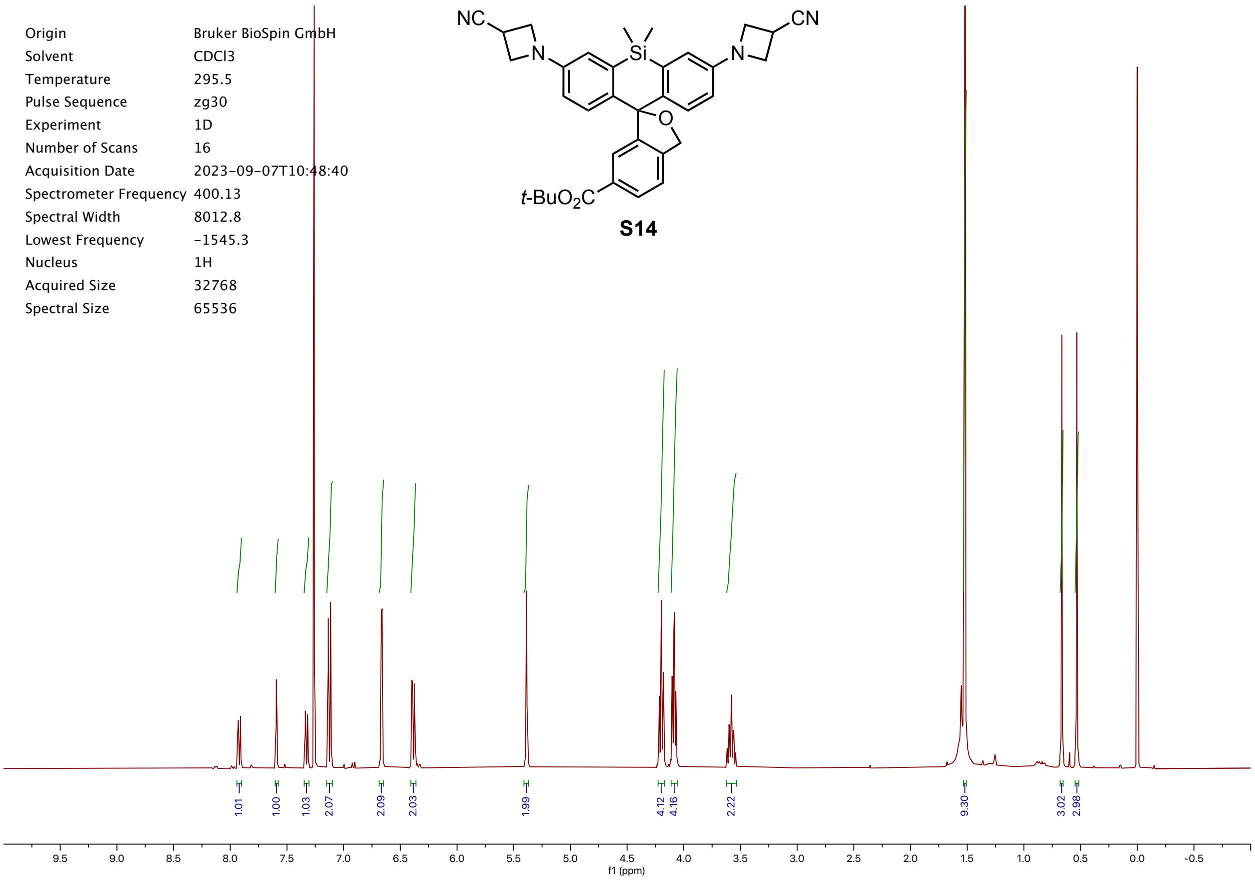
NC
CN
Si
N
N
O
t-BuO2C
S14
Origin Bruker BioSpin GmbH
Solvent CDCl3
Temperature 295.5
Pulse Sequence zg30
Experiment 1D
Number of Scans 16
Acquisition Date 2023-09-07T10:48:40
Spectrometer Frequency 400.13
Spectral Width 8012.8
Lowest Frequency -1545.3
Nucleus 1H
Acquired Size 32768
Spectral Size 65536
1.01
1.00
1.03
2.07
2.09
2.03
1.99
4.12
4.16
2.22
9.30
3.02
2.98
f1 (ppm)

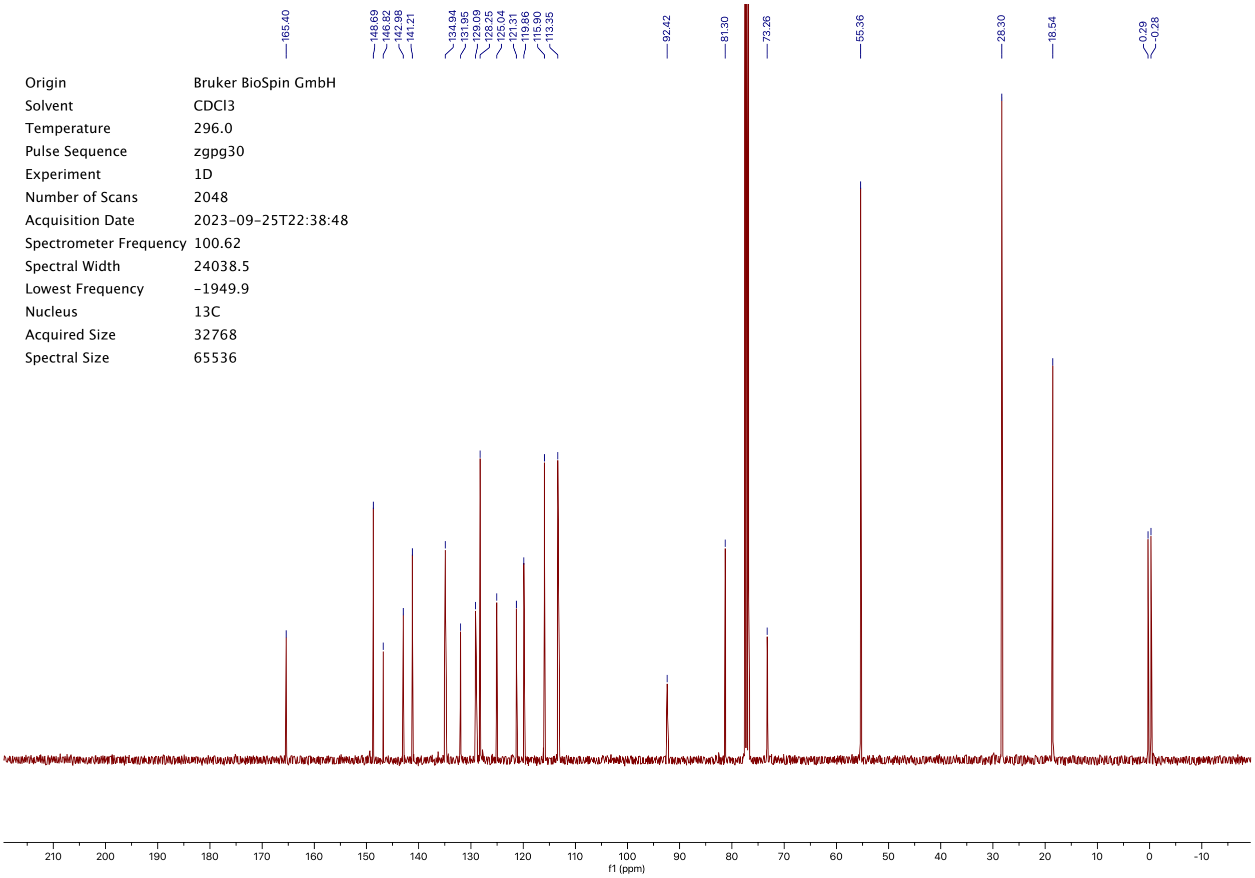
165.40
148.69
146.82
142.98
141.21
134.94
131.95
129.09
128.25
125.04
121.31
119.86
115.90
113.35
92.42
81.30
73.26
55.36
28.30
18.54
0.29
-0.28
Origin Bruker BioSpin GmbH
Solvent CDCl3
Temperature 296.0
Pulse Sequence zgpg30
Experiment 1D
Number of Scans 2048
Acquisition Date 2023-09-25T22:38:48
Spectrometer Frequency 100.62
Spectral Width 24038.5
Lowest Frequency -1949.9
Nucleus 13C
Acquired Size 32768
Spectral Size 65536
f1 (ppm)

Origin	Bruker BioSpin GmbH
Solvent	CDCl3
Temperature	295.6
Pulse Sequence	zg30
Experiment	1D
Number of Scans	16
Acquisition Date	2023-10-17T19:27:30
Spectrometer Frequency	400.13
Spectral Width	8012.8
Lowest Frequency	-1548.1
Nucleus	1H
Acquired Size	32768
Spectral Size	65536

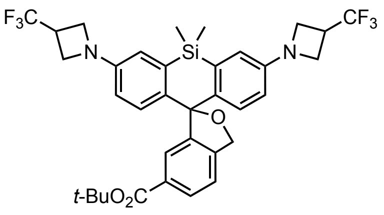

S15

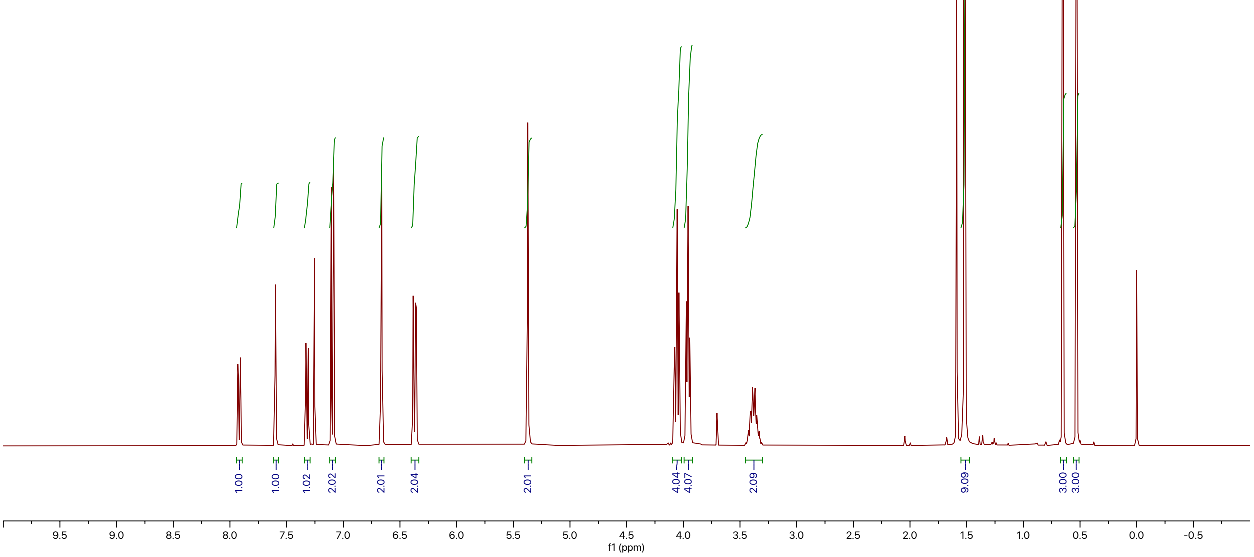

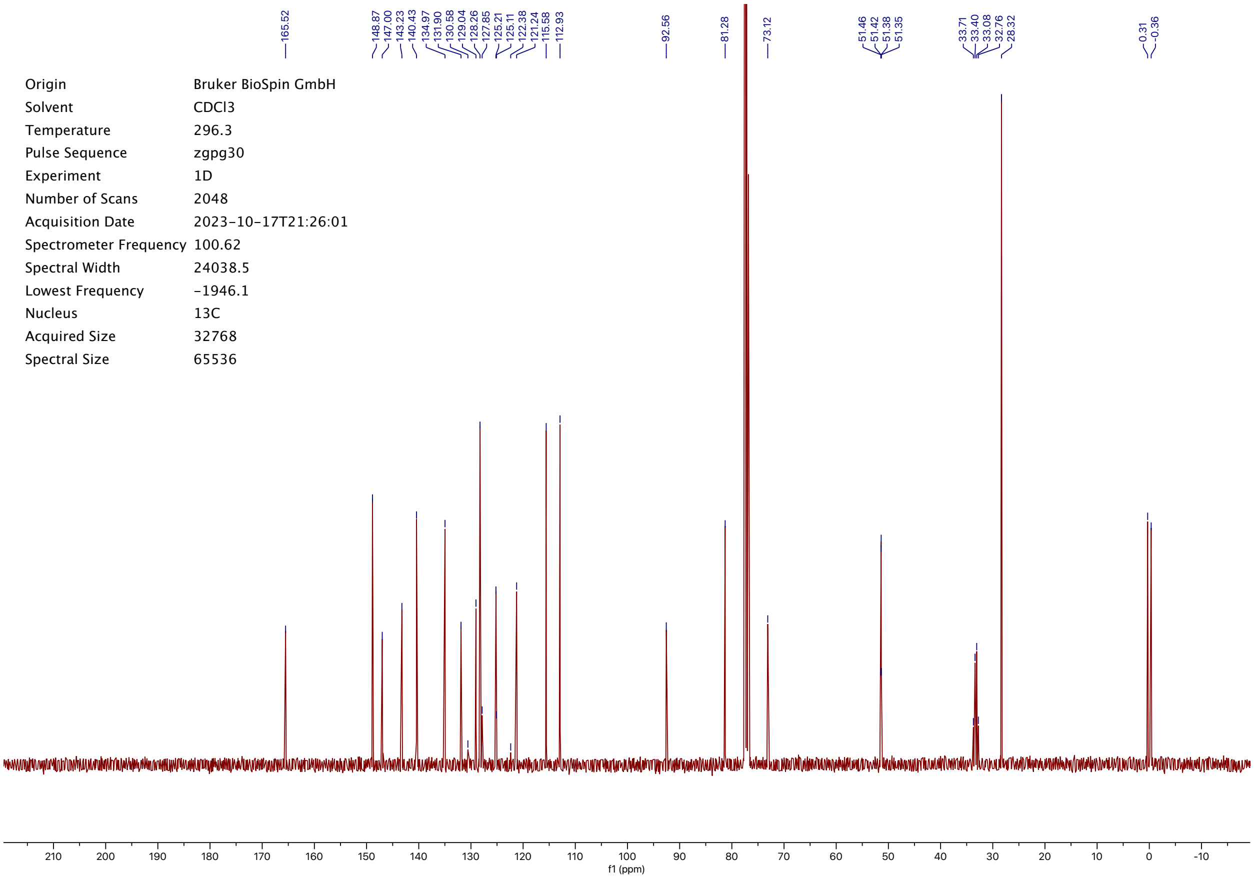

Origin	Bruker BioSpin GmbH
Solvent	CDCl3
Temperature	296.3
Pulse Sequence	zgpg30
Experiment	1D
Number of Scans	2048
Acquisition Date	2023-10-17T21:26:01
Spectrometer Frequency	100.62
Spectral Width	24038.5
Lowest Frequency	-1946.1
Nucleus	13C
Acquired Size	32768
Spectral Size	65536

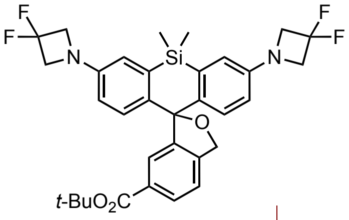

S16

Origin	Bruker BioSpin GmbH
Solvent	CDCl3
Temperature	295.5
Pulse Sequence	zg30
Experiment	1D
Number of Scans	16
Acquisition Date	2023-10-17T16:46:48
Spectrometer Frequency	400.13
Spectral Width	8012.8
Lowest Frequency	-1548.4
Nucleus	1H
Acquired Size	32768
Spectral Size	65536

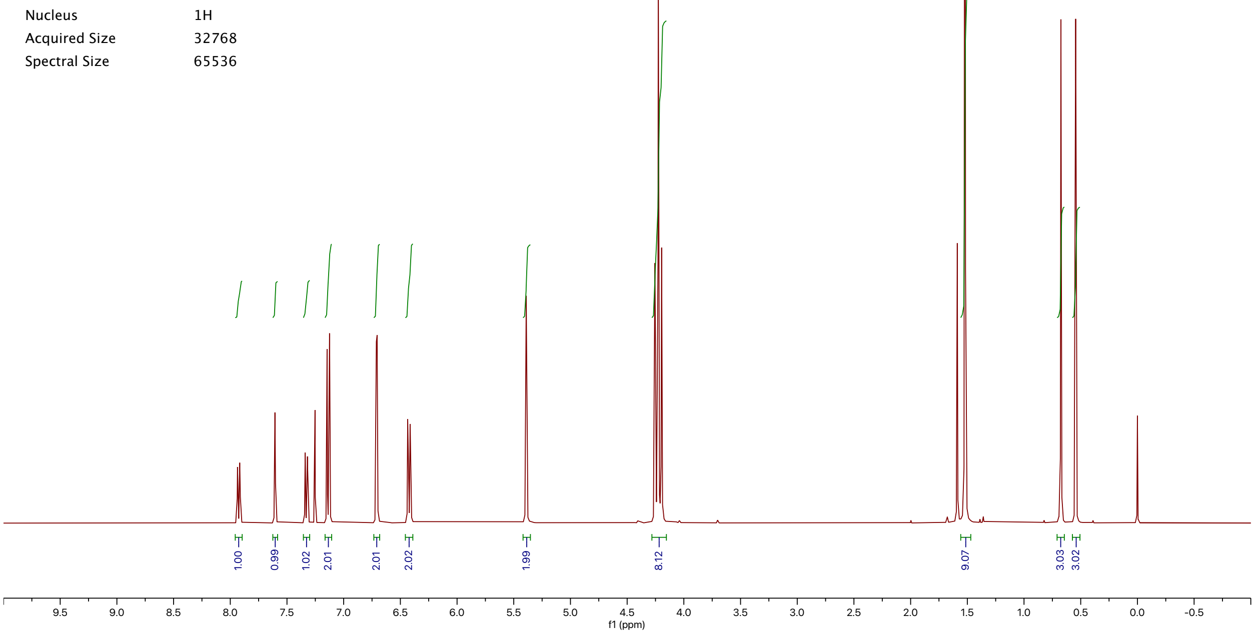

Origin	Bruker BioSpin GmbH
Solvent	CDCl3
Temperature	296.2
Pulse Sequence	zgpg30
Experiment	1D
Number of Scans	2048
Acquisition Date	2023-10-17T18:45:14
Spectrometer Frequency	100.62
Spectral Width	24038.5
Lowest Frequency	-1946.4
Nucleus	13C
Acquired Size	32768
Spectral Size	65536

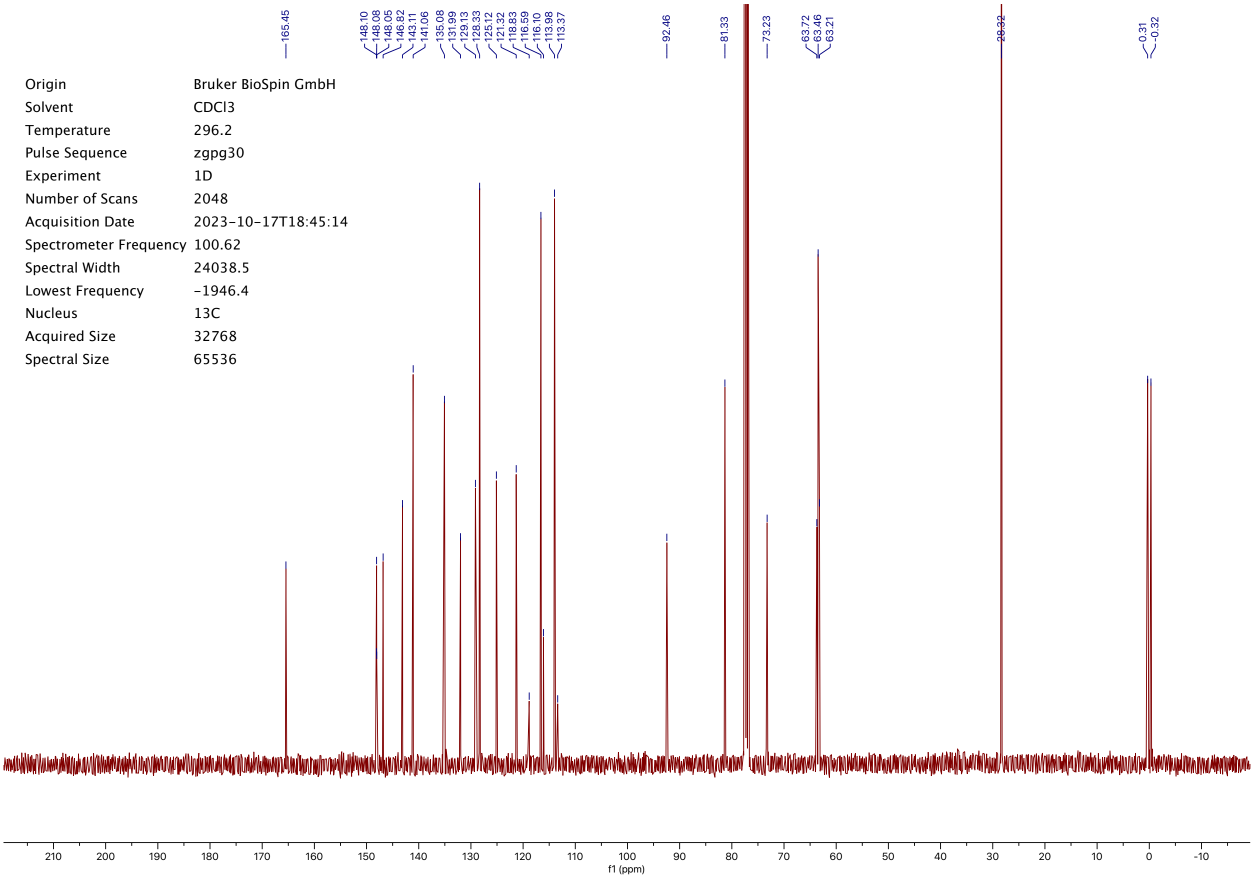

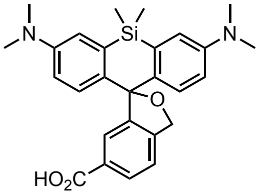

S17

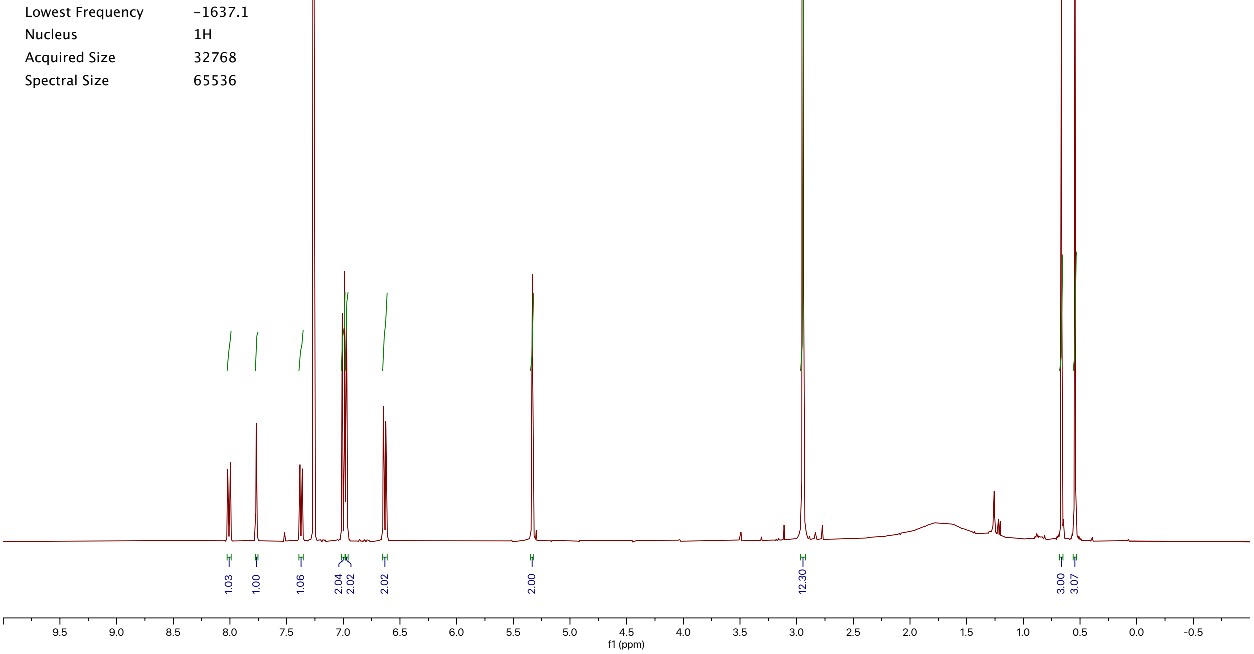

Origin	Bruker BioSpin GmbH
Solvent	CDCl3
Temperature	303.0
Pulse Sequence	zg30
Experiment	1D
Number of Scans	16
Acquisition Date	2024-06-27T11:46:08
Spectrometer Frequency	400.13
Spectral Width	8196.7
Lowest Frequency	-1637.1
Nucleus	1H
Acquired Size	32768
Spectral Size	65536

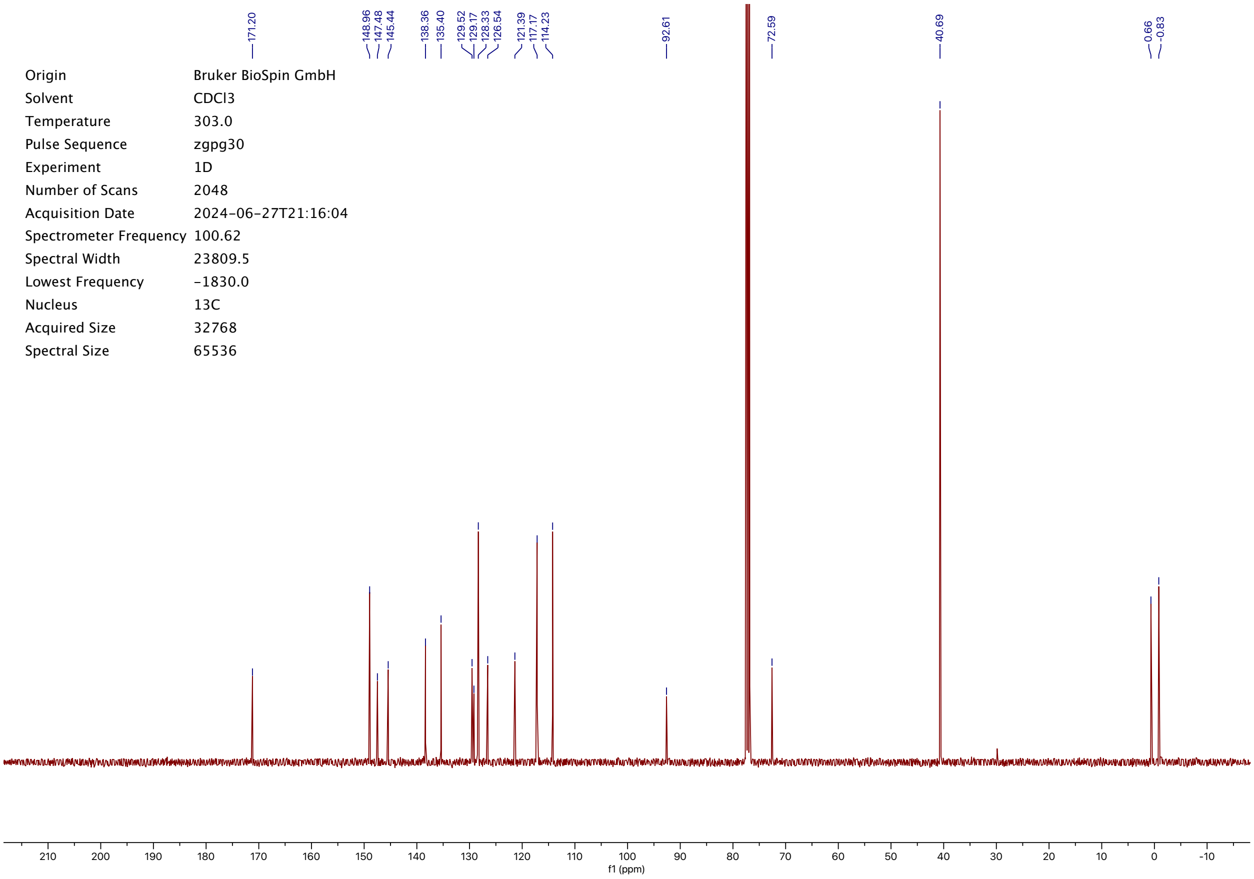

Origin	Bruker BioSpin GmbH
Solvent	CDCl3
Temperature	303.0
Pulse Sequence	zgpg30
Experiment	1D
Number of Scans	2048
Acquisition Date	2024-06-27T21:16:04
Spectrometer Frequency	100.62
Spectral Width	23809.5
Lowest Frequency	-1830.0
Nucleus	13C
Acquired Size	32768
Spectral Size	65536

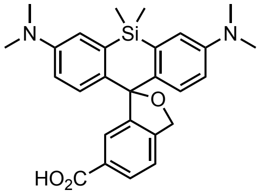

N
Si
N
O
HO2C

S17

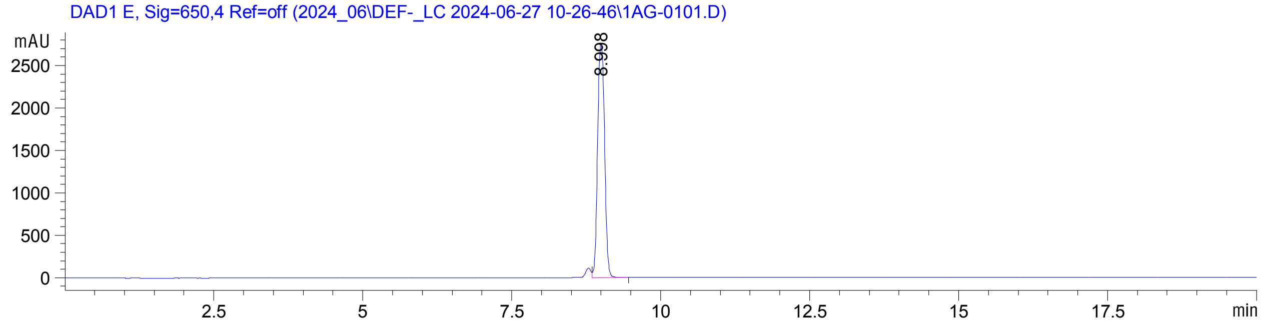

DAD1 E, Sig=650,4 Ref=off (2024_06\DEF-_LC 2024-06-27 10-26-46\1AG-0101.D)
mAU
2500
2000
1500
1000
500
0
8.998
2.5
5
7.5
10
12.5
15
17.5
min

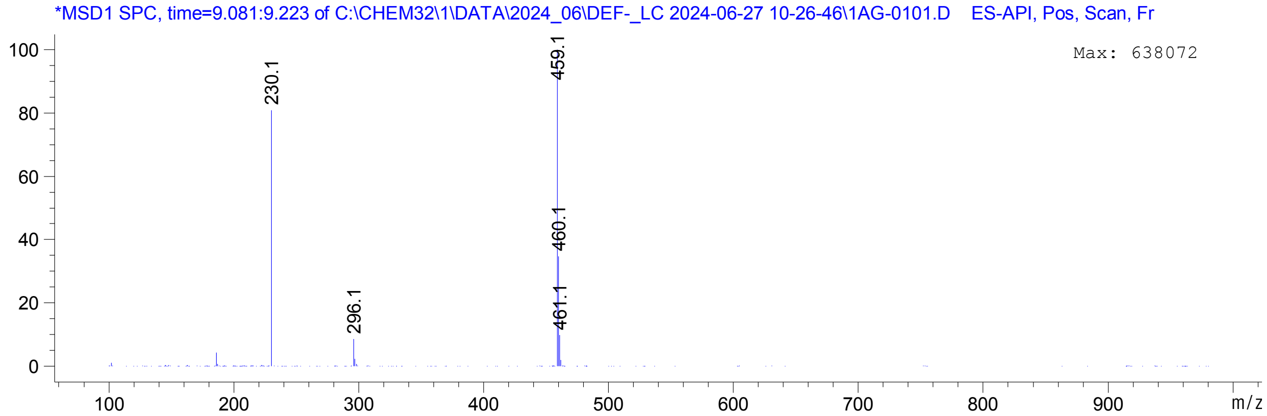

*MSD1 SPC, time=9.081:9.223 of C:\CHEM32\1\DATA\2024_06\DEF-_LC 2024-06-27 10-26-46\1AG-0101.D ES-API, Pos, Scan, Fr
Max: 638072
100
80
60
40
20
0
230.1
296.1
459.1
460.1
461.1
100
200
300
400
500
600
700
800
900
m/z

Origin	Bruker BioSpin GmbH
Solvent	CDCl3
Temperature	295.4
Pulse Sequence	zg30
Experiment	1D
Number of Scans	16
Acquisition Date	2023-10-17T09:41:07
Spectrometer Frequency	400.13
Spectral Width	8012.8
Lowest Frequency	-1573.2
Nucleus	1H
Acquired Size	32768
Spectral Size	65536

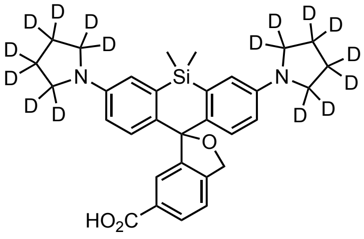

S18

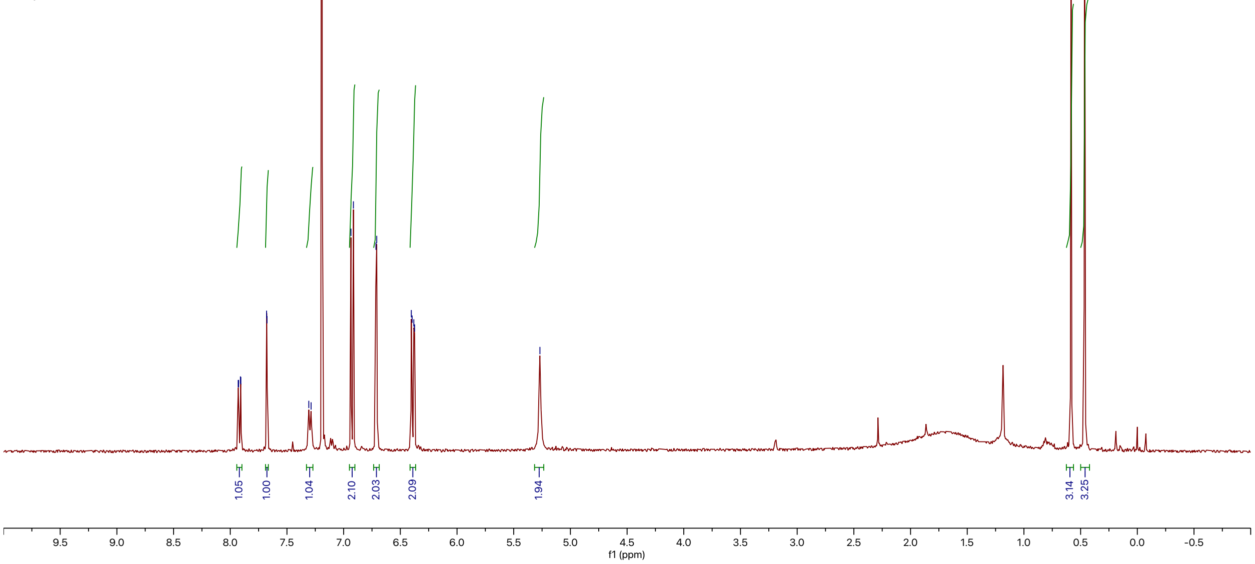

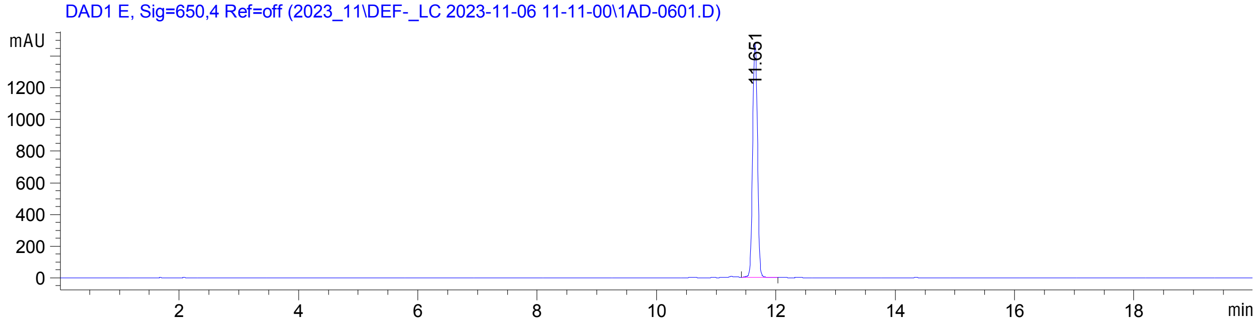

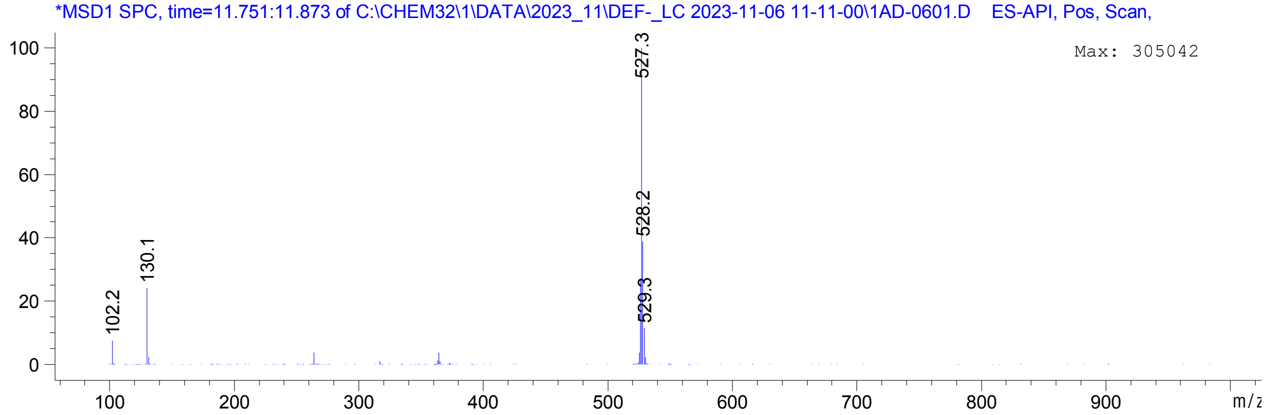

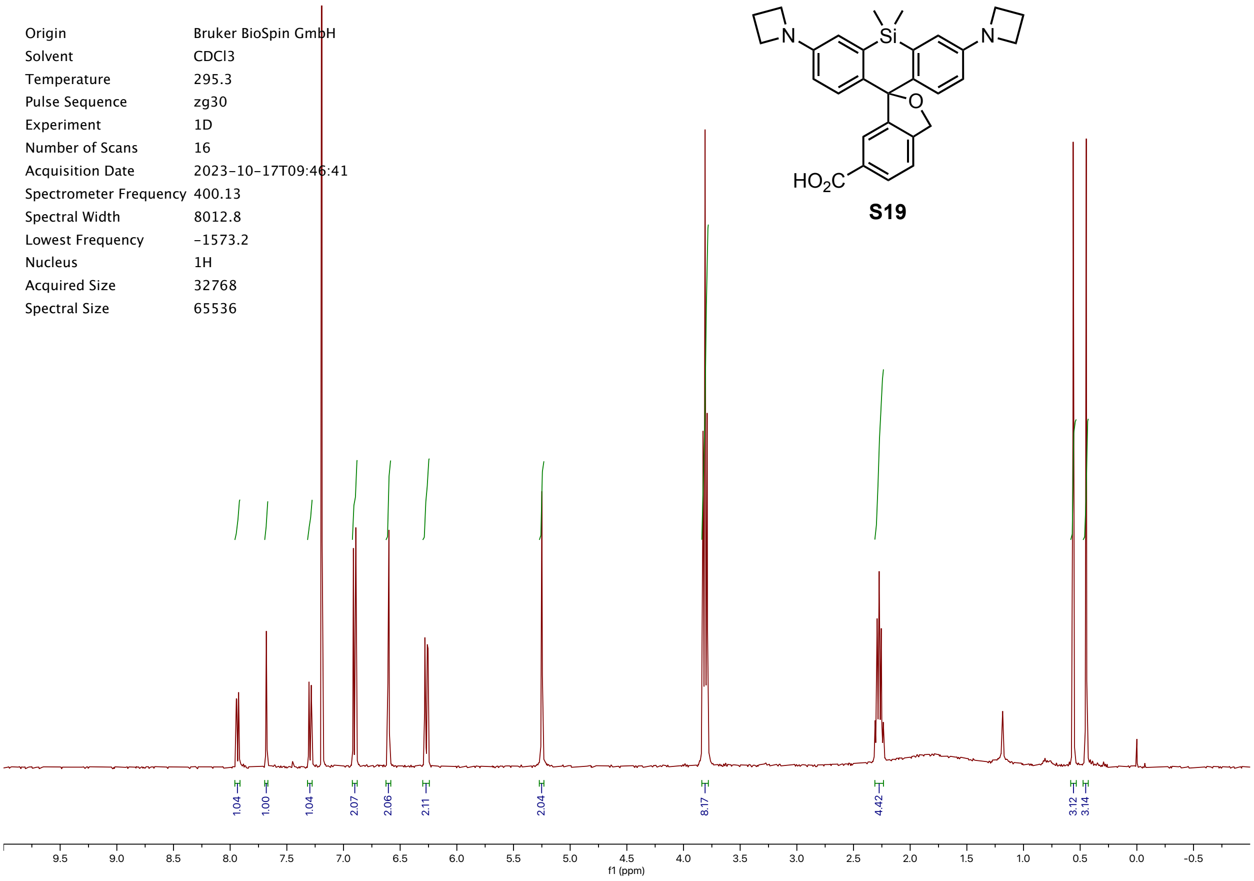
Origin Bruker BioSpin GmbH
Solvent CDCl3
Temperature 295.3
Pulse Sequence zg30
Experiment 1D
Number of Scans 16
Acquisition Date 2023-10-17T09:46:41
Spectrometer Frequency 400.13
Spectral Width 8012.8
Lowest Frequency -1573.2
Nucleus 1H
Acquired Size 32768
Spectral Size 65536
1.04
1.00
1.04
2.07
2.06
2.11
2.04
8.17
4.42
3.12
3.14
9.5 9.0 8.5 8.0 7.5 7.0 6.5 6.0 5.5 5.0 4.5 4.0 3.5 3.0 2.5 2.0 1.5 1.0 0.5 0.0 -0.5
f1 (ppm)
Si
N
N
O
HO_2C

S19

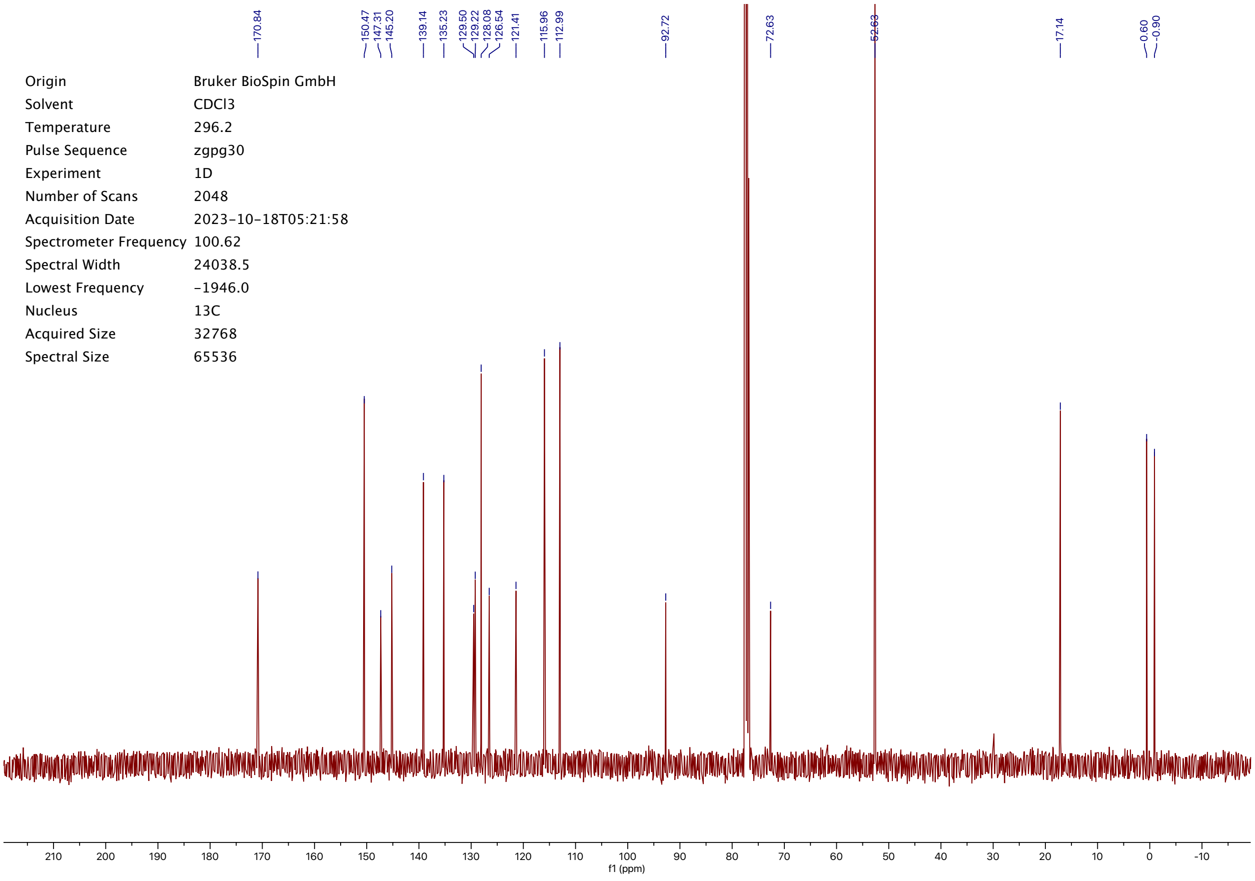
170.84
150.47
147.31
145.20
139.14
135.23
129.50
129.22
128.08
126.54
121.41
115.96
112.99
92.72
72.63
52.63
17.14
0.60
-0.90
Origin Bruker BioSpin GmbH
Solvent CDCl3
Temperature 296.2
Pulse Sequence zgpg30
Experiment 1D
Number of Scans 2048
Acquisition Date 2023-10-18T05:21:58
Spectrometer Frequency 100.62
Spectral Width 24038.5
Lowest Frequency -1946.0
Nucleus 13C
Acquired Size 32768
Spectral Size 65536
210 200 190 180 170 160 150 140 130 120 110 100 90 80 70 60 50 40 30 20 10 0 -10
f1 (ppm)

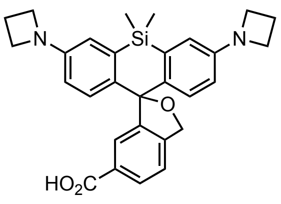
N
Si
N
O
HO_2C

S19

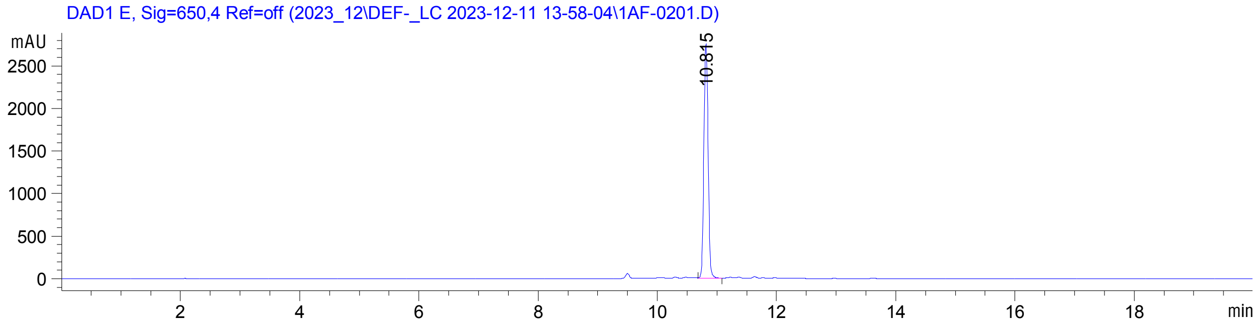
DAD1 E, Sig=650,4 Ref=off (2023_12\DEF-_LC 2023-12-11 13-58-04\1AF-0201.D)
mAU
2500
2000
1500
1000
500
0
10.815
2
4
6
8
10
12
14
16
18
min

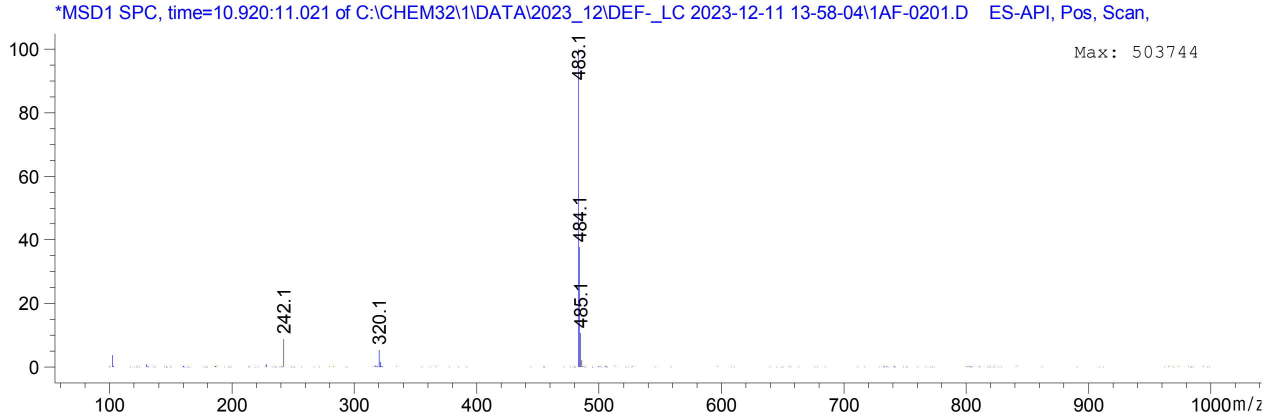
*MSD1 SPC, time=10.920:11.021 of C:\CHEM32\1\DATA\2023_12\DEF-_LC 2023-12-11 13-58-04\1AF-0201.D ES-API, Pos, Scan,
Max: 503744
100
80
60
40
20
0
483.1
484.1
485.1
242.1
320.1
100
200
300
400
500
600
700
800
900
1000
m/z

Origin	Bruker BioSpin GmbH
Solvent	CDCl3
Temperature	295.4
Pulse Sequence	zg30
Experiment	1D
Number of Scans	16
Acquisition Date	2023-10-19T09:12:35
Spectrometer Frequency	400.13
Spectral Width	8012.8
Lowest Frequency	-1545.7
Nucleus	1H
Acquired Size	32768
Spectral Size	65536

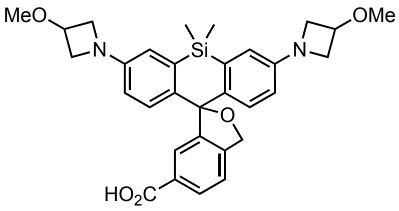

S20

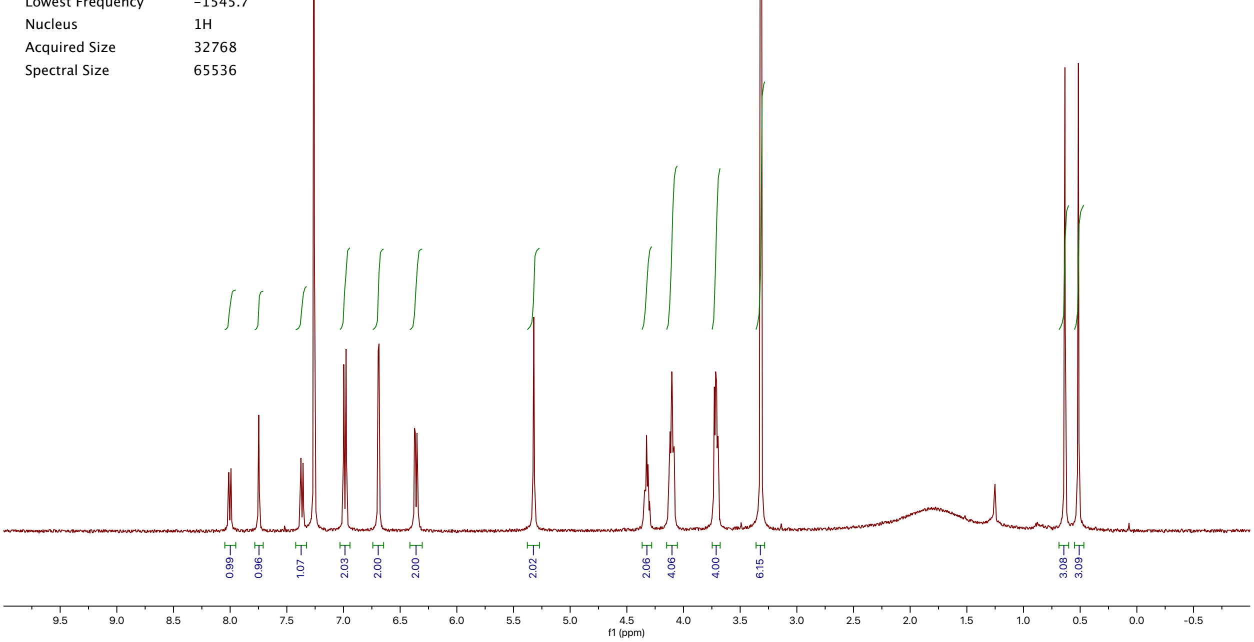

Origin	Bruker BioSpin GmbH
Solvent	CDCl3
Temperature	296.1
Pulse Sequence	zgpg30
Experiment	1D
Number of Scans	2048
Acquisition Date	2023-10-23T20:04:25
Spectrometer Frequency	100.62
Spectral Width	24038.5
Lowest Frequency	-1946.7
Nucleus	13C
Acquired Size	32768
Spectral Size	65536

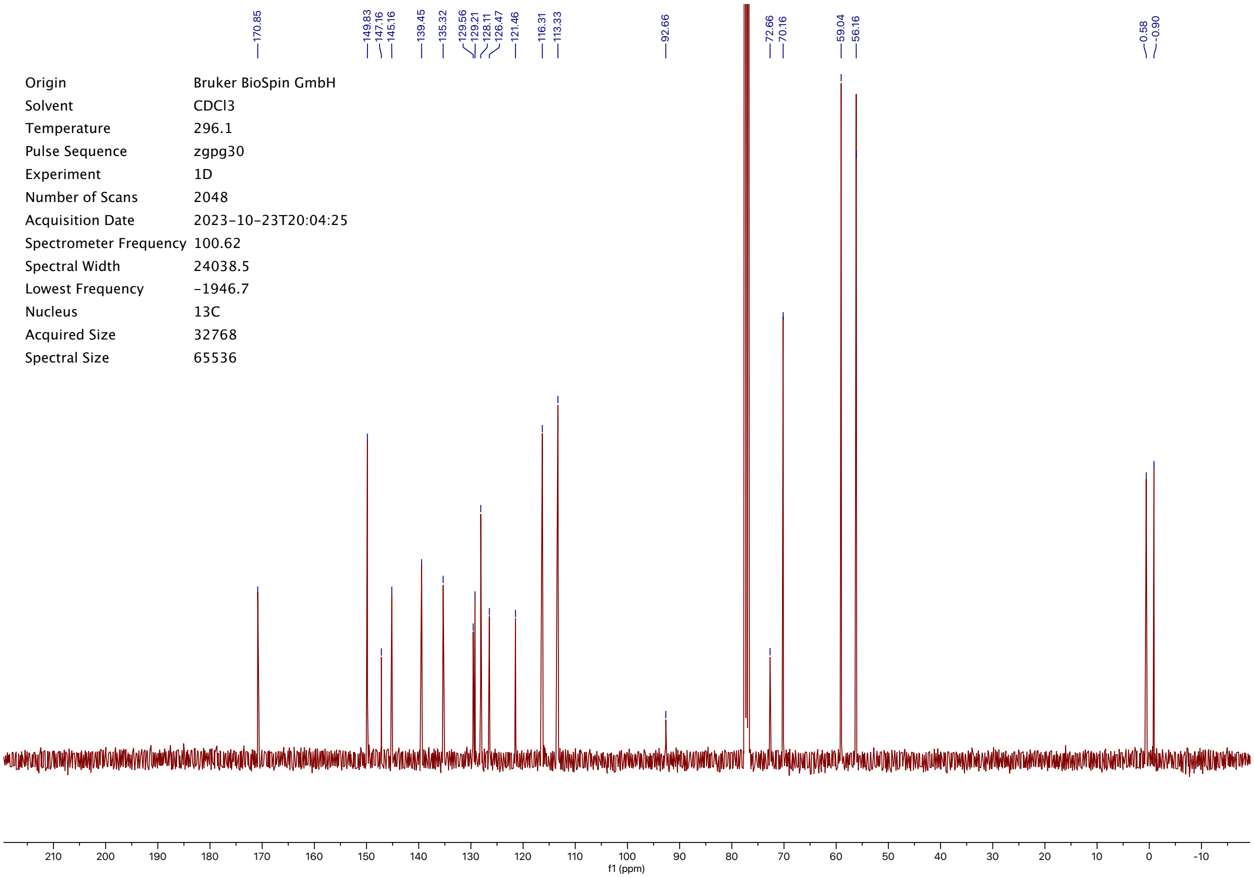

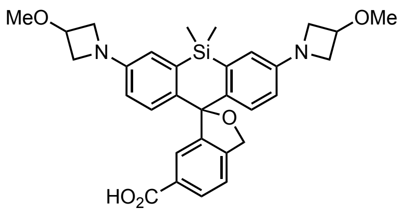
MeO
OMe
Si
N
N
O
HO2C

S20

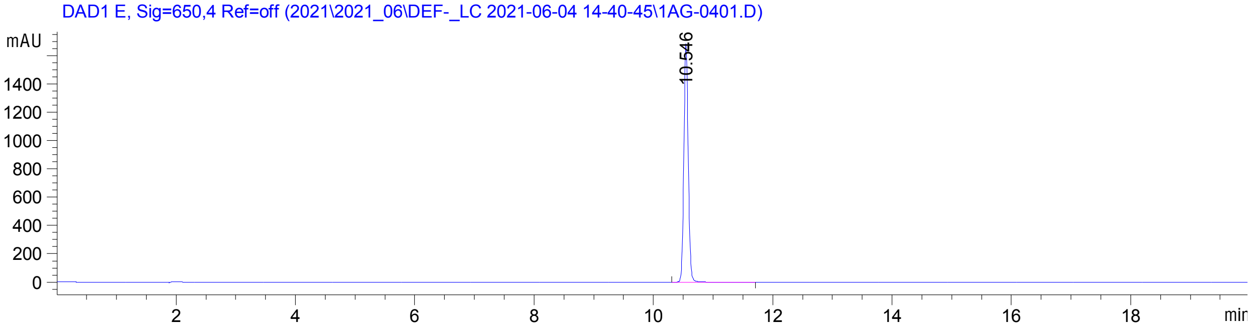
DAD1 E, Sig=650,4 Ref=off (2021\2021_06\DEF-_LC 2021-06-04 14-40-45\1AG-0401.D)
mAU
10.546
min

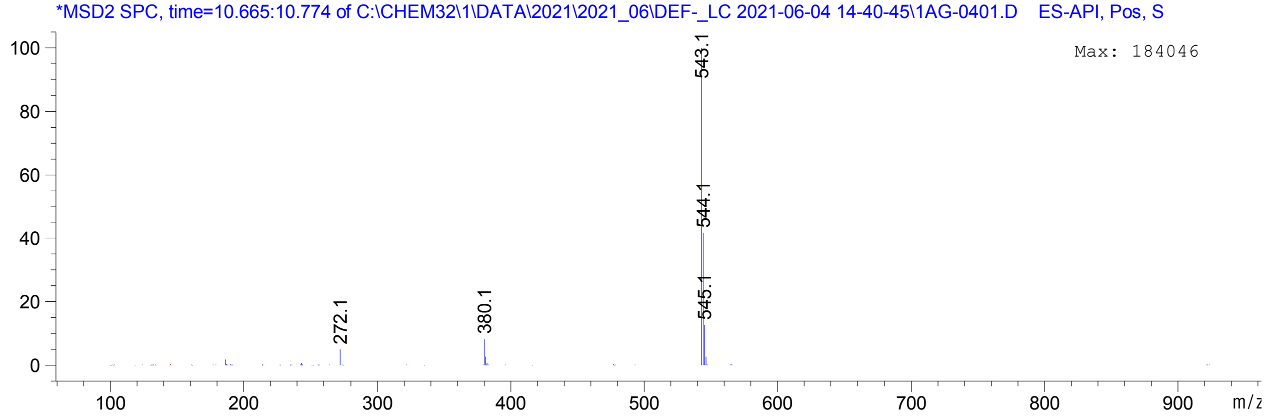
*MSD2 SPC, time=10.665:10.774 of C:\CHEM32\1\DATA\2021\2021_06\DEF-_LC 2021-06-04 14-40-45\1AG-0401.D ES-API, Pos, S
Max: 184046
543.1
544.1
545.1
272.1
380.1
m/z

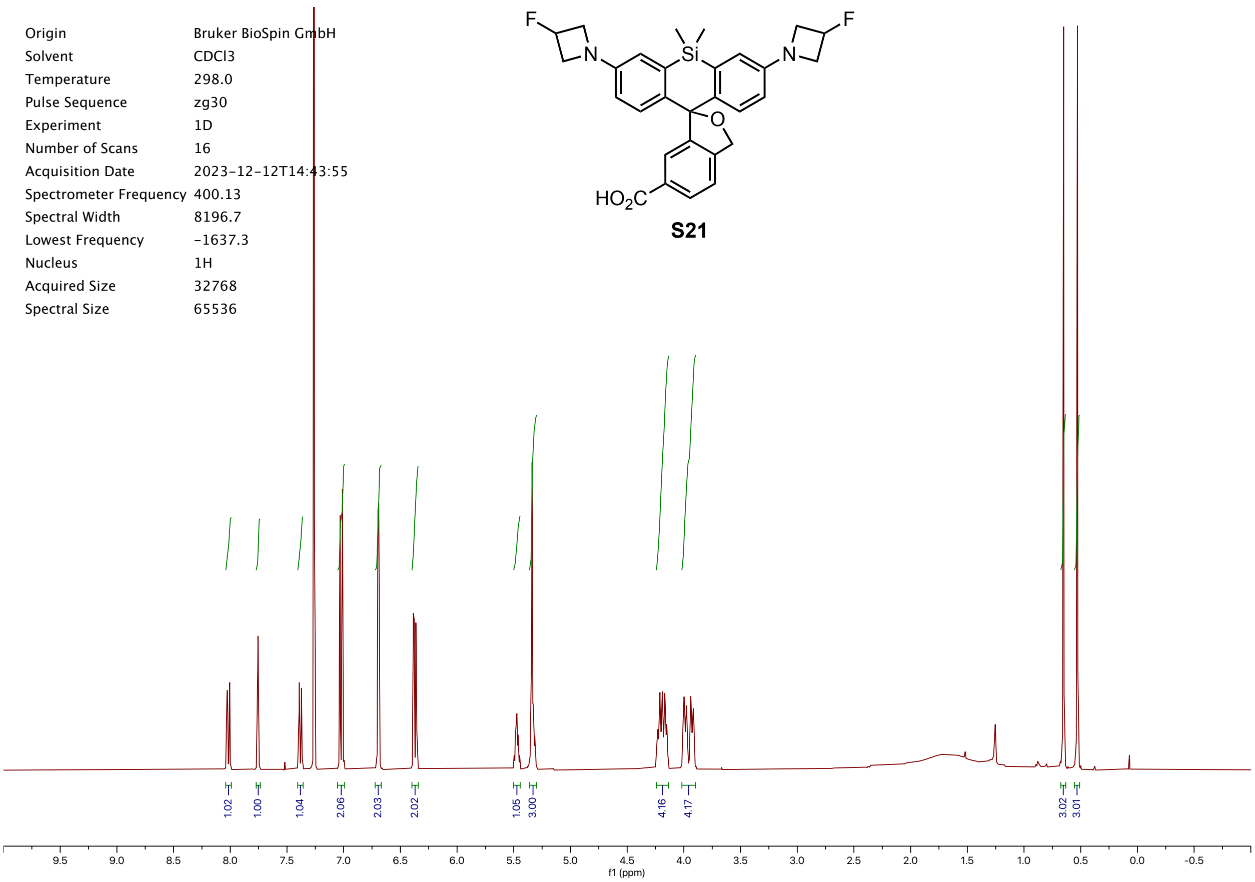
Origin Bruker BioSpin GmbH
Solvent CDCl3
Temperature 298.0
Pulse Sequence zg30
Experiment 1D
Number of Scans 16
Acquisition Date 2023-12-12T14:43:55
Spectrometer Frequency 400.13
Spectral Width 8196.7
Lowest Frequency -1637.3
Nucleus 1H
Acquired Size 32768
Spectral Size 65536
S21
f1 (ppm)

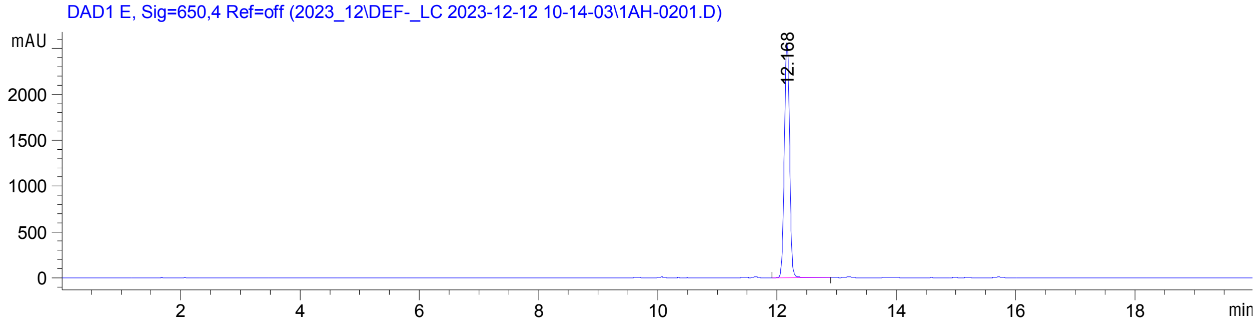
DAD1 E, Sig=650,4 Ref=off (2023_12\DEF-_LC 2023-12-12 10-14-03\1AH-0201.D)
mAU
12.168
min

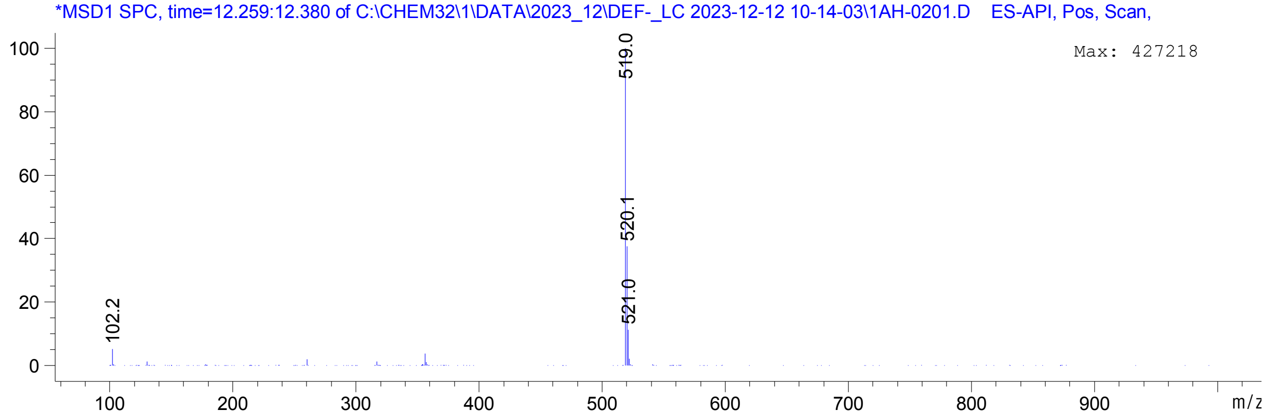
*MSD1 SPC, time=12.259:12.380 of C:\CHEM32\1\DATA\2023_12\DEF-_LC 2023-12-12 10-14-03\1AH-0201.D ES-API, Pos, Scan,
Max: 427218
519.0
520.1
521.0
102.2
m/z

Origin	Bruker BioSpin GmbH
Solvent	CDCl3
Temperature	295.3
Pulse Sequence	zg30
Experiment	1D
Number of Scans	16
Acquisition Date	2023-10-18T10:01:26
Spectrometer Frequency	400.13
Spectral Width	8012.8
Lowest Frequency	-1545.6
Nucleus	1H
Acquired Size	32768
Spectral Size	65536

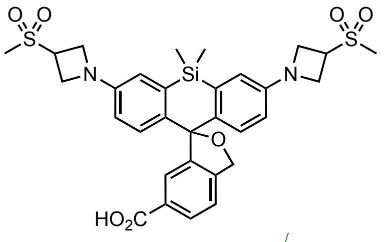

S22

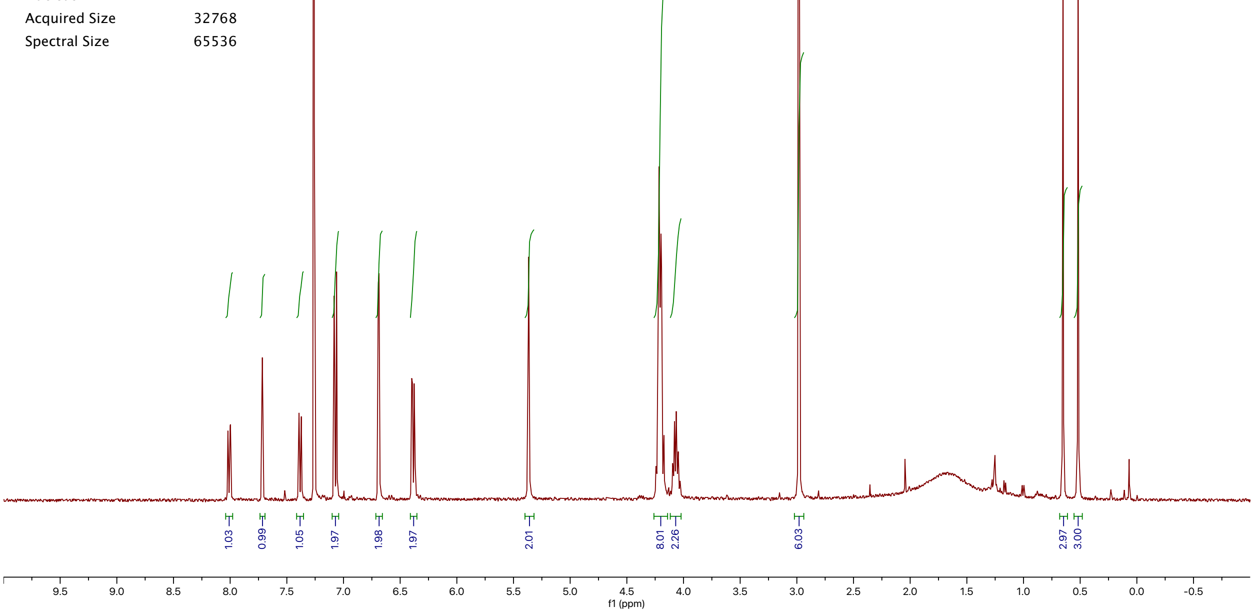

DAD1 E, Sig=650,4 Ref=off (2023_10\DEF-_LC 2023-10-23 09-38-16\1AA-0101.D)

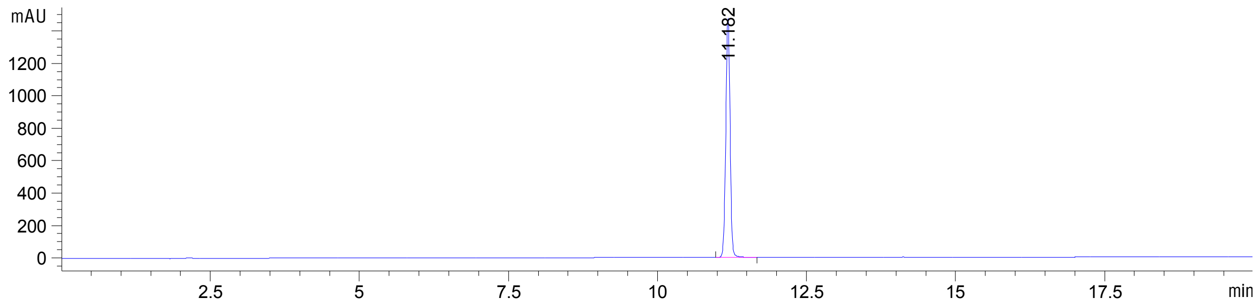

*MSD2 SPC, time=11.207:11.424 of C:\CHEM32\1\DATA\2023_10\DEF-_LC 2023-10-23 09-38-16\1AA-0101.D ES-API, Pos, Scan,

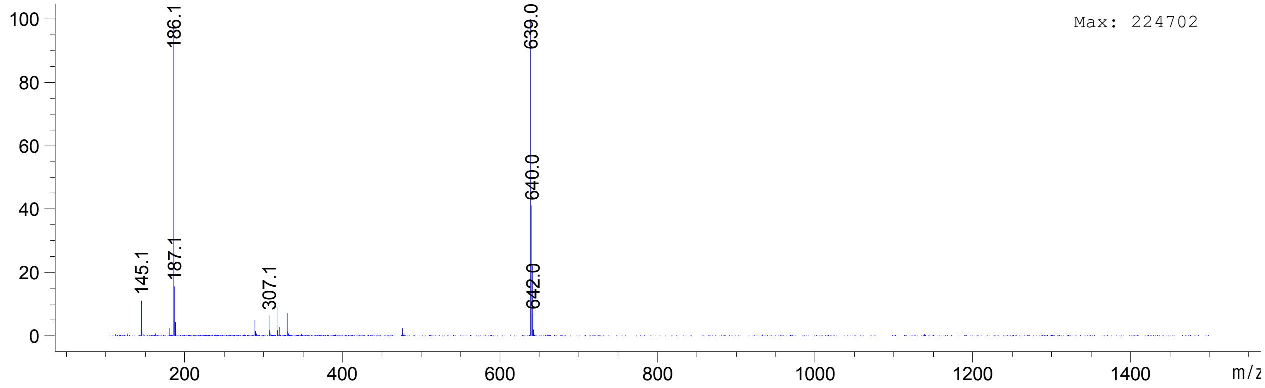

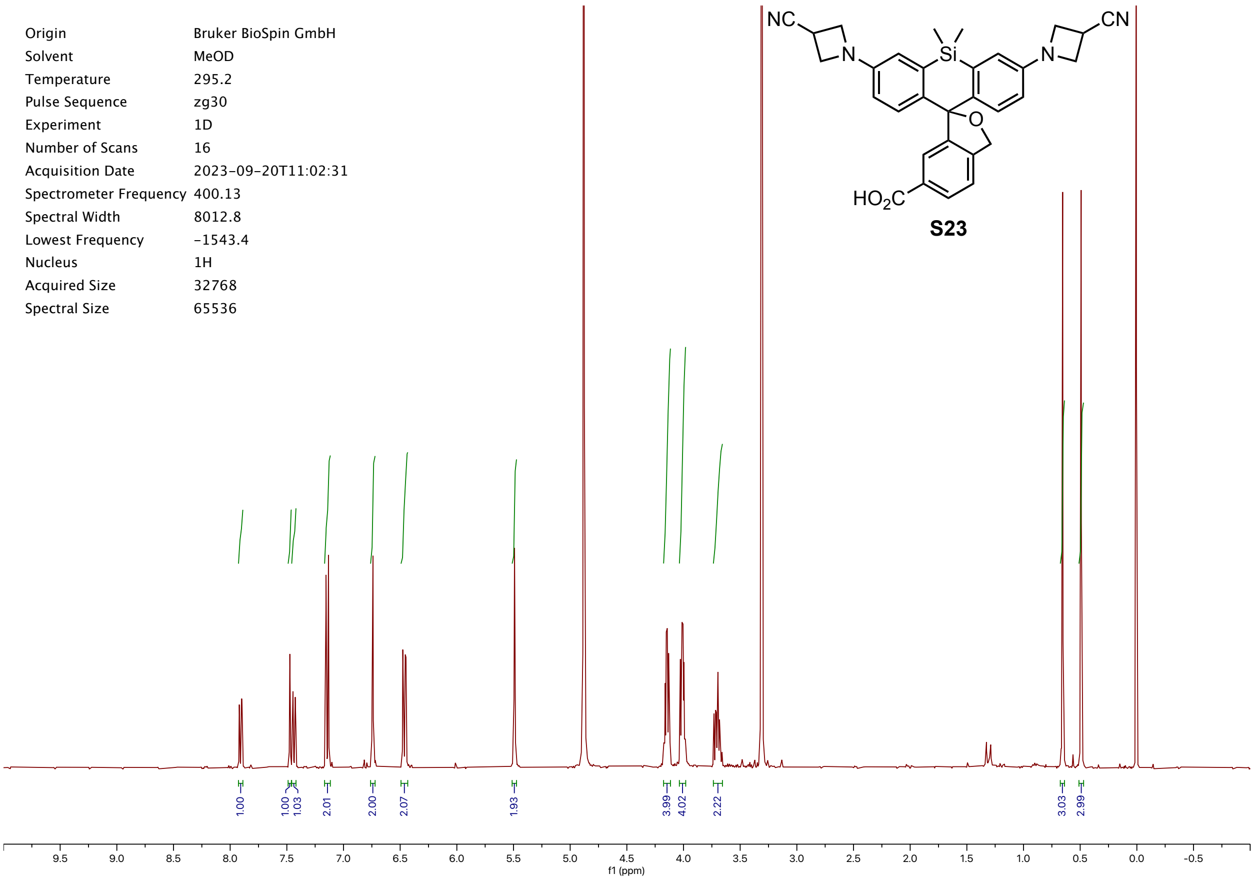
Origin Bruker BioSpin GmbH
Solvent MeOD
Temperature 295.2
Pulse Sequence zg30
Experiment 1D
Number of Scans 16
Acquisition Date 2023-09-20T11:02:31
Spectrometer Frequency 400.13
Spectral Width 8012.8
Lowest Frequency -1543.4
Nucleus 1H
Acquired Size 32768
Spectral Size 65536
NC
CN
N
N
Si
O
HO2C
S23
1.00
1.00
1.03
2.01
2.00
2.07
1.93
3.99
4.02
2.22
3.03
2.99
9.5 9.0 8.5 8.0 7.5 7.0 6.5 6.0 5.5 5.0 4.5 4.0 3.5 3.0 2.5 2.0 1.5 1.0 0.5 0.0 -0.5
f1 (ppm)

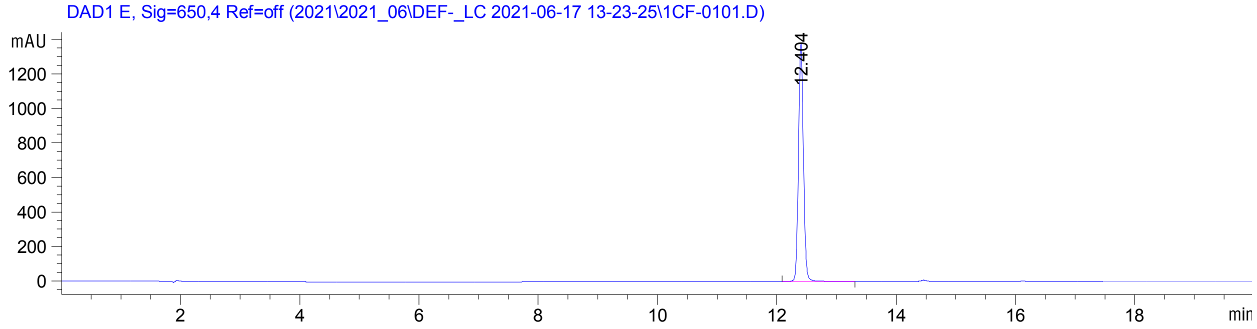
DAD1 E, Sig=650,4 Ref=off (2021\2021_06\DEF-_LC 2021-06-17 13-23-25\1CF-0101.D)
mAU
1200
1000
800
600
400
200
0
12.404
2 4 6 8 10 12 14 16 18
min

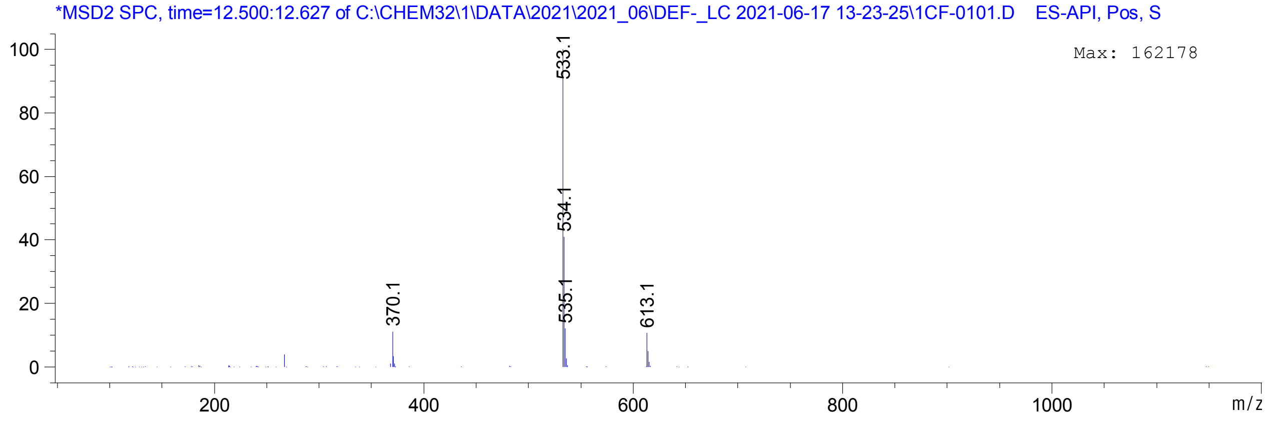
*MSD2 SPC, time=12.500:12.627 of C:\CHEM32\1\DATA\2021\2021_06\DEF-_LC 2021-06-17 13-23-25\1CF-0101.D ES-API, Pos, S
Max: 162178
100
80
60
40
20
0
533.1
534.1
535.1
370.1
613.1
200 400 600 800 1000
m/z

Origin	Bruker BioSpin GmbH
Solvent	CDCl3
Temperature	298.0
Pulse Sequence	zg30
Experiment	1D
Number of Scans	16
Acquisition Date	2023-11-10T14:52:49
Spectrometer Frequency	400.13
Spectral Width	8012.8
Lowest Frequency	-1546.1
Nucleus	1H
Acquired Size	32768
Spectral Size	65536

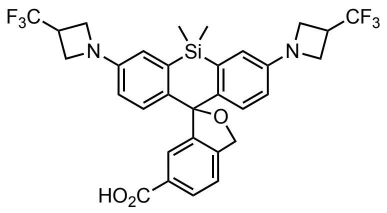

S24

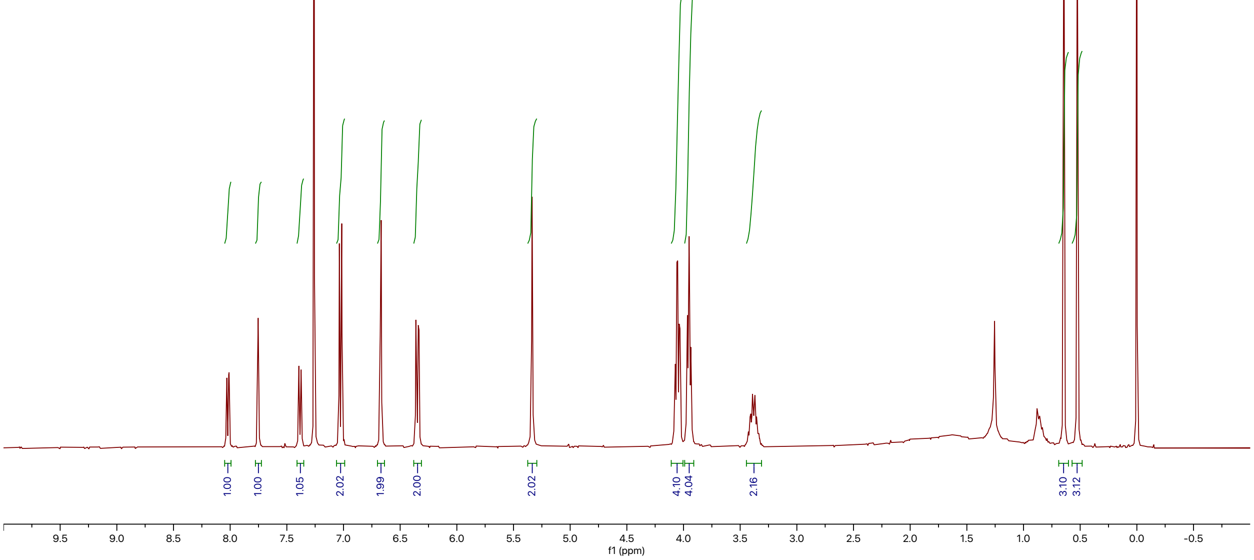

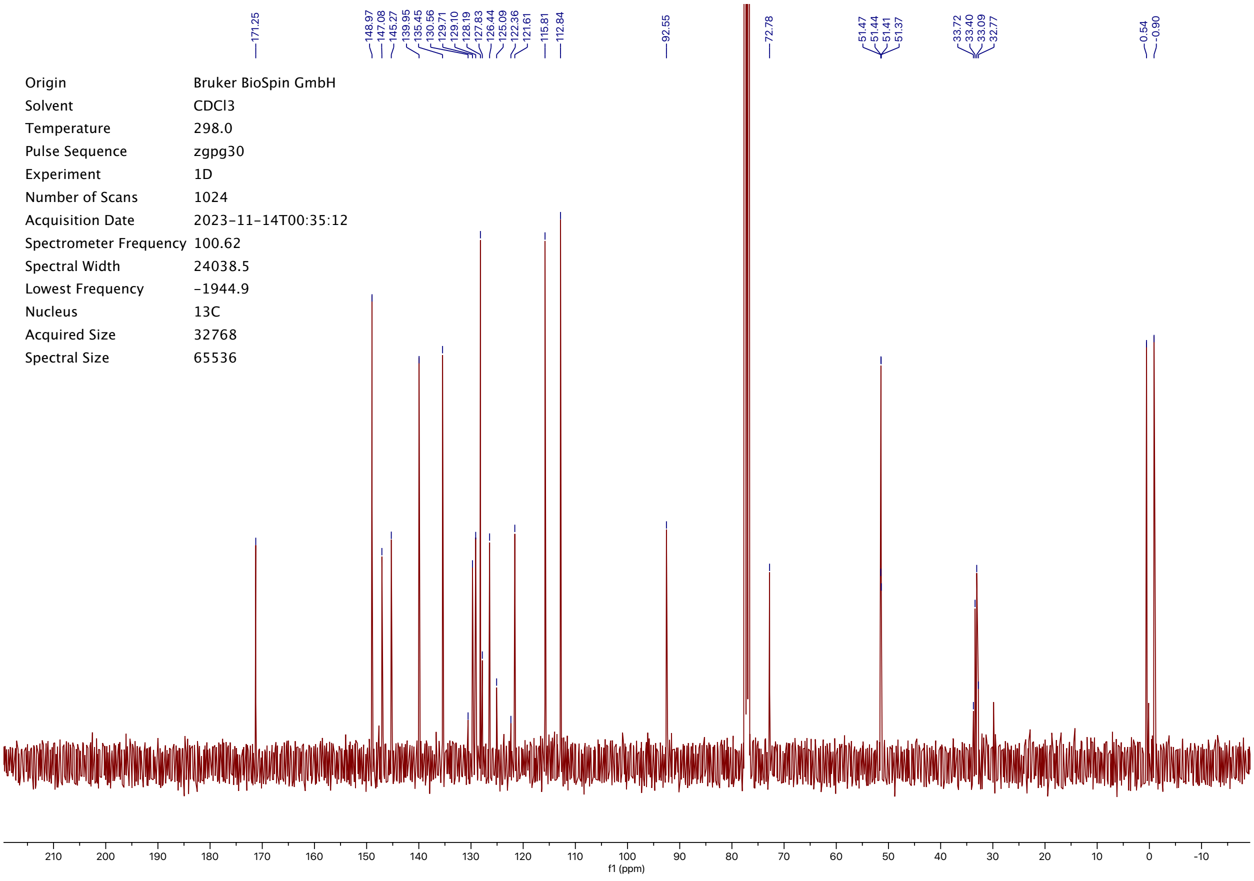

Origin	Bruker BioSpin GmbH
Solvent	CDCl3
Temperature	298.0
Pulse Sequence	zgpg30
Experiment	1D
Number of Scans	1024
Acquisition Date	2023-11-14T00:35:12
Spectrometer Frequency	100.62
Spectral Width	24038.5
Lowest Frequency	-1944.9
Nucleus	13C
Acquired Size	32768
Spectral Size	65536

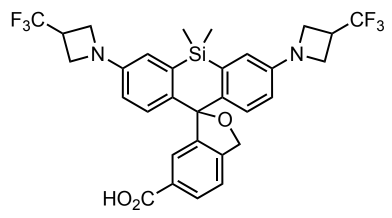
F3C
CF3
N
N
Si
O
HO2C

S24

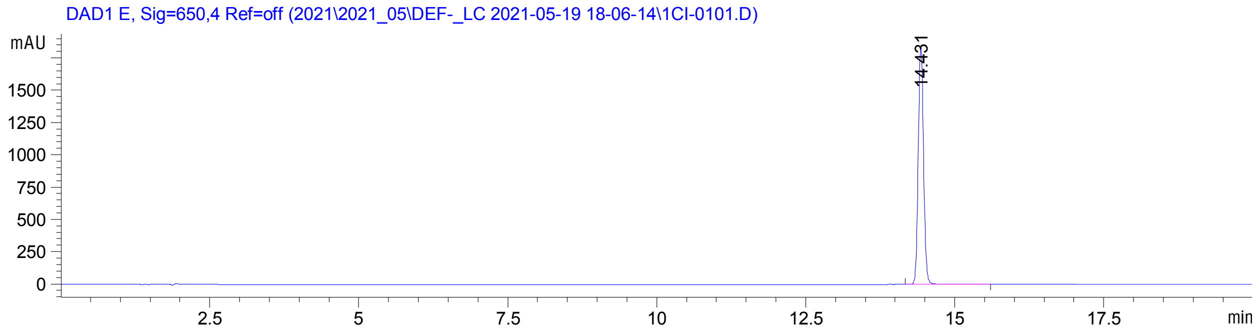
DAD1 E, Sig=650,4 Ref=off (2021\2021_05\DEF-_LC 2021-05-19 18-06-14\1CI-0101.D)
mAU
1500
1250
1000
750
500
250
0
14.431
2.5
5
7.5
10
12.5
15
17.5
min

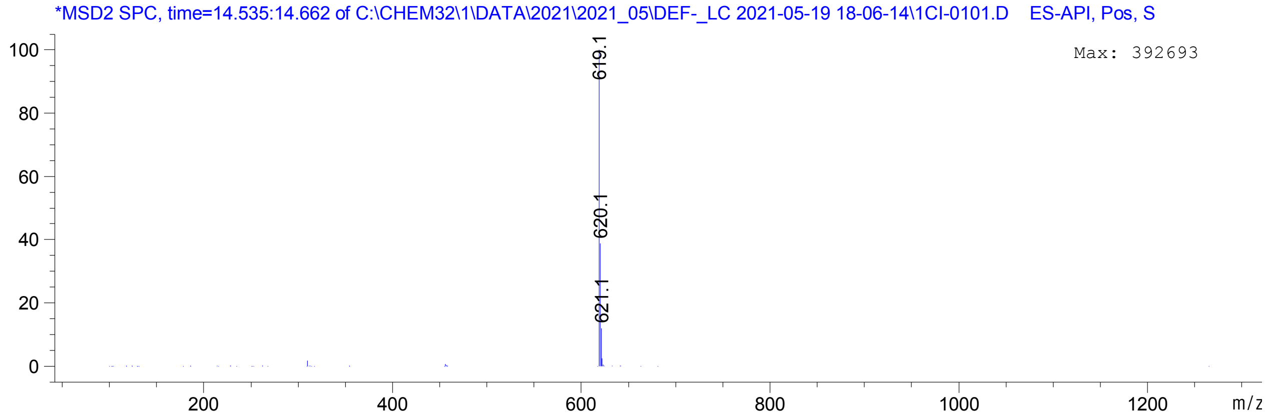
*MSD2 SPC, time=14.535:14.662 of C:\CHEM32\1\DATA\2021\2021_05\DEF-_LC 2021-05-19 18-06-14\1CI-0101.D ES-API, Pos, S
Max: 392693
100
80
60
40
20
0
619.1
620.1
621.1
200
400
600
800
1000
1200
m/z

Origin	Bruker BioSpin GmbH
Solvent	CDCl3
Temperature	298.0
Pulse Sequence	zg30
Experiment	1D
Number of Scans	16
Acquisition Date	2023-11-10T14:45:46
Spectrometer Frequency	400.13
Spectral Width	8012.8
Lowest Frequency	-1546.2
Nucleus	1H
Acquired Size	32768
Spectral Size	65536

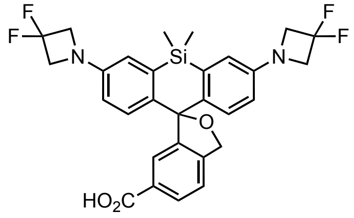

S25

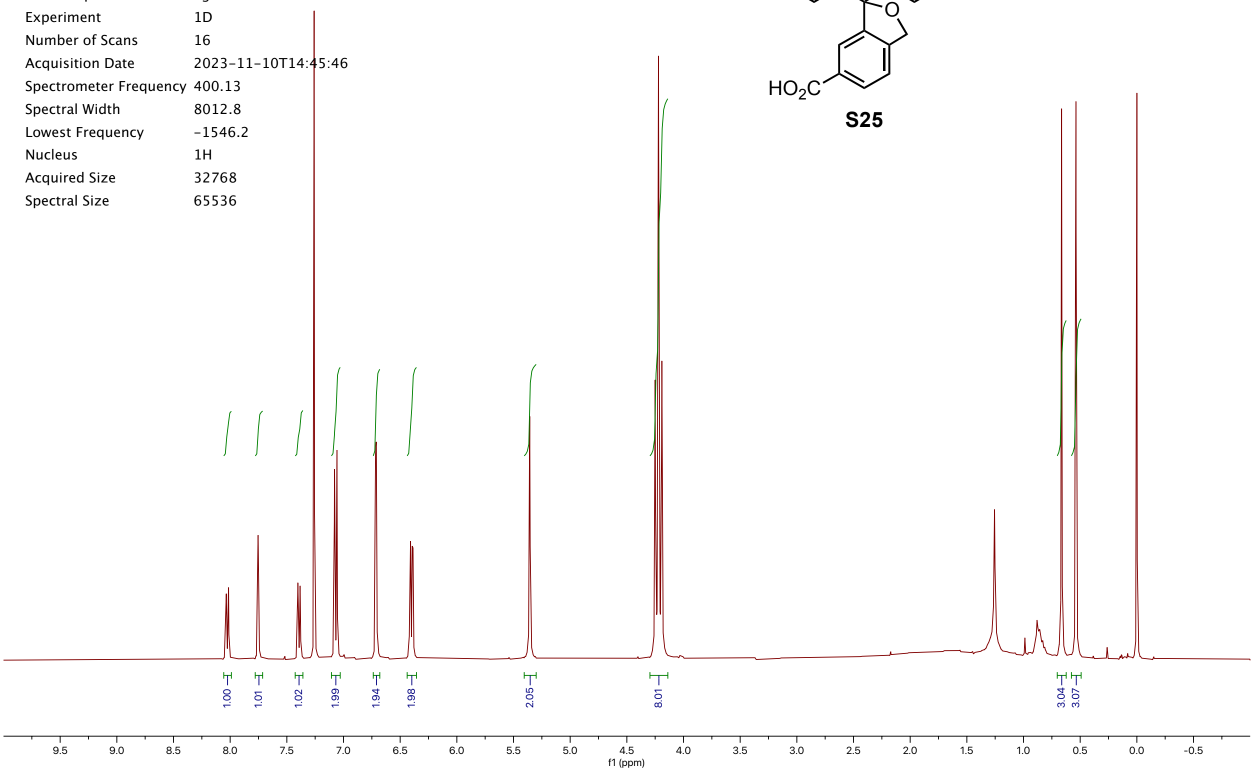

Origin	Bruker BioSpin GmbH
Solvent	CDCl3
Temperature	298.0
Pulse Sequence	zgpg30
Experiment	1D
Number of Scans	512
Acquisition Date	2023-11-14T12:56:45
Spectrometer Frequency	100.62
Spectral Width	24038.5
Lowest Frequency	-1945.5
Nucleus	13C
Acquired Size	32768
Spectral Size	65536

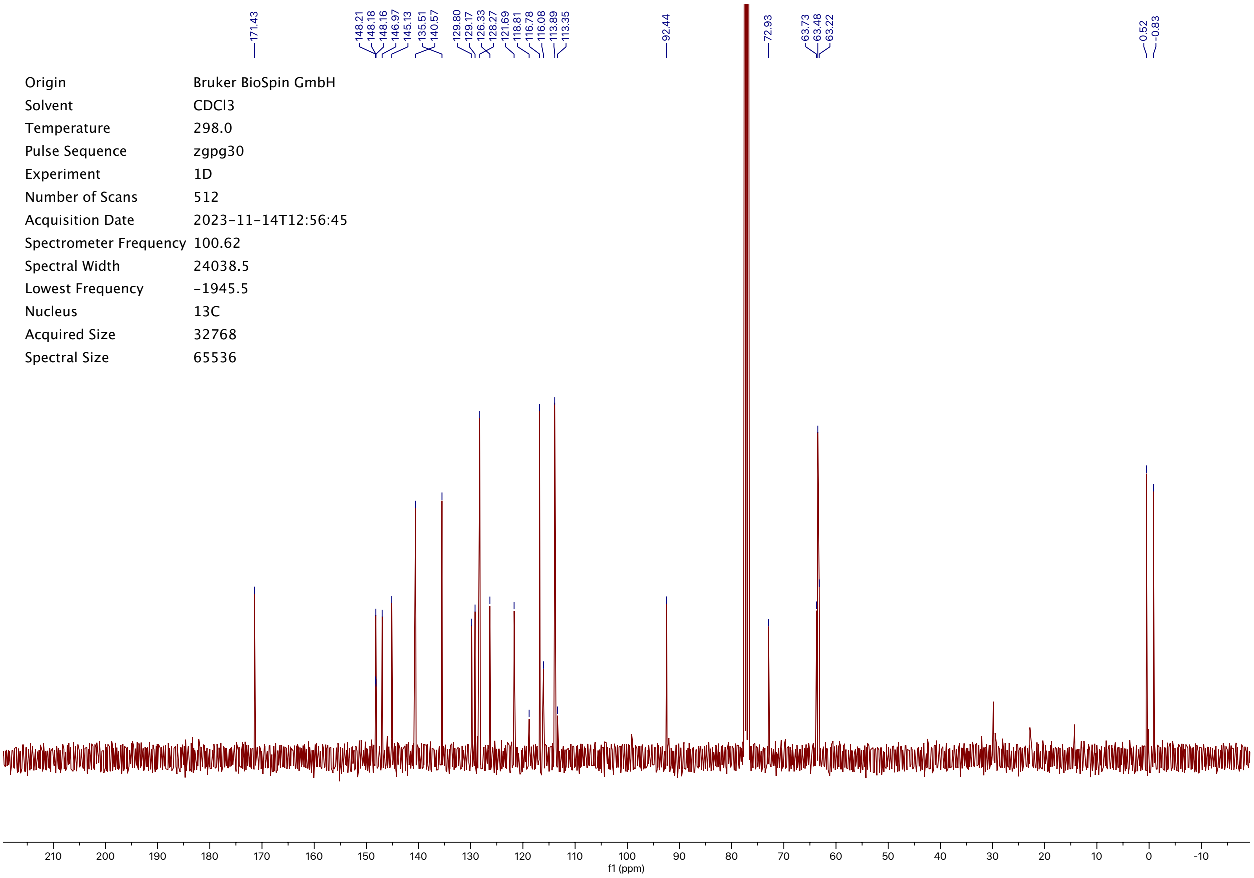

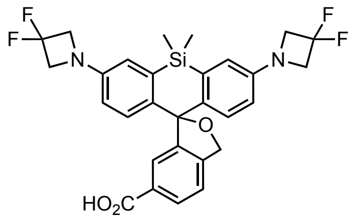

S25

DAD1 E, Sig=650,4 Ref=off (2021\2021_04\DEF-_LC 2021-04-23 12-42-30\1AE-0101.D)

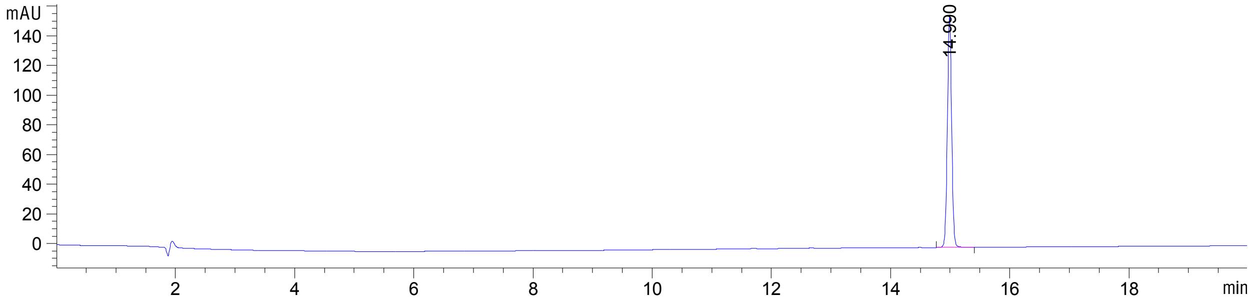

*MSD2 SPC, time=15.062:15.244 of C:\CHEM32\1\DATA\2021\2021_04\DEF-_LC 2021-04-23 12-42-30\1AE-0101.D ES-API, Pos, S

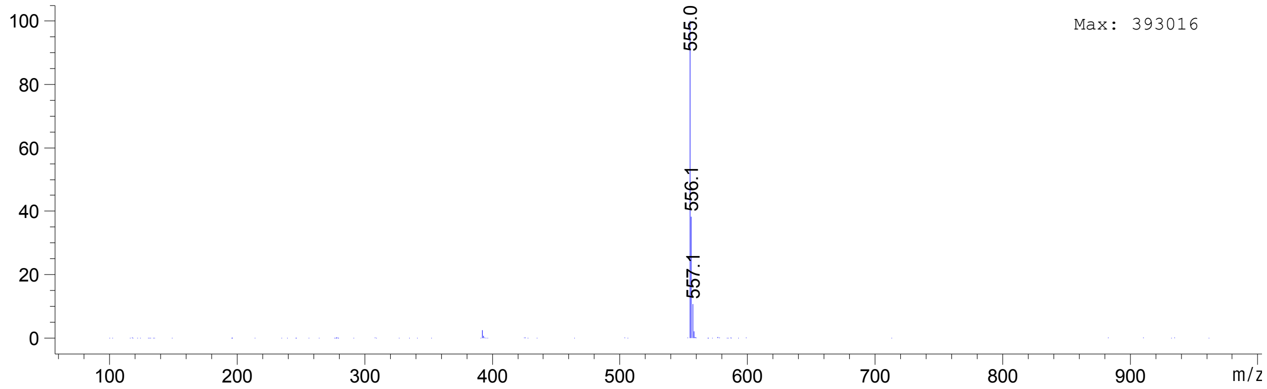

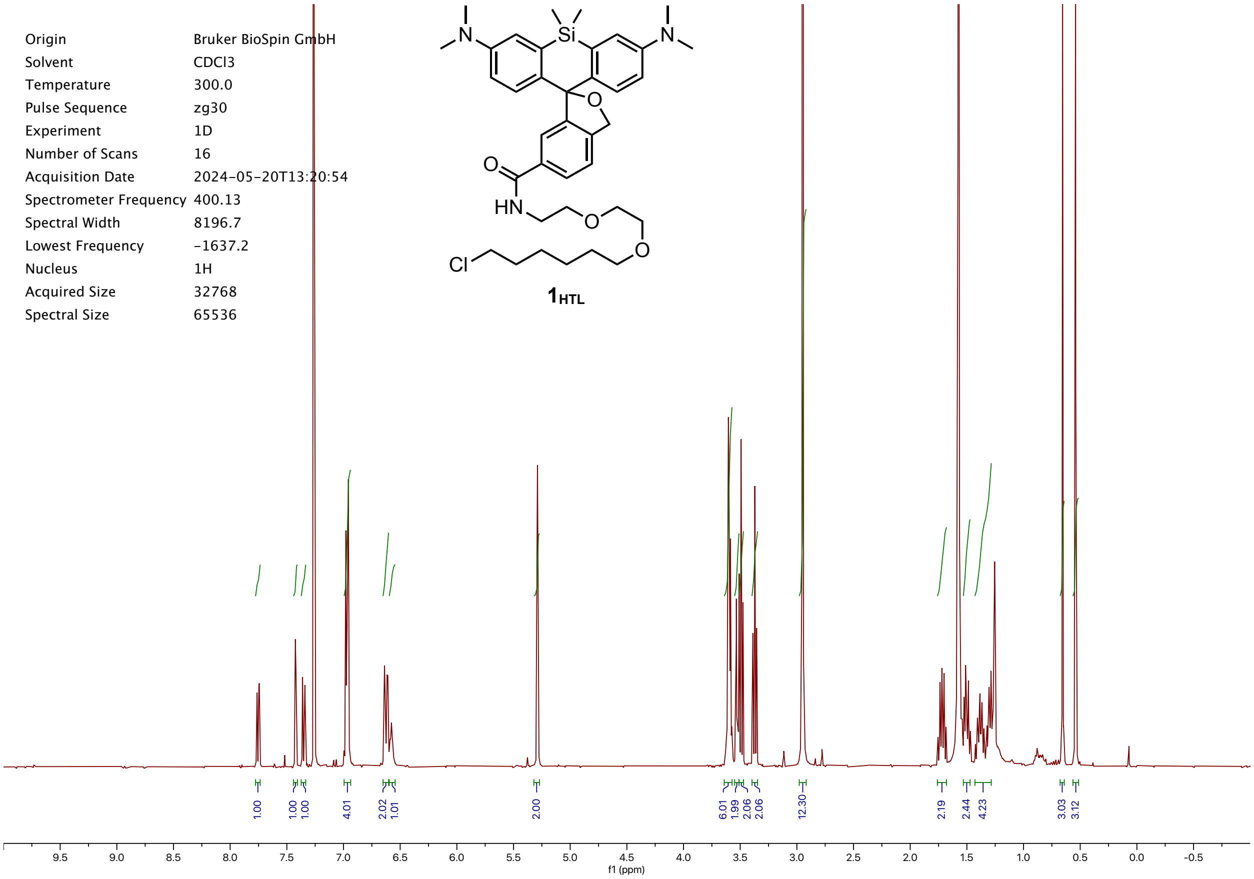
Origin Bruker BioSpin GmbH
Solvent CDCl3
Temperature 300.0
Pulse Sequence zg30
Experiment 1D
Number of Scans 16
Acquisition Date 2024-05-20T13:20:54
Spectrometer Frequency 400.13
Spectral Width 8196.7
Lowest Frequency -1637.2
Nucleus 1H
Acquired Size 32768
Spectral Size 65536
1.00 1.00 1.00 4.01 2.02 1.01 2.00 6.01 1.99 2.06 2.06 12.30 2.19 2.44 4.23 3.03 3.12
f1 (ppm)

$\mathbf{1_{HTL}}$

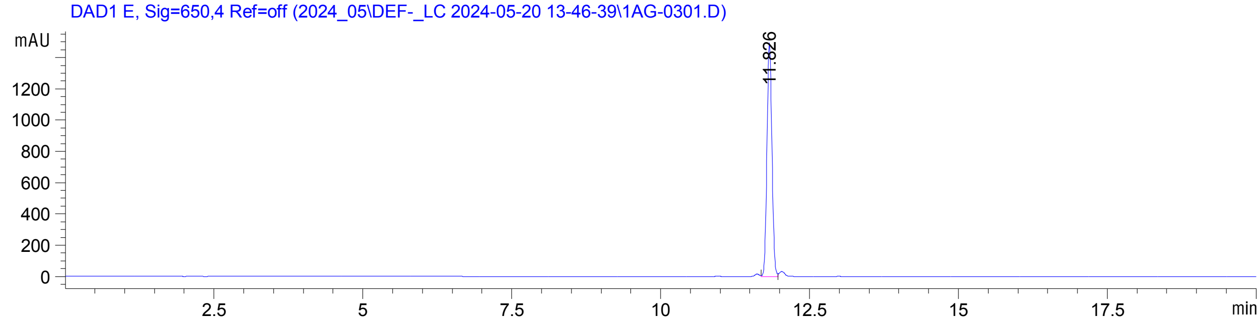
DAD1 E, Sig=650,4 Ref=off (2024_05\DEF-_LC 2024-05-20 13-46-39\1AG-0301.D)
mAU
11.826
min

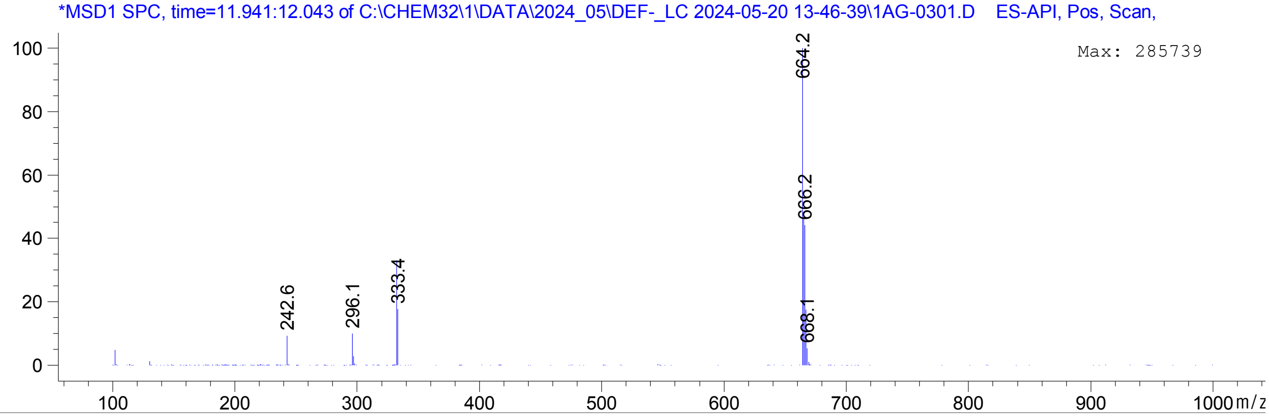
*MSD1 SPC, time=11.941:12.043 of C:\CHEM32\1\DATA\2024_05\DEF-_LC 2024-05-20 13-46-39\1AG-0301.D ES-API, Pos, Scan,
Max: 285739
664.2
666.2
668.1
242.6
296.1
333.4
m/z

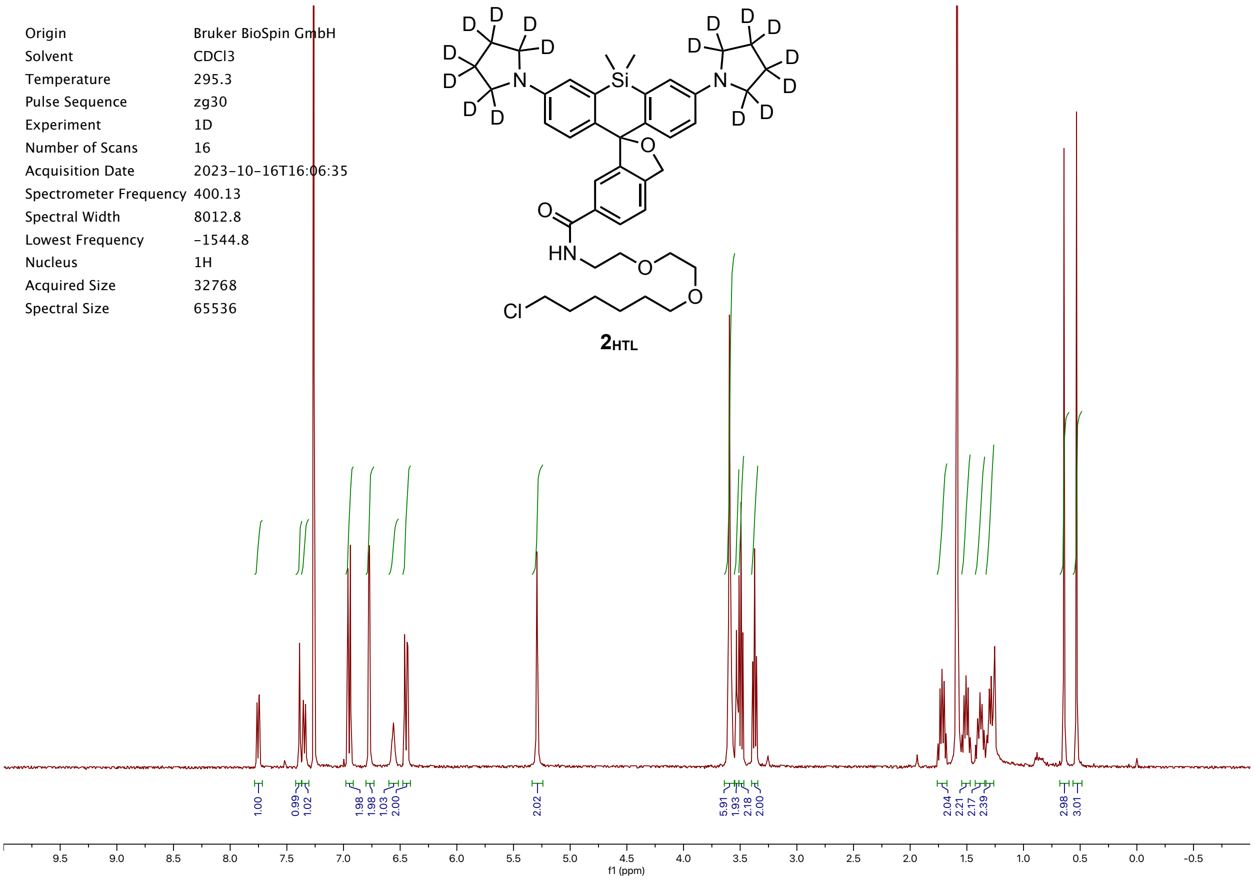
Origin Bruker BioSpin GmbH
Solvent CDCl3
Temperature 295.3
Pulse Sequence zg30
Experiment 1D
Number of Scans 16
Acquisition Date 2023-10-16T16:06:35
Spectrometer Frequency 400.13
Spectral Width 8012.8
Lowest Frequency -1544.8
Nucleus 1H
Acquired Size 32768
Spectral Size 65536
1.00 0.99 1.02 1.98 1.98 1.03 2.00 2.02 5.91 1.93 2.18 2.00 2.04 2.21 2.17 2.39 2.98 3.01
f1 (ppm)

2HTL

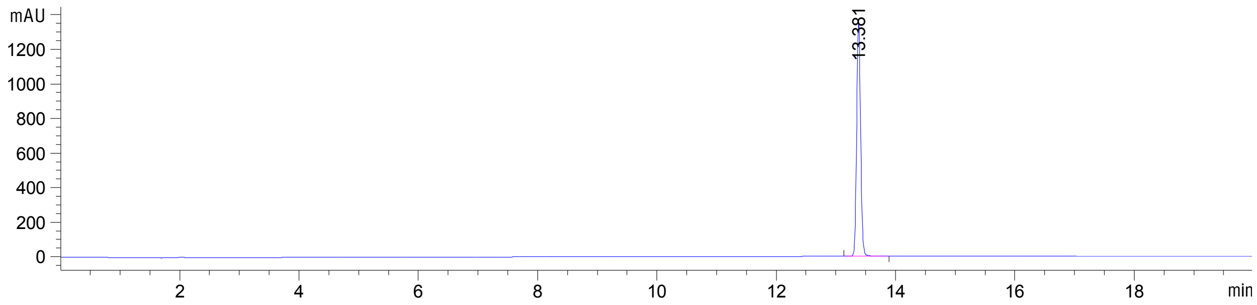
DAD1 E, Sig=650,4 Ref=off (2023_09\DEF-_LC 2023-10-06 14-57-23\1AF-0101.D)
mAU
13.381
min

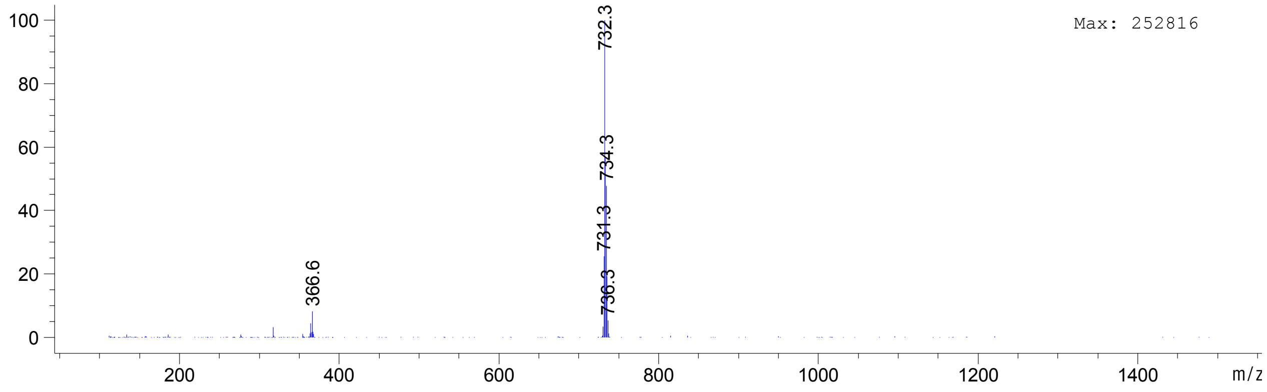
*MSD2 SPC, time=13.470:13.597 of C:\CHEM32\1\DATA\2023_09\DEF-_LC 2023-10-06 14-57-23\1AF-0101.D ES-API, Pos, Scan,
Max: 252816
732.3
734.3
731.3
736.3
366.6
m/z

Origin	Bruker BioSpin GmbH
Solvent	CDCl3
Temperature	295.3
Pulse Sequence	zg30
Experiment	1D
Number of Scans	16
Acquisition Date	2023-10-16T09:47:30
Spectrometer Frequency	400.13
Spectral Width	8012.8
Lowest Frequency	-1544.6
Nucleus	1H
Acquired Size	32768
Spectral Size	65536

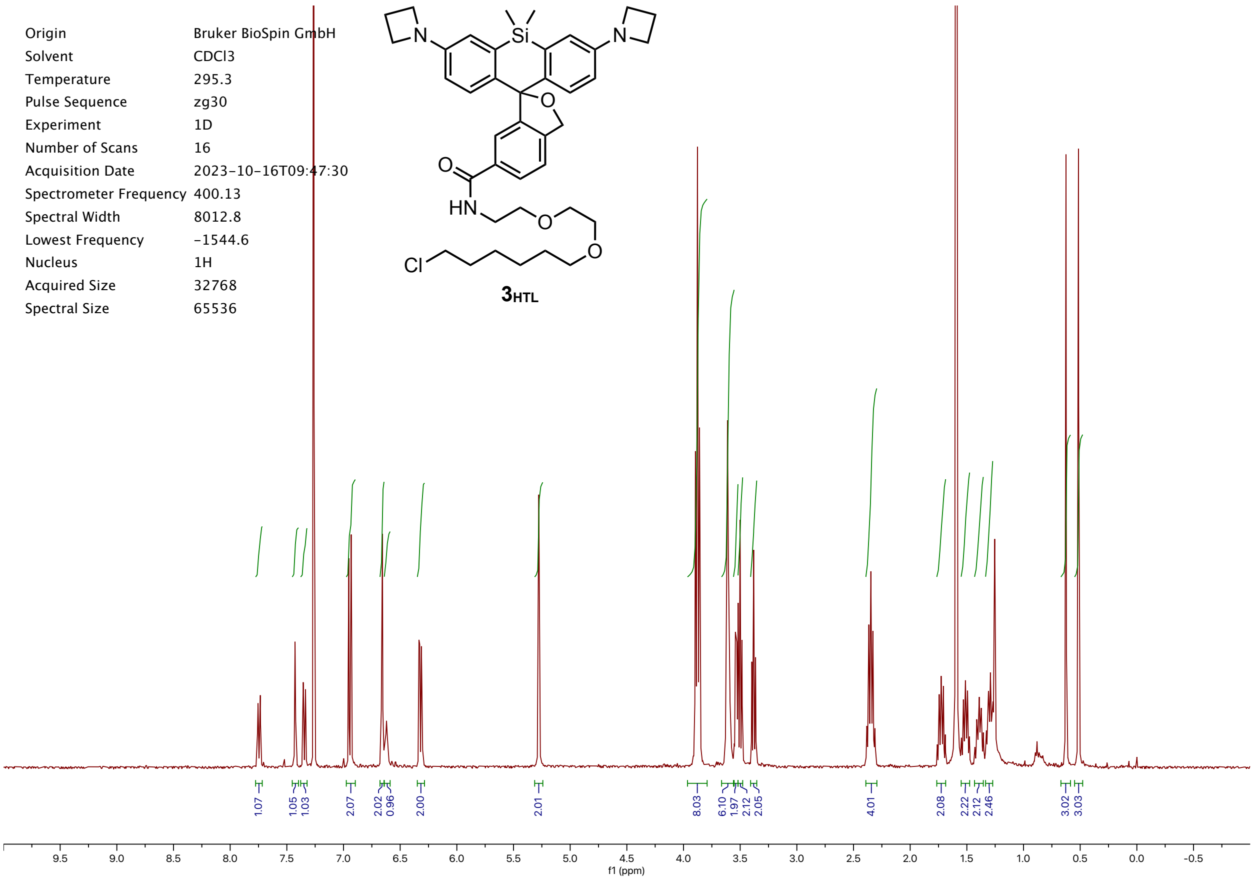

DAD1 E, Sig=650,4 Ref=off (2023_10\DEF-_LC 2023-10-16 10-59-39\1AE-0101.D)

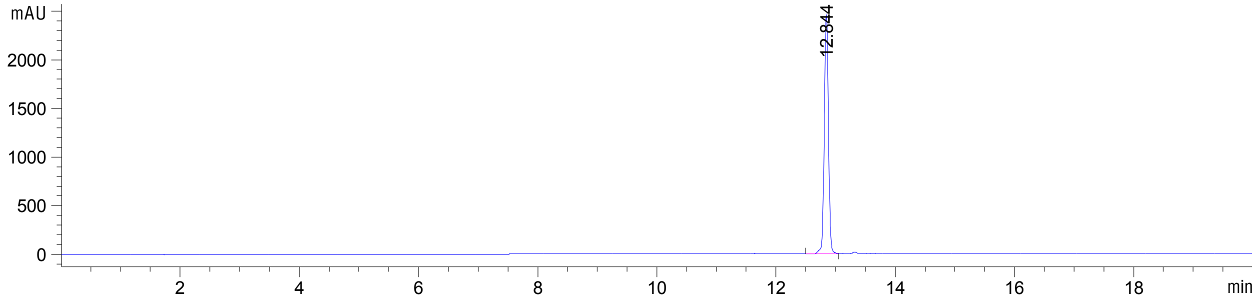

*MSD2 SPC, time=12.945:13.054 of C:\CHEM32\1\DATA\2023_10\DEF-_LC 2023-10-16 10-59-39\1AE-0101.D ES-API, Pos, Scan,

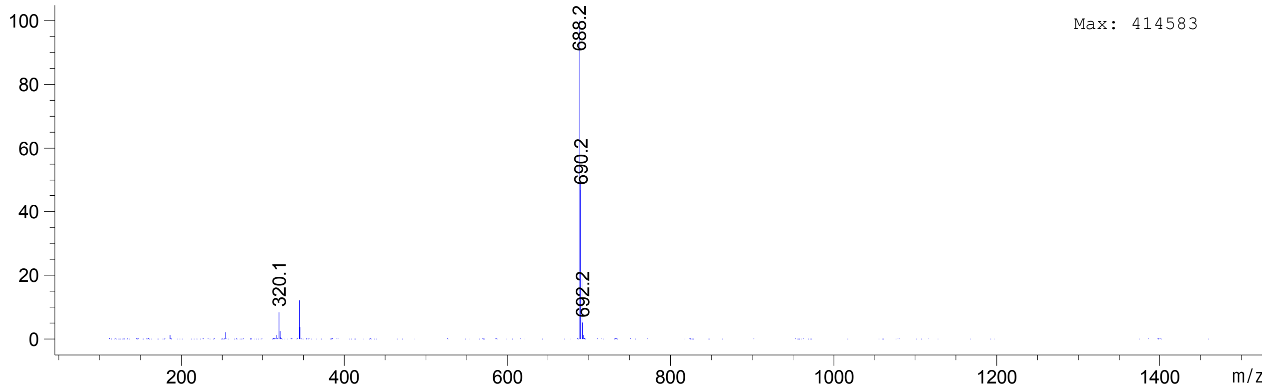

Origin	Bruker BioSpin GmbH
Solvent	CDCl3
Temperature	295.6
Pulse Sequence	zg30
Experiment	1D
Number of Scans	16
Acquisition Date	2023-10-23T14:25:11
Spectrometer Frequency	400.13
Spectral Width	8012.8
Lowest Frequency	-1597.4
Nucleus	1H
Acquired Size	32768
Spectral Size	65536

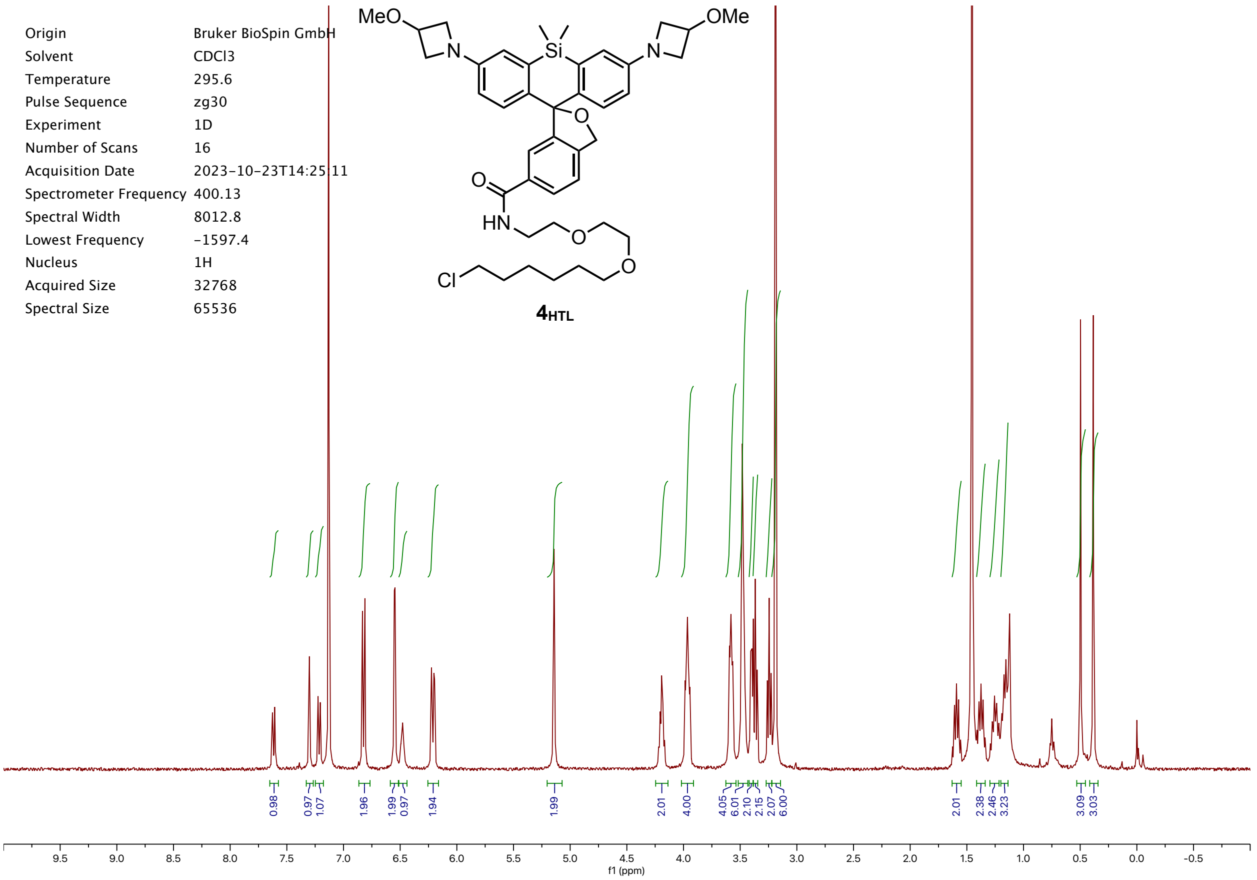

4HTL

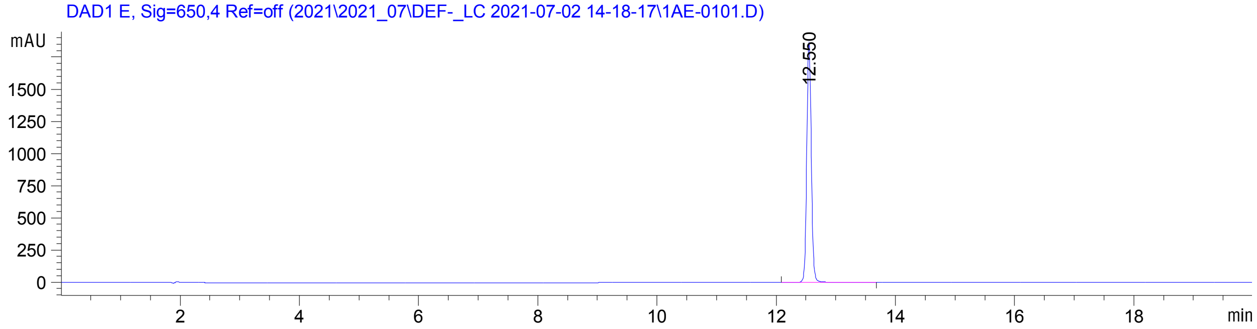

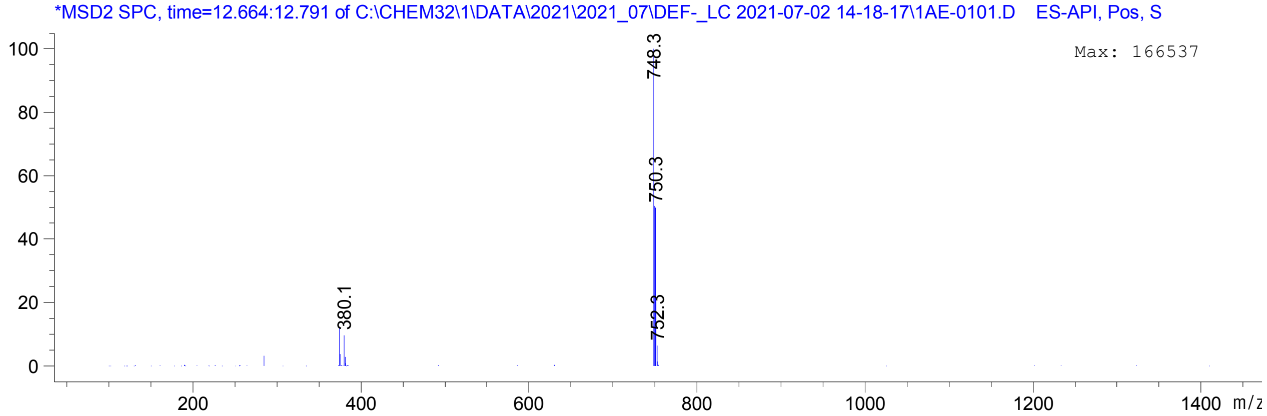

Origin	Bruker BioSpin GmbH
Solvent	CDCl3
Temperature	295.2
Pulse Sequence	zg30
Experiment	1D
Number of Scans	16
Acquisition Date	2023-10-16T16:01:52
Spectrometer Frequency	400.13
Spectral Width	8012.8
Lowest Frequency	-1572.5
Nucleus	1H
Acquired Size	32768
Spectral Size	65536

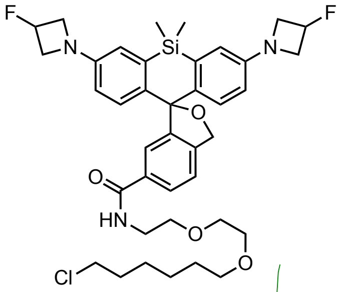

$\mathbf{5}_{\mathbf{HTL}}$

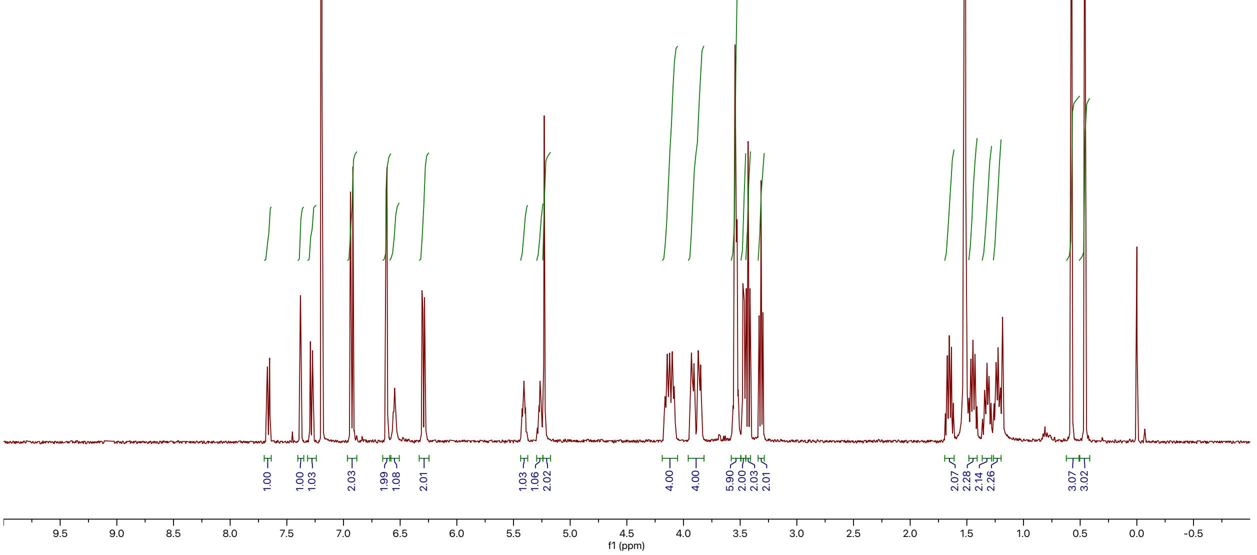

DAD1 E, Sig=650,4 Ref=off (2023_09\DEF-_LC 2023-10-05 15-22-22\1AE-0101.D)

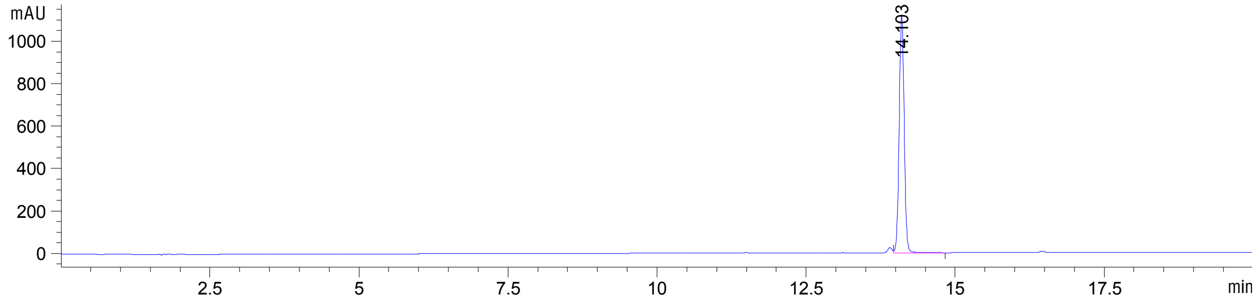

*MSD2 SPC, time=14.194:14.321 of C:\CHEM32\1\DATA\2023_09\DEF-_LC 2023-10-05 15-22-22\1AE-0101.D ES-API, Pos, Scan,

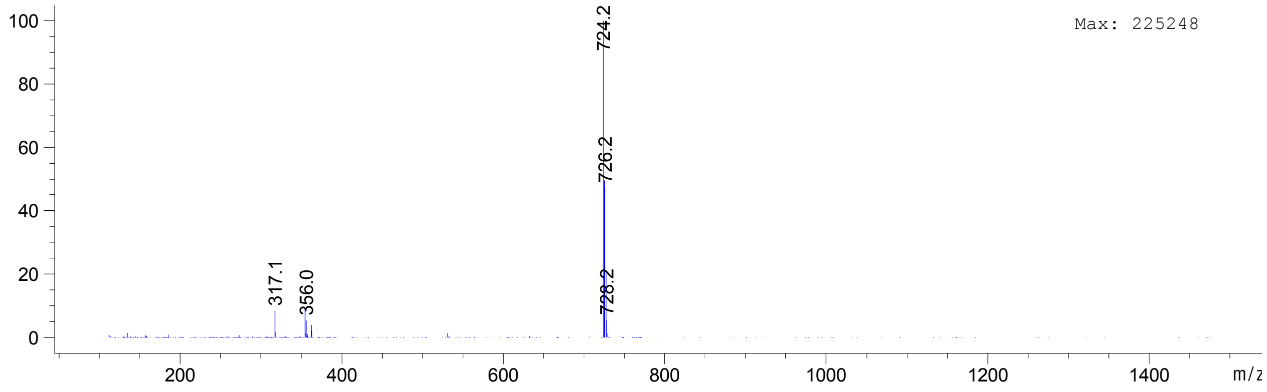

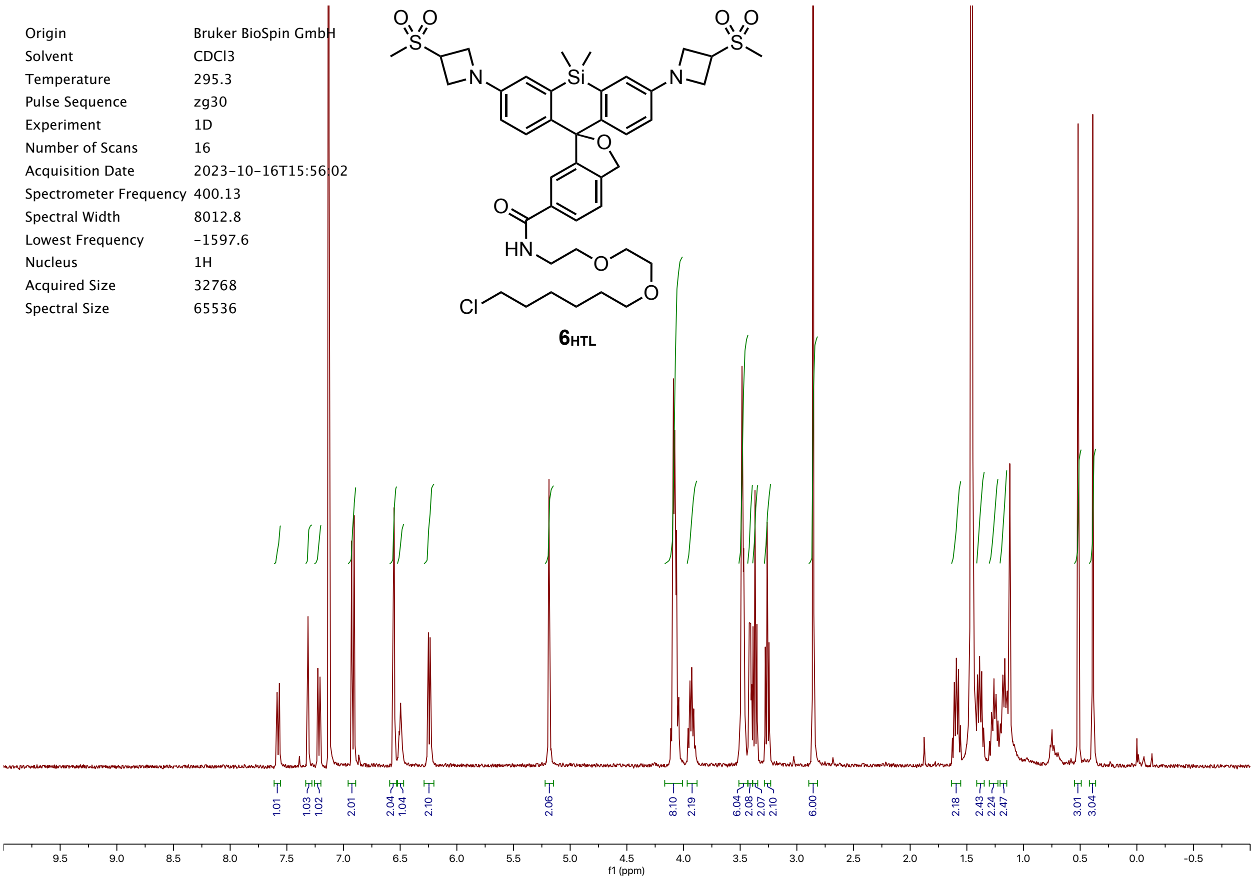

Origin Bruker BioSpin GmbH
Solvent CDCl3
Temperature 295.3
Pulse Sequence zg30
Experiment 1D
Number of Scans 16
Acquisition Date 2023-10-16T15:56:02
Spectrometer Frequency 400.13
Spectral Width 8012.8
Lowest Frequency -1597.6
Nucleus 1H
Acquired Size 32768
Spectral Size 65536
6HTL
1.01 1.03 1.02 2.01 2.04 1.04 2.10 2.06 8.10 2.19 6.04 2.08 2.07 2.10 6.00 2.18 2.43 2.24 2.47 3.01 3.04
f1 (ppm)

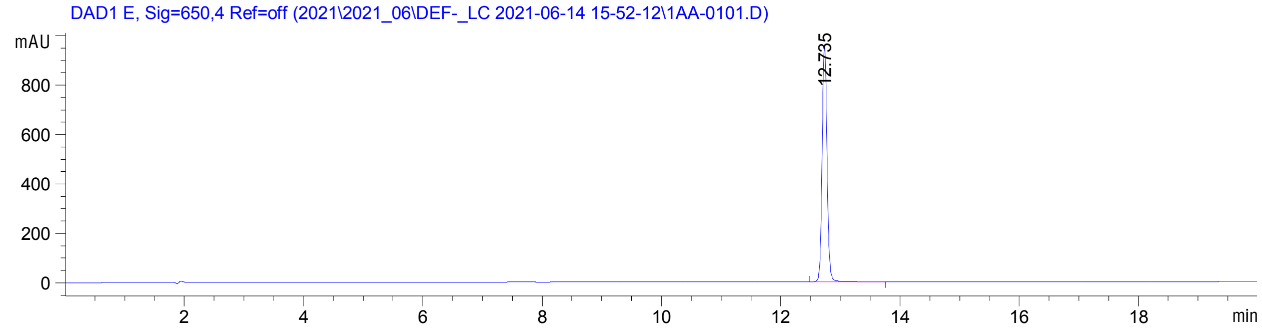

DAD1 E, Sig=650,4 Ref=off (2021\2021_06\DEF-_LC 2021-06-14 15-52-12\1AA-0101.D)
mAU
12.735
min

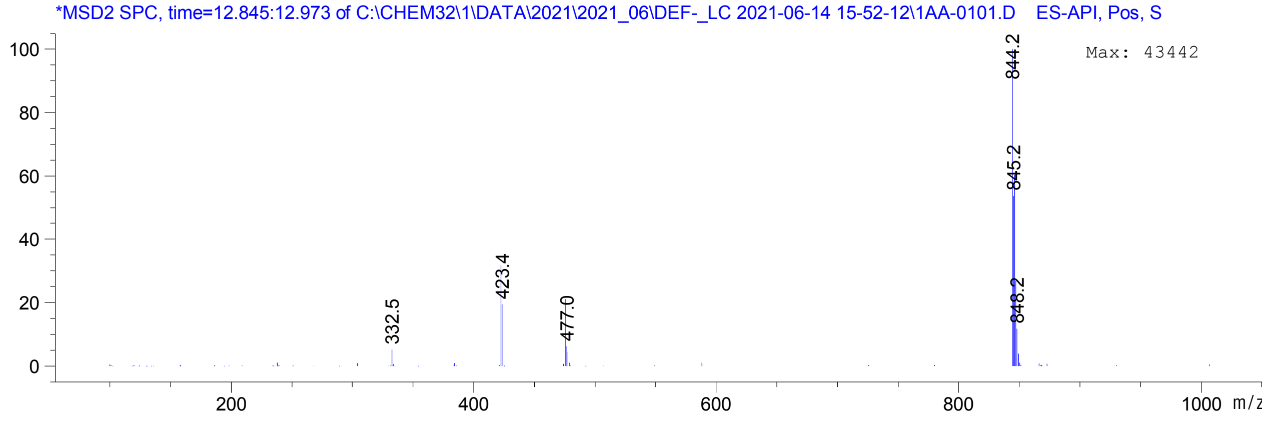

*MSD2 SPC, time=12.845:12.973 of C:\CHEM32\1\DATA\2021\2021_06\DEF-_LC 2021-06-14 15-52-12\1AA-0101.D ES-API, Pos, S
Max: 43442
844.2
845.2
848.2
423.4
477.0
332.5
m/z

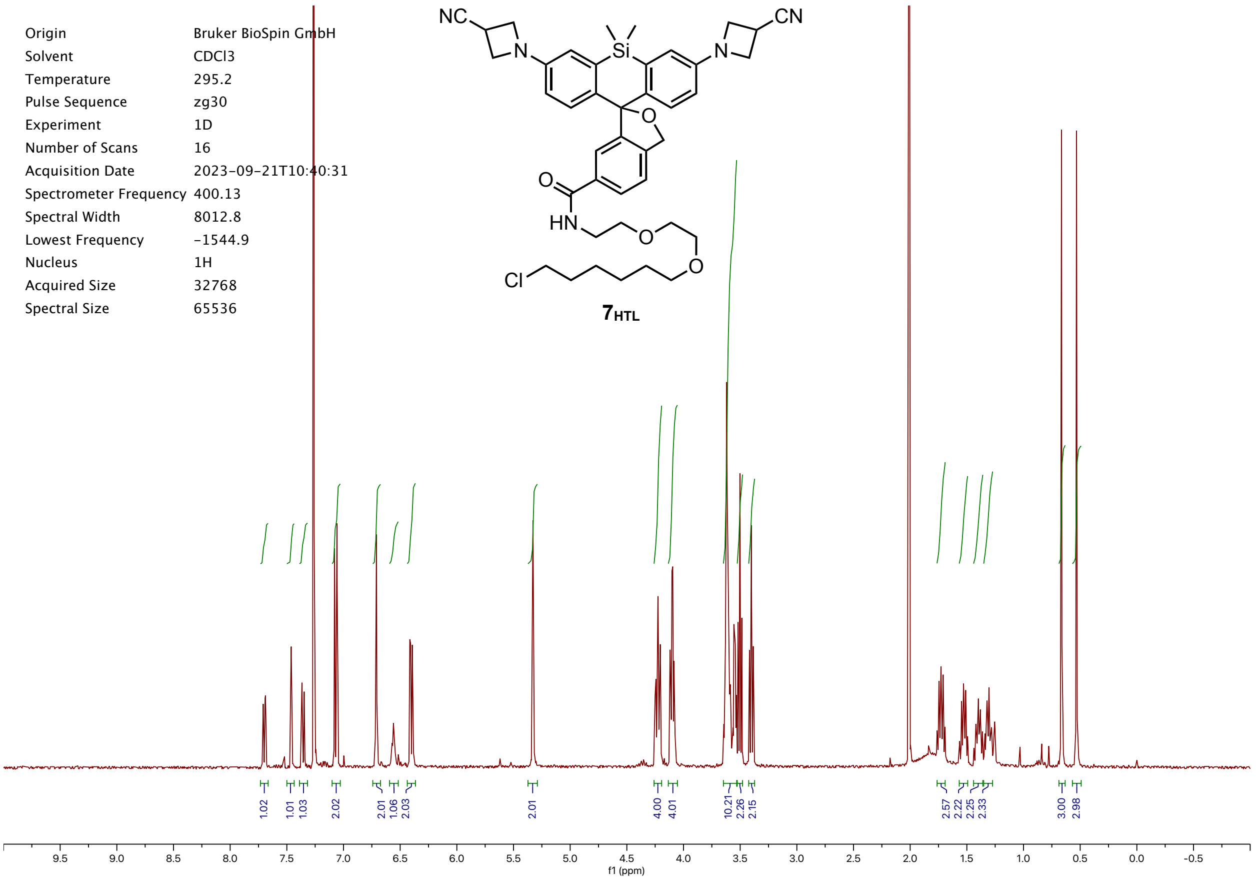

Origin Bruker BioSpin GmbH
Solvent CDCl3
Temperature 295.2
Pulse Sequence zg30
Experiment 1D
Number of Scans 16
Acquisition Date 2023-09-21T10:40:31
Spectrometer Frequency 400.13
Spectral Width 8012.8
Lowest Frequency -1544.9
Nucleus 1H
Acquired Size 32768
Spectral Size 65536
NC
CN
N
N
Si
O
O
HN
O
O
Cl
7HTL
1.02
1.01
1.03
2.02
2.01
1.06
2.03
2.01
4.00
4.01
10.21
2.26
2.15
2.57
2.22
2.25
2.33
3.00
2.98
f1 (ppm)

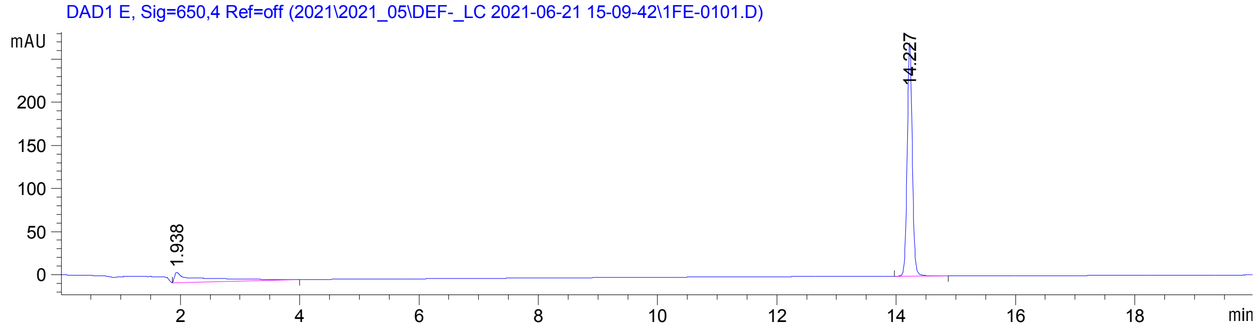

DAD1 E, Sig=650,4 Ref=off (2021\2021_05\DEF-_LC 2021-06-21 15-09-42\1FE-0101.D)
mAU
14.227
1.938
min

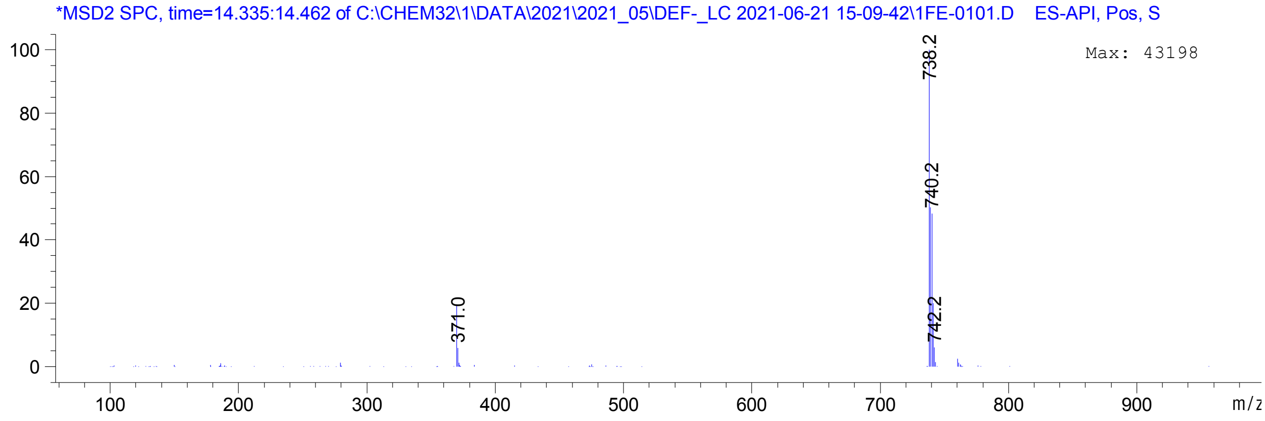

*MSD2 SPC, time=14.335:14.462 of C:\CHEM32\1\DATA\2021\2021_05\DEF-_LC 2021-06-21 15-09-42\1FE-0101.D ES-API, Pos, S
Max: 43198
738.2
740.2
742.2
371.0
m/z

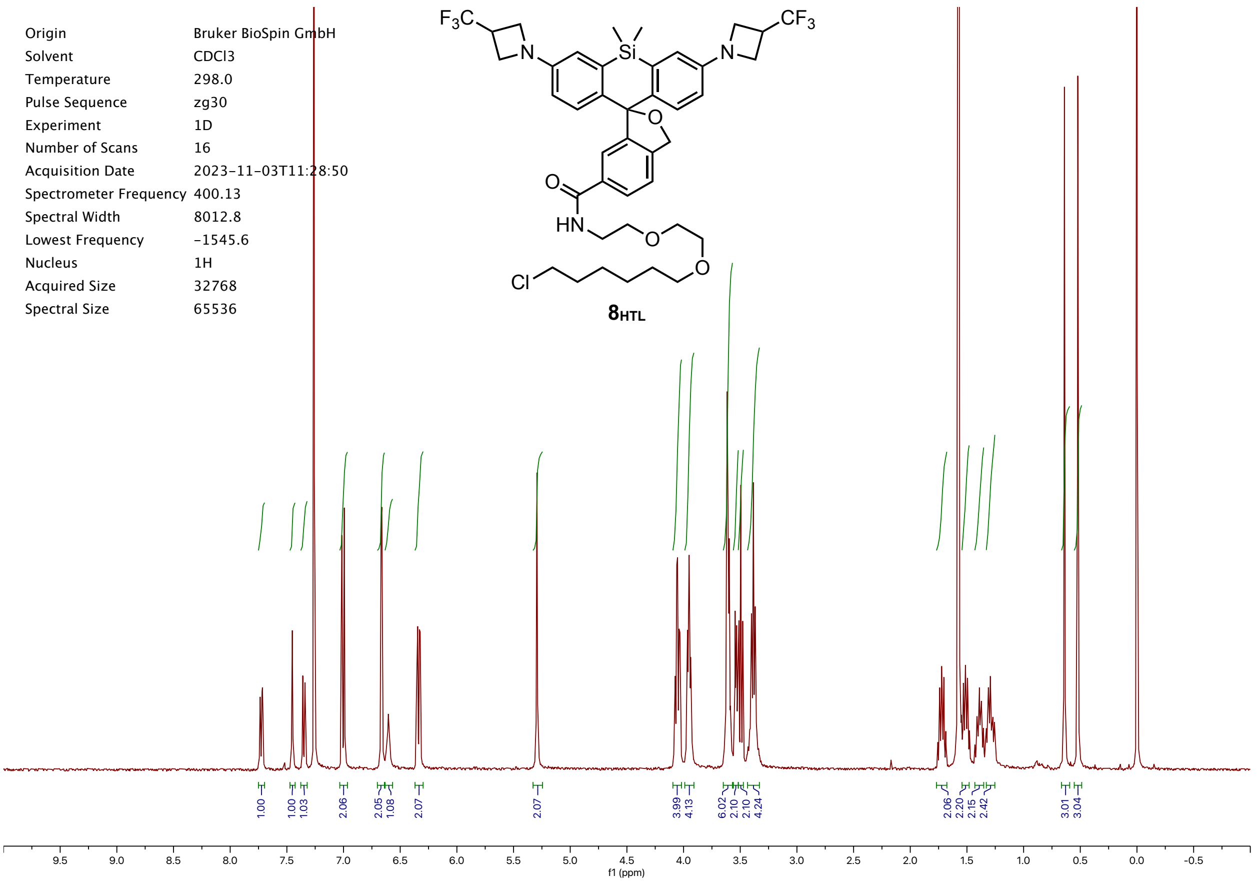

Origin Bruker BioSpin GmbH
Solvent CDCl3
Temperature 298.0
Pulse Sequence zg30
Experiment 1D
Number of Scans 16
Acquisition Date 2023-11-03T11:28:50
Spectrometer Frequency 400.13
Spectral Width 8012.8
Lowest Frequency -1545.6
Nucleus 1H
Acquired Size 32768
Spectral Size 65536
f1 (ppm)

8HTL

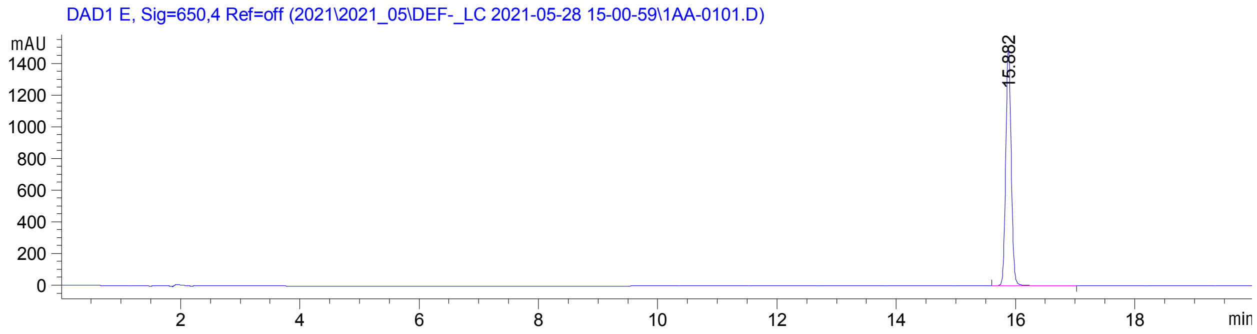

DAD1 E, Sig=650,4 Ref=off (2021\2021_05\DEF-_LC 2021-05-28 15-00-59\1AA-0101.D)
mAU
15.882
min

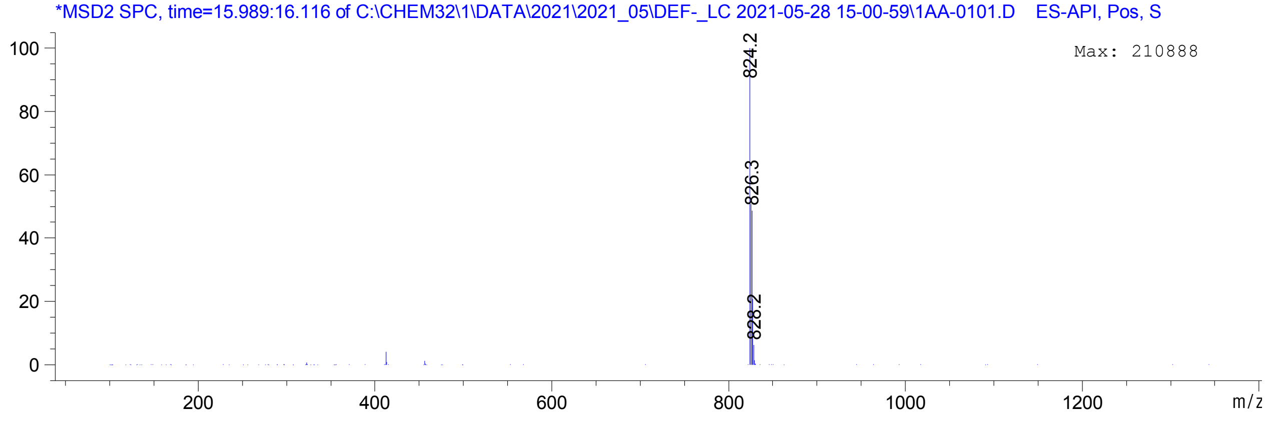

*MSD2 SPC, time=15.989:16.116 of C:\CHEM32\1\DATA\2021\2021_05\DEF-_LC 2021-05-28 15-00-59\1AA-0101.D ES-API, Pos, S
Max: 210888
824.2
826.3
828.2
m/z

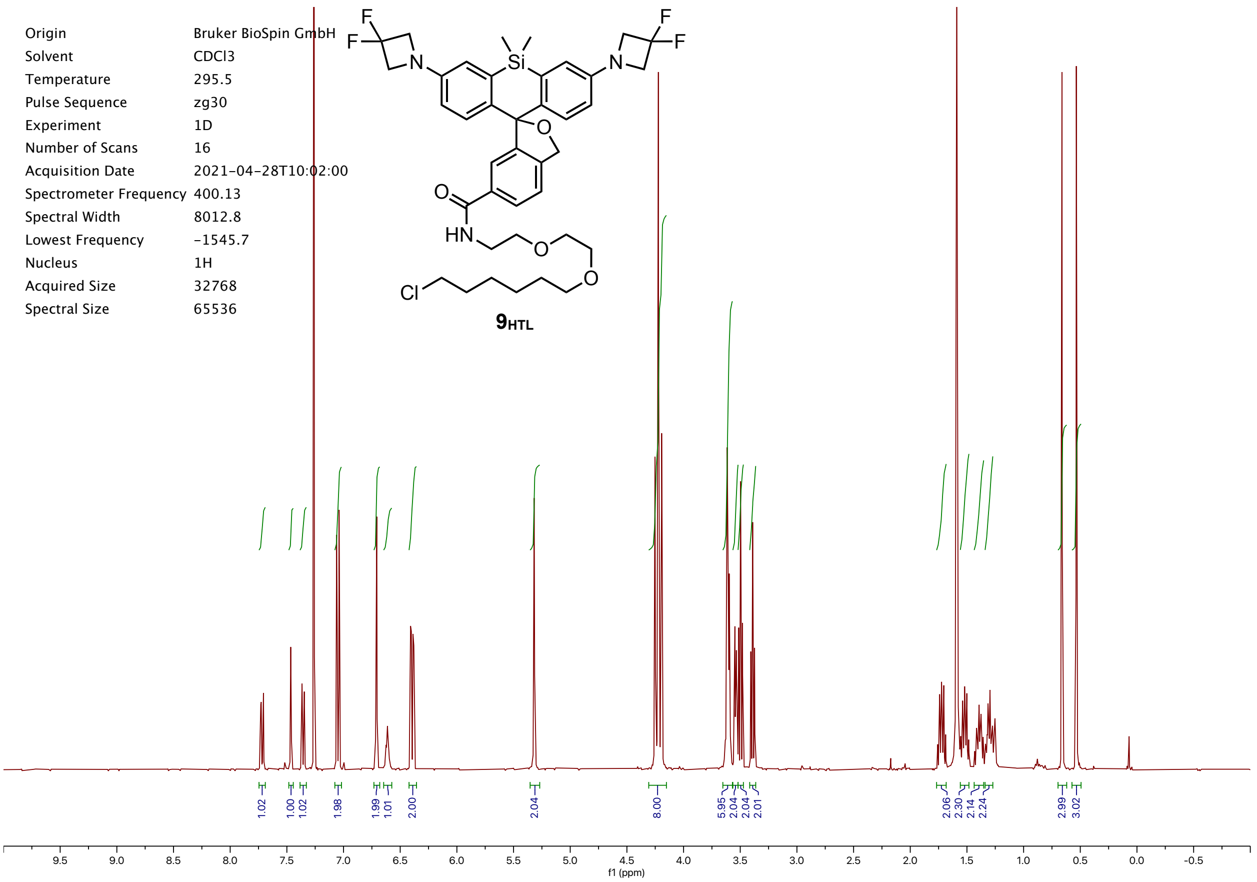
Origin Bruker BioSpin GmbH
Solvent CDCl3
Temperature 295.5
Pulse Sequence zg30
Experiment 1D
Number of Scans 16
Acquisition Date 2021-04-28T10:02:00
Spectrometer Frequency 400.13
Spectral Width 8012.8
Lowest Frequency -1545.7
Nucleus 1H
Acquired Size 32768
Spectral Size 65536
9HTL
1.02
1.00
1.02
1.98
1.99
1.01
2.00
2.04
8.00
5.95
2.04
2.04
2.01
2.06
2.30
2.14
2.24
2.99
3.02
f1 (ppm)

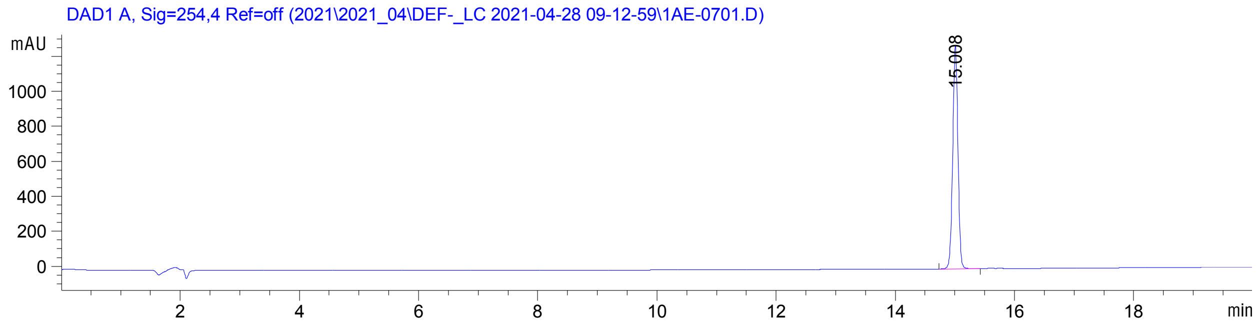
DAD1 A, Sig=254,4 Ref=off (2021\2021_04\DEF-_LC 2021-04-28 09-12-59\1AE-0701.D)
mAU
15.008
min

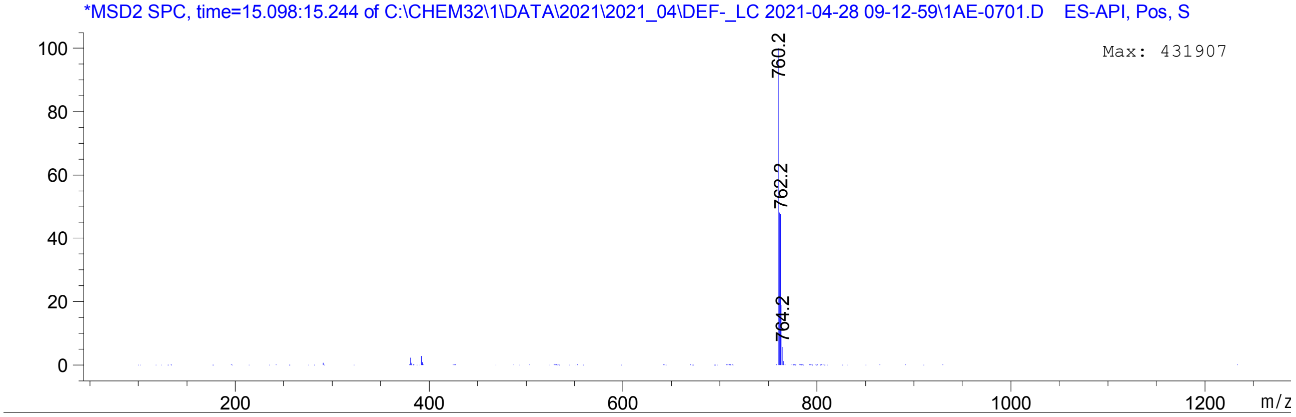
*MSD2 SPC, time=15.098:15.244 of C:\CHEM32\1\DATA\2021\2021_04\DEF-_LC 2021-04-28 09-12-59\1AE-0701.D ES-API, Pos, S
Max: 431907
760.2
762.2
764.2
m/z

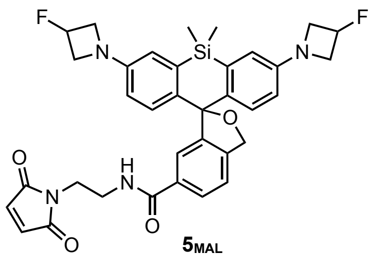

5MAL

Origin	Bruker BioSpin GmbH
Solvent	CDCl3
Temperature	298.0
Pulse Sequence	zg30
Experiment	1D
Number of Scans	16
Acquisition Date	2023-12-14T11:22:04
Spectrometer Frequency	400.13
Spectral Width	8196.7
Lowest Frequency	-1637.3
Nucleus	1H
Acquired Size	32768
Spectral Size	65536

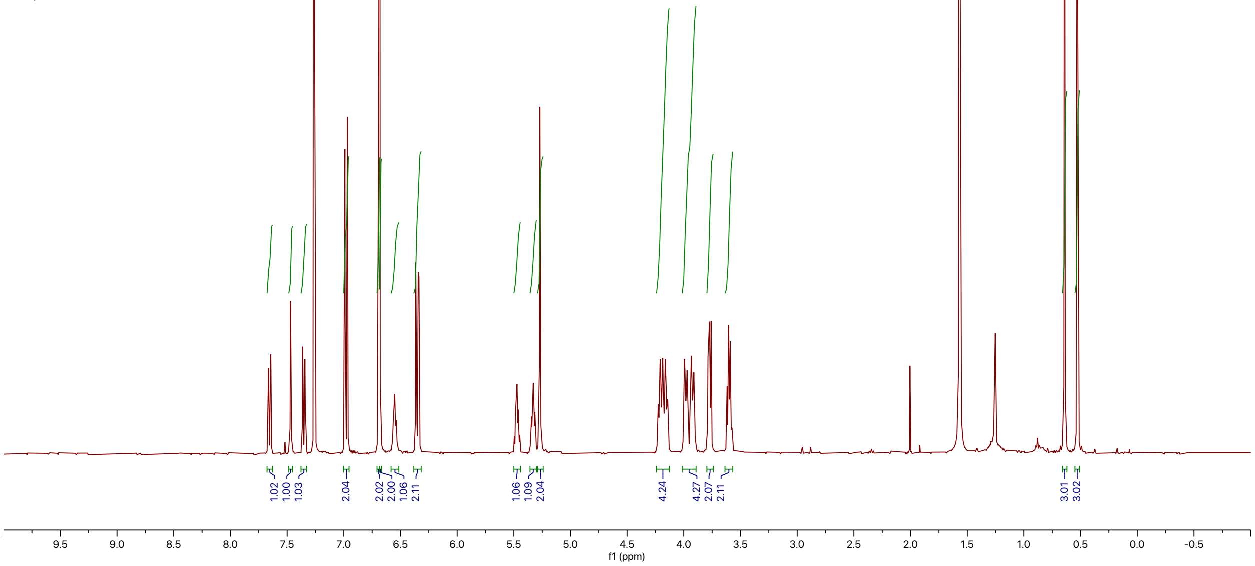

Origin	Bruker BioSpin GmbH
Solvent	CDCl3
Temperature	298.0
Pulse Sequence	zgpg30
Experiment	1D
Number of Scans	2048
Acquisition Date	2023-12-14T20:02:38
Spectrometer Frequency	100.62
Spectral Width	23809.5
Lowest Frequency	-1832.3
Nucleus	13C
Acquired Size	32768
Spectral Size	65536

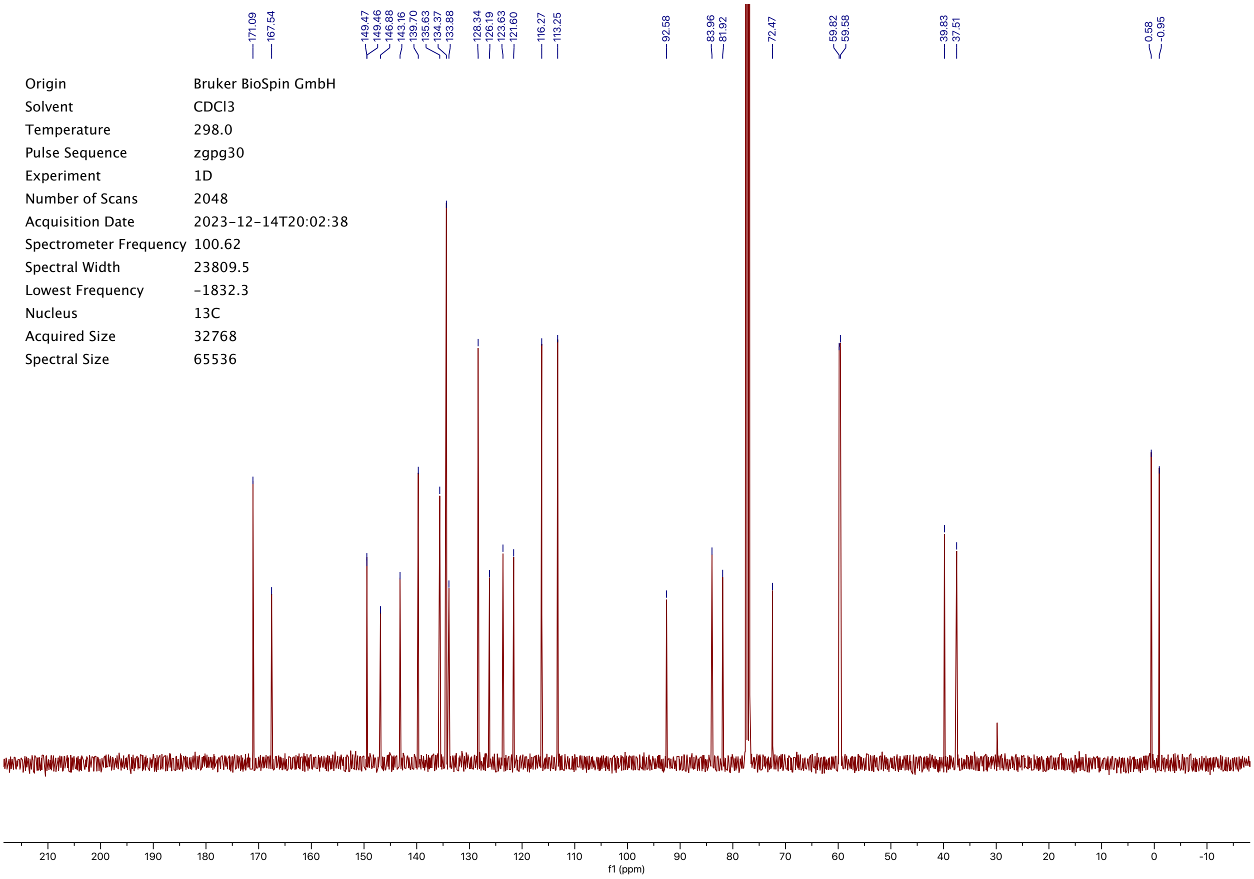

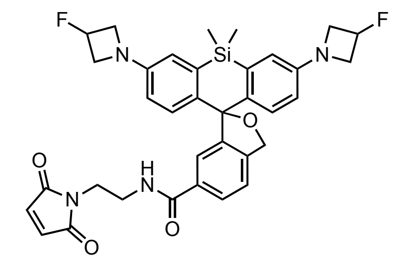

5$_{\mathbf{MAL}}$

DAD1 E, Sig=650,4 Ref=off (2023_11\DEF-_LC 2023-11-06 11-11-00\1AC-0501.D)

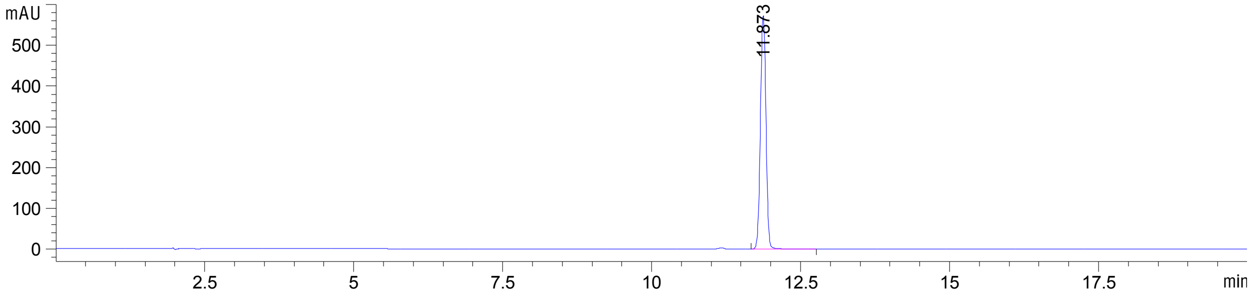

*MSD1 SPC, time=11.962:12.104 of C:\CHEM32\1\DATA\2023_11\DEF-_LC 2023-11-06 11-11-00\1AC-0501.D ES-API, Pos, Scan,

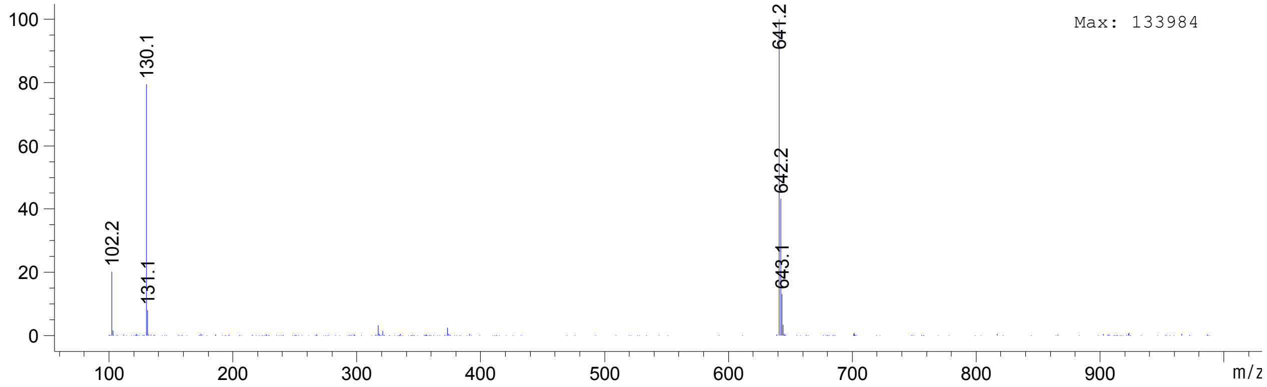

Origin	Bruker BioSpin GmbH
Solvent	CDCl3
Temperature	295.3
Pulse Sequence	zg30
Experiment	1D
Number of Scans	16
Acquisition Date	2023-10-18T10:06:43
Spectrometer Frequency	400.13
Spectral Width	8012.8
Lowest Frequency	-1618.7
Nucleus	1H
Acquired Size	32768
Spectral Size	65536

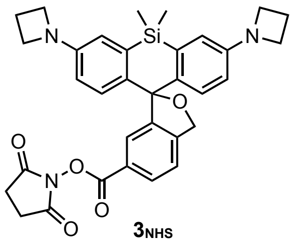

3_{NHS}

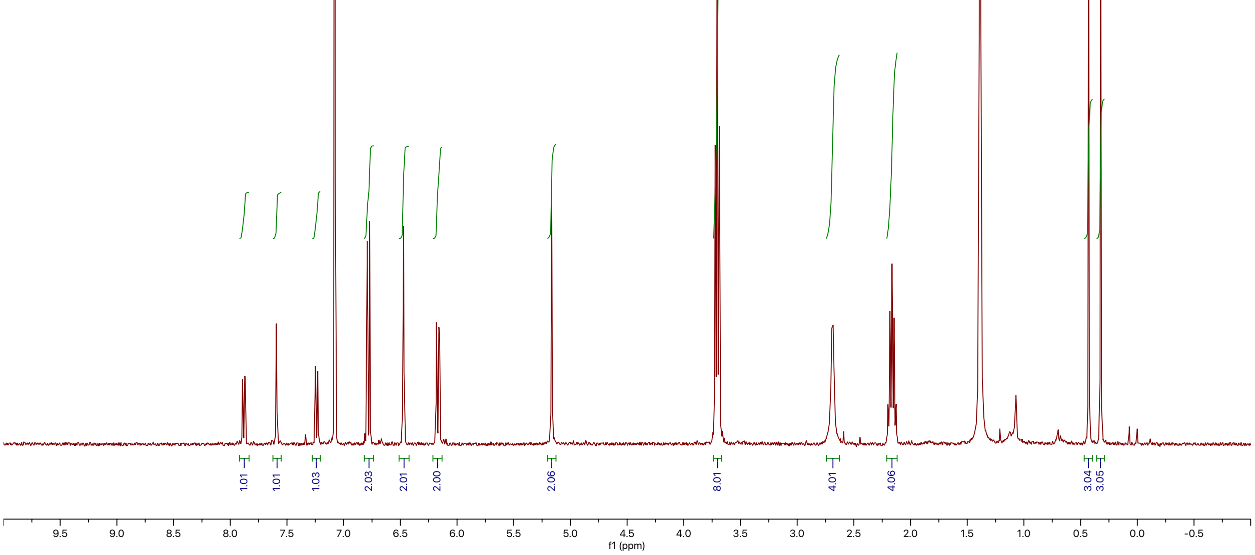

Origin	Bruker BioSpin GmbH
Solvent	CDCl3
Temperature	296.1
Pulse Sequence	zgpg30
Experiment	1D
Number of Scans	2048
Acquisition Date	2023-10-18T21:35:17
Spectrometer Frequency	100.62
Spectral Width	24038.5
Lowest Frequency	-1945.9
Nucleus	13C
Acquired Size	32768
Spectral Size	65536

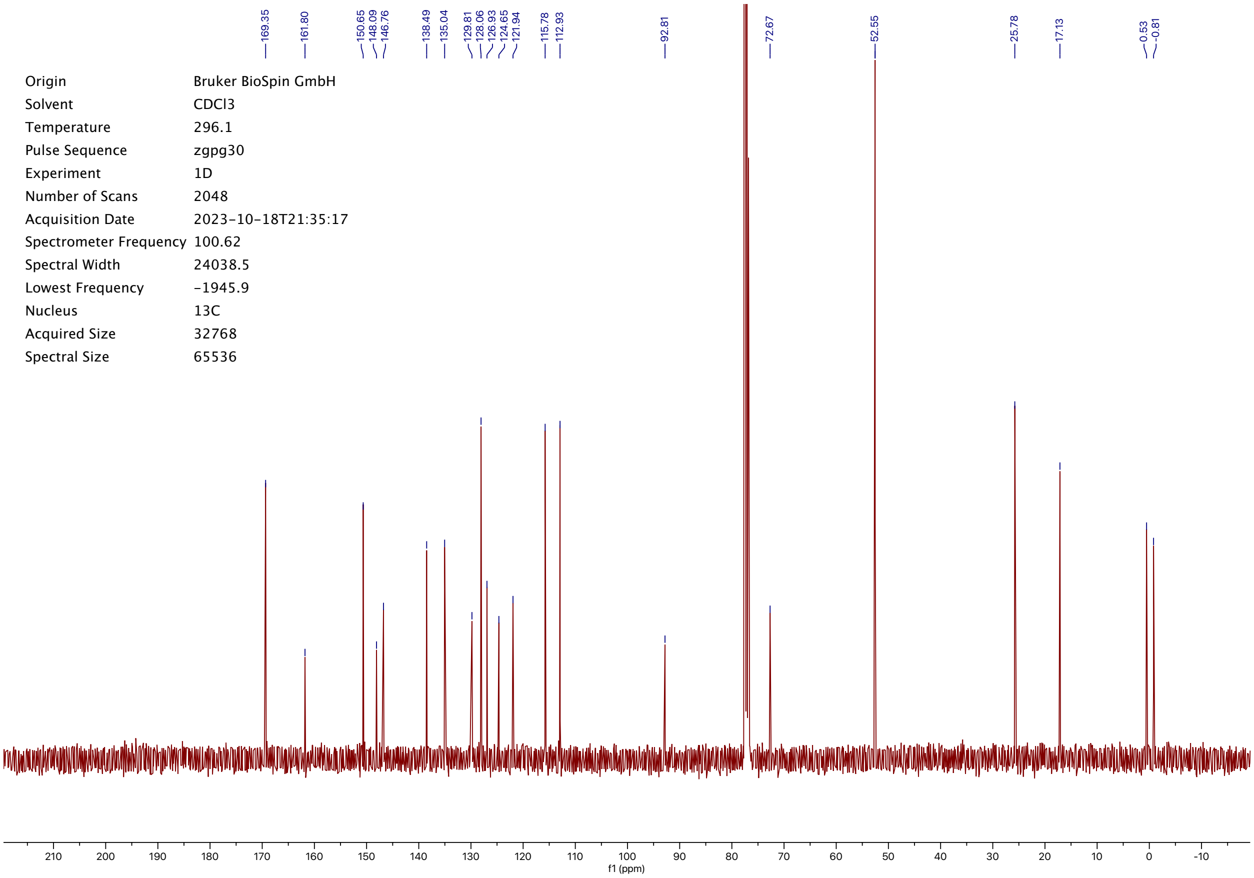

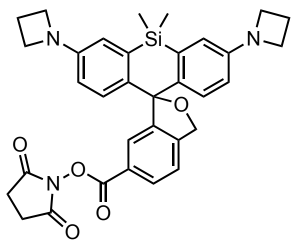

$\mathbf{3_{NHS}}$

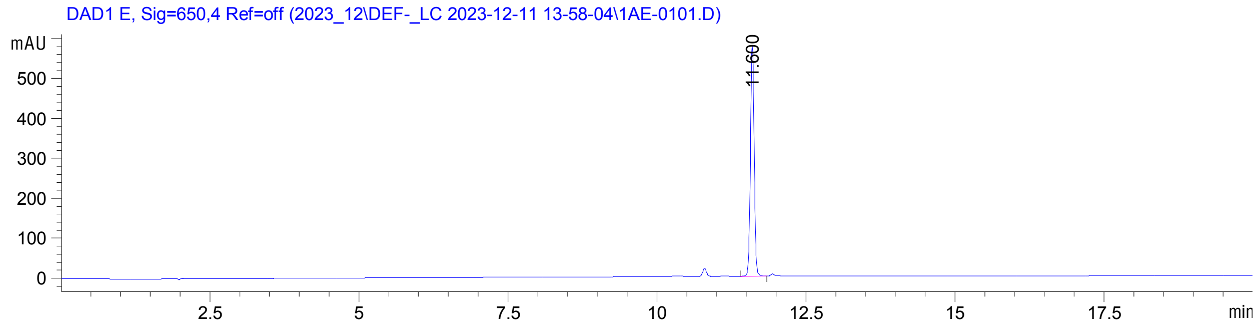
DAD1 E, Sig=650,4 Ref=off (2023_12\DEF-_LC 2023-12-11 13-58-04\1AE-0101.D)
mAU
500
400
300
200
100
0
11.600
2.5
5
7.5
10
12.5
15
17.5
min

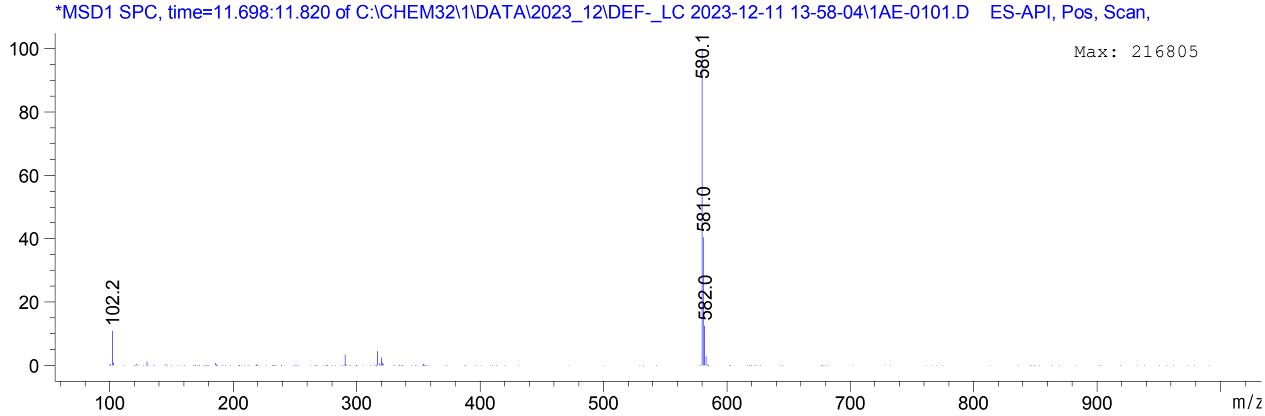
*MSD1 SPC, time=11.698:11.820 of C:\CHEM32\1\DATA\2023_12\DEF-_LC 2023-12-11 13-58-04\1AE-0101.D ES-API, Pos, Scan,
Max: 216805
100
80
60
40
20
0
580.1
581.0
582.0
102.2
100
200
300
400
500
600
700
800
900
m/z

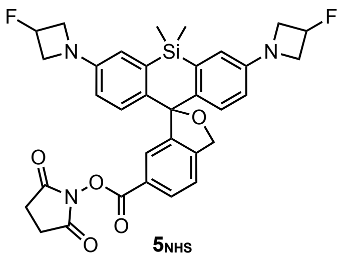

Origin	Bruker BioSpin GmbH
Solvent	CDCl3
Temperature	298.0
Pulse Sequence	zg30
Experiment	1D
Number of Scans	16
Acquisition Date	2023-11-08T12:18:24
Spectrometer Frequency	400.13
Spectral Width	8012.8
Lowest Frequency	-1535.6
Nucleus	1H
Acquired Size	32768
Spectral Size	65536

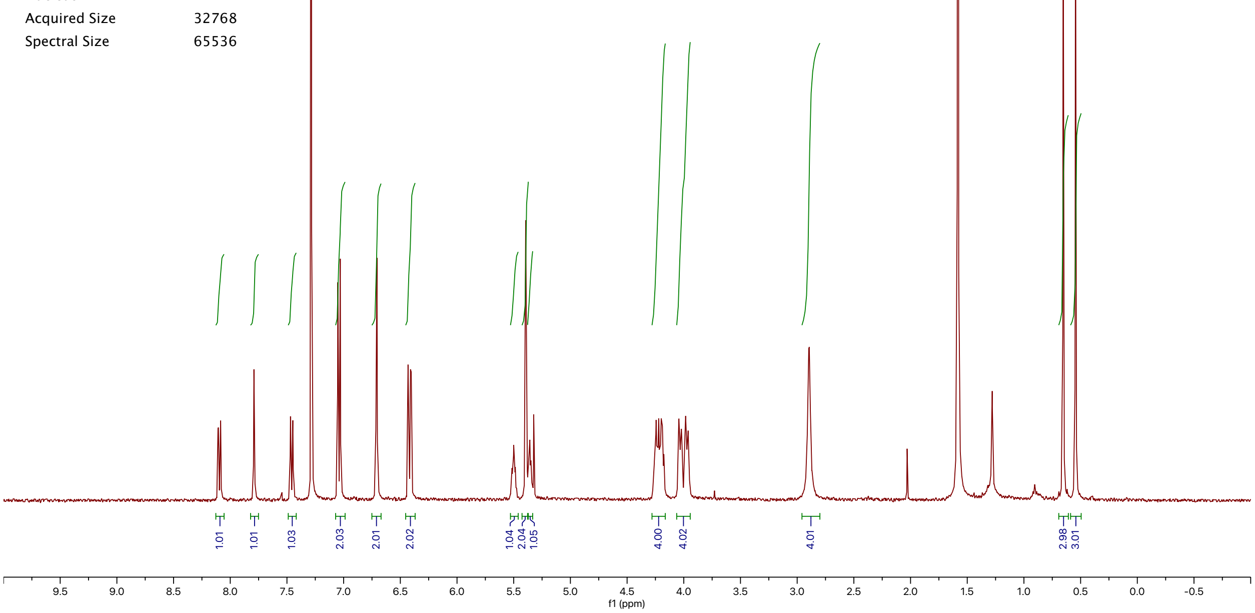

Origin	Bruker BioSpin GmbH
Solvent	CDCl3
Temperature	298.0
Pulse Sequence	zgpg30
Experiment	1D
Number of Scans	2048
Acquisition Date	2023-11-08T15:33:16
Spectrometer Frequency	100.62
Spectral Width	24038.5
Lowest Frequency	-1946.2
Nucleus	13C
Acquired Size	32768
Spectral Size	65536

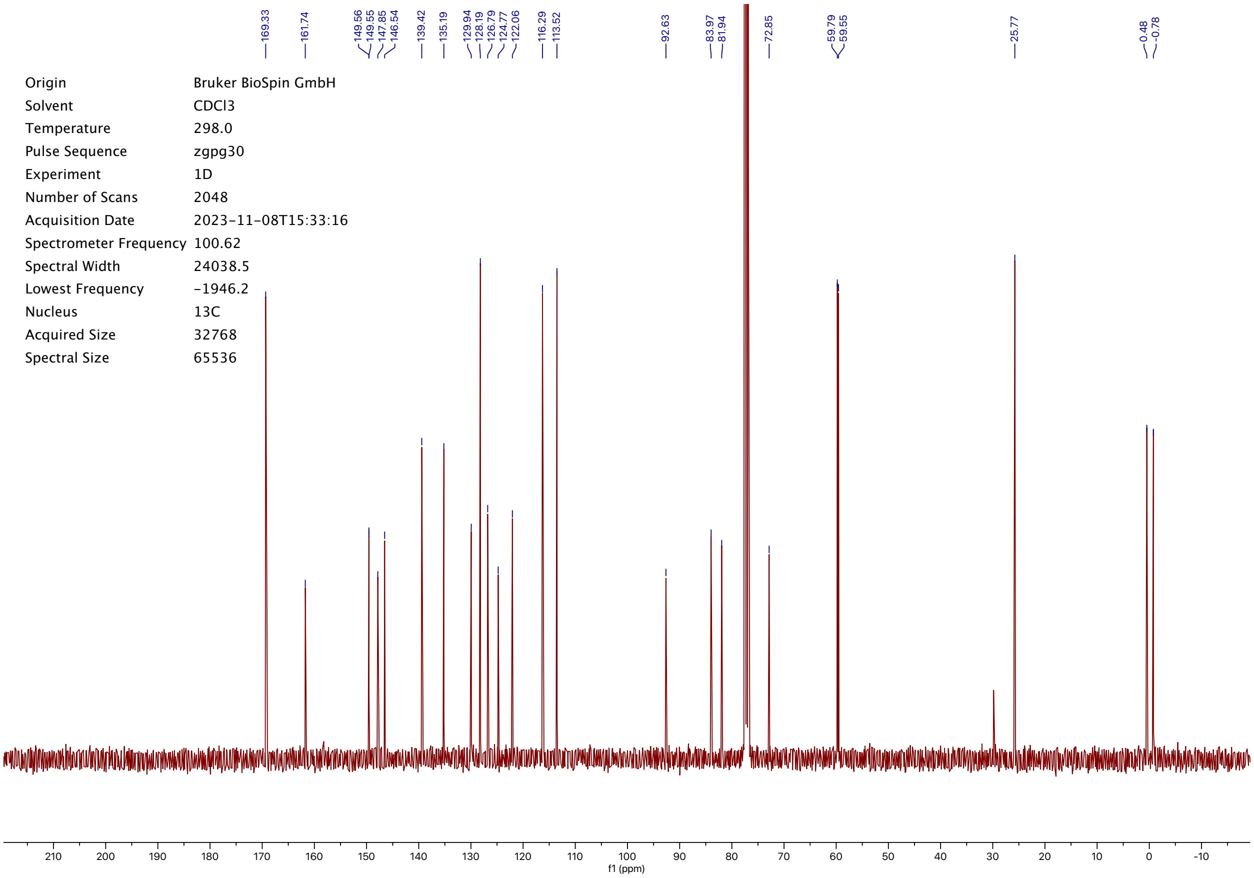

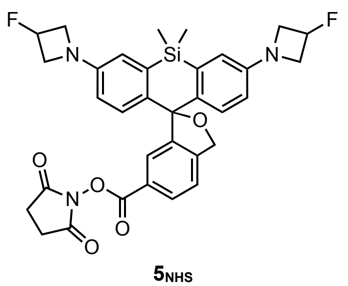
F
N
Si
N
F
O
O
N
O
O
O
O

5$_{NHS}$

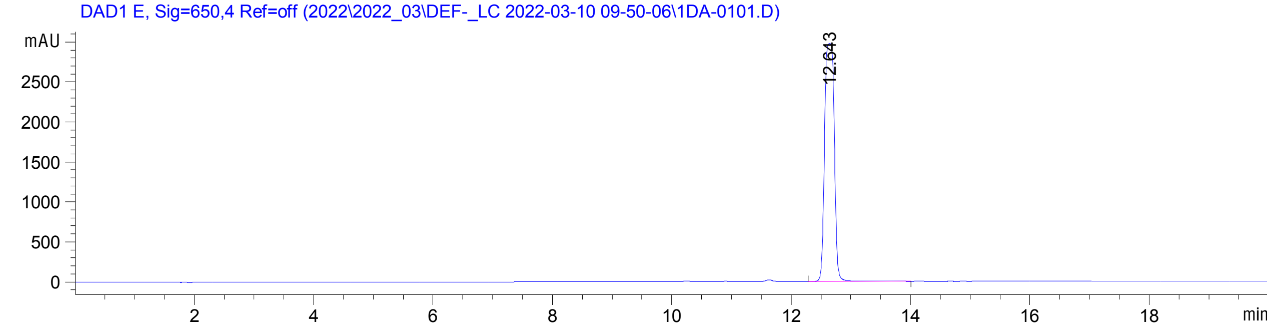
DAD1 E, Sig=650,4 Ref=off (2022\2022_03\DEF-_LC 2022-03-10 09-50-06\1DA-0101.D)
mAU
2500
2000
1500
1000
500
0
12.643
2
4
6
8
10
12
14
16
18
min

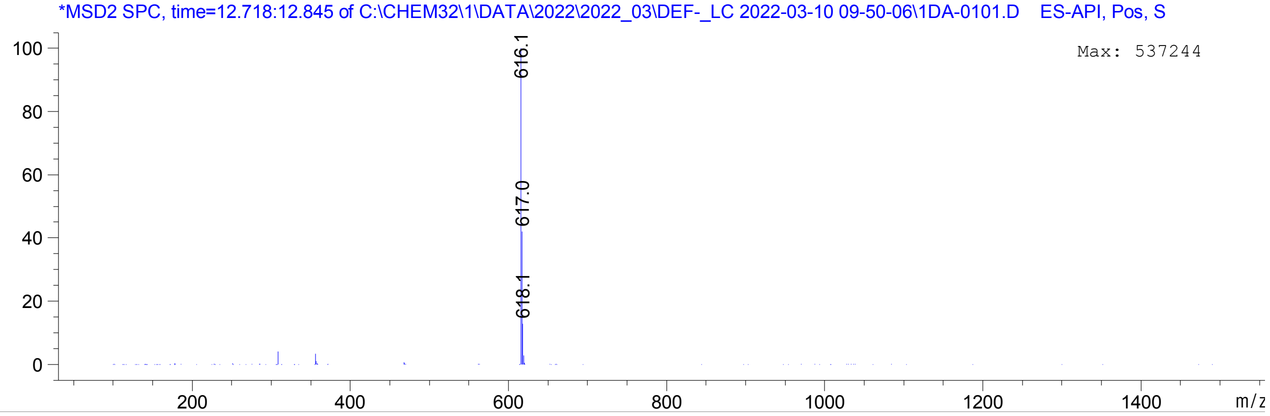
*MSD2 SPC, time=12.718:12.845 of C:\CHEM32\1\DATA\2022\2022_03\DEF-_LC 2022-03-10 09-50-06\1DA-0101.D ES-API, Pos, S
Max: 537244
100
80
60
40
20
0
616.1
617.0
618.1
200
400
600
800
1000
1200
1400
m/z

6NHS

Origin	Bruker BioSpin GmbH
Solvent	CDCl3
Temperature	298.0
Pulse Sequence	zg30
Experiment	1D
Number of Scans	16
Acquisition Date	2023-12-12T14:37:06
Spectrometer Frequency	400.13
Spectral Width	8196.7
Lowest Frequency	-1637.4
Nucleus	1H
Acquired Size	32768
Spectral Size	65536

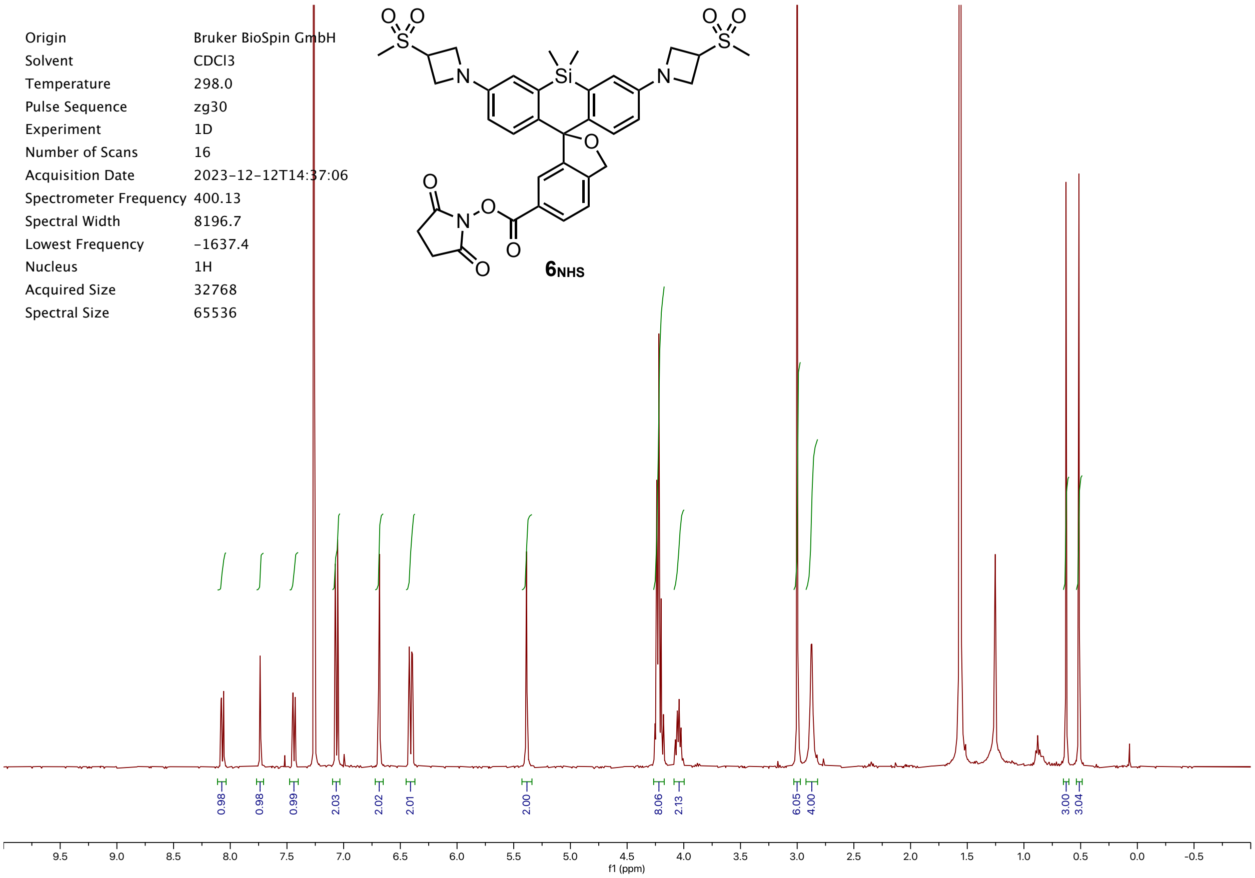

Origin	Bruker BioSpin GmbH
Solvent	CDCl3
Temperature	296.3
Pulse Sequence	zgpg30
Experiment	1D
Number of Scans	2048
Acquisition Date	2023-10-25T20:04:21
Spectrometer Frequency	100.62
Spectral Width	24038.5
Lowest Frequency	-1948.1
Nucleus	13C
Acquired Size	32768
Spectral Size	65536

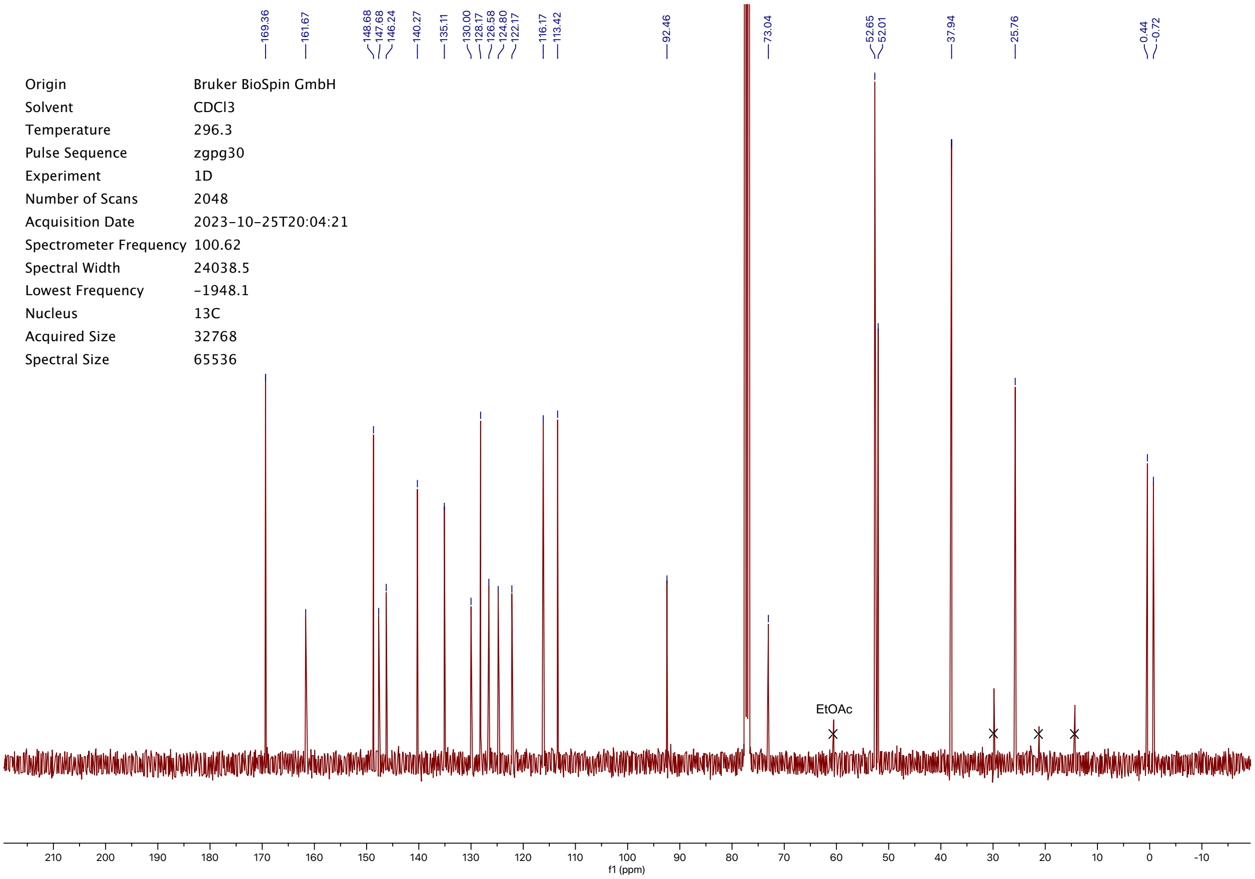

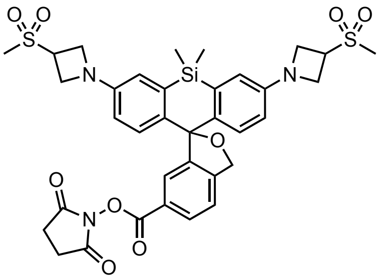
O
O
S
N
Si
N
S
O
O
O
O
O
N
O
O
O

6_{NHS}

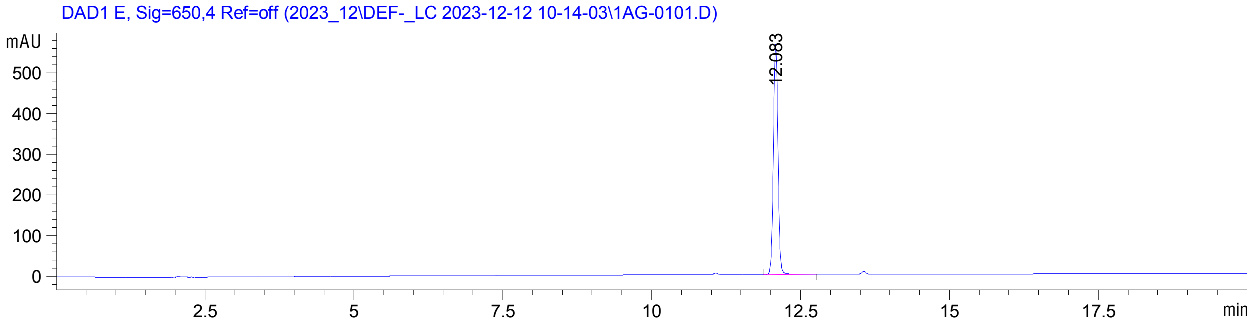
DAD1 E, Sig=650,4 Ref=off (2023_12\DEF-_LC 2023-12-12 10-14-03\1AG-0101.D)
mAU
500
400
300
200
100
0
12.083
2.5
5
7.5
10
12.5
15
17.5
min

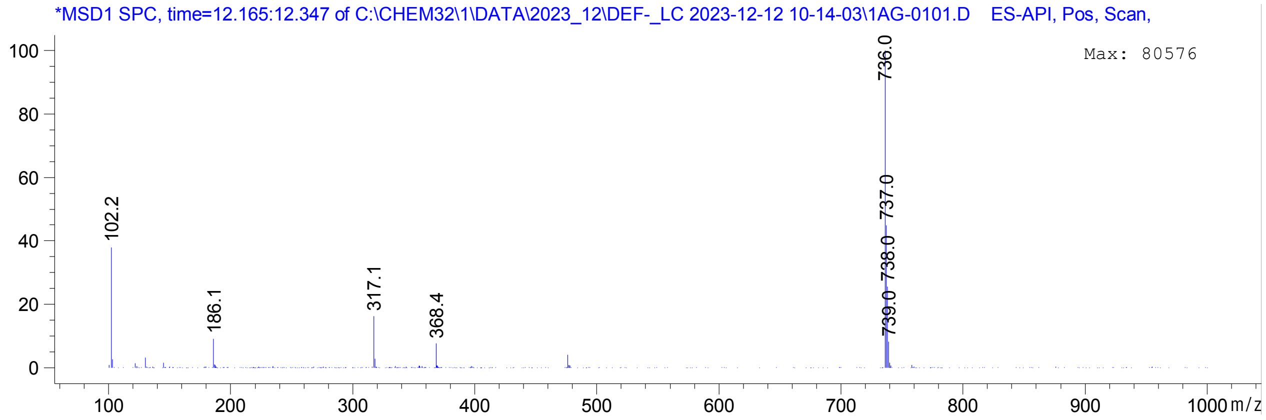
*MSD1 SPC, time=12.165:12.347 of C:\CHEM32\1\DATA\2023_12\DEF-_LC 2023-12-12 10-14-03\1AG-0101.D ES-API, Pos, Scan,
Max: 80576
100
80
60
40
20
0
102.2
186.1
317.1
368.4
736.0
737.0
738.0
739.0
100
200
300
400
500
600
700
800
900
1000
m/z

Origin	Bruker BioSpin GmbH
Solvent	MeOD
Temperature	298.0
Pulse Sequence	zg30
Experiment	1D
Number of Scans	16
Acquisition Date	2023-12-15T10:41:04
Spectrometer Frequency	400.13
Spectral Width	8196.7
Lowest Frequency	-1635.2
Nucleus	1H
Acquired Size	32768
Spectral Size	65536

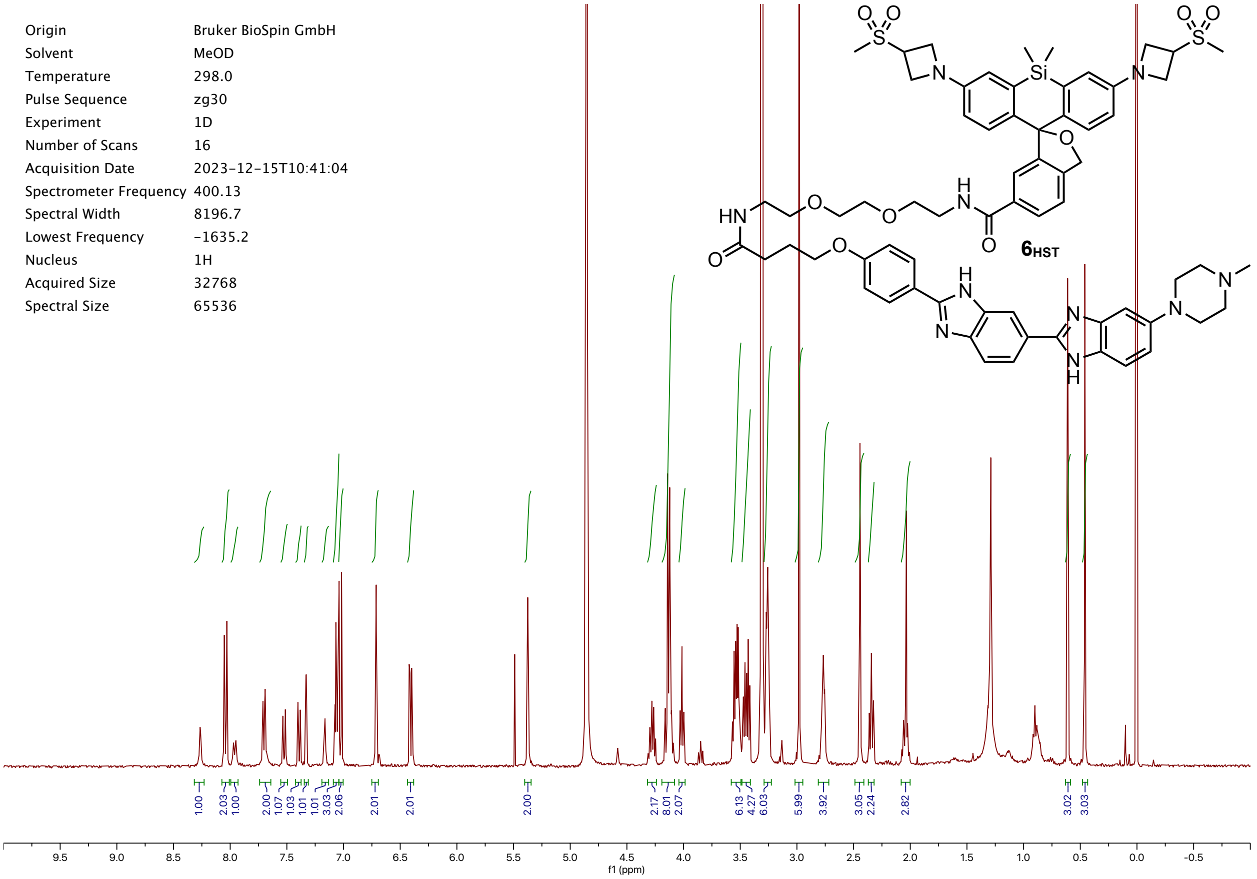

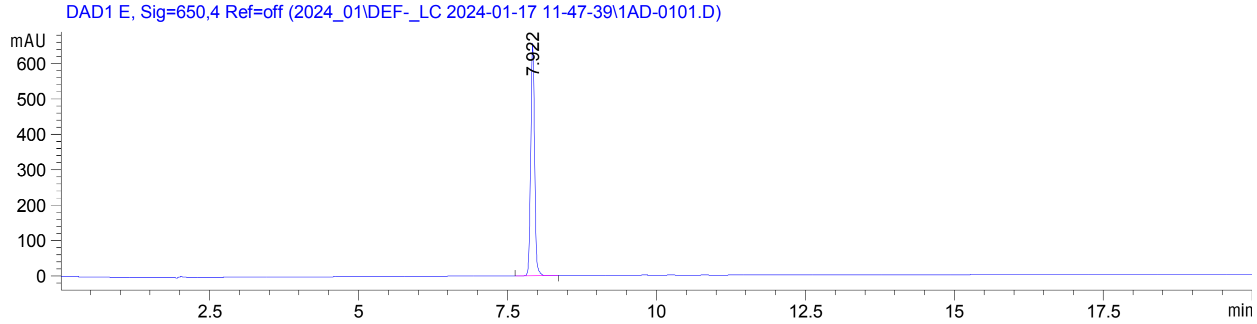

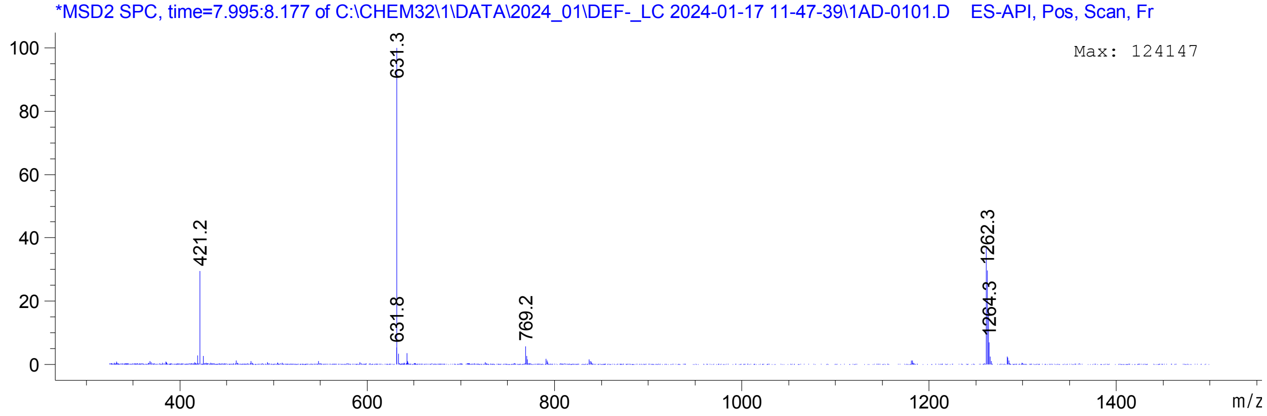

Supplement: Supplementary file 1 — Supplementary Notes 1 and 2. [file 41592_2026_3062_MOESM1_ESM.pdf]
