# Supplementary material for: A series of spontaneously blinking dyes for super-resolution microscopy
Source: Nat Methods. 2026 Apr 15;23(5):909–13. doi: 10.1038/s41592-026-03062-5 (PMC13167466; doi:10.1038/s41592-026-03062-5)
Supplement: Supplementary file 2 — Reporting Summary [file 41592_2026_3062_MOESM2_ESM.pdf]

nature portfolio

Corresponding author(s): Luke D. Lavis

Last updated by author(s): 01/09/2025

Reporting Summary

Nature Portfolio wishes to improve the reproducibility of the work that we publish. This form provides structure for consistency and transparency in reporting. For further information on Nature Portfolio policies, see our Editorial Policies and the Editorial Policy Checklist.

Statistics

For all statistical analyses, confirm that the following items are present in the figure legend, table legend, main text, or Methods section.

n/a	Confirmed	
☐	☒	The exact sample size (*n*) for each experimental group/condition, given as a discrete number and unit of measurement
☐	☒	A statement on whether measurements were taken from distinct samples or whether the same sample was measured repeatedly
☒	☐	The statistical test(s) used AND whether they are one- or two-sided<br>*Only common tests should be described solely by name; describe more complex techniques in the Methods section.*
☒	☐	A description of all covariates tested
☒	☐	A description of any assumptions or corrections, such as tests of normality and adjustment for multiple comparisons
☐	☒	A full description of the statistical parameters including central tendency (e.g. means) or other basic estimates (e.g. regression coefficient) AND variation (e.g. standard deviation) or associated estimates of uncertainty (e.g. confidence intervals)
☒	☐	For null hypothesis testing, the test statistic (e.g. *F*, *t*, *r*) with confidence intervals, effect sizes, degrees of freedom and *P* value noted<br>*Give P values as exact values whenever suitable.*
☒	☐	For Bayesian analysis, information on the choice of priors and Markov chain Monte Carlo settings
☒	☐	For hierarchical and complex designs, identification of the appropriate level for tests and full reporting of outcomes
☒	☐	Estimates of effect sizes (e.g. Cohen's *d*, Pearson's *r*), indicating how they were calculated

Our web collection on statistics for biologists contains articles on many of the points above.

Software and code

Policy information about availability of computer code

Data collection	The Shimadzu analytical HPLC instrument was controlled by Shimadzu LabSolutions 5.106 software. The Shimadzu preparative HPLC instruments were controlled by Shimadzu LabSolutions 5.106 software. The Shimadzu LC/MS system was controlled by LabSolutions 5.120 or 5.98SP1 software. The Agilent LC/MS system was controlled by Agilent LC/MSD ChemStation Rev. B04.03 [54] software. The Biotage Isolera flash chromatography system was controlled by Biotage OS 852M software. The Buchi Rotavapor was running Buchi firmware version 01.02.00.00 with a vacuum pump running Buchi firmware version 01.00.00.00 and controller running Buchi firmware 01.01.00.00. The NMR was controlled by IconNMR 4.7.7 Build 20 software. The Cary Model 100 spectrometer was controlled by CaryWinUV Scan Application 6.4.0.1610 software. The Cary Eclipse was controlled by Cary Eclipse Scan Application 1.2(147) software. The Quantaurus Quantum Yield spectrophotometer was controlled by PLQY software U6039-05 4.6.0. The Zeiss ELYRA microscope was controlled by Zen2.1 SP3 software.
Data analysis	NMR data were analyzed using Bruker TopSpin 3.2 or MestReNova 15.0.0-34764 software. Spectra and other graphical data were analyzed using GraphPad Prism 10 for macOS version 10.2.0 (335) software. Fluorescence microscopy images were processed and analyzed using the software and plug-ins decribed in the Methods section.

For manuscripts utilizing custom algorithms or software that are central to the research but not yet described in published literature, software must be made available to editors and reviewers. We strongly encourage code deposition in a community repository (e.g. GitHub). See the Nature Portfolio guidelines for submitting code & software for further information.

Data

Policy information about availability of data

All manuscripts must include a data availability statement. This statement should provide the following information, where applicable:

- Accession codes, unique identifiers, or web links for publicly available datasets
- A description of any restrictions on data availability
- For clinical datasets or third party data, please ensure that the statement adheres to our policy

The authors declare that the data supporting the findings of this study are available within the paper, its Supplementary Information files, and its Source Data files. Should any raw data files be needed in another format they are available from the corresponding author upon reasonable request.

Human research participants

Policy information about studies involving human research participants and Sex and Gender in Research.

Reporting on sex and gender	*Use the terms sex (biological attribute) and gender (shaped by social and cultural circumstances) carefully in order to avoid confusing both terms. Indicate if findings apply to only one sex or gender; describe whether sex and gender were considered in study design whether sex and/or gender was determined based on self-reporting or assigned and methods used. Provide in the source data disaggregated sex and gender data where this information has been collected, and consent has been obtained for sharing of individual-level data; provide overall numbers in this Reporting Summary. Please state if this information has not been collected. Report sex- and gender-based analyses where performed, justify reasons for lack of sex- and gender-based analysis.*
Population characteristics	*Describe the covariate-relevant population characteristics of the human research participants (e.g. age, genotypic information, past and current diagnosis and treatment categories). If you filled out the behavioural & social sciences study design questions and have nothing to add here, write "See above."*
Recruitment	*Describe how participants were recruited. Outline any potential self-selection bias or other biases that may be present and how these are likely to impact results.*
Ethics oversight	*Identify the organization(s) that approved the study protocol.*

Note that full information on the approval of the study protocol must also be provided in the manuscript.

Field-specific reporting

Please select the one below that is the best fit for your research. If you are not sure, read the appropriate sections before making your selection.

☒ Life sciences ☐ Behavioural & social sciences ☐ Ecological, evolutionary & environmental sciences

For a reference copy of the document with all sections, see nature.com/documents/nr-reporting-summary-flat.pdf

Life sciences study design

All studies must disclose on these points even when the disclosure is negative.

Sample size	All quantitative imaging experiments were performed on replicate samples or fields of view as reported in the Methods and Figure legends.
Data exclusions	No data were excluded from this study.
Replication	All imaging and data were repeated and showed similar results.
Randomization	We compared a relatively small number of compounds allowing the use of cell culture samples that were the same age, treated in the same way, and analyzed by a standard protocol. Randomization was not necessary for these experiments.
Blinding	We compared a relatively small number of compounds allowing the use of cell culture samples that were the same age, treated in the same way, and analyzed by a standard protocol. Blinding was not necessary for these experiments.

Reporting for specific materials, systems and methods

We require information from authors about some types of materials, experimental systems and methods used in many studies. Here, indicate whether each material, system or method listed is relevant to your study. If you are not sure if a list item applies to your research, read the appropriate section before selecting a response.

Materials & experimental systems

n/a	Involved in the study
☒	☐ Antibodies
☐	☒ Eukaryotic cell lines
☒	☐ Palaeontology and archaeology
☒	☐ Animals and other organisms
☒	☐ Clinical data
☒	☐ Dual use research of concern

Methods

n/a	Involved in the study
☒	☐ ChIP-seq
☒	☐ Flow cytometry
☒	☐ MRI-based neuroimaging

Eukaryotic cell lines

Policy information about cell lines and Sex and Gender in Research

Cell line source(s)	COS7, U2OS, hTERT RPE-1, and CHOK-1 cells were from ATCC. The mouse ES JM8.N4 cells, derived from the C57BL/6N mouse strain, were a gift from Robert Tjian's lab (University of California, Berkeley).
Authentication	The COS7, U2OS, hTERT RPE-1, and CHOK-1 cells were authenticated by ATCC and were not re-authenticated in the Legant Lab, the Galbraith Lab, or at Janelia. The JM8.N4 cells were authenticated by short tandem repeat DNA profiling and approved by the NIH 4D Nucleome project as a Tier2 cell line.
Mycoplasma contamination	The Legant Lab, the Galbraith Lab and the Janelia Cell Culture Facility regularly tests for mycoplasma contamination. The cell lines used in this study tested negative.
Commonly misidentified lines (See ICLAC register)	No commonly misidentified cell lines were used in this study.
